# Supplementary material for: Synthesis of Ethyl Pyrimidine-Quinolincarboxylates Selected from Virtual Screening as Enhanced Lactate Dehydrogenase (LDH) Inhibitors
Source: Int J Mol Sci. 2024 Sep 9;25(17):9744. doi: 10.3390/ijms25179744 (PMC11396203; doi:10.3390/ijms25179744)
Supplement: Supplementary file 1 [file ijms-25-09744-s001.zip › ijms-3152455-supplementary.pdf]

SUPPLEMENTARY MATERIAL

Synthesis of Ethyl Pyrimidine-Quinolincarboxylates Selected from Virtual Screening as Enhanced Lactate Dehydrogenase (LDH) Inhibitors

Iván Díaz, M.Sc.; Sofía Salido,Ph.D.; Manuel Nogueras, Ph.D.; Justo Cobo*, Ph.D.;
Facultad de Ciencias Experimentales, Departamento de Química Inorgánica y Orgánica,
Universidad de Jaén, E-23071 Jaén, España.
E–mail: jcobo@ujaen.es

INDEX

1. In-silico studies 3

2. Structure characterization 14

Compound **4** 14

Compound **5** 19

Compound **6** 23

Compound **9** 27

Compound **10** 30

Compound **11** 34

Compound **12** 37

Compound **13a** 40

Compound **13b** 43

Compound **14a** 46

Compound **14b** 49

Compound **15a** 52

Compound **15b** 55

Compound **15c** 58

Compound **15d** 61

Compound **16a** 64

Compound **16b** 68

Compound **16c** 72

Compound **16d** 76

Compound **17a** 80

Compound **17b** 83

Compound **17c** 86

Compound **17d** 89

Compound **18a** 92

Compound **18b** 95

Compound **18c** 98

Compound **18d** 101

3. Biological assays 104

3.1. LDHA dose-response inhibition curves 104

3.2. LDHB dose-response inhibition curves 109

1. In-silico studies

Table S1. Affinity and energy values (kcal/mol) involved in the interaction of esters (**15**-**18**)(**a**-**d**) with the main aminoacid residues when docked in the **W31** site of *h*LDHA. Pose selection was done according to the best affinity value.

Hybrid	Affinity	Arg^{168}	His^{192}	Asn^{137}	Asp^{194}
**15a**	-9.060	-2.8	0.0	0.0	0.0
**15b**	-10.403	0.0	-3.0	-3.3	0.0
**15c**	-9.661	-6.0	0.0	0.0	0.0
**15d**	-10.063	-6.1	0.0	0.0	0.0
**16a**	-9.180	-6.2	0.0	0.0	0.0
**16b**	-10.396	-5.9	0.0	0.0	0.0
**16c**	-9.068	-6.8	0.0	0.0	0.0
**16d**	-9.961	-6.5	0.0	0.0	0.0
**17a**	-9.564	-5.8	0.0	0.0	0.0
**17b**	-9.611	-5.8	0.0	0.0	0.0
**17c**	-9.329	-6.3	0.0	0.0	0.0
**17d**	-10.490	-6.1	0.0	0.0	0.0
**18a**	-9.571	-2.1	0.0	0.0	0.0
**18b**	-9.740	-5.5	0.0	0.0	0.0
**18c**	-9.303	0.0	-7.1	-1.1	0.0
**18d**	-10.470	-6.4	0.0	0.0	0.0

Table S2. Affinity and energy values (kcal/mol) involved in the interaction of esters (**15**-**18**)(**a**-**d**) with the main aminoacid residues when docked in the **NADH** site of *h*LDHA. Pose selection was done according to the best affinity value.

Hybrid	Affinity	Asp^{51}	Val^{135}	Arg^{98}	Val^{30}
**NADH**	-13.280	-7.7	-3.7	-16.9	-5.6
**15a**	-9.092	0.0	0.0	0.0	-0.7
**15b**	-9.728	0.0	0.0	-3.8	0.0
**15c**	-9.570	0.0	0.0	0.0	-0.7
**15d**	-9.027	0.0	0.0	0.0	0.0
**16a**	-9.403	0.0	0.0	0.0	-4.7
**16b**	-9.396	0.0	0.0	0.0	-0.9
**16c**	-9.706	0.0	0.0	0.0	-0.7
**16d**	-9.524	0.0	0.0	-5.1	0.0
**17a**	-9.276	0.0	0.0	0.0	0.0
**17b**	-8.334	0.0	0.0	-4.0	0.0
**17c**	-9.624	0.0	0.0	0.0	0.0
**17d**	-10.180	-1.5	0.0	0.0	-3.2
**18a**	-8.718	0.0	0.0	0.0	0.0
**18b**	-9.742	0.0	0.0	0.0	0.0
**18c**	-9.759	0.0	0.0	-4.5	0.0
**18d**	-10.140	-5.2	0.0	0.0	-2.4

Table S3. Affinity and energy values (kcal/mol) involved in the interaction of esters (**15**-**18**)(**a**-**d**) with the main aminoacid residues when docked in the **extended** site of *h*LDHA. Pose selection was done according to the best affinity value.

Hybrid	Affinity	Arg^{168}	His^{192}	Asn^{137}	Asp^{194}	Asp^{51}	Val^{135}	Arg^{98}	Val^{30}
**15a**	-8.467	0.0	0.0	-2.8	0.0	0.0	0.0	0.0	0.0
**15b**	-8.167	0.0	0.0	0.0	0.0	0.0	0.0	0.0	0.0
**15c**	-8.230	-0.8	0.0	-4.3	0.0	0.0	0.0	0.0	0.0
**15d**	-8.074	0.0	0.0	0.0	0.0	0.0	0.0	0.0	0.0
**16a**	-8.244	0.0	0.0	-0.8	-3.9	0.0	0.0	0.0	0.0
**16b**	-8.183	0.0	0.0	0.0	0.0	0.0	0.0	0.0	0.0
**16c**	-8.279	-0.9	0.0	-4.3	0.0	0.0	0.0	0.0	0.0
**16d**	-8.546	0.0	-2.1	-2.1	0.0	0.0	0.0	0.0	0.0
**17a**	-8.219	0.0	0.0	-2.0	-4.2	0.0	0.0	0.0	0.0
**17b**	-8.434	0.0	0.0	0.0	0.0	0.0	0.0	0.0	0.0
**17c**	-8.436	-0.7	0.0	-4.3	0.0	0.0	0.0	0.0	0.0
**17d**	-8.248	0.0	0.0	0.0	0.0	0.0	0.0	0.0	0.0
**18a**	-8.482	0.0	0.0	-2.7	0.0	0.0	0.0	0.0	0.0
**18b**	-9.198	0.0	-0.9	-3.6	0.0	0.0	0.0	0.0	0.0
**18c**	-8.636	-0.9	0.0	-4.3	0.0	0.0	0.0	0.0	0.0
**18d**	-8.077	0.0	0.0	0.0	0.0	0.0	0.0	0.0	0.0

Table S4. Affinity and energy values (kcal/mol) involved in the interaction of selected pose (according to the reported filtering criteria) for esters (**15**-**18**)(**a**-**d**) with the main aminoacid residues when docked in the **W31** site of *h*LDHA.

R_1	Aryl	Hybrid	Affinity	Arg^{168}	His^{192}	Asn^{137}	Asp^{194}
H	4-ClC_6H_4	**15a**	-9.885	-5.5	0.0	0.0	0.0
	Naphth-2-yl	**15b**	-9.441	-8.7	0.0	0.0	0.0
	C_6H_5	**15c**	-9.477	-5.2	-0.5	0.0	-3.3
	Styril	**15d**	-10.001	-6.4	0.0	0.0	0.0
F	4-ClC_6H_4	**16a**	-9.767	-6.5	0.0	0.0	0.0
	Naphth-2-yl	**16b**	-10.375	-6.0	0.0	0.0	0.0
	C_6H_5	**16c**	-9.084	-6.8	-1.0	0.0	0.0
	Styril	**16d**	-10.106	-6.3	0.0	0.0	0.0
Cl	4-ClC_6H_4	**17a**	-10.052	-6.2	0.0	0.0	0.0
	Naphth-2-yl	**17b**	-10.440	-6.4	0.0	0.0	0.0
	C_6H_5	**17c**	-9.568	-7.6	0.0	0.0	-0.6
	Styril	**17d**	-10.194	-6.4	0.0	0.0	0.0
Br	4-ClC_6H_4	**18a**	-9.919	-4.8	-0.6	0.0	0.0
	Naphth-2-yl	**18b**	-10.214	-5.7	0.0	0.0	-0.5
	C_6H_5	**18c**	-9.836	-6.4	0.0	0.0	0.0
	Styril	**18d**	-10.150	-6.4	0.0	0.0	0.0

Table S5. Affinity and energy values (kcal/mol) involved in the interaction of selected pose for esters (**15**-**18**)(**a**-**d**) with the main aminoacid residues when docked in the **extended** site of *h*LDHB.

Hybrid	Affinity	Asp^{53}	Val^{99}	Val^{137}	Ser^{162}	Gln^{31}	Gly^{98}	Arg^{100}	Val^{32}	Asn^{139}
**NADH**	-13.805	-8.7	-2.0	-4.5	-0.9	-5.8	0.0	-14.8	-6.2	-4.9
**15a**	-8.709	-0.5	0.0	0.0	0.0	0.0	0.0	0.0	0.0	0.0
**15b**	-8.790	0.0	0.0	0.0	0.0	0.0	0.0	-0.6	0.0	0.0
**15c**	-7.533	0.0	-0.6	0.0	0.0	0.0	-4.0	0.0	0.0	0.0
**15d**	-7.501	0.0	0.0	0.0	0.0	0.0	-6.4	0.0	0.0	0.0
**16a**	-8.219	0.0	0.0	0.0	0.0	0.0	-2.9	0.0	0.0	0.0
**16b**	-8.347	0.0	0.0	0.0	0.0	0.0	-2.1	0.0	0.0	0.0
**16c**	-8.130	0.0	0.0	0.0	0.0	0.0	-4.4	0.0	0.0	0.0
**16d**	-7.533	-0.6	0.0	0.0	0.0	-3.4	0.0	0.0	0.0	0.0
**17a**	-8.095	0.0	0.0	0.0	0.0	0.0	-5.4	0.0	0.0	0.0
**17b**	-8.241	0.0	0.0	0.0	0.0	0.0	-3.7	0.0	0.0	0.0
**17c**	-7.770	0.0	0.0	0.0	0.0	0.0	-7.0	0.0	0.0	0.0
**17d**	-7.727	0.0	0.0	0.0	0.0	0.0	-6.7	0.0	0.0	0.0
**18a**	-8.849	-0.7	0.0	0.0	0.0	0.0	0.0	-2.9	0.0	0.0
**18b**	-9.286	-0.5	0.0	0.0	0.0	0.0	-2.5	0.0	-4.4	0.0
**18c**	-7.302	0.0	0.0	0.0	0.0	0.0	-3.9	0.0	0.0	0.0
**18d**	-7.791	0.0	0.0	0.0	0.0	-3.3	0.0	-2.8	0.0	0.0

Table S6. Affinity and energy values (kcal/mol) involved in the interaction of selected pose for esters (**15**-**18**)(**a**-**d**) with the main aminoacid residues when docked in the **H1U** site of *h*LDHB.

Hybrid	Affinity	Glu214	Ser203	Ser211	Lys310
**15a**	-6.883	-7.2	0.0	0.0	-0.5
**15b**	-7.560	-6.8	0.0	0.0	-6.4
**15c**	-7.117	-5.0	0.0	-3.8	0.0
**15d**	-7.126	-7.1	0.0	0.0	0.0
**16a**	-7.840	-9.8	0.0	0.0	-11.1
**16b**	-7.742	-7.1	0.0	0.0	-0.5
**16c**	-6.707	-7.6	0.0	-0.5	-6.2
**16d**	-7.260	-6.4	0.0	0.0	0.0
**17a**	-7.994	-9.7	0.0	0.0	-8.4
**17b**	-6.921	-8.2	0.0	0.0	-6.9
**17c**	-6.884	-6.8	0.0	0.0	-7.3
**17d**	-7.348	-6.2	0.0	0.0	-1.4
**18a**	-8.057	-9.6	0.0	0.0	-10.6
**18b**	-7.036	-8.1	0.0	0.0	-7.7
**18c**	-7.049	-6.8	0.0	0.0	-7.3
**18d**	-6.772	-8.1	0.0	0.0	-7.1

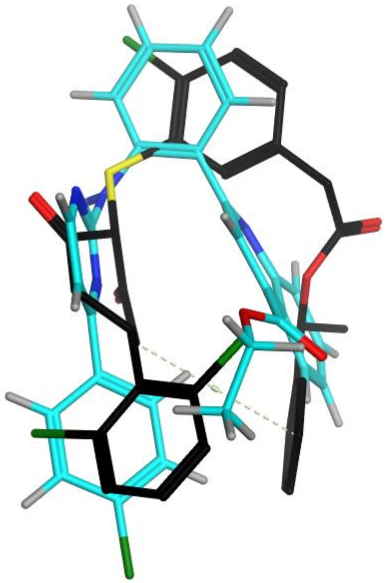

Figure S1. Pose comparison of **15a** (turquoise) and reference **W31** (black) in the *h*LDHA active site.

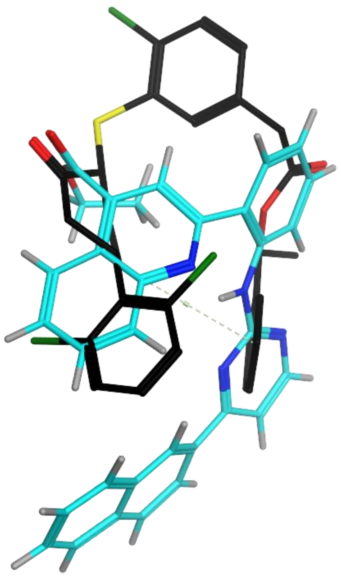

Figure S2. Pose comparison of **15b** (turquoise) and reference **W31** (black) in the *h*LDHA active site.

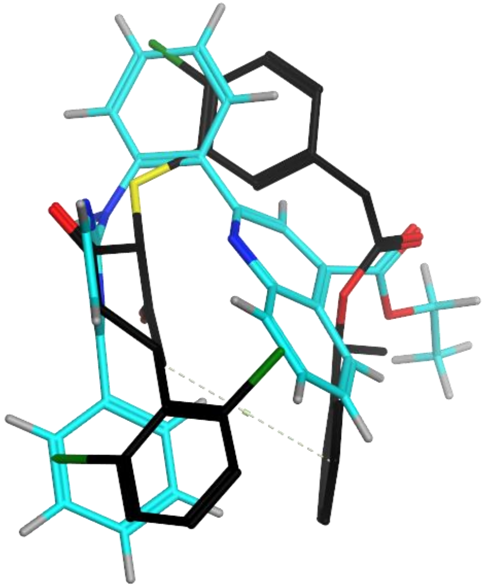

Figure S3. Pose comparison of **15c** (turquoise) and reference **W31** (black) in the *h*LDHA active site.

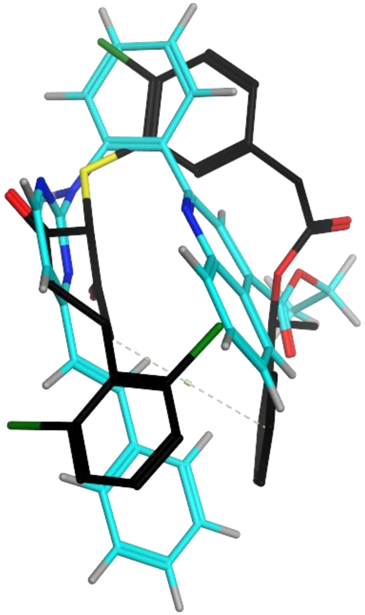

Figure S4. Pose comparison of **15d** (turquoise) and reference **W31** (black) in the *h*LDHA active site.

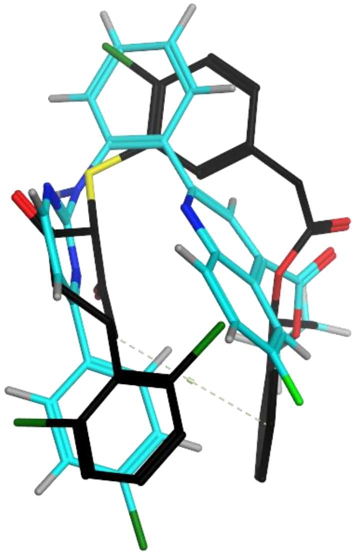

Figure S5. Pose comparison of **16a** (turquoise) and reference **W31** (black) in the *h*LDHA active site.

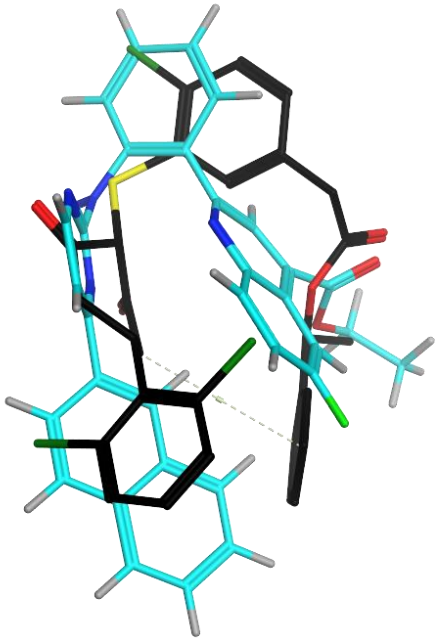

Figure S6. Pose comparison of **16b** (turquoise) and reference **W31** (black) in the *h*LDHA active site.

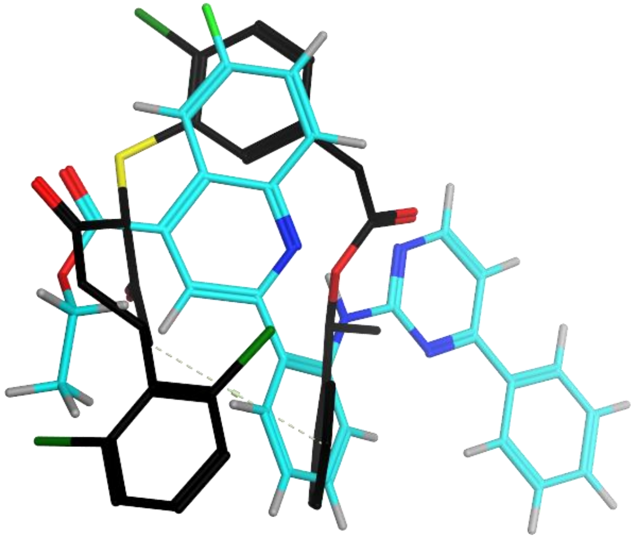

Figure S7. Pose comparison of **16c** (turquoise) and reference **W31** (black) in the *h*LDHA active site.

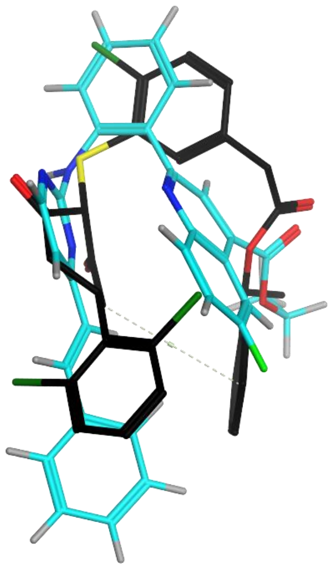

Figure S8. Pose comparison of **16d** (turquoise) and reference **W31** (black) in the *h*LDHA active site.

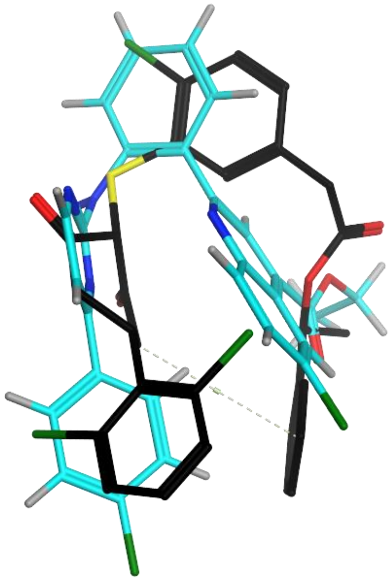

Figure S9. Pose comparison of **17a** (turquoise) and reference **W31** (black) in the *h*LDHA active site.

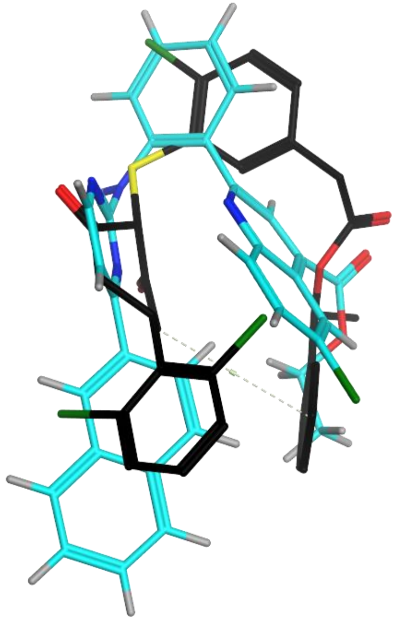

Figure S10. Pose comparison of **17b** (turquoise) and reference **W31** (black) in the *h*LDHA active site.

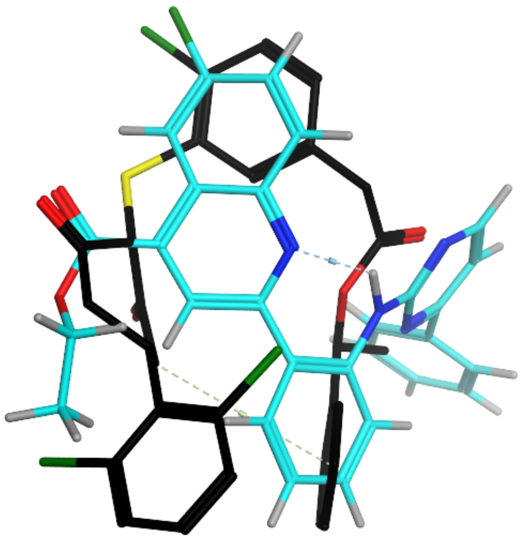

Figure S11. Pose comparison of **17c** (turquoise) and reference **W31** (black) in the *h*LDHA active site.

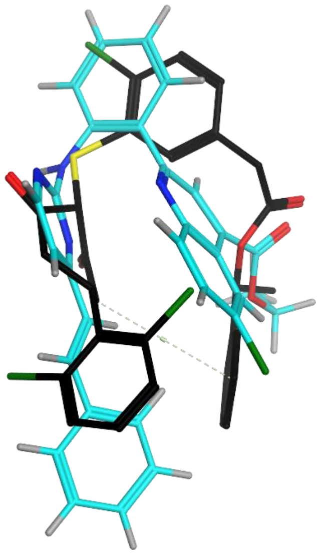

Figure S12. Pose comparison of **17d** (turquoise) and reference **W31** (black) in the *h*LDHA active site.

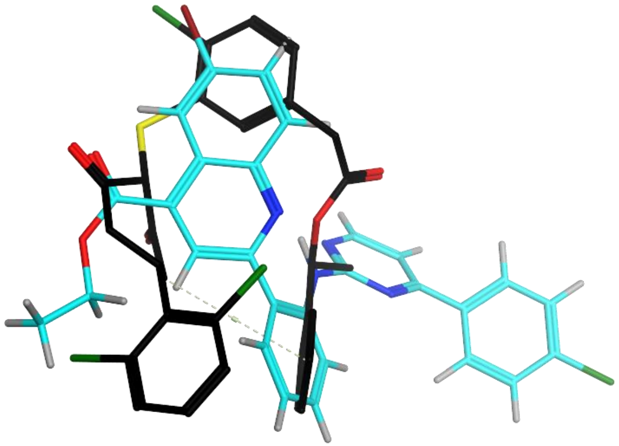

Figure S13. Pose comparison of **18a** (turquoise) and reference **W31** (black) in the *h*LDHA active site.

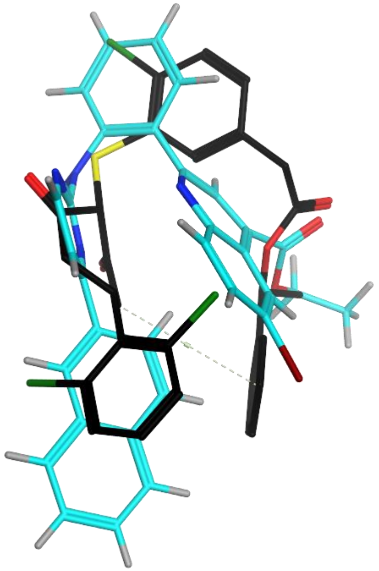

Figure S14. Pose comparison of **18b** (turquoise) and reference **W31** (black) in the *h*LDHA active site.

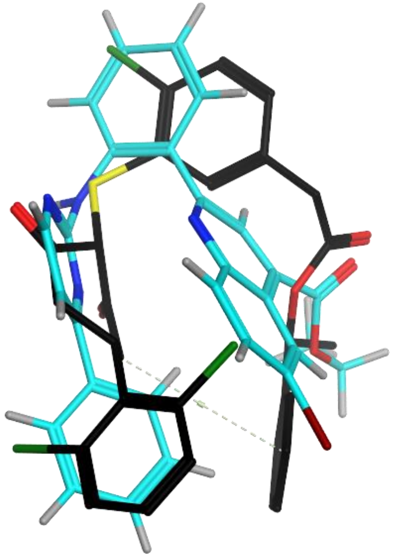

Figure S15. Pose comparison of **18c** (turquoise) and reference **W31** (black) in the *h*LDHA active site.

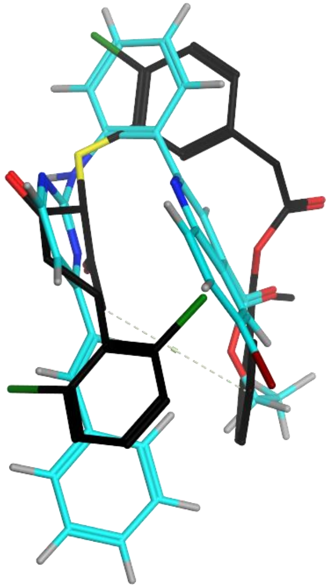

Figure S16. Pose comparison of **18d** (turquoise) and reference **W31** (black) in the *h*LDHA active site.

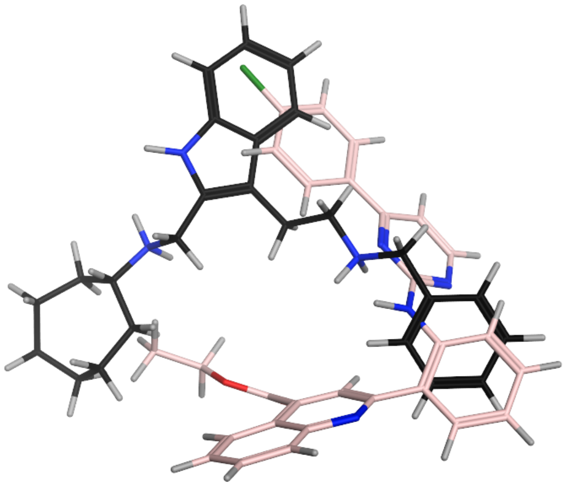

Figure S17. Pose comparison of **15a** (pink) and reference **H1U** (black) in the *h*LDHB allosteric site.

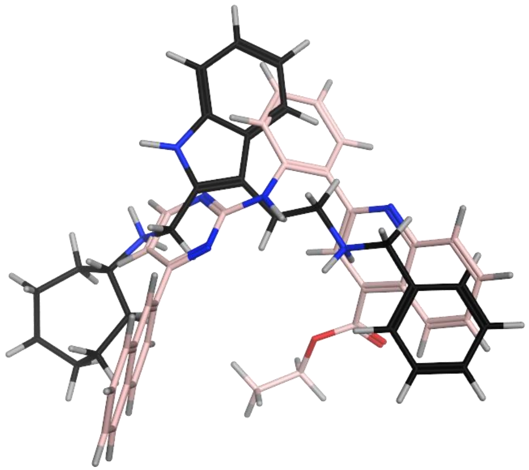

Figure S18. Pose comparison of **15b** (pink) and reference **H1U** (black) in the *h*LDHB allosteric site.

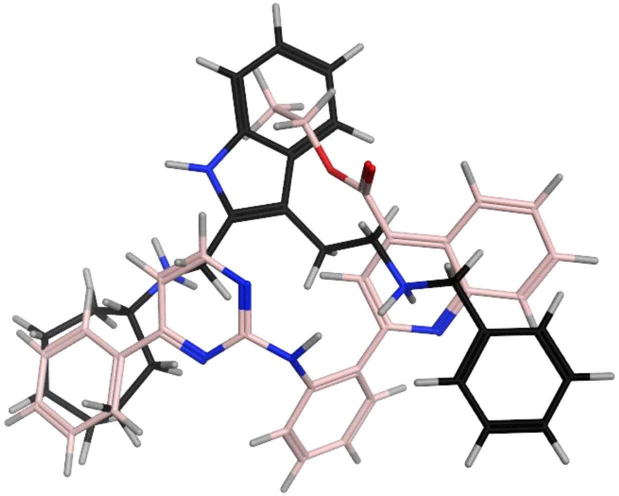

Figure S19. Pose comparison of **15c** (pink) and reference **H1U** (black) in the *h*LDHB allosteric site.

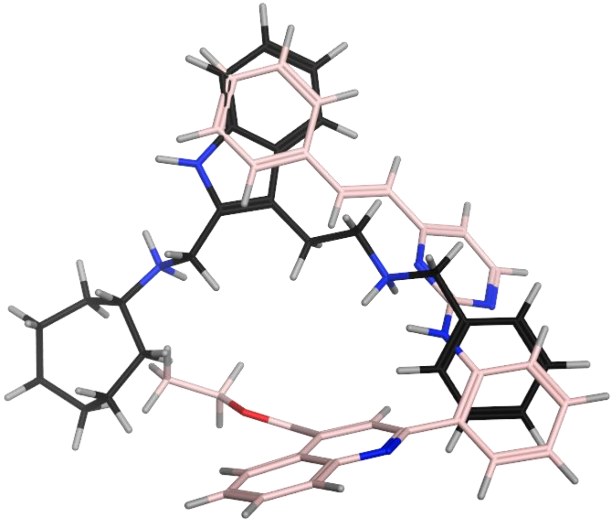

Figure S20. Pose comparison of **15d** (pink) and reference **H1U** (black) in the *h*LDHB allosteric site.

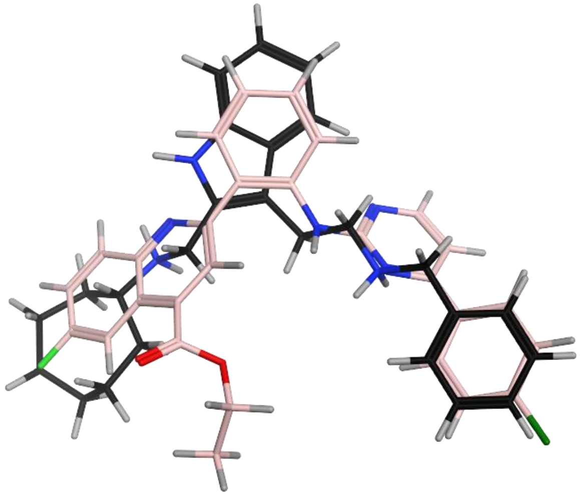

Figure S21. Pose comparison of **16a** (pink) and reference **H1U** (black) in the *h*LDHB allosteric site.

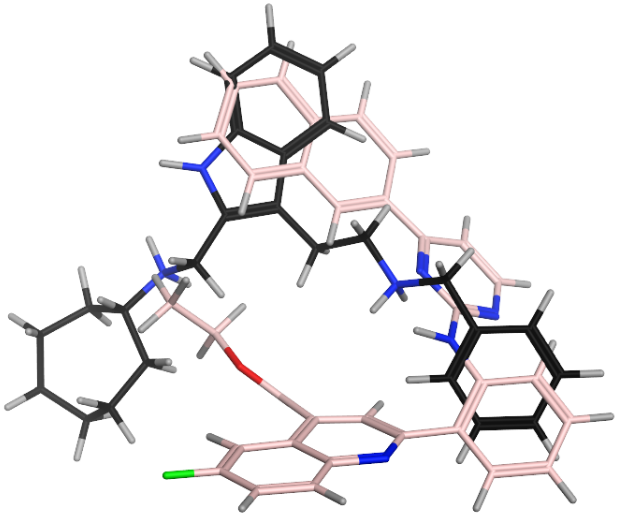

Figure S22. Pose comparison of **16b** (pink) and reference **H1U** (black) in the *h*LDHB allosteric site.

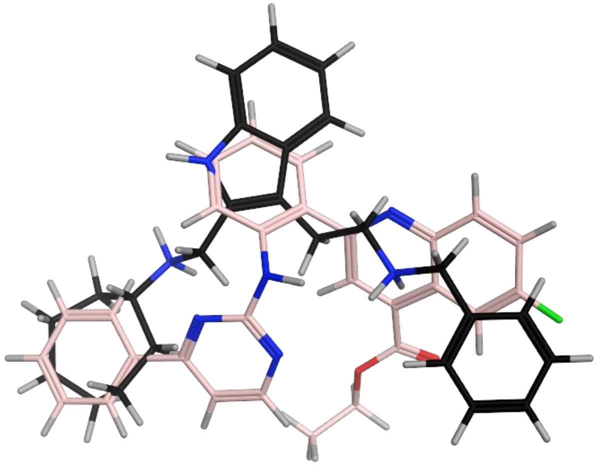

Figure S23. Pose comparison of **16c** (pink) and reference **H1U** (black) in the *h*LDHB allosteric site.

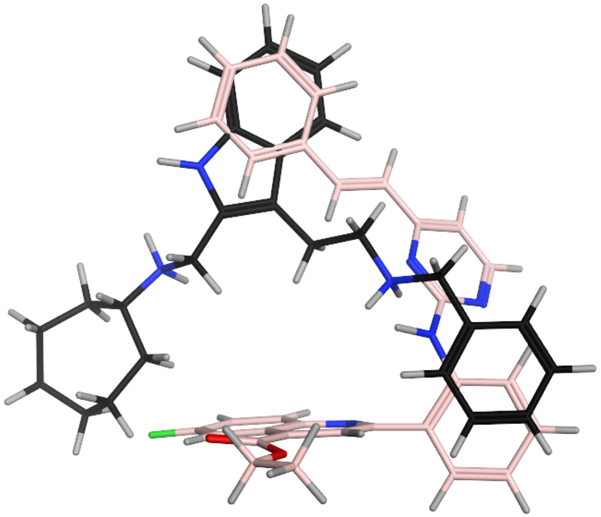

Figure S24. Pose comparison of **16d** (pink) and reference **H1U** (black) in the *h*LDHB allosteric site.

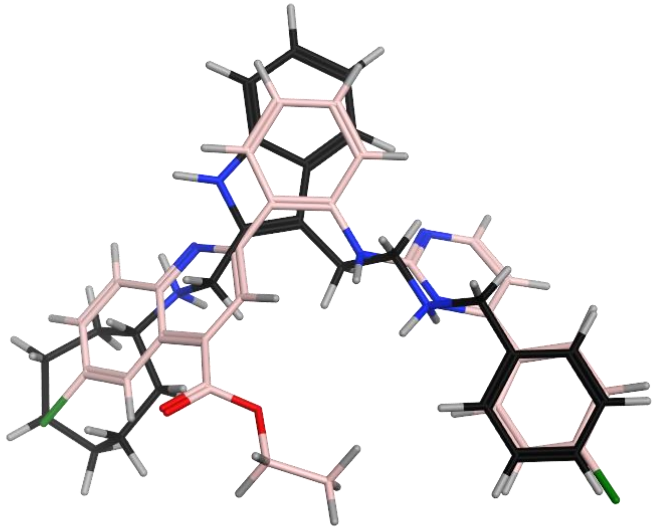

Figure S25. Pose comparison of **17a** (pink) and reference **H1U** (black) in the *h*LDHB allosteric site.

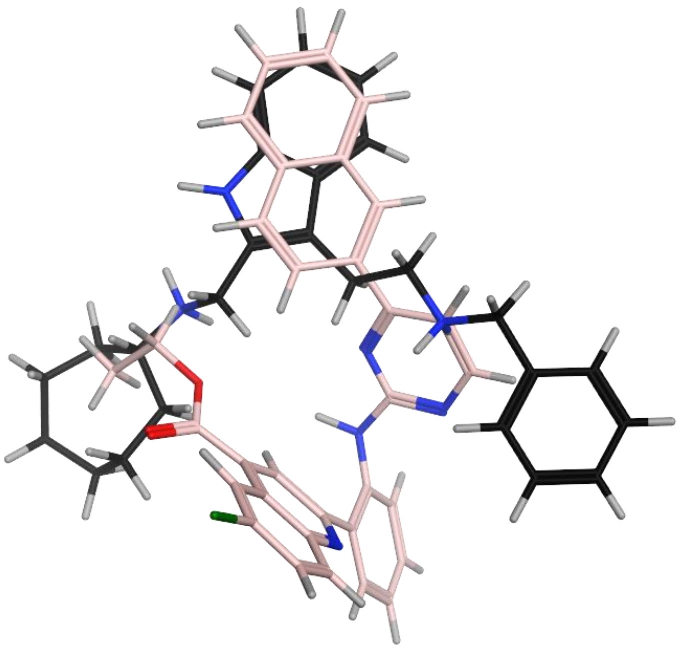

Figure S26. Pose comparison of **17b** (pink) and reference **H1U** (black) in the *h*LDHB allosteric site.

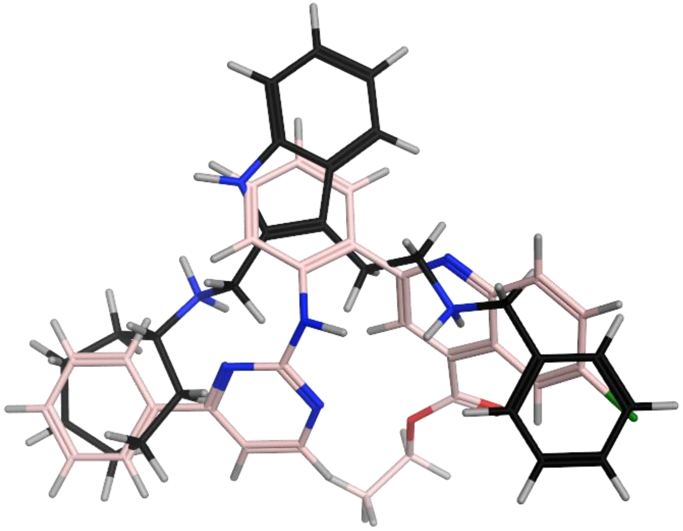

Figure S27. Pose comparison of **17c** (pink) and reference **H1U** (black) in the *h*LDHB allosteric site.

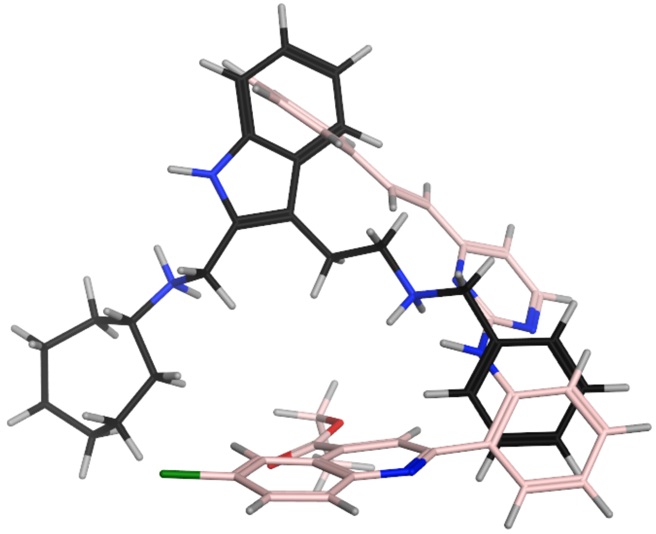

Figure S28. Pose comparison of **17d** (pink) and reference **H1U** (black) in the *h*LDHB allosteric site.

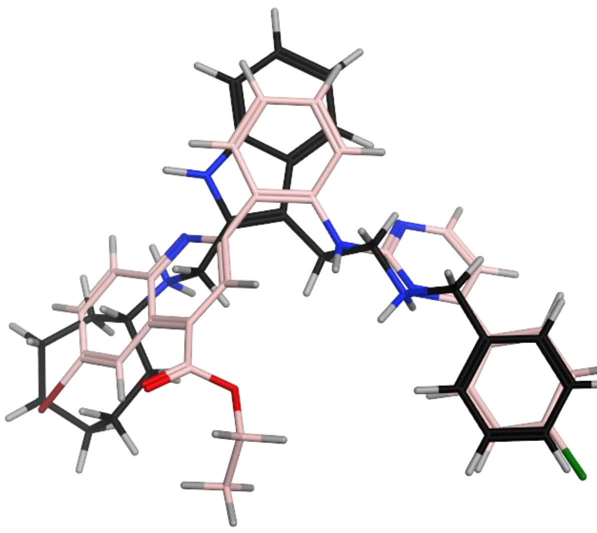

Figure S29. Pose comparison of **18a** (pink) and reference **H1U** (black) in the *h*LDHB allosteric site.

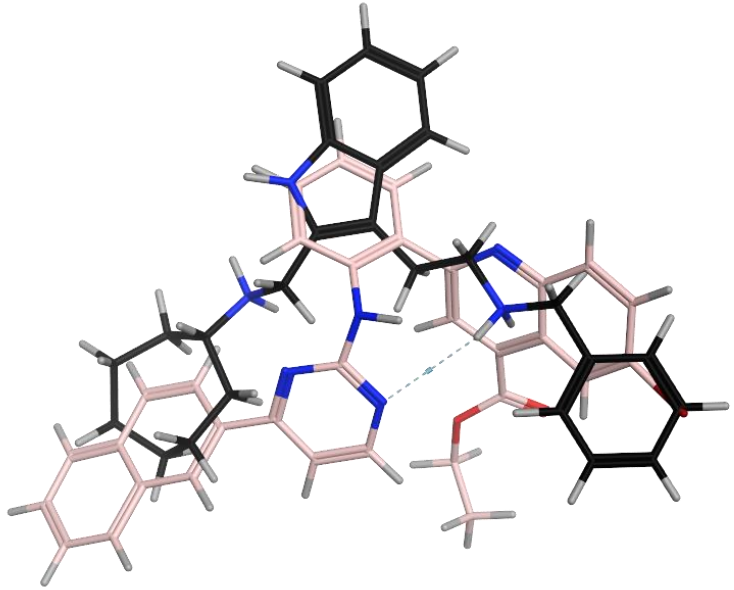

Figure S30. Pose comparison of **18b** (pink) and reference **H1U** (black) in the *h*LDHB allosteric site.

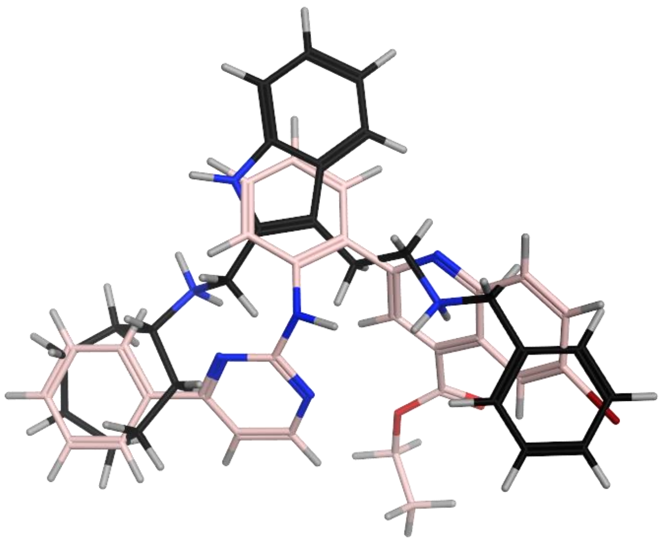

Figure S31. Pose comparison of **18c** (pink) and reference **H1U** (black) in the *h*LDHB allosteric site.

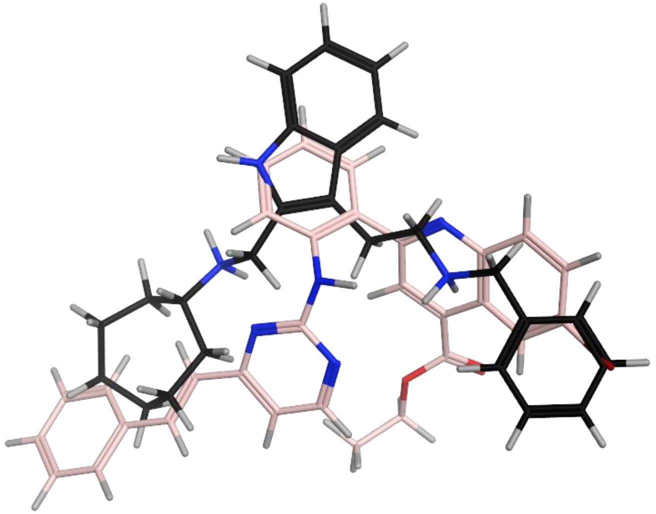

Figure S32. Pose comparison of **18d** (pink) and reference **H1U** (black) in the *h*LDHB allosteric site.

2. Structure characterization

Compound **4**

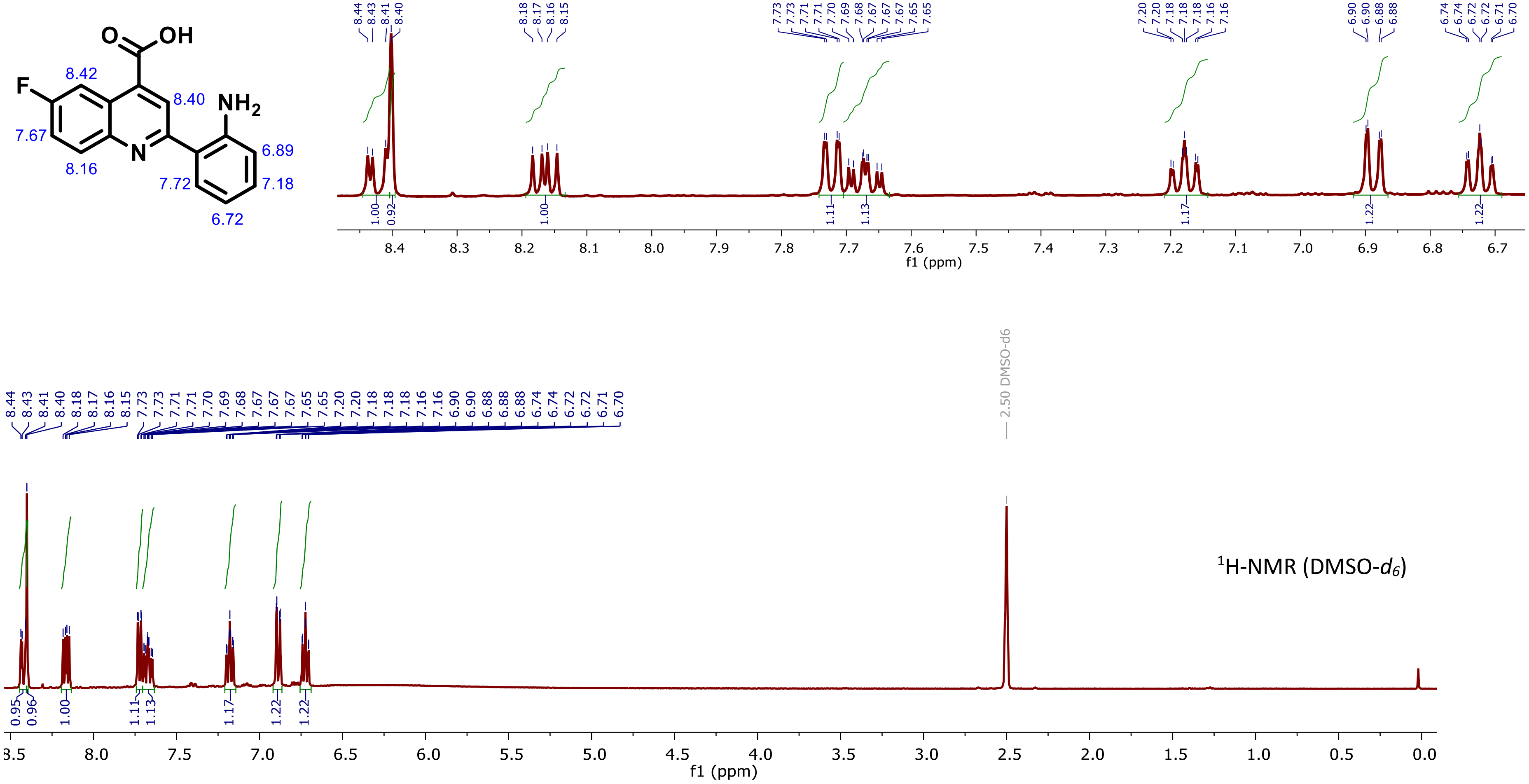

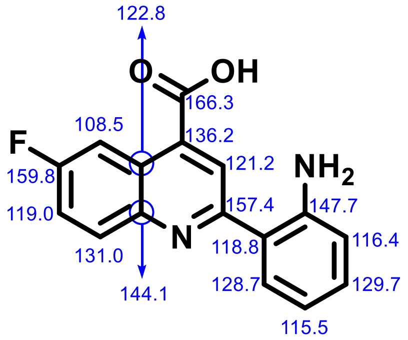
122.8
O
OH
166.3
136.2
108.5
F
159.8
121.2
NH2
157.4
147.7
119.0
N
118.8
116.4
131.0
144.1
128.7
129.7
115.5

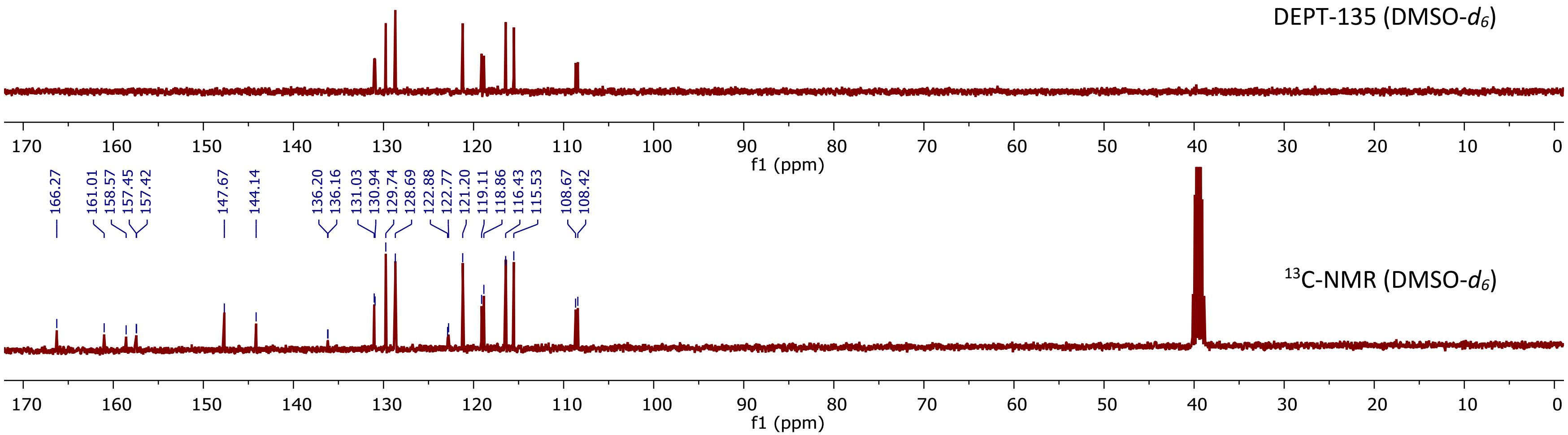
DEPT-135 (DMSO-d6)
170 160 150 140 130 120 110 100 90 80 70 60 50 40 30 20 10 0
f1 (ppm)
166.27
161.01
158.57
157.45
157.42
147.67
144.14
136.20
136.16
131.03
130.94
129.74
128.69
122.88
122.77
121.20
119.11
118.86
116.43
115.53
108.67
108.42
13C-NMR (DMSO-d6)
170 160 150 140 130 120 110 100 90 80 70 60 50 40 30 20 10 0
f1 (ppm)

^{13}C-^{19}F couplings in ^{13}C-NMR. Only coupled peaks have been represented to make it easier to understand.

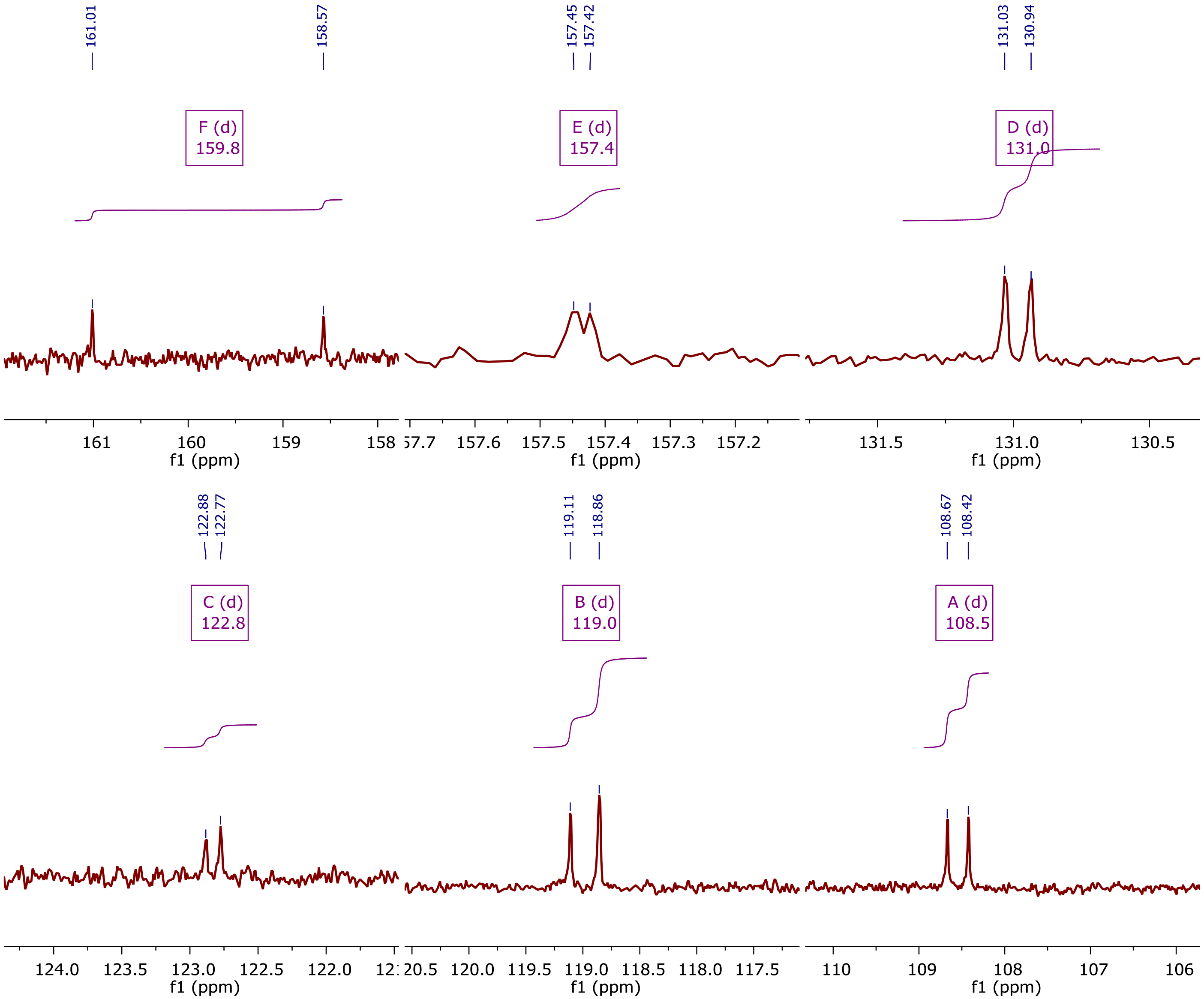

EI MS (70eV)

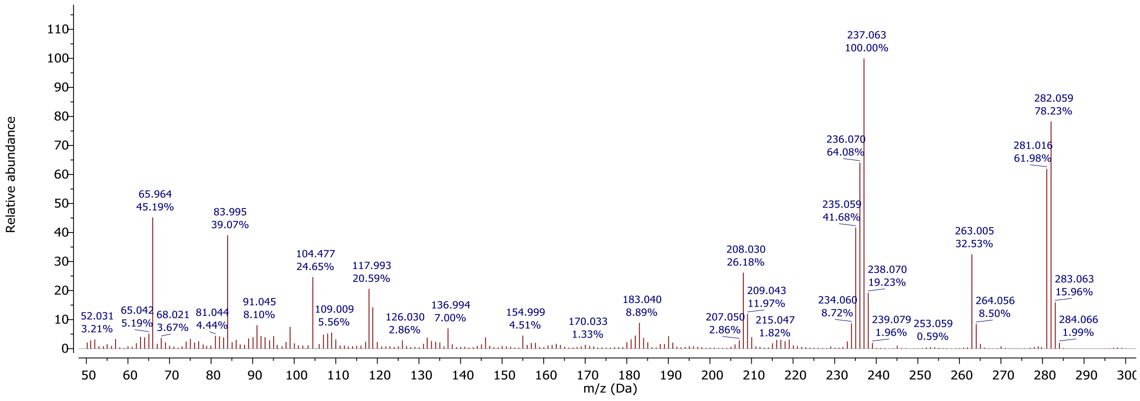

ESI-QTOF (positive ionization)

MS Zoomed Spectrum

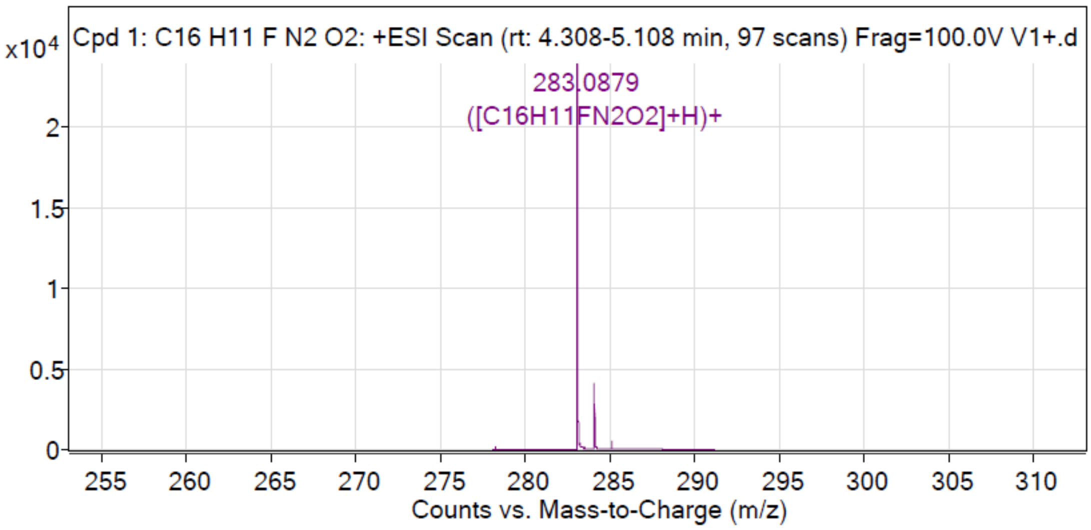

MS Spectrum Peak List

m/z	Calc *m/z*	Diff(ppm)	z	Abund	Formula	Ion
283.0879	283.0877	-0.65	1	23979.17	C16H11FN2O2	(M+H)+
284.0912	284.0909	-1.16	1	4124.12	C16H11FN2O2	(M+H)+
285.0938	285.0936	-0.82	1	465.64	C16H11FN2O2	(M+H)+
286.097	286.0961	-2.9	1	46.11	C16H11FN2O2	(M+H)+

ESI-QTOF (negative ionization)

MS Zoomed Spectrum

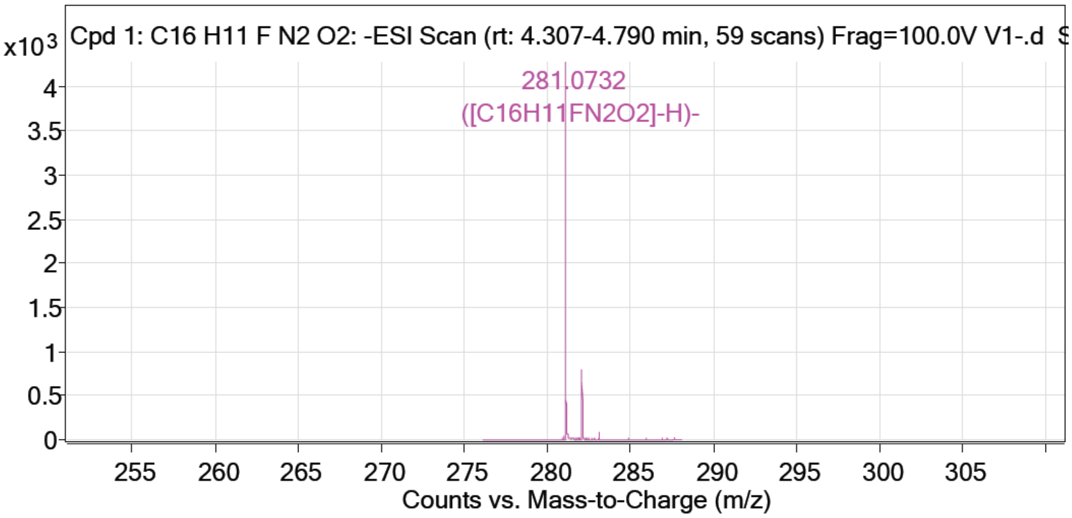

MS Spectrum Peak List

m/z	Calc *m/z*	Diff(ppm)	z	Abund	Formula	Ion
281.0732	281.0732	0.1	1	4354.5	C16H11FN2O2	(M-H)-
282.0763	282.0763	-0.05	1	808.67	C16H11FN2O2	(M-H)-
283.0795	283.079	-1.84	1	89.17	C16H11FN2O2	(M-H)-

IR

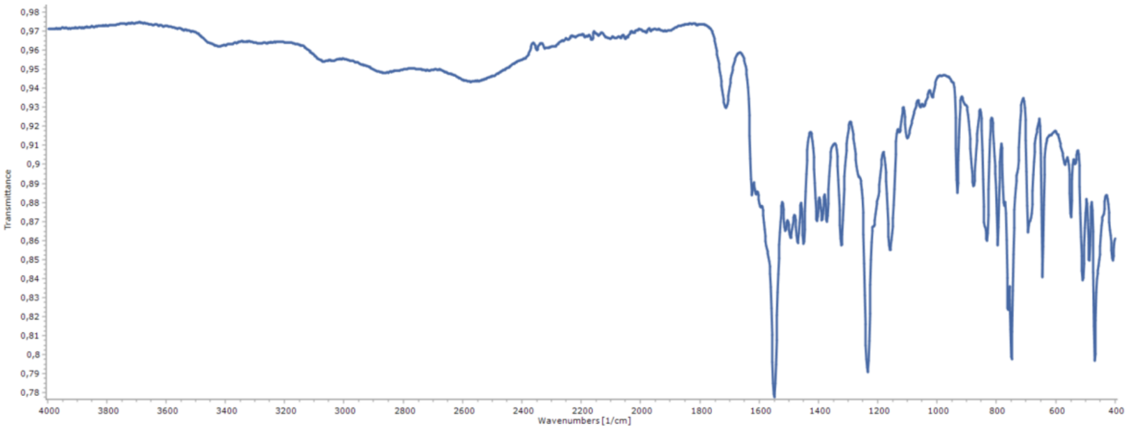

Compound **5**

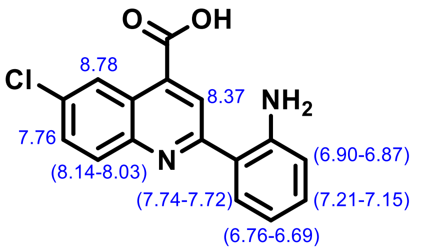

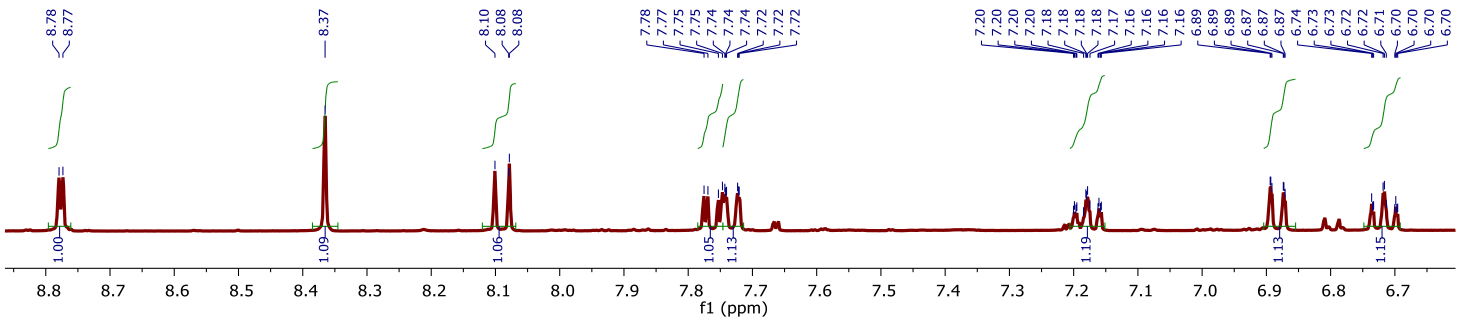

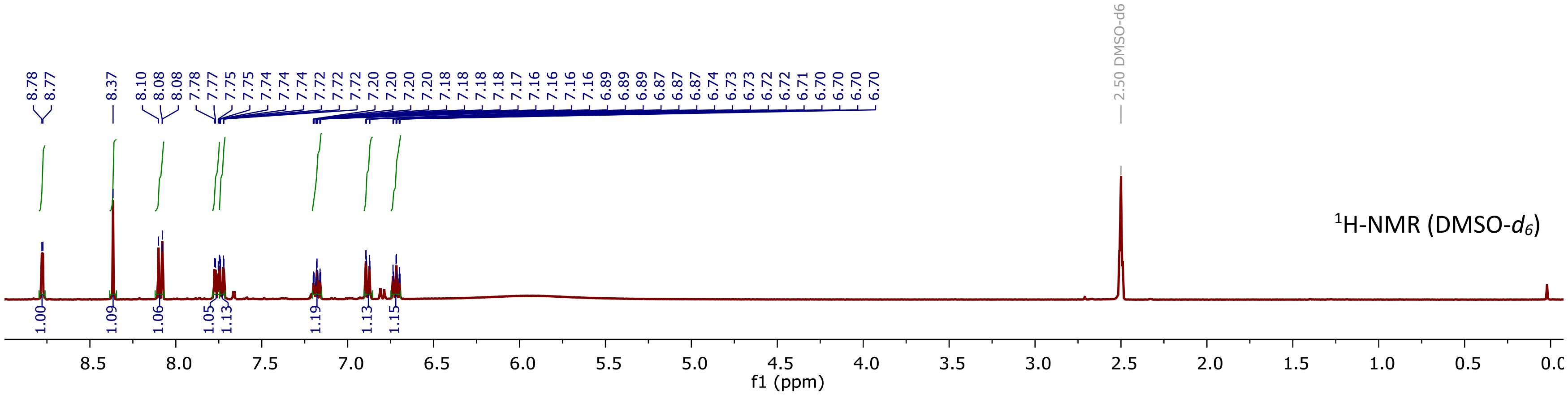

^{1}H-NMR (DMSO-d_6)

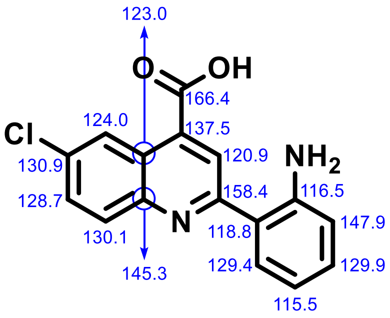

123.0
O
OH
166.4
124.0
137.5
Cl
130.9
120.9
NH2
128.7
158.4
116.5
N
147.9
130.1
118.8
145.3
129.4
129.9
115.5

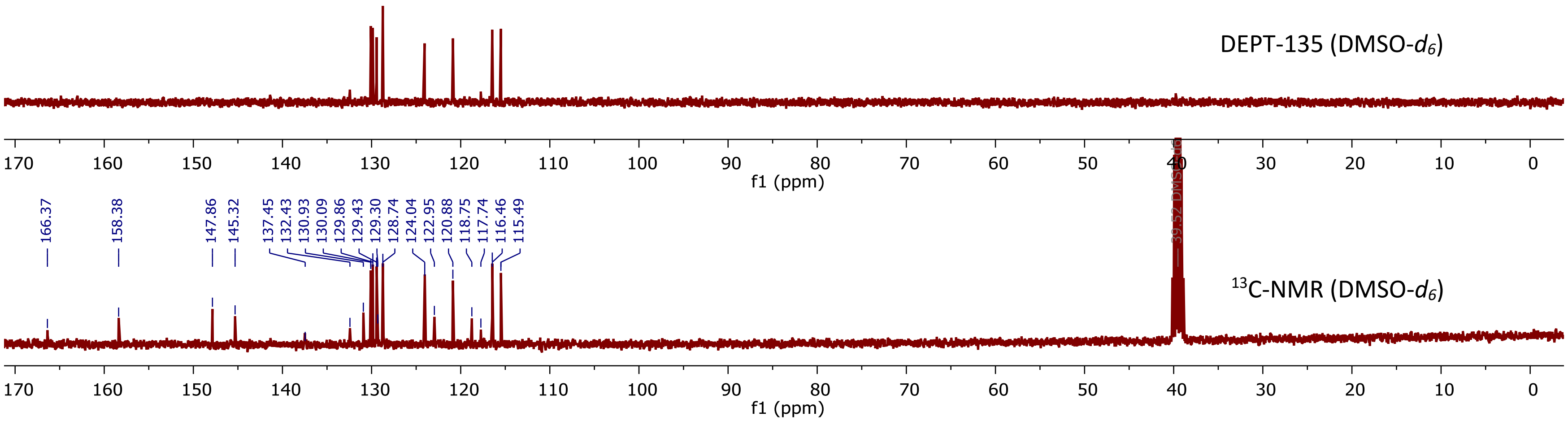

DEPT-135 (DMSO-d6)
170 160 150 140 130 120 110 100 90 80 70 60 50 40 30 20 10 0
f1 (ppm)
166.37
158.38
147.86
145.32
137.45
132.43
130.93
130.09
129.86
129.43
129.30
128.74
124.04
122.95
120.88
118.75
117.74
116.46
115.49
39.52 DMSO-d6
13C-NMR (DMSO-d6)
170 160 150 140 130 120 110 100 90 80 70 60 50 40 30 20 10 0
f1 (ppm)

EI MS (70eV)

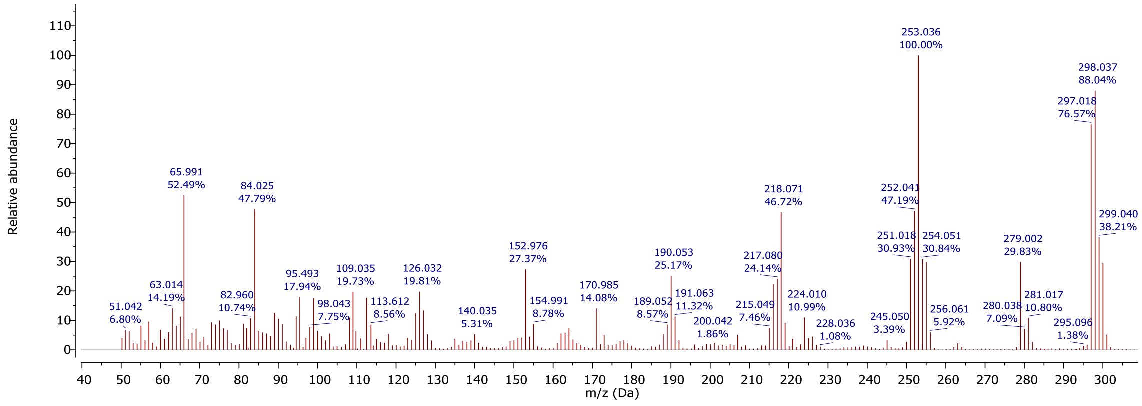

ESI-QTOF (positive ionization)

MS Zoomed Spectrum

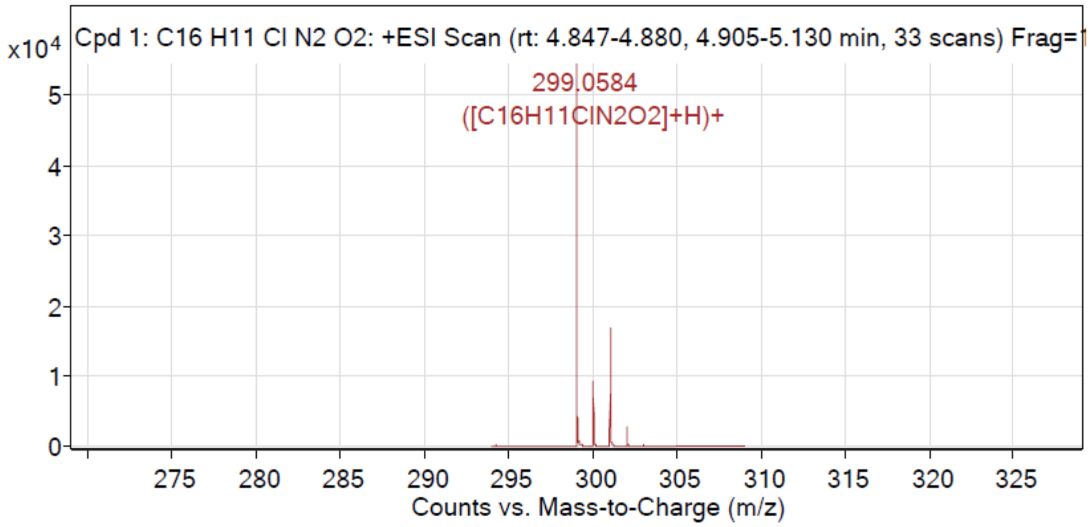

MS Spectrum Peak List

m/z	Calc *m/z*	Diff(ppm)	z	Abund	Formula	Ion
299.0584	299.0582	-0.58	1	54928.41	C16H11ClN2O2	(M+H)+
300.0614	300.0613	-0.18	1	9197.05	C16H11ClN2O2	(M+H)+
301.0557	301.0557	-0.02	1	17015.27	C16H11ClN2O2	(M+H)+
302.0587	302.0586	-0.52	1	2882.98	C16H11ClN2O2	(M+H)+
303.0614	303.0612	-0.71	1	338.4	C16H11ClN2O2	(M+H)+
304.0634	304.0637	1.23	1	36.11	C16H11ClN2O2	(M+H)+

ESI-QTOF (negative ionization)

MS Zoomed Spectrum

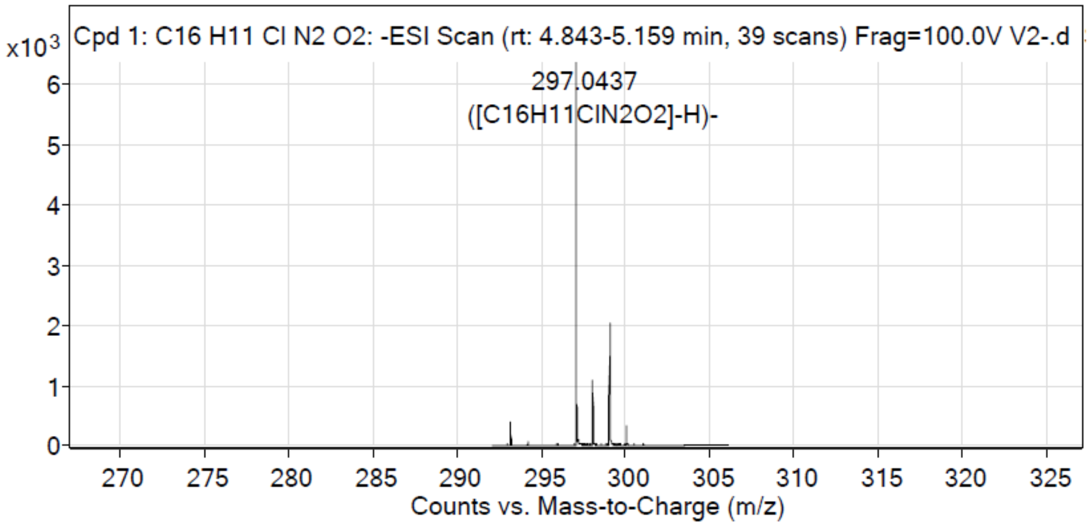

MS Spectrum Peak List

m/z	Calc *m/z*	Diff(ppm)	z	Abund	Formula	Ion
297.0437	297.0436	-0.15	1	6371.53	C16H11ClN2O2	(M-H)-
298.0471	298.0468	-1.04	1	1097.88	C16H11ClN2O2	(M-H)-
299.0413	299.0412	-0.51	1	2092.87	C16H11ClN2O2	(M-H)-
300.0437	300.044	0.94	1	342.55	C16H11ClN2O2	(M-H)-
301.0487	301.0466	-6.76	1	31.24	C16H11ClN2O2	(M-H)-

IR

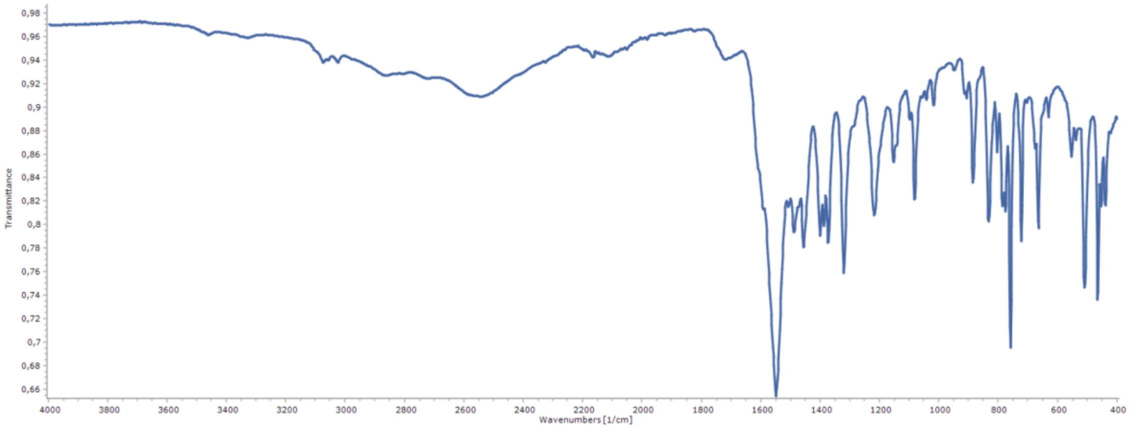

Compound **6**

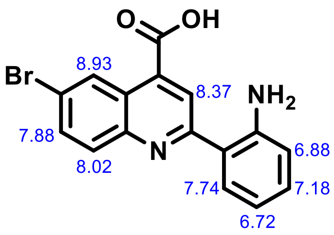

O
OH
Br
8.93
8.37
NH2
7.88
N
6.88
8.02
7.74
7.18
6.72

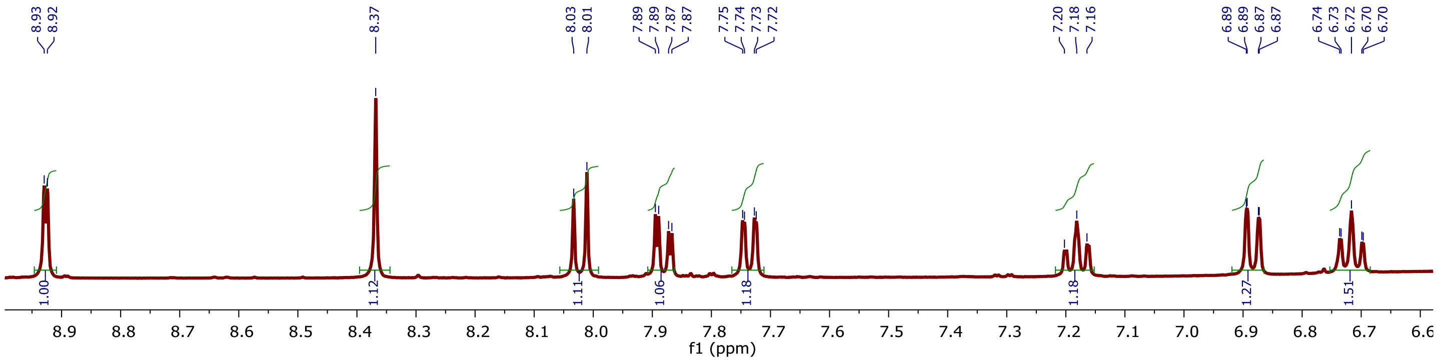

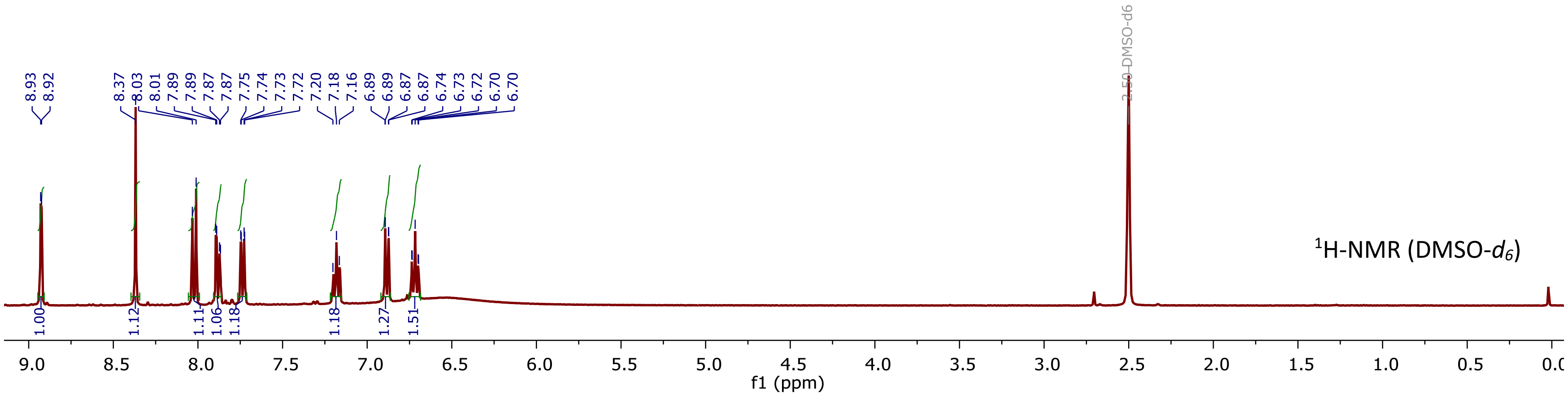

2.50 DMSO-d6
1H-NMR (DMSO-d6)
f1 (ppm)

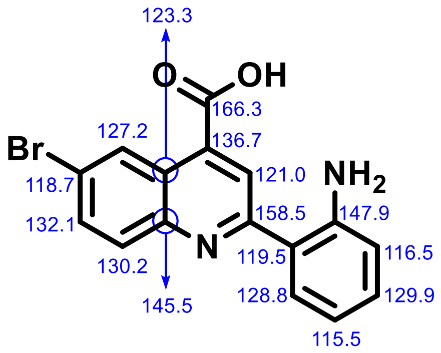

123.3
O
OH
166.3
136.7
127.2
Br
121.0
NH2
118.7
158.5
147.9
132.1
N
119.5
116.5
130.2
145.5
128.8
129.9
115.5

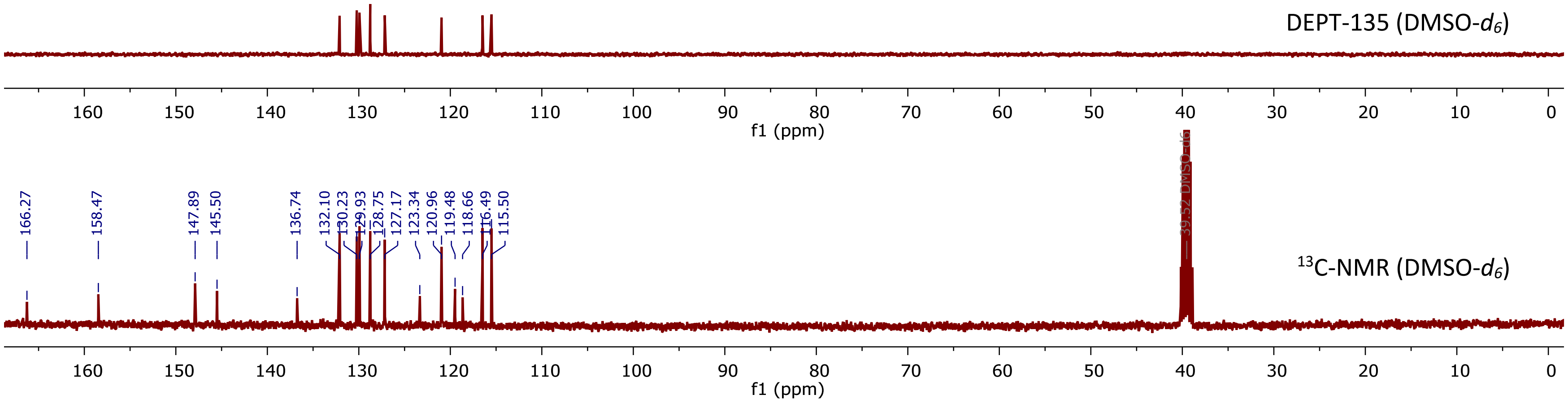

DEPT-135 (DMSO-d6)
f1 (ppm)
166.27
158.47
147.89
145.50
136.74
132.10
130.23
129.93
128.75
127.17
123.34
120.96
119.48
118.66
116.49
115.50
39.52 DMSO-d6
13C-NMR (DMSO-d6)
f1 (ppm)

EI MS (70eV)

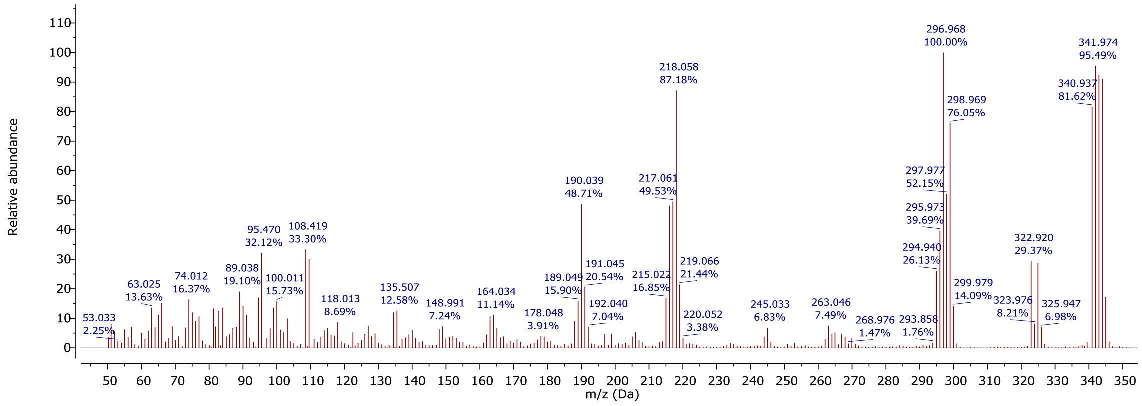

ESI-QTOF (positive ionization)

MS Zoomed Spectrum

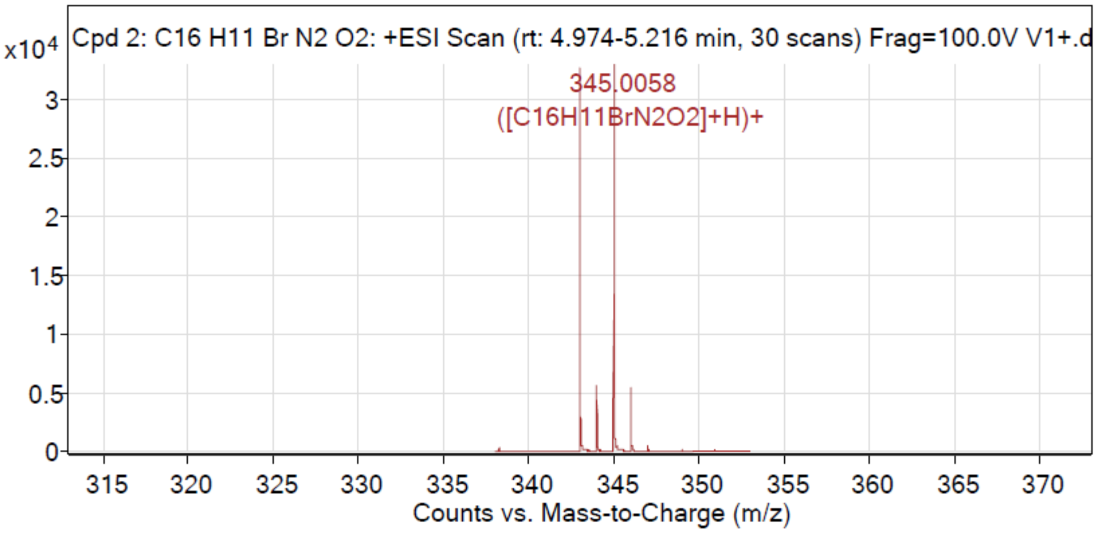

MS Spectrum Peak List

m/z	Calc *m/z*	Diff(ppm)	z	Abund	Formula	Ion
343.0076	343.0077	0.19	1	32719.25	C16H11BrN2O2	(M+H)+
344.0108	344.0108	-0.06	1	5710.41	C16H11BrN2O2	(M+H)+
345.0058	345.0058	-0.02	1	33001.97	C16H11BrN2O2	(M+H)+
346.0088	346.0088	0.11	1	5543.34	C16H11BrN2O2	(M+H)+
347.0118	347.0115	-0.83	1	545.28	C16H11BrN2O2	(M+H)+
348.0167	348.0141	-7.46	1	63.63	C16H11BrN2O2	(M+H)+

ESI-QTOF (negative ionization)

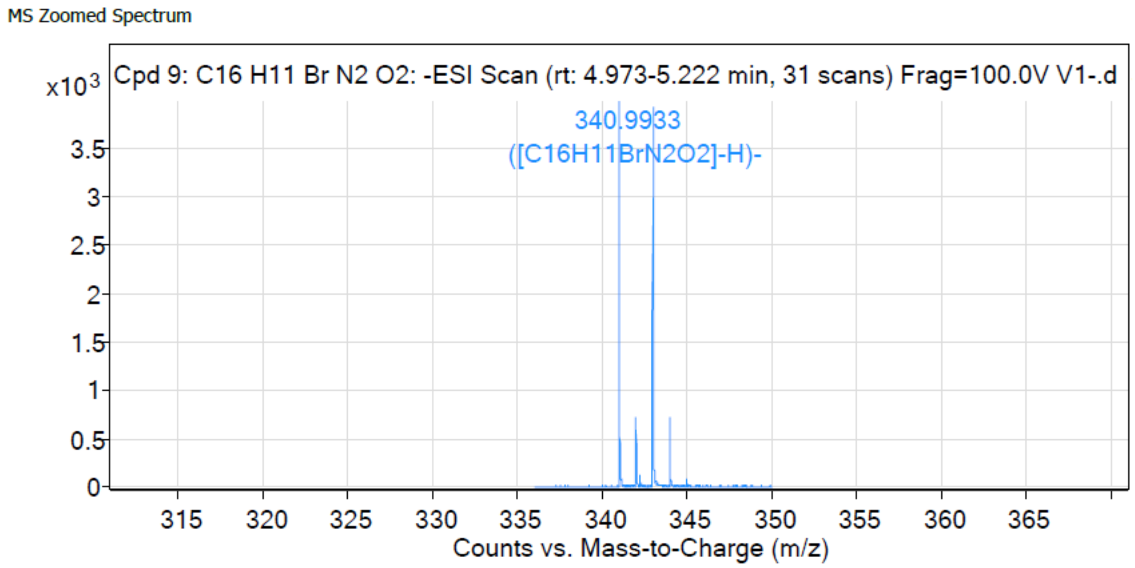

MS Spectrum Peak List

m/z	Calc *m/z*	Diff(ppm)	z	Abund	Formula	Ion
340.9933	340.9931	-0.49	1	4083.53	C16H11BrN2O2	(M-H)-
341.9964	341.9962	-0.38	1	733.42	C16H11BrN2O2	(M-H)-
342.9915	342.9912	-0.85	1	3978.05	C16H11BrN2O2	(M-H)-
343.9944	343.9943	-0.29	1	722.68	C16H11BrN2O2	(M-H)-
344.9979	344.9969	-2.91	1	84.04	C16H11BrN2O2	(M-H)-

IR

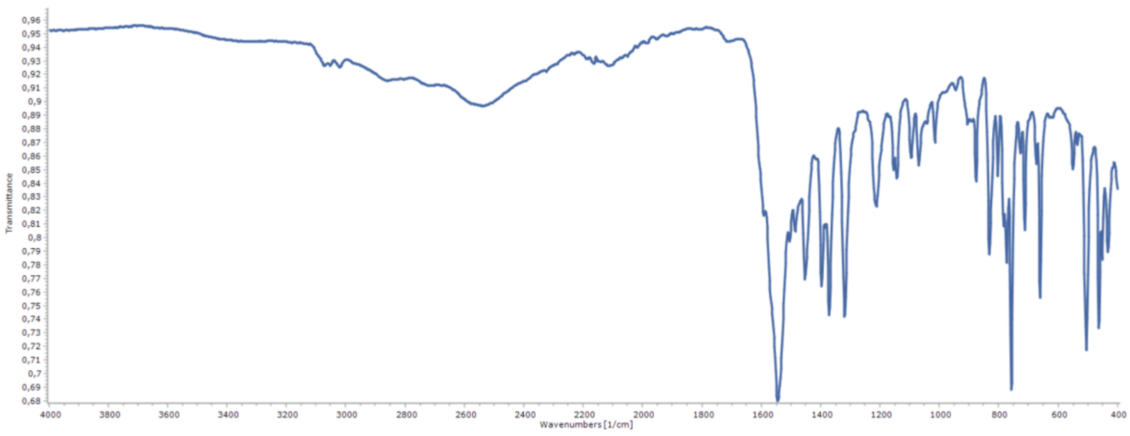

Compound **9**

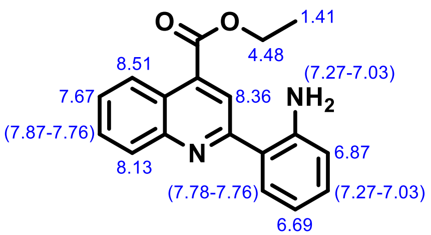

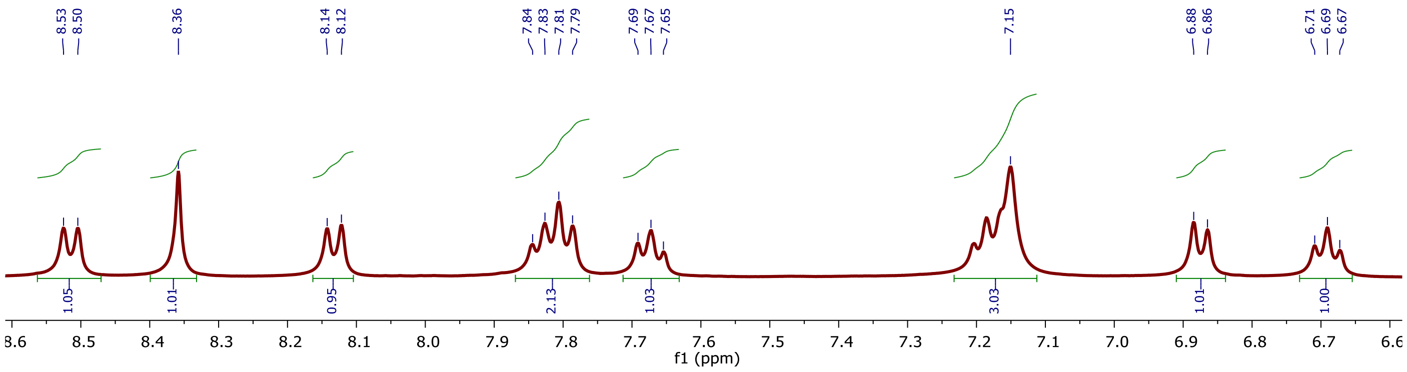

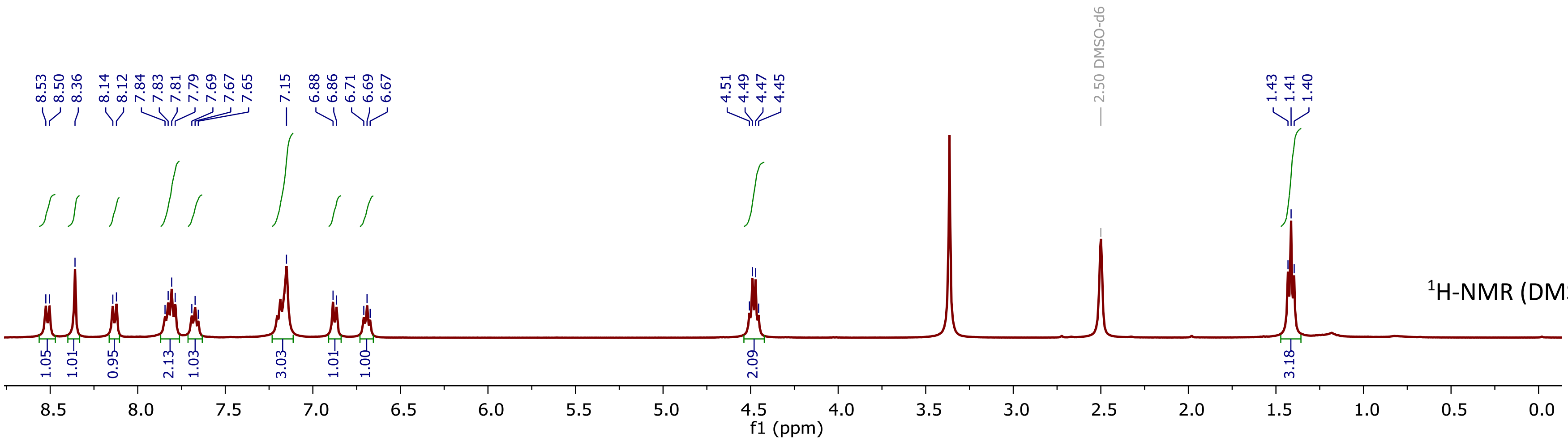

^{1}H-NMR (DMSO-d_6)

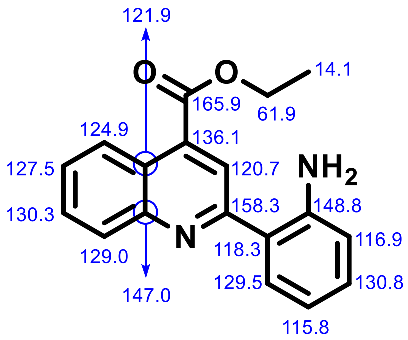

DEPT-135 (DMSO-d_6)

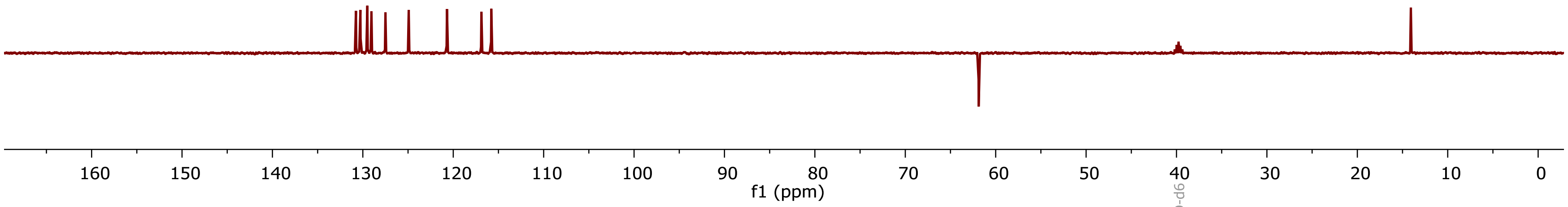

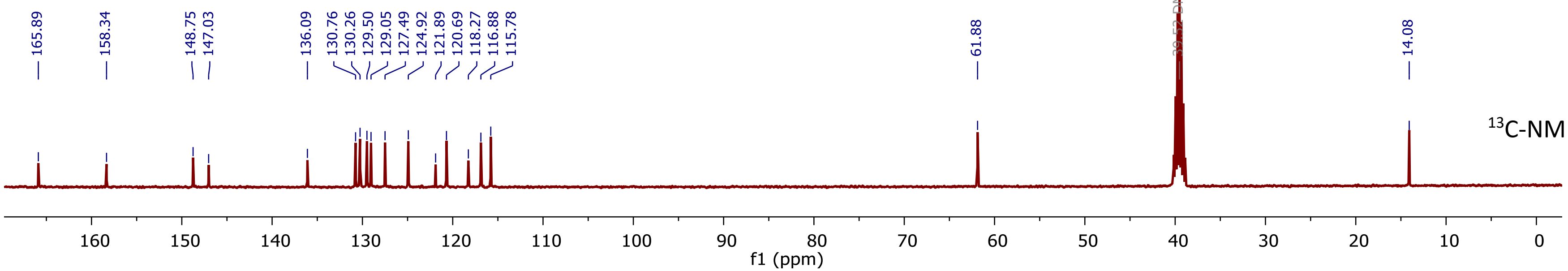

^{13}C-NMR (DMSO-d_6)

EI MS (70eV)

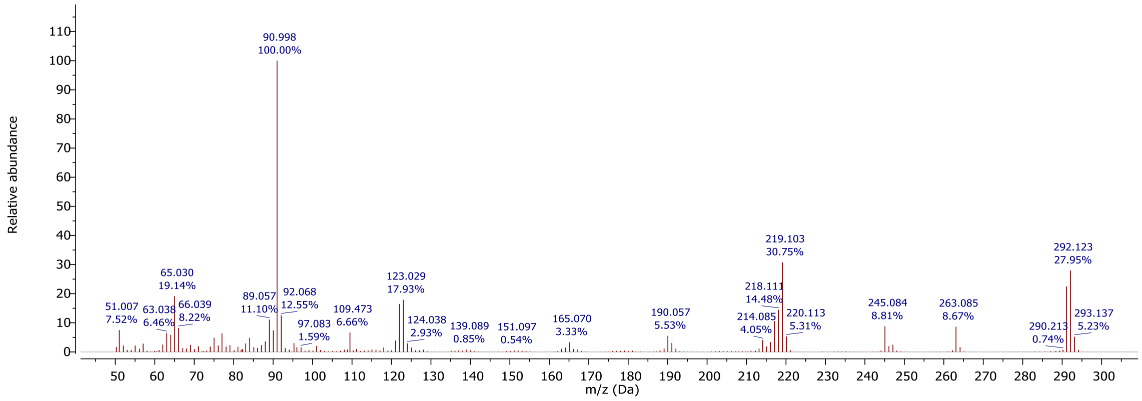

ESI-QTOF (positive ionization)

MS Zoomed Spectrum

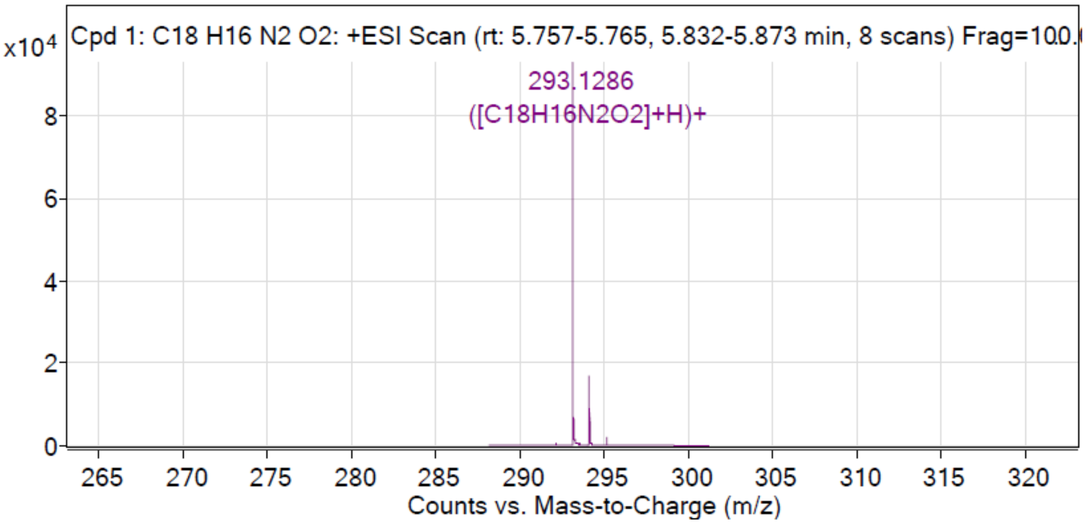

MS Spectrum Peak List

m/z	Calc *m/z*	Diff(ppm)	z	Abund	Formula	Ion
293.1286	293.1285	-0.56	1	93212.11	C18H16N2O2	(M+H)+
294.1319	294.1316	-1.12	1	17093.06	C18H16N2O2	(M+H)+
295.1344	295.1344	0.09	1	2107.36	C18H16N2O2	(M+H)+
296.1386	296.1371	-5.08	1	196.75	C18H16N2O2	(M+H)+

IR

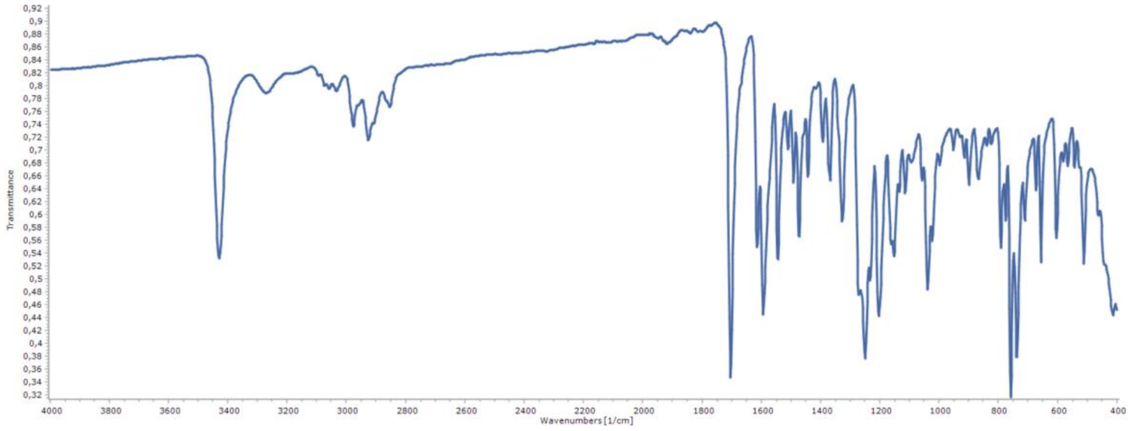

Compound **10**

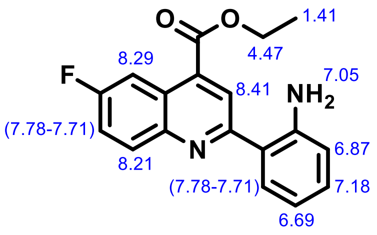

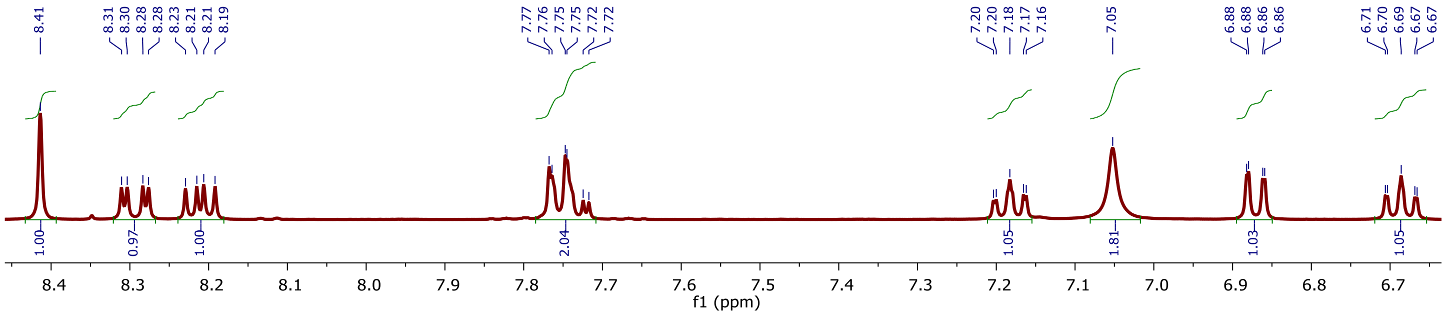

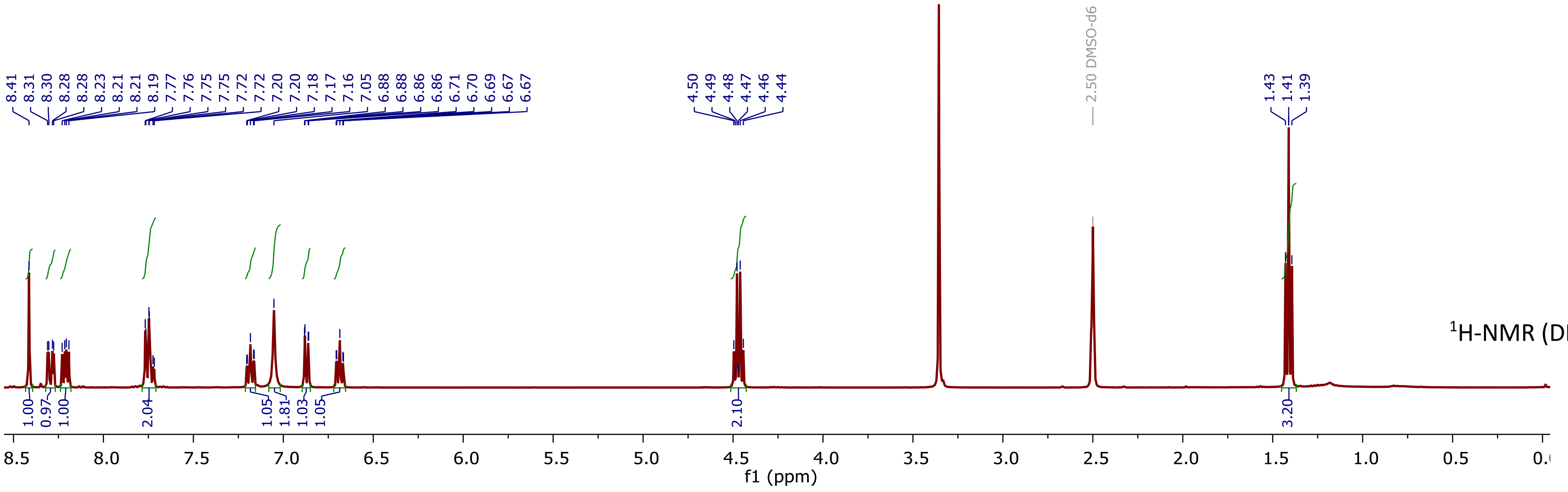

^{1}H-NMR (DMSO-d_6)

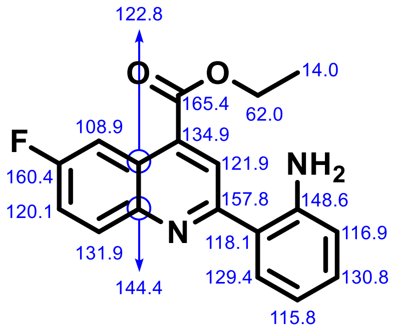

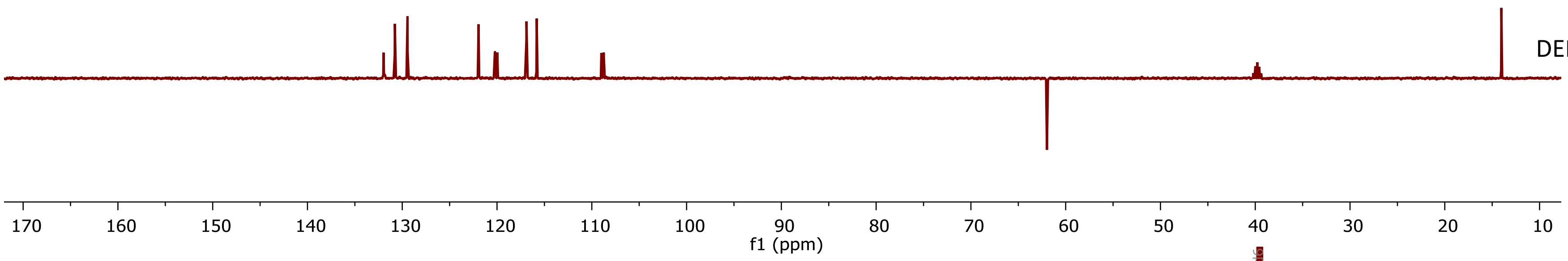

DEPT-135 (DMSO-d_6)

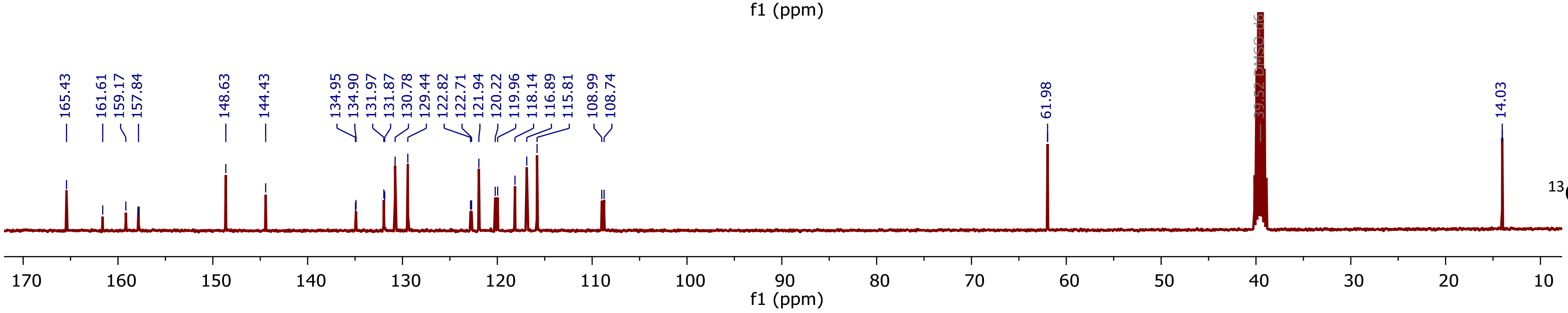

^{13}C-NMR (DMSO-d_6)

^{13}C-^{19}F couplings in ^{13}C-NMR. Only coupled peaks have been represented to make it easier to understand.

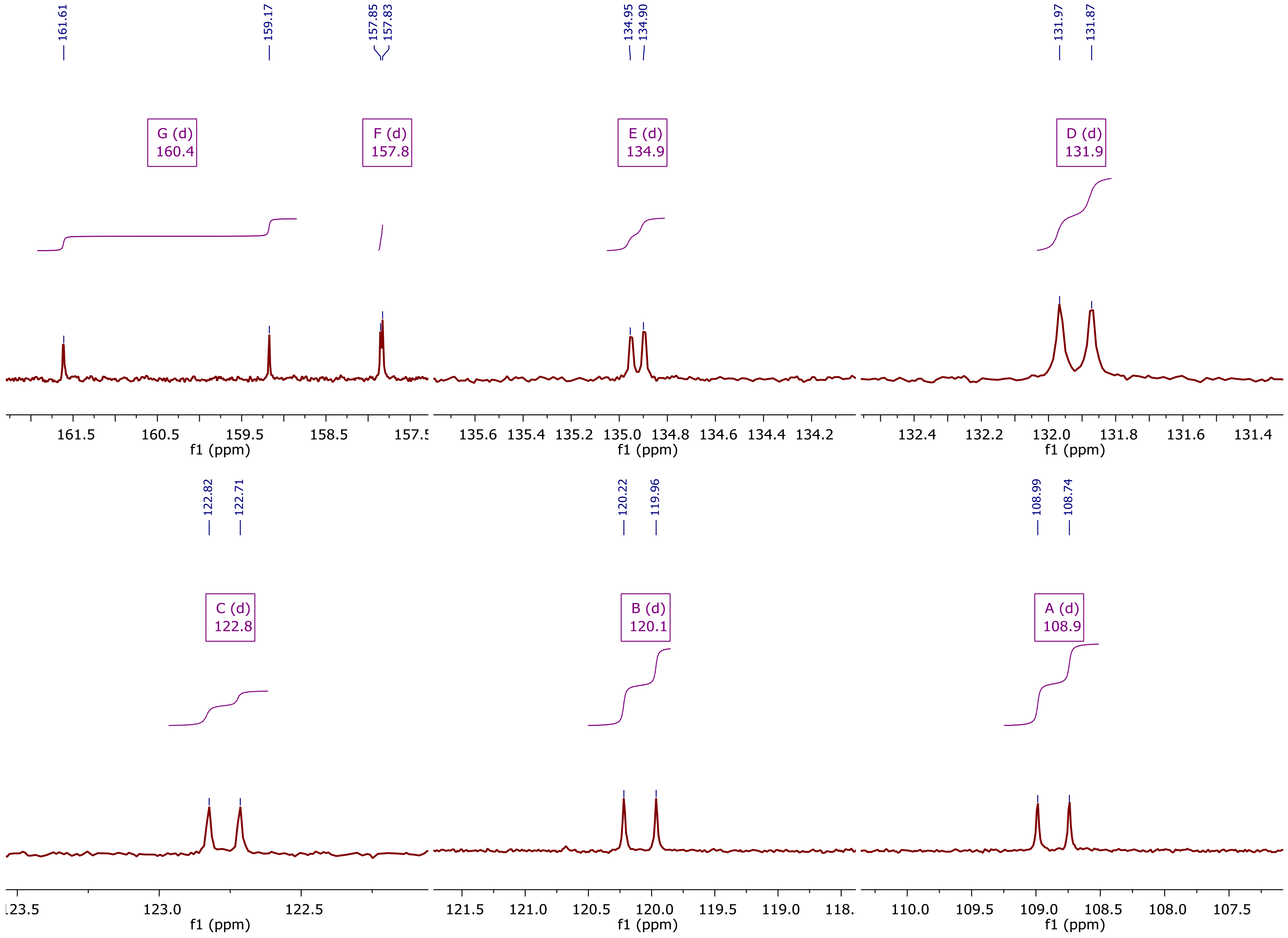

EI MS (70eV)

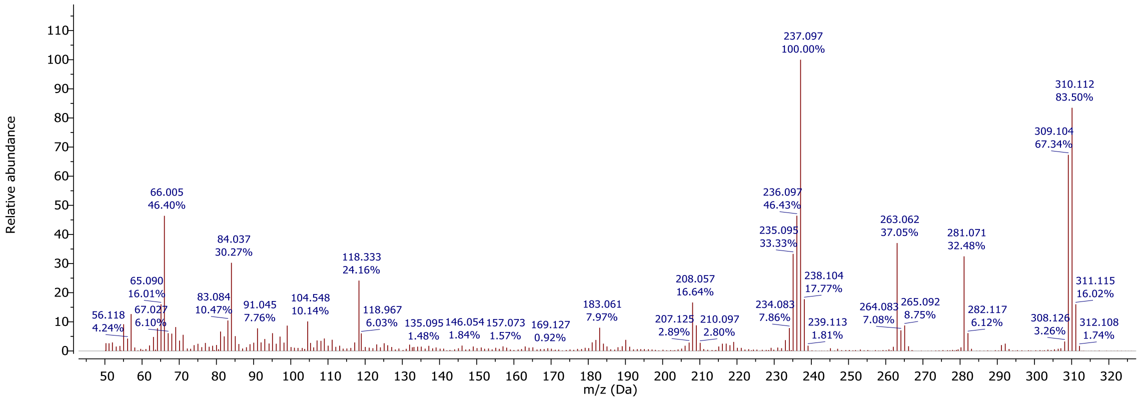

ESI-QTOF (positive ionization)

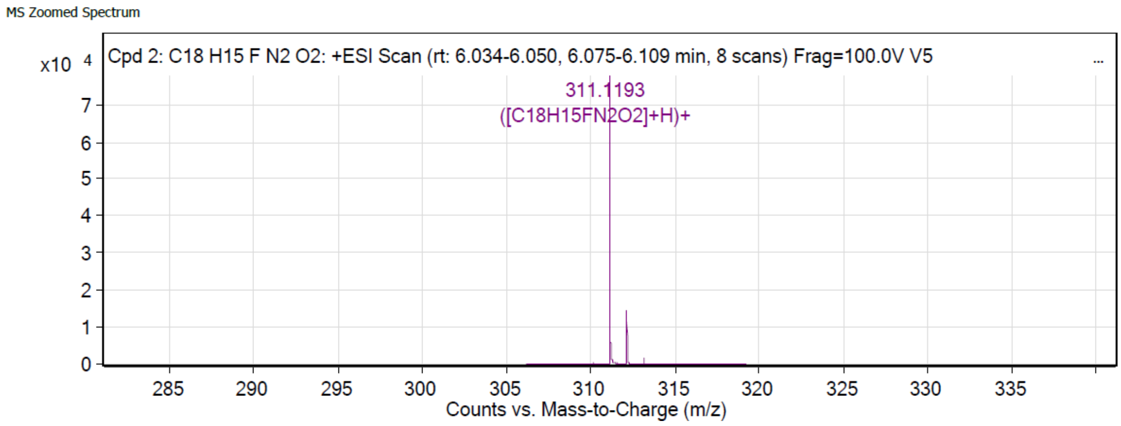

MS Spectrum Peak List

m/z	Calc *m/z*	Diff(ppm)	z	Abund	Formula	Ion
311.1193	311.119	-0.76	1	78483.28	C18H15FN2O2	(M+H)+
312.1226	312.1222	-1.42	1	14394.33	C18H15FN2O2	(M+H)+
313.1259	313.125	-2.92	1	1625.32	C18H15FN2O2	(M+H)+
314.1284	314.1276	-2.51	1	147.62	C18H15FN2O2	(M+H)+

IR

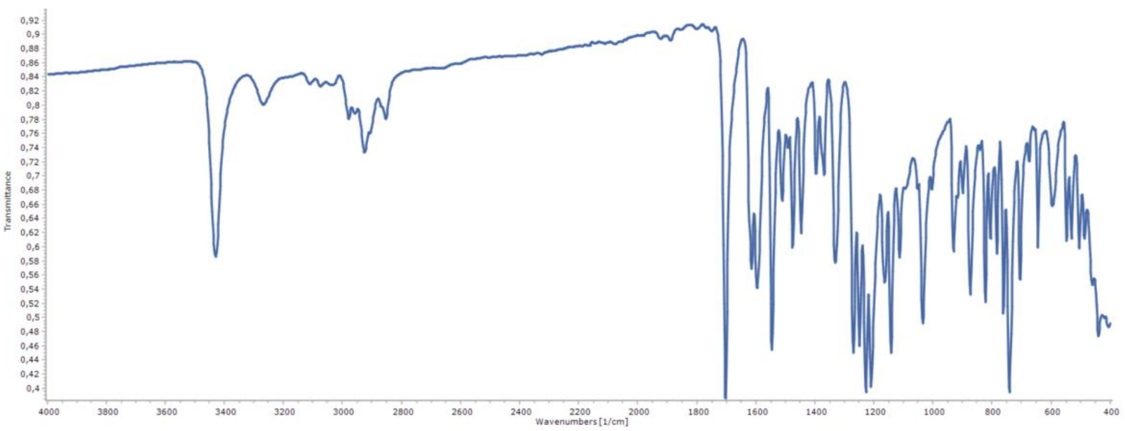

Compound **11**

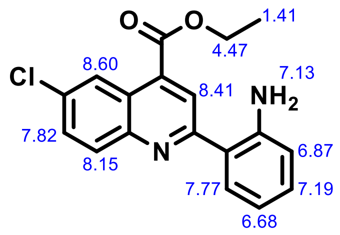

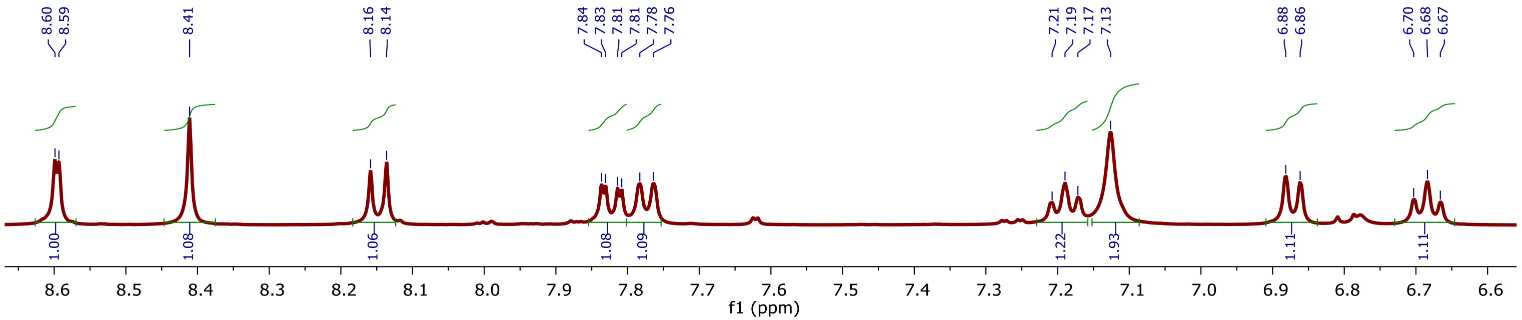

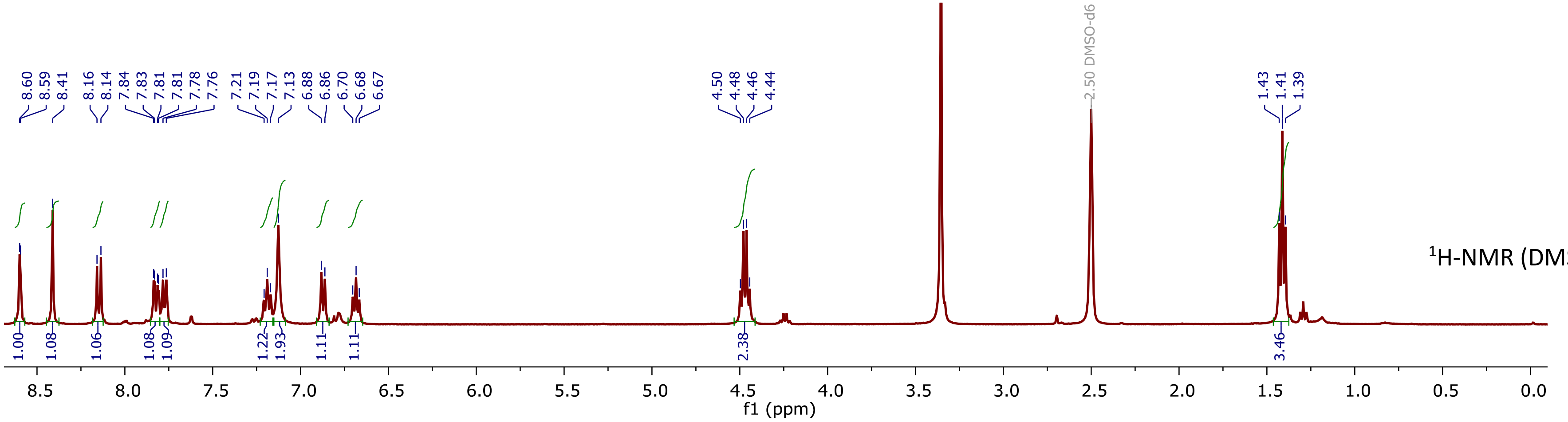

^{1}H-NMR (DMSO-d_6)

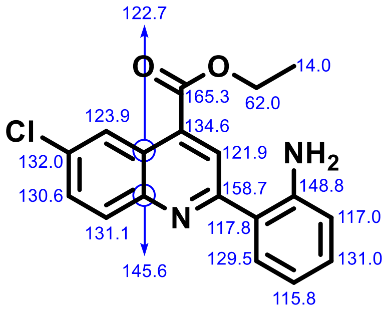

122.7
14.0
165.3
62.0
123.9
134.6
Cl
121.9
NH2
132.0
158.7
148.8
130.6
117.8
117.0
131.1
N
129.5
131.0
145.6
115.8

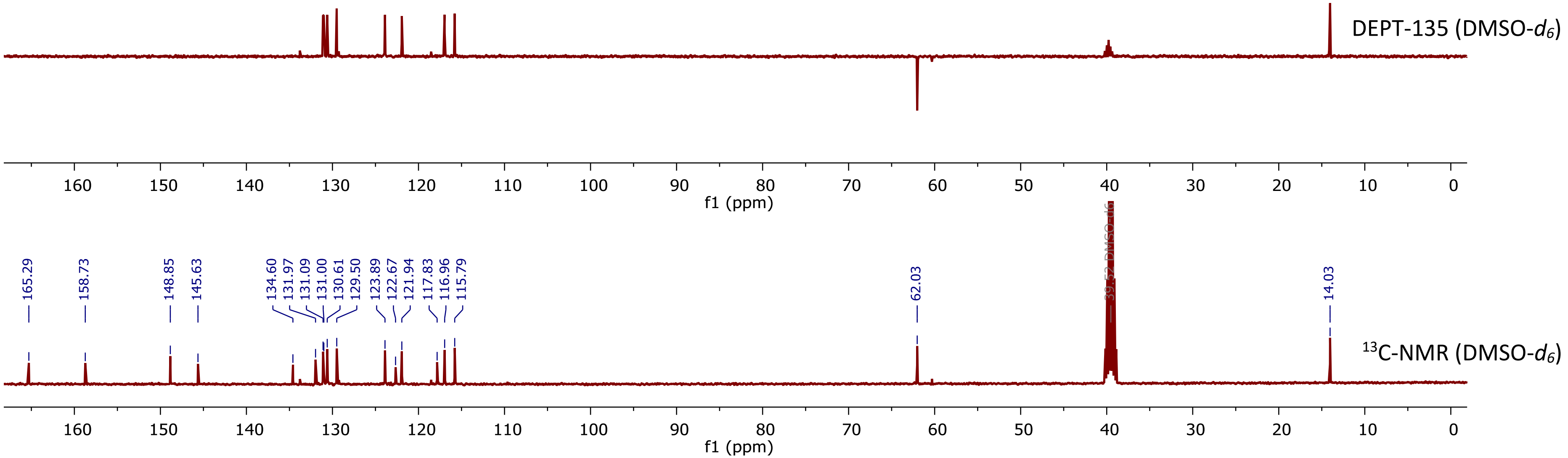

DEPT-135 (DMSO-d6)
f1 (ppm)
165.29
158.73
148.85
145.63
134.60
131.97
131.09
131.00
130.61
129.50
123.89
122.67
121.94
117.83
116.96
115.79
62.03
14.03
13C-NMR (DMSO-d6)
f1 (ppm)

EI MS (70eV)

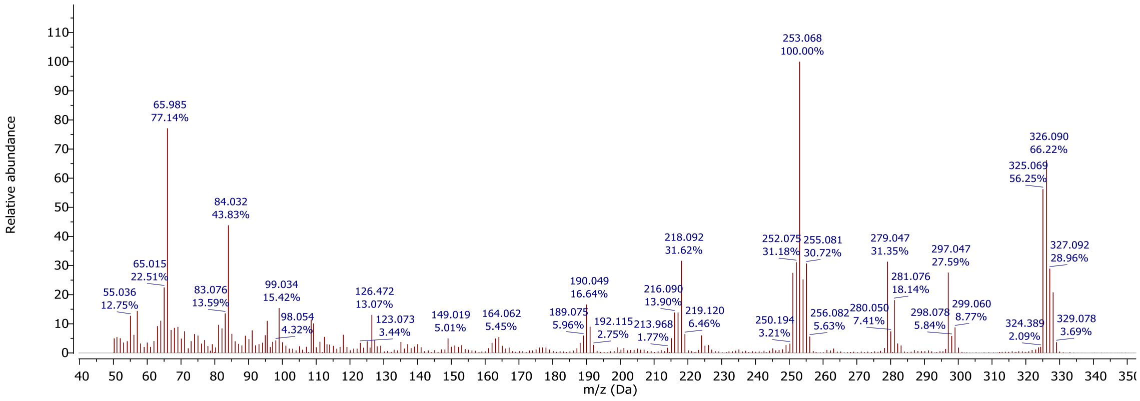

ESI-QTOF (positive ionization)

MS Zoomed Spectrum

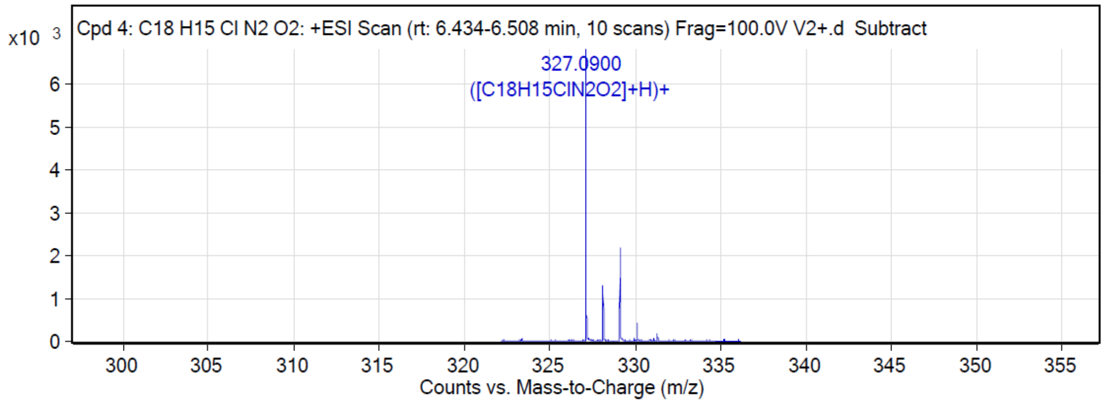

MS Spectrum Peak List

m/z	Calc *m/z*	Diff(ppm)	z	Abund	Formula	Ion
327.09	327.0895	-1.5	1	6856.84	C18H15ClN2O2	(M+H)+
328.0932	328.0926	-1.61	1	1321.54	C18H15ClN2O2	(M+H)+
329.0875	329.0872	-1.2	1	2242.85	C18H15ClN2O2	(M+H)+
330.0897	330.0899	0.77	1	429.65	C18H15ClN2O2	(M+H)+
331.0925	331.0926	0.39	1	72.54	C18H15ClN2O2	(M+H)+

IR

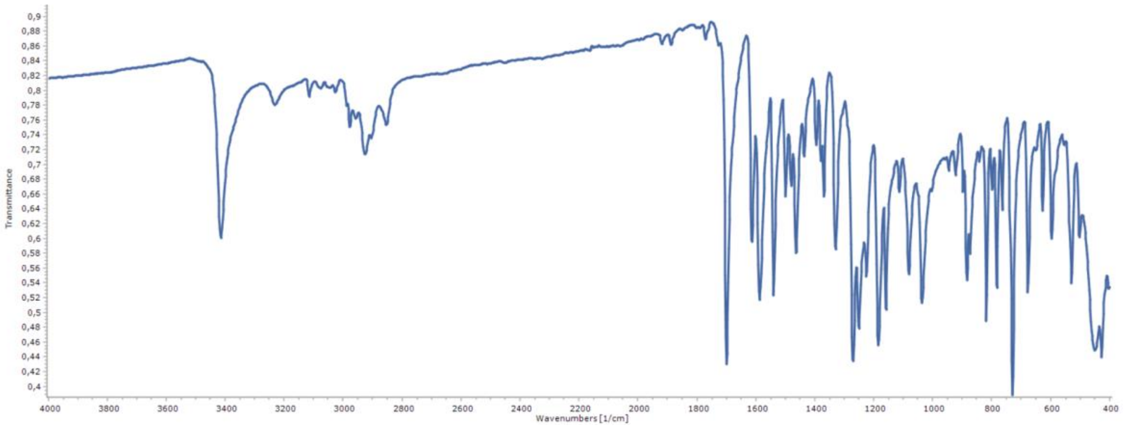

Compound **12**

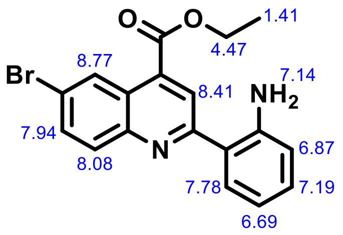

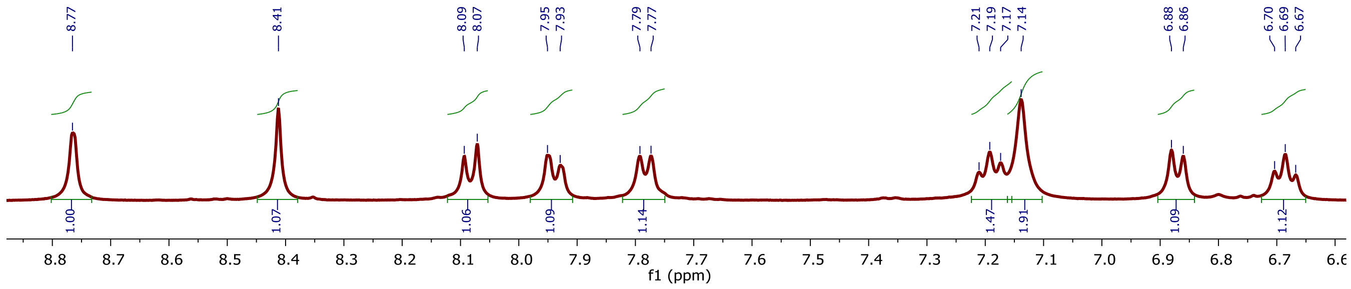

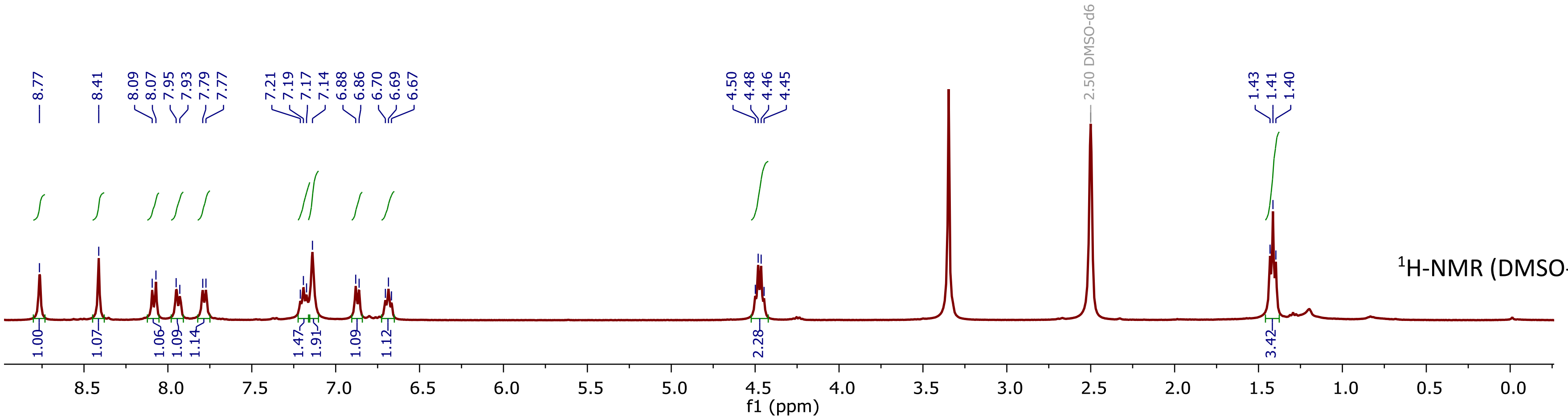

^{1}H-NMR (DMSO-d_6)

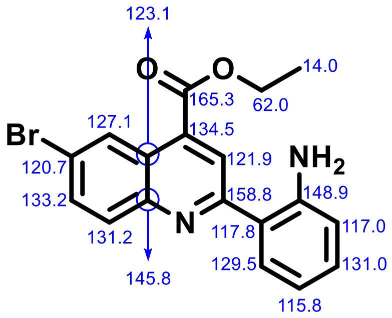
123.1
O
O
14.0
165.3
62.0
Br
127.1
134.5
121.9
NH2
120.7
158.8
148.9
133.2
N
117.8
117.0
131.2
129.5
131.0
145.8
115.8

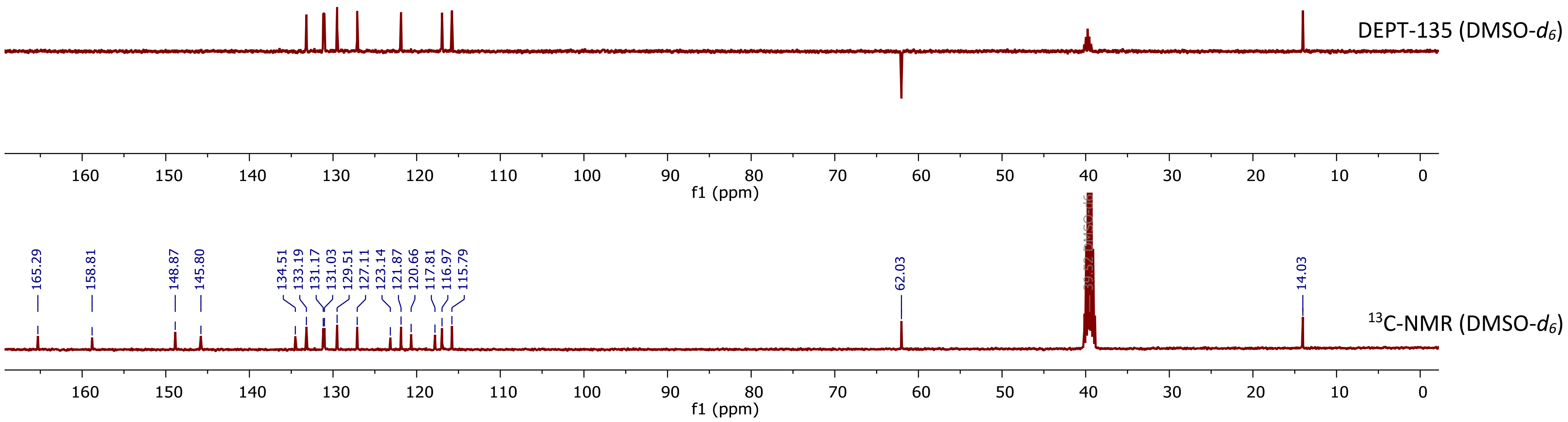
DEPT-135 (DMSO-d6)
160 150 140 130 120 110 100 90 80 70 60 50 40 30 20 10 0
f1 (ppm)
165.29
158.81
148.87
145.80
134.51
133.19
131.17
131.03
129.51
127.11
123.14
121.87
120.66
117.81
116.97
115.79
62.03
39.52 DMSO-d6
14.03
13C-NMR (DMSO-d6)
160 150 140 130 120 110 100 90 80 70 60 50 40 30 20 10 0
f1 (ppm)

EI MS (70eV)

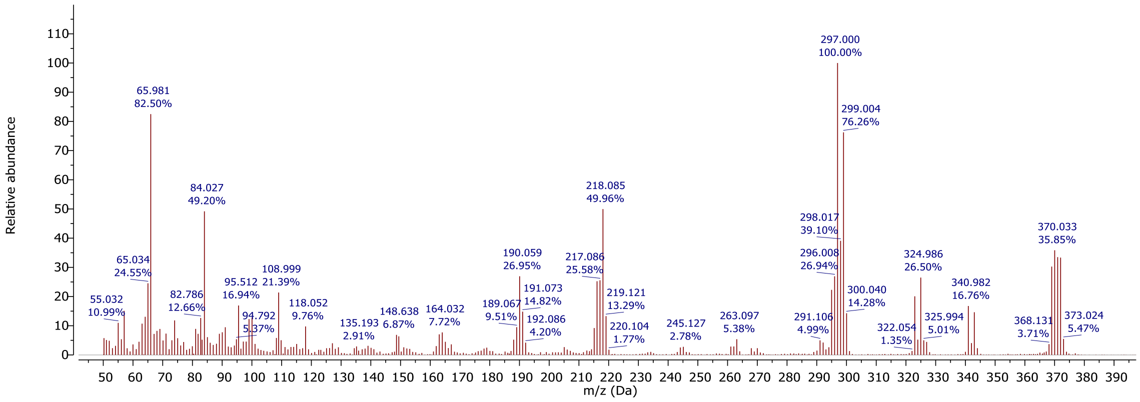

ESI-QTOF (positive ionization)

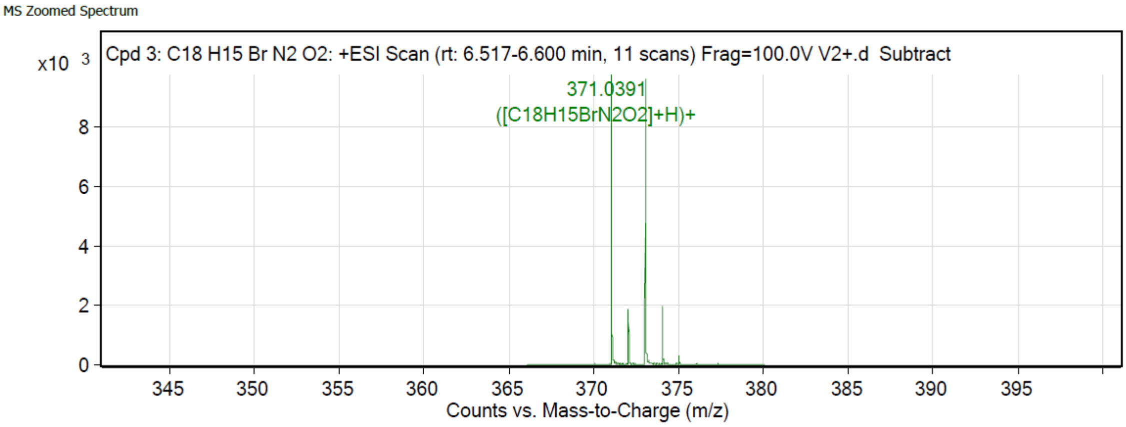

MS Spectrum Peak List

m/z	Calc *m/z*	Diff(ppm)	z	Abund	Formula	Ion
371.0391	371.039	-0.44	1	9931.9	C18H15BrN2O2	(M+H)+
372.0419	372.0421	0.5	1	1882.26	C18H15BrN2O2	(M+H)+
373.0372	373.0371	-0.19	1	9657.04	C18H15BrN2O2	(M+H)+
374.0401	374.0402	0.14	1	1987.29	C18H15BrN2O2	(M+H)+
375.0419	375.0429	2.63	1	289.29	C18H15BrN2O2	(M+H)+

IR

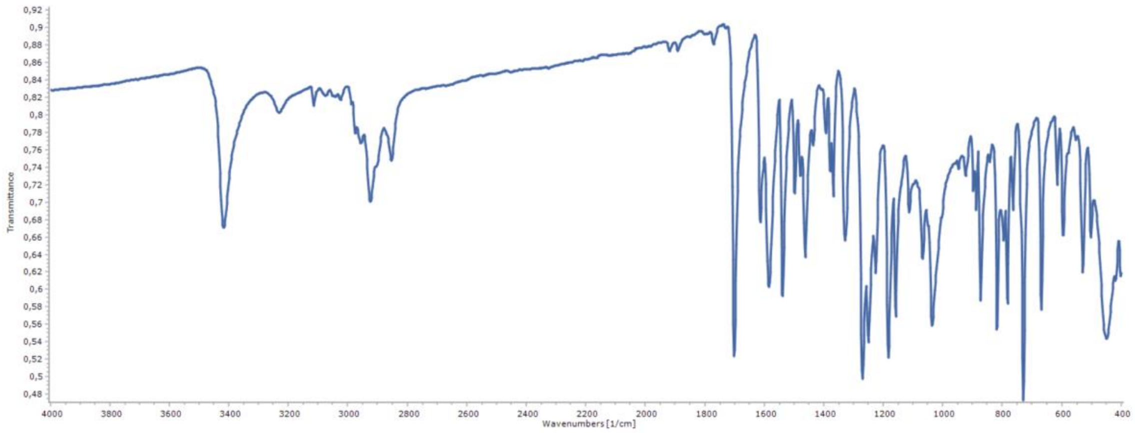

Compound **13a**

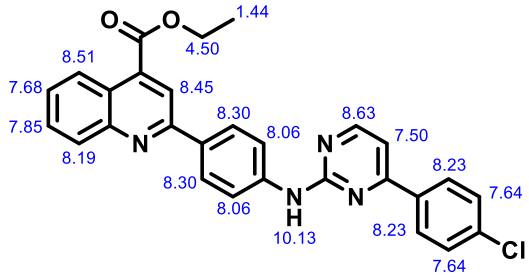

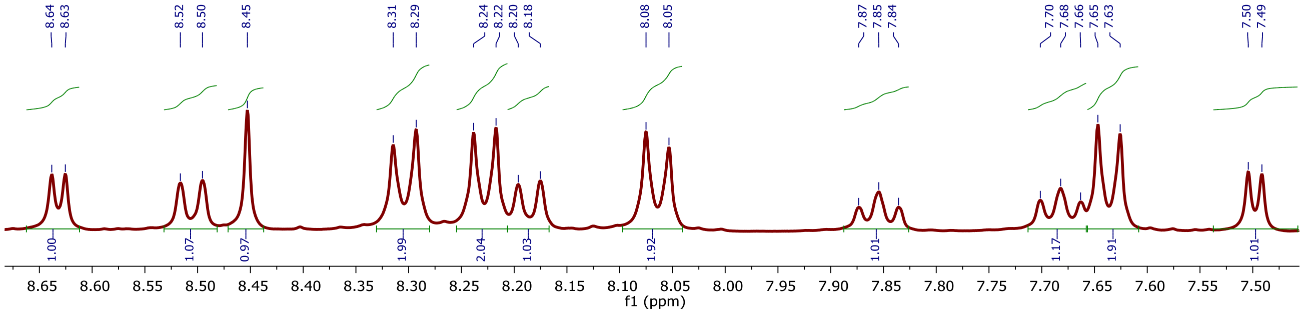

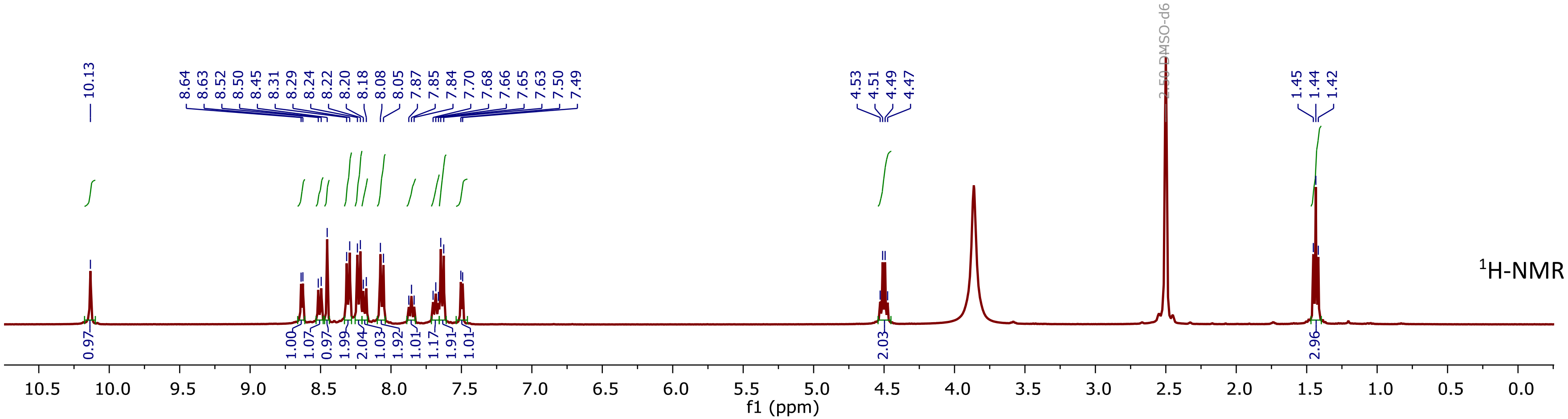

^{1}H-NMR (DMSO-d_6)

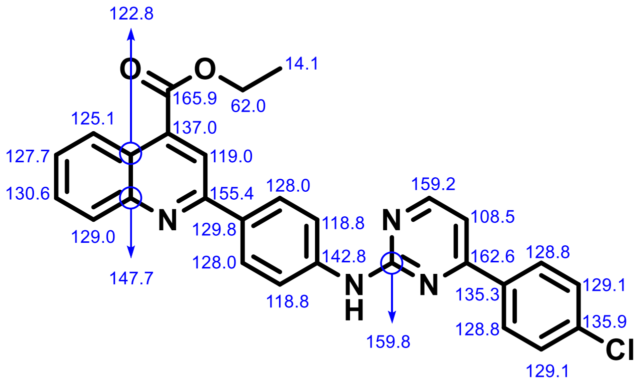
122.8
14.1
165.9
62.0
125.1
137.0
127.7
119.0
130.6
155.4
128.0
159.2
129.8
118.8
108.5
129.0
142.8
162.6
128.8
128.0
147.7
129.1
118.8
135.3
135.9
159.8
128.8
Cl
129.1

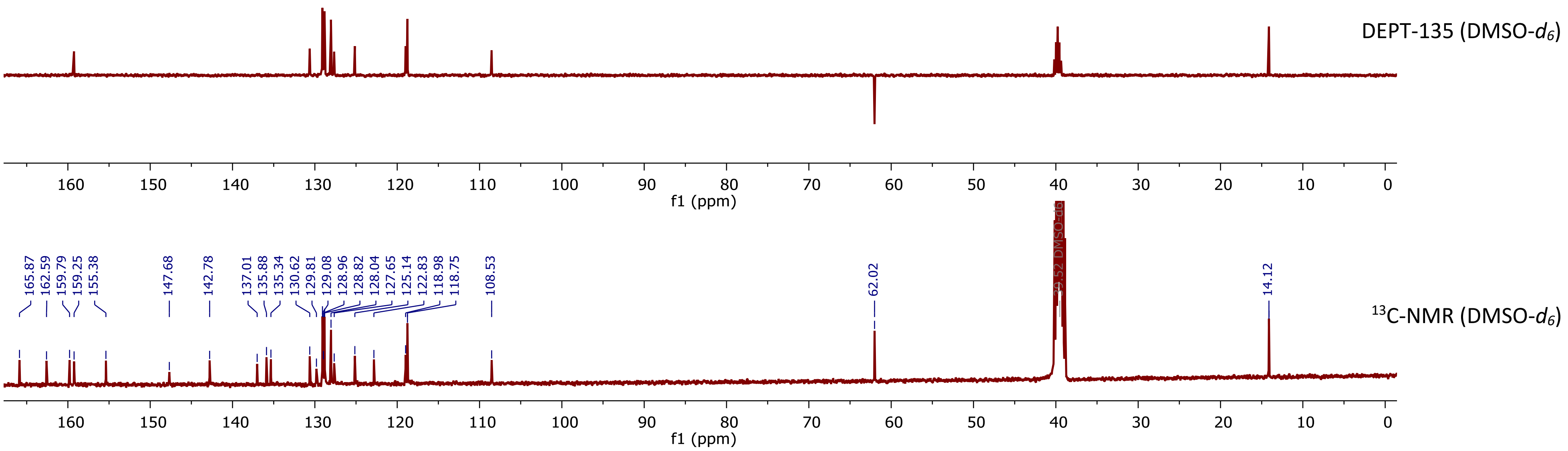
DEPT-135 (DMSO-d_6)
f1 (ppm)
165.87
162.59
159.79
159.25
155.38
147.68
142.78
137.01
135.88
135.34
130.62
129.81
129.08
128.96
128.82
128.04
127.65
125.14
122.83
118.98
118.75
108.53
62.02
14.12
^{13}C-NMR (DMSO-d_6)
f1 (ppm)

EI MS (70eV)

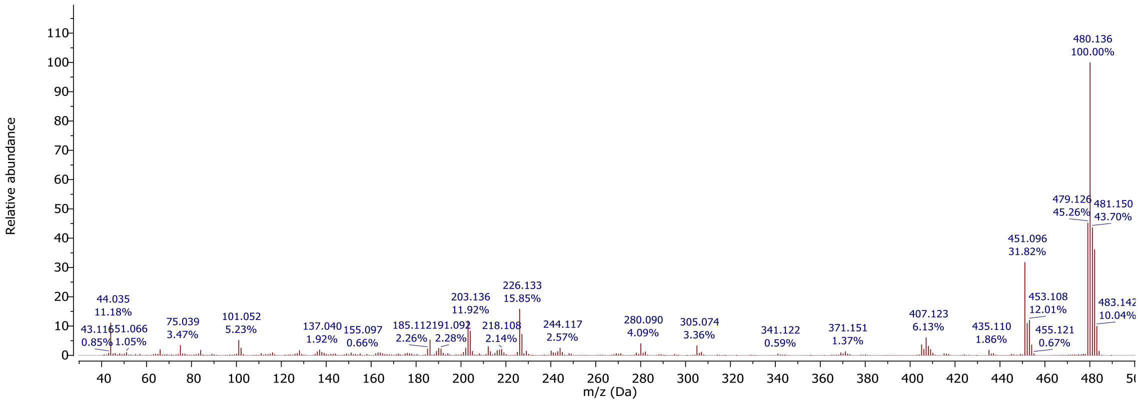

ESI-QTOF (positive ionization)

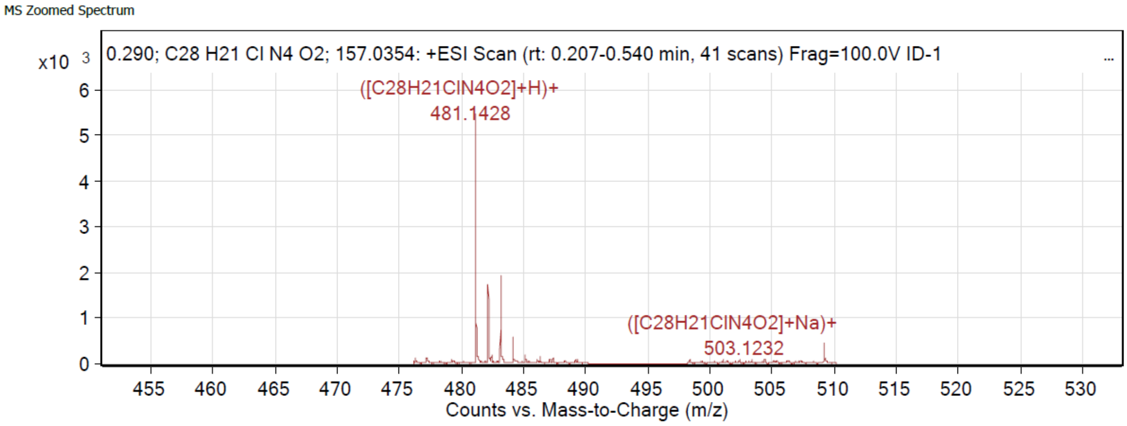

MS Spectrum Peak List

m/z	Calc *m/z*	Diff(ppm)	z	Abund	Formula	Ion
481.1428	481.1426	-0.45	1	5473.86	C28H21ClN4O2	(M+H)+
482.1467	482.1457	-2.2	1	1771.85	C28H21ClN4O2	(M+H)+
483.1418	483.1409	-1.88	1	1951.64	C28H21ClN4O2	(M+H)+
484.1443	484.1432	-2.15	1	578.14	C28H21ClN4O2	(M+H)+
485.1449	485.1459	2.2	1	147.64	C28H21ClN4O2	(M+H)+
503.1232	503.1245	2.7	1	73.82	C28H21ClN4O2	(M+Na)+
504.1162	504.1276	22.55	1	43.03	C28H21ClN4O2	(M+Na)+
505.1281	505.1229	-10.27	1	58.35	C28H21ClN4O2	(M+Na)+

IR

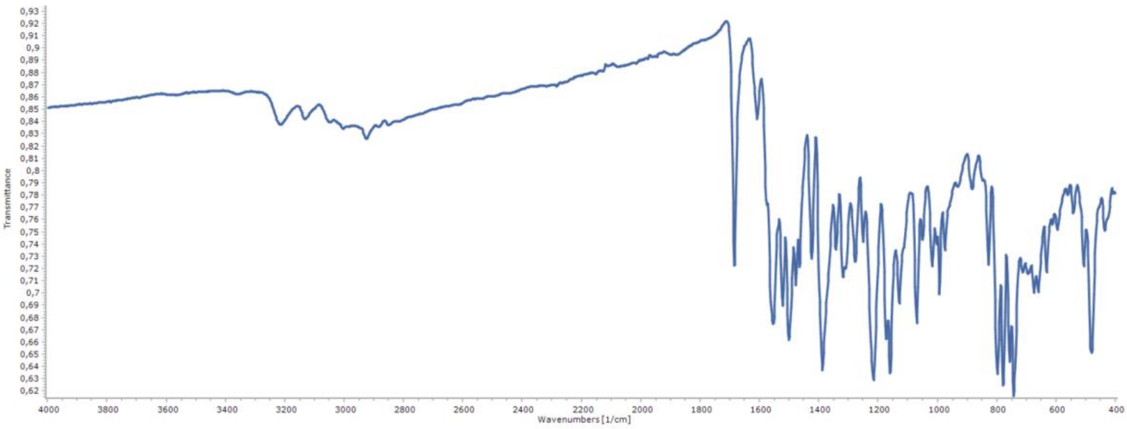

Compound **13b**

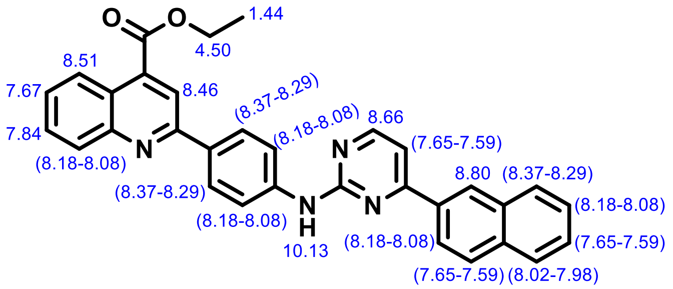

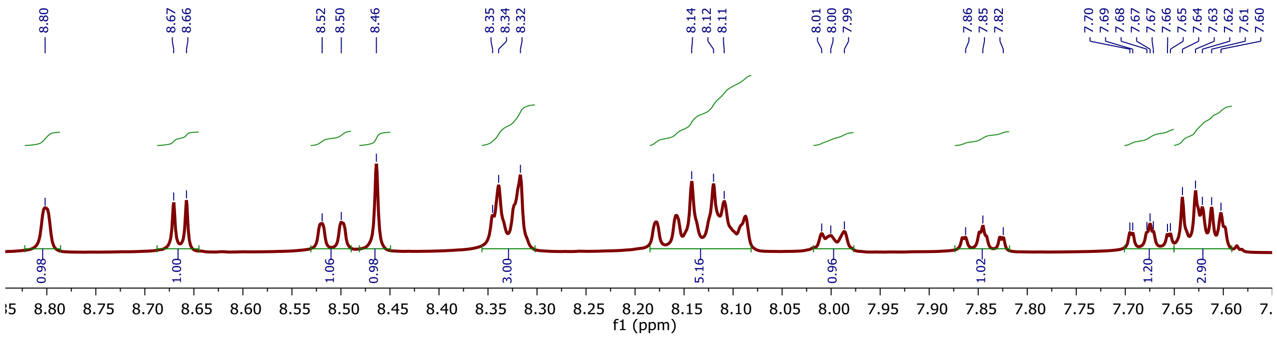

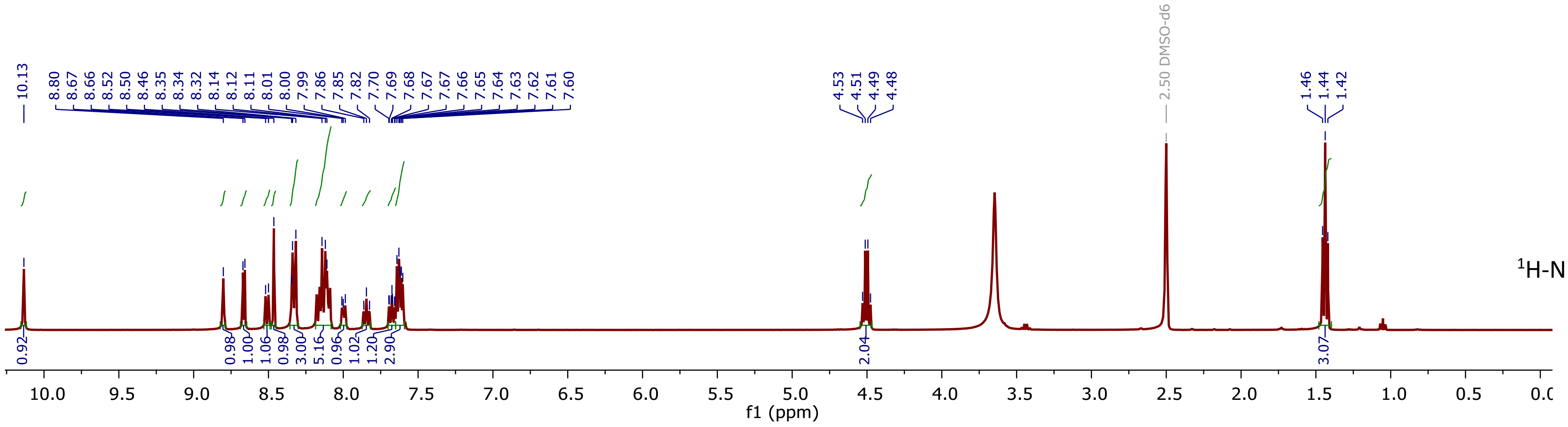

^{1}H-NMR (DMSO-d_6)

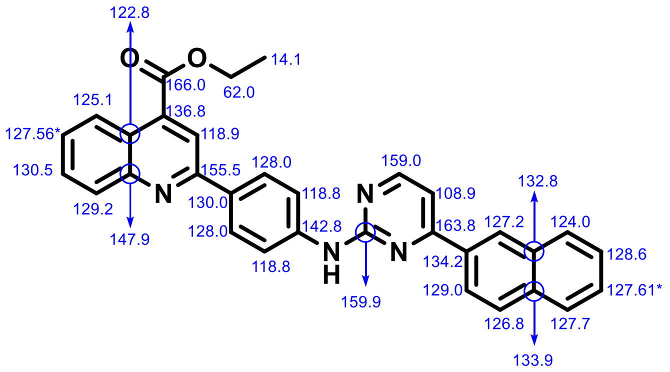

122.8
14.1
166.0
62.0
125.1
136.8
127.56*
118.9
130.5
155.5
128.0
159.0
118.8
108.9
132.8
129.2
130.0
142.8
163.8
127.2
124.0
128.0
128.6
147.9
118.8
134.2
129.0
127.61*
159.9
126.8
127.7
133.9

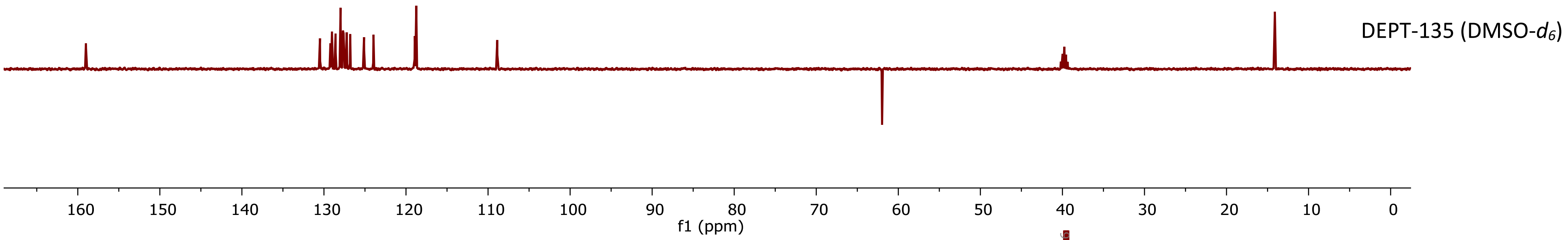

DEPT-135 (DMSO-d_6)
f1 (ppm)

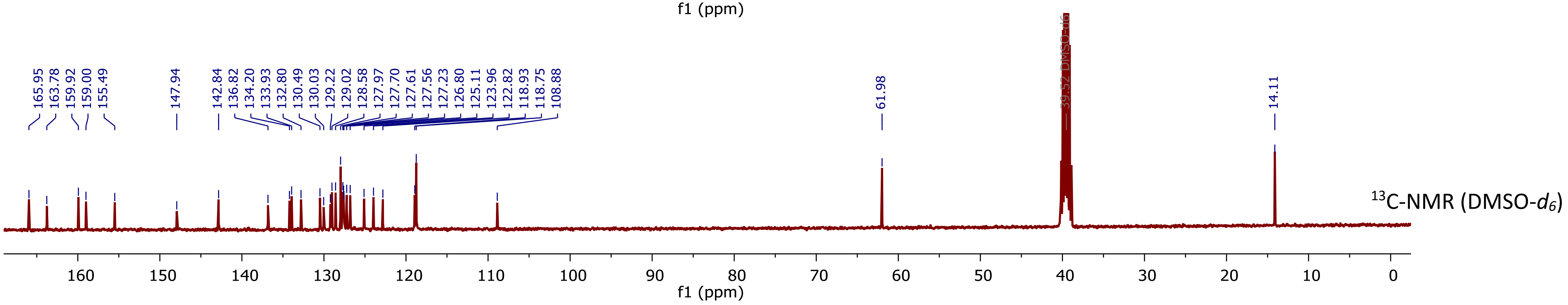

165.95
163.78
159.92
159.00
155.49
147.94
142.84
136.82
134.20
133.93
132.80
130.49
130.03
129.22
129.02
128.58
127.97
127.70
127.61
127.56
127.23
126.80
125.11
123.96
122.82
118.93
118.75
108.88
61.98
39.52 DMSO-d6
14.11
^{13}C-NMR (DMSO-d_6)
f1 (ppm)

EI MS (70eV)

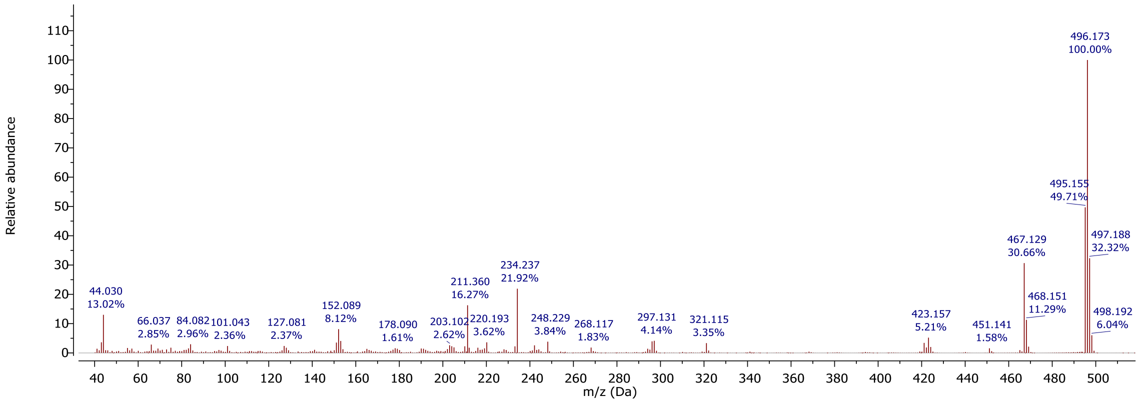

ESI-QTOF (positive ionization)

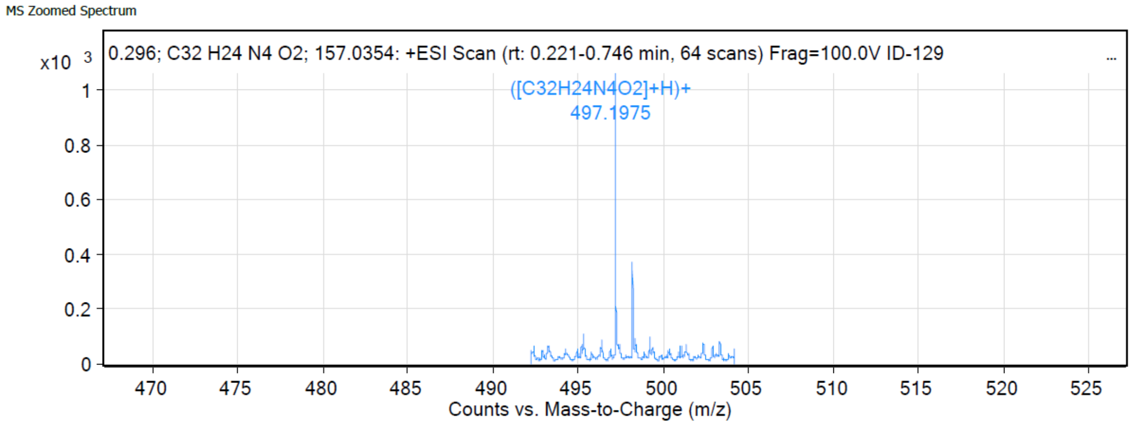

MS Spectrum Peak List

m/z	Calc *m/z*	Diff(ppm)	z	Abund	Formula	Ion
497.1975	497.1972	-0.5	1	1060.32	C32H24N4O2	(M+H)+
498.2009	498.2003	-1.09	1	374.05	C32H24N4O2	(M+H)+
499.2054	499.2033	-4.16	1	96.6	C32H24N4O2	(M+H)+

IR

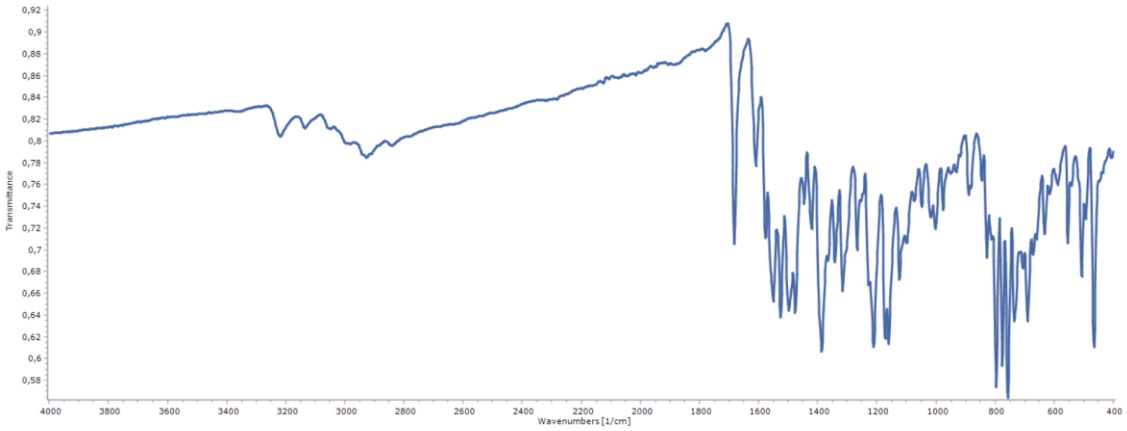

Compound **14a**

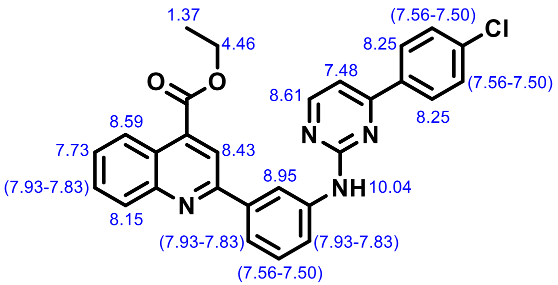

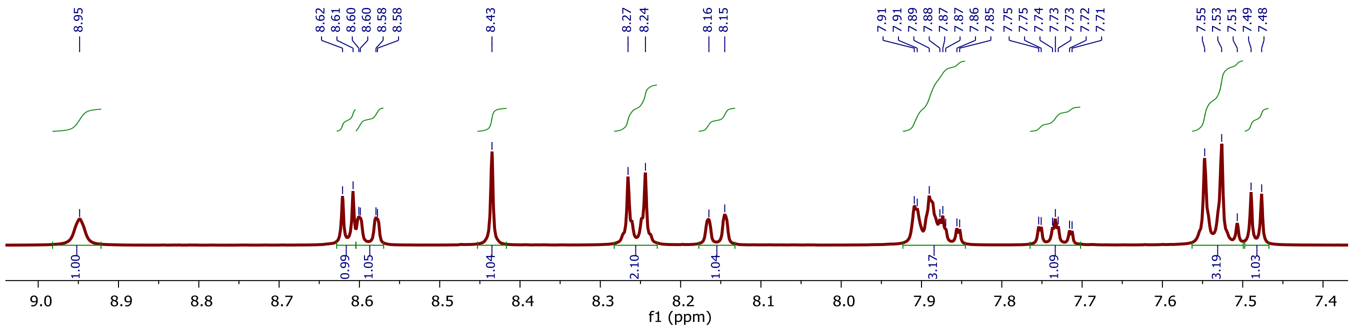

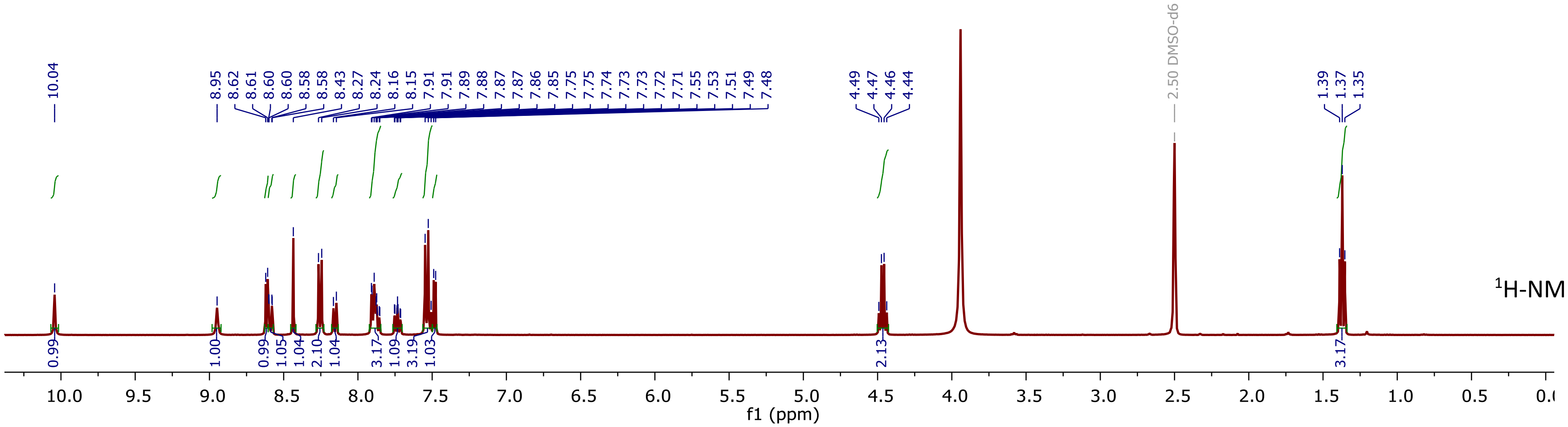

^{1}H-NMR (DMSO-d_6)

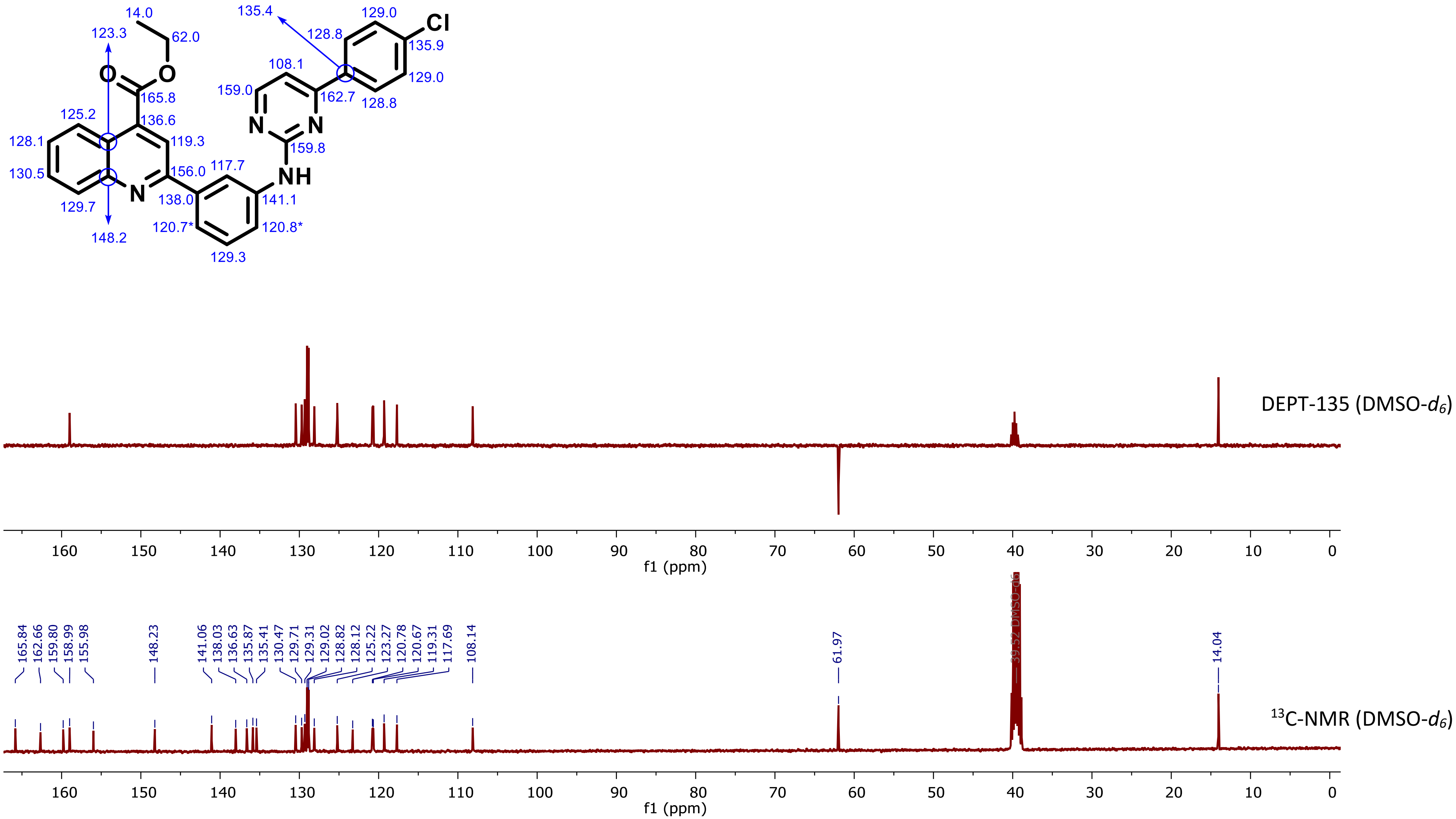
14.0
62.0
123.3
135.4
129.0
Cl
128.8
135.9
108.1
O
O
165.8
159.0
129.0
162.7
128.8
125.2
136.6
N
N
128.1
119.3
159.8
156.0
117.7
NH
130.5
129.7
N
138.0
141.1
120.7*
120.8*
148.2
129.3
DEPT-135 (DMSO-d6)
f1 (ppm)
165.84
162.66
159.80
158.99
155.98
148.23
141.06
138.03
136.63
135.87
135.41
130.47
129.71
129.31
129.02
128.82
128.12
125.22
123.27
120.78
120.67
119.31
117.69
108.14
61.97
39.52 DMSO-d6
14.04
13C-NMR (DMSO-d6)
f1 (ppm)

EI MS (70eV)

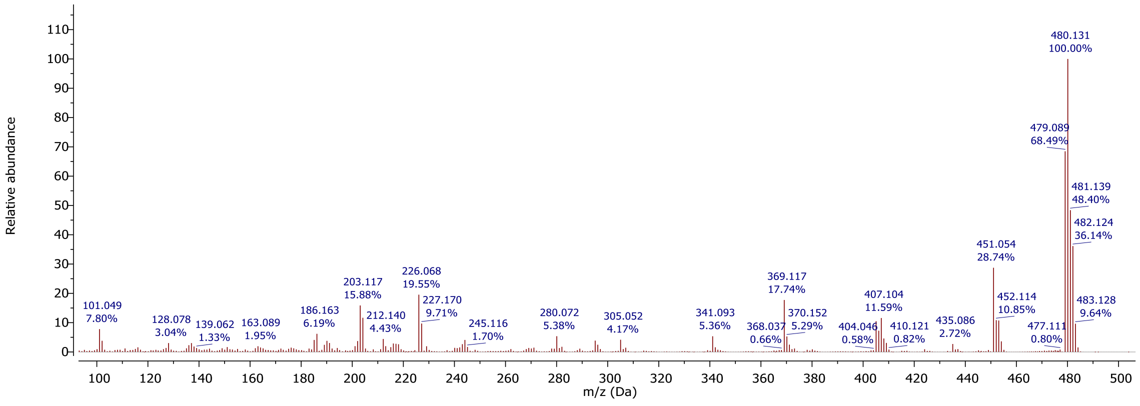

ESI-QTOF (positive ionization)

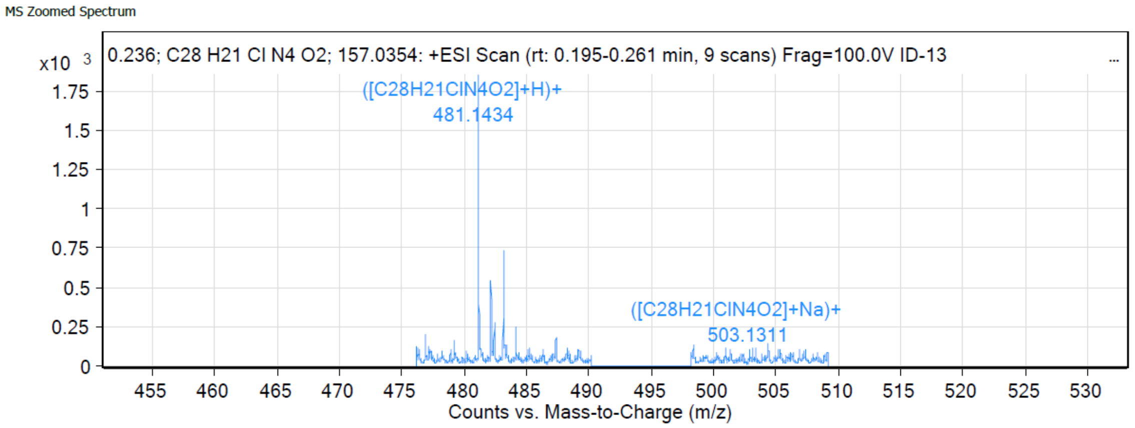

MS Spectrum Peak List

m/z	Calc *m/z*	Diff(ppm)	z	Abund	Formula	Ion
481.1434	481.1426	-1.65	1	1858.02	C28H21ClN4O2	(M+H)+
482.1468	482.1457	-2.28	1	550.61	C28H21ClN4O2	(M+H)+
483.1417	483.1409	-1.51	1	743.43	C28H21ClN4O2	(M+H)+
484.1446	484.1432	-2.87	1	248.57	C28H21ClN4O2	(M+H)+
485.1464	485.1459	-0.93	1	79.29	C28H21ClN4O2	(M+H)+
503.1311	503.1245	-13.12	1	117.9	C28H21ClN4O2	(M+Na)+
504.1385	504.1276	-21.55	1	65.09	C28H21ClN4O2	(M+Na)+

IR

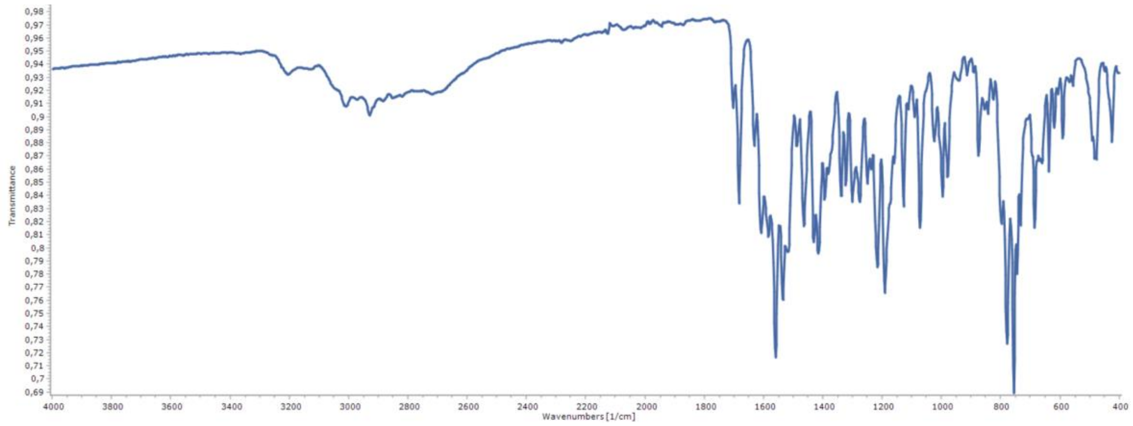

Compound **14b**

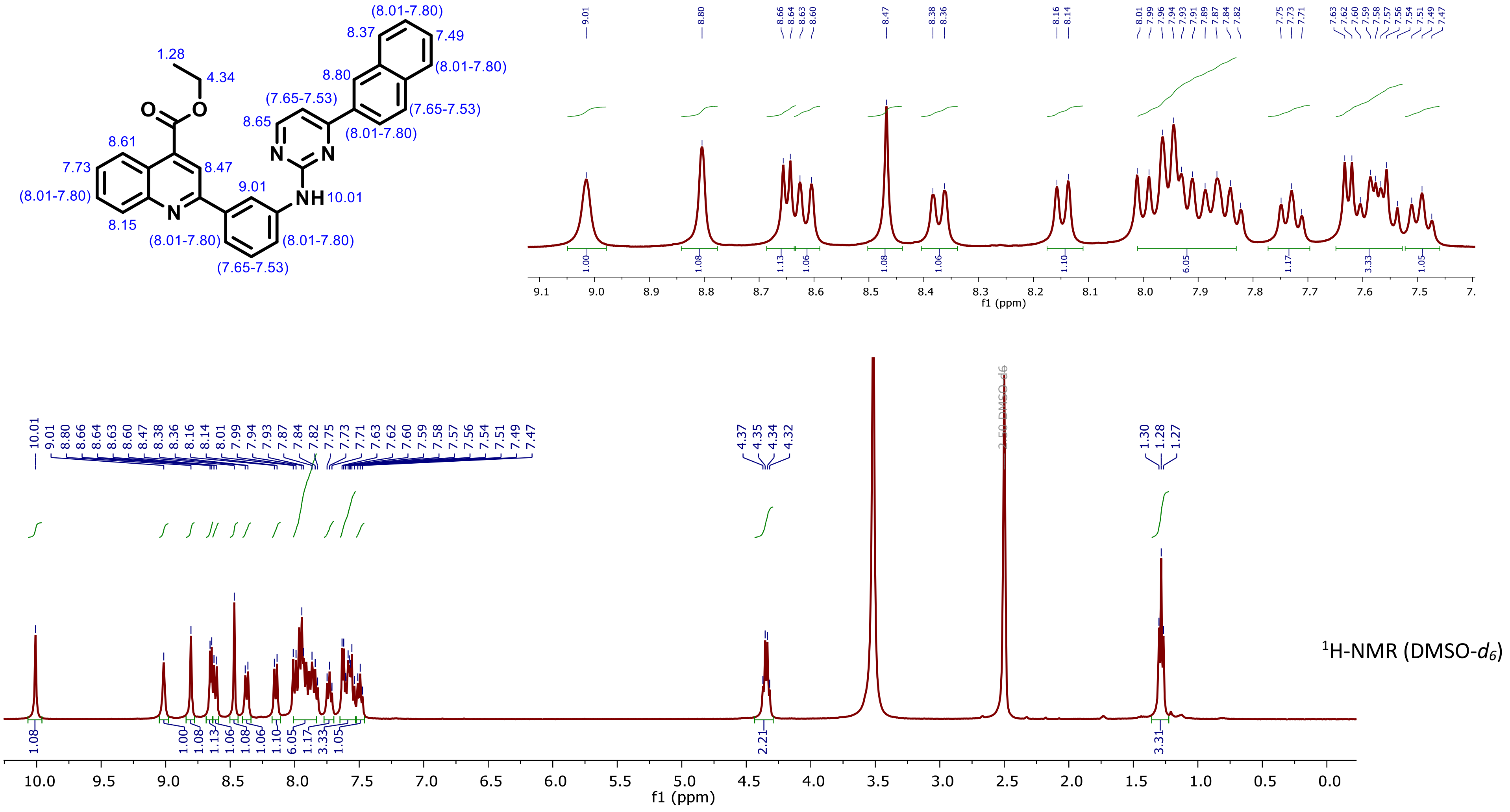

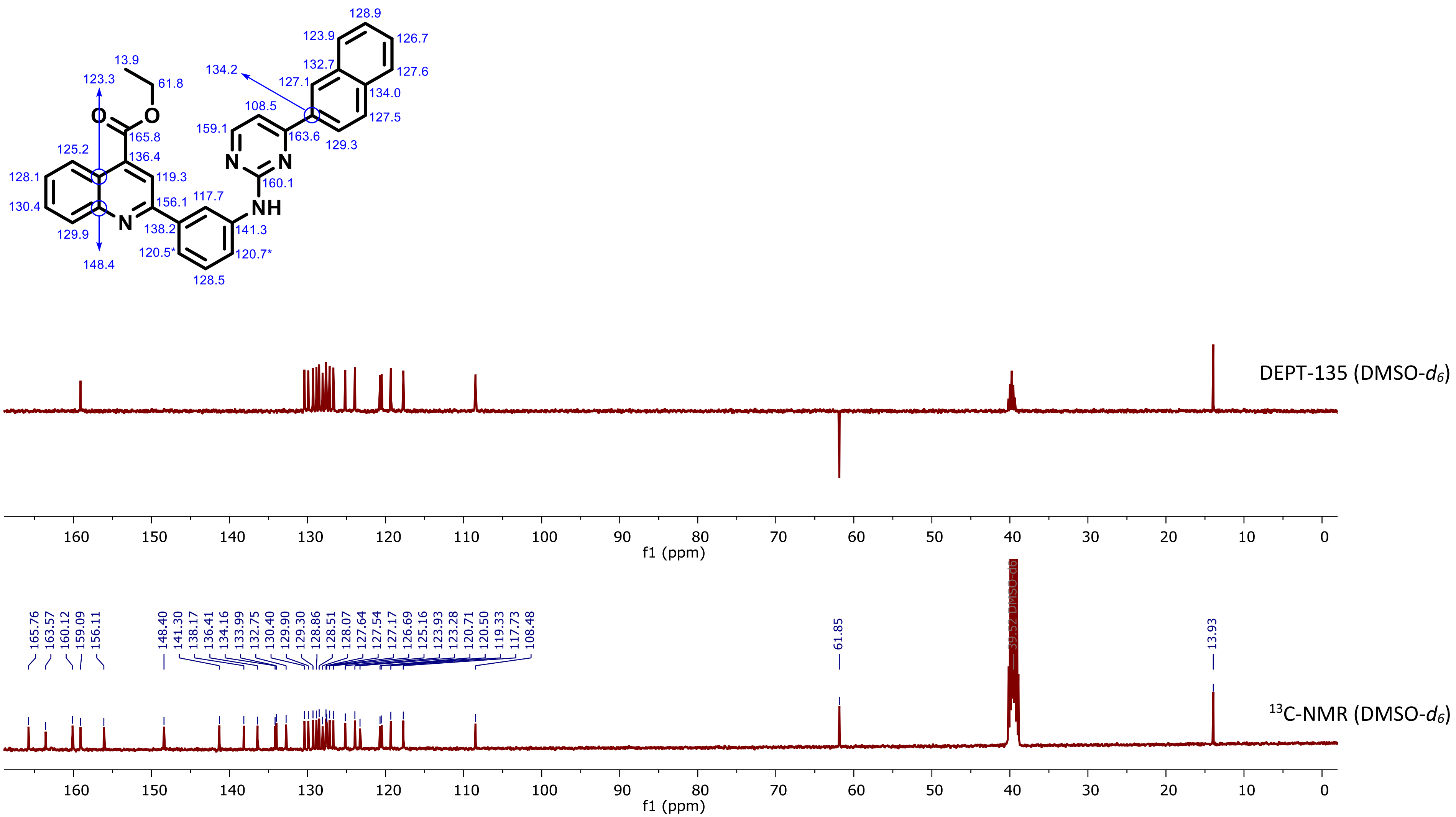
13.9
61.8
123.3
165.8
136.4
125.2
128.1
119.3
130.4
156.1
129.9
148.4
138.2
117.7
120.5*
128.5
120.7*
141.3
NH
160.1
159.1
108.5
163.6
134.2
127.1
132.7
123.9
128.9
126.7
127.6
134.0
127.5
129.3
DEPT-135 (DMSO-d6)
f1 (ppm)
165.76
163.57
160.12
159.09
156.11
148.40
141.30
138.17
136.41
134.16
133.99
132.75
130.40
129.90
129.30
128.86
128.51
128.07
127.64
127.54
127.17
126.69
125.16
123.93
123.28
120.71
120.50
119.33
117.73
108.48
61.85
39.52 DMSO-d6
13.93
13C-NMR (DMSO-d6)
f1 (ppm)

EI MS (70eV)

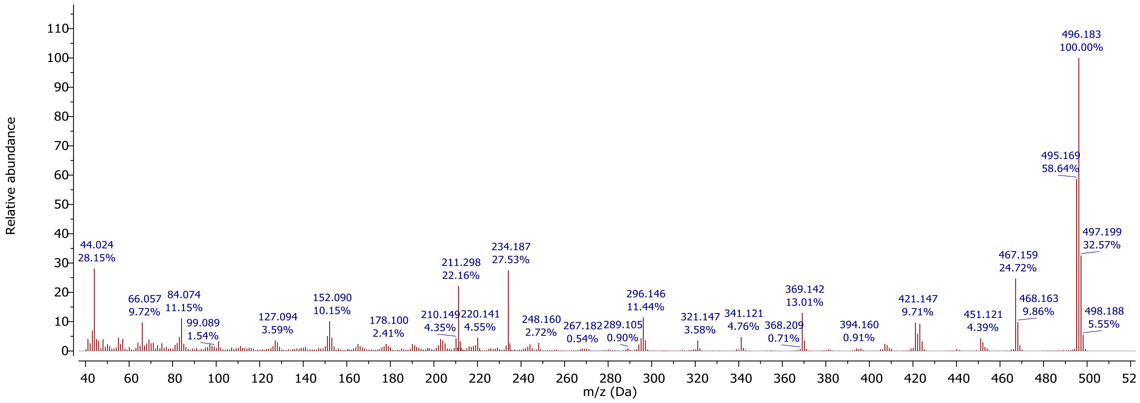

ESI-QTOF (positive ionization)

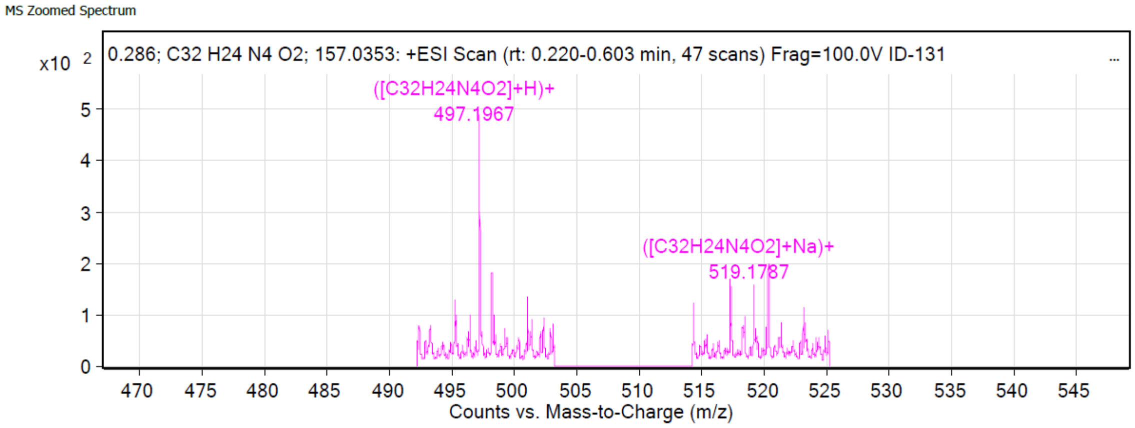

MS Spectrum Peak List

m/z	Calc *m/z*	Diff(ppm)	z	Abund	Formula	Ion
497.1967	497.1972	0.96	1	490.44	C32H24N4O2	(M+H)+
498.202	498.2003	-3.42	1	184.71	C32H24N4O2	(M+H)+
519.1787	519.1791	0.77	1	157.58	C32H24N4O2	(M+Na)+
520.1797	520.1823	4.9	1	75.74	C32H24N4O2	(M+Na)+

IR

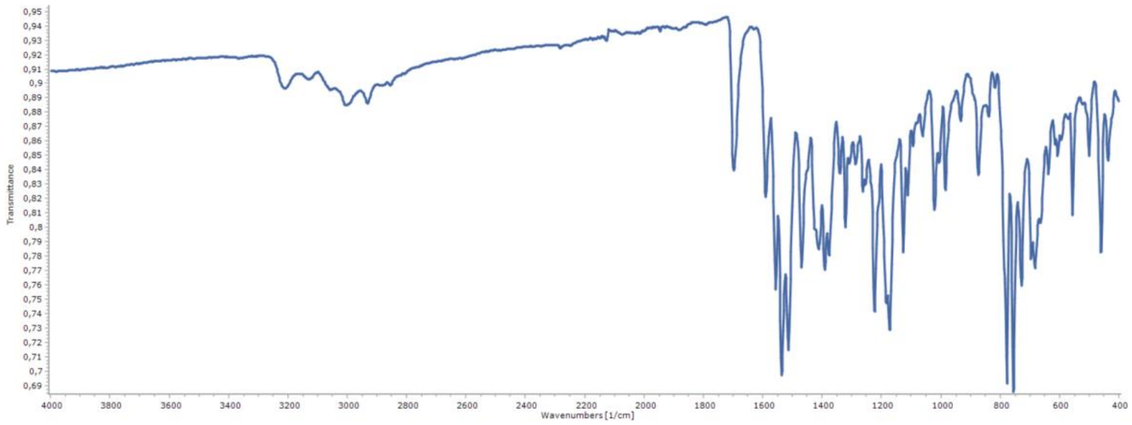

Compound **15a**

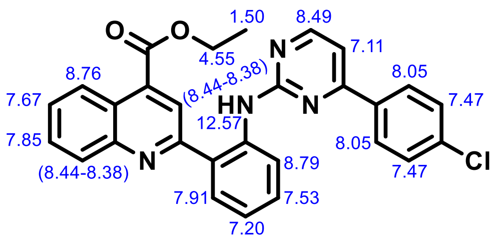

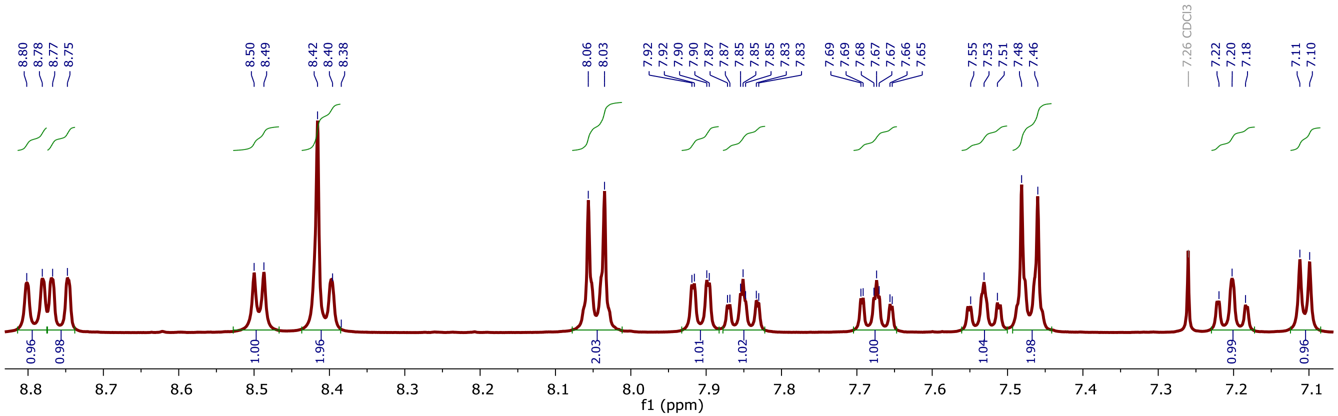

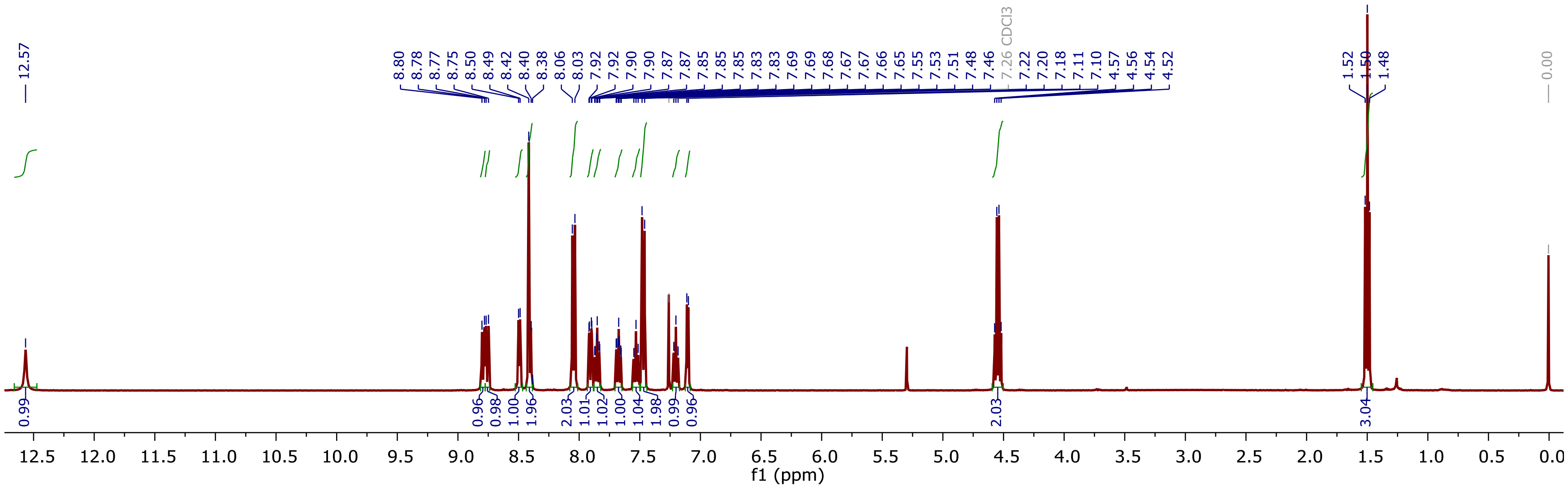

^{1}H-NMR ($CDCl_3$)

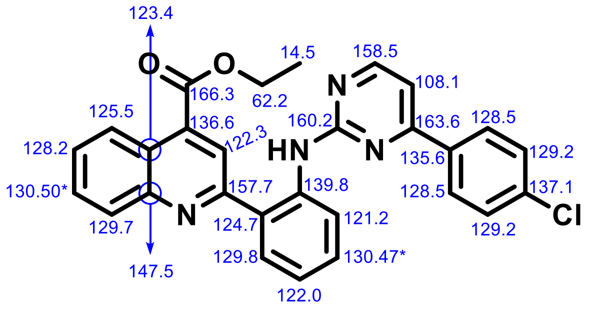
123.4
14.5
158.5
166.3
62.2
108.1
125.5
136.6
160.2
163.6
128.5
122.3
128.2
HN
135.6
129.2
139.8
130.50*
157.7
128.5
137.1
Cl
121.2
129.7
N
124.7
129.2
147.5
129.8
130.47*
122.0

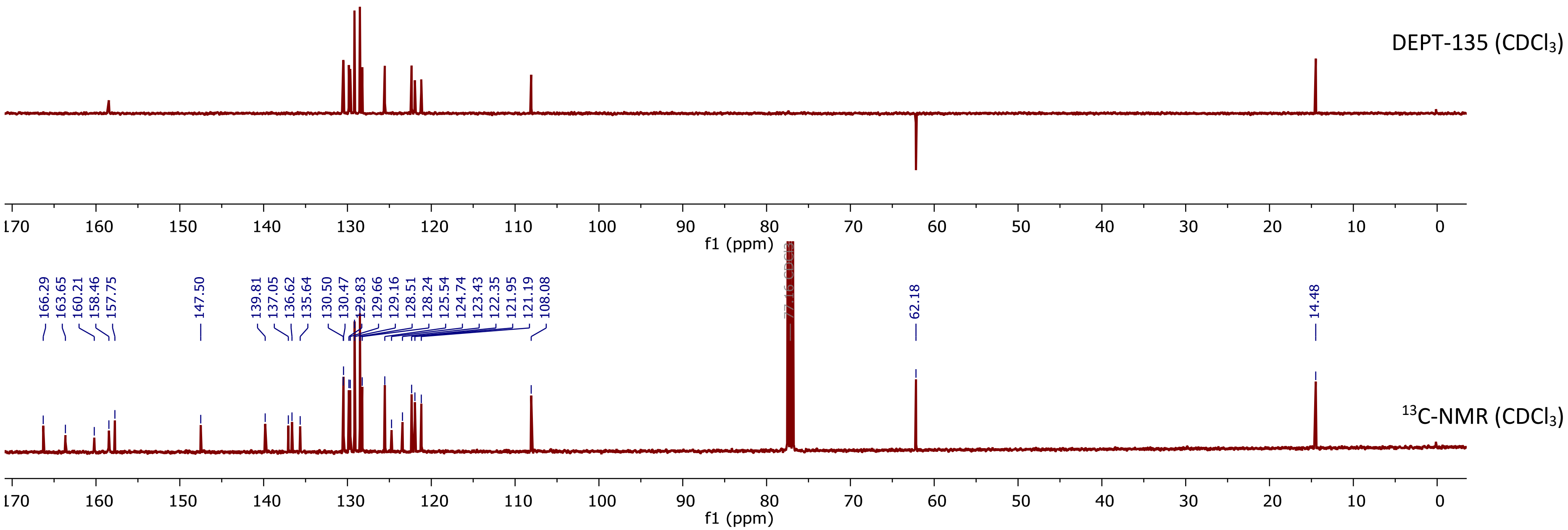
DEPT-135 ($CDCl_3$)
170 160 150 140 130 120 110 100 90 80 70 60 50 40 30 20 10 0
f1 (ppm)
166.29
163.65
160.21
158.46
157.75
147.50
139.81
137.05
136.62
135.64
130.50
130.47
129.83
129.66
129.16
128.51
128.24
125.54
124.74
123.43
122.35
121.95
121.19
108.08
77.16 CDCl3
62.18
14.48
^{13}C-NMR ($CDCl_3$)
170 160 150 140 130 120 110 100 90 80 70 60 50 40 30 20 10 0
f1 (ppm)

EI MS (70eV)

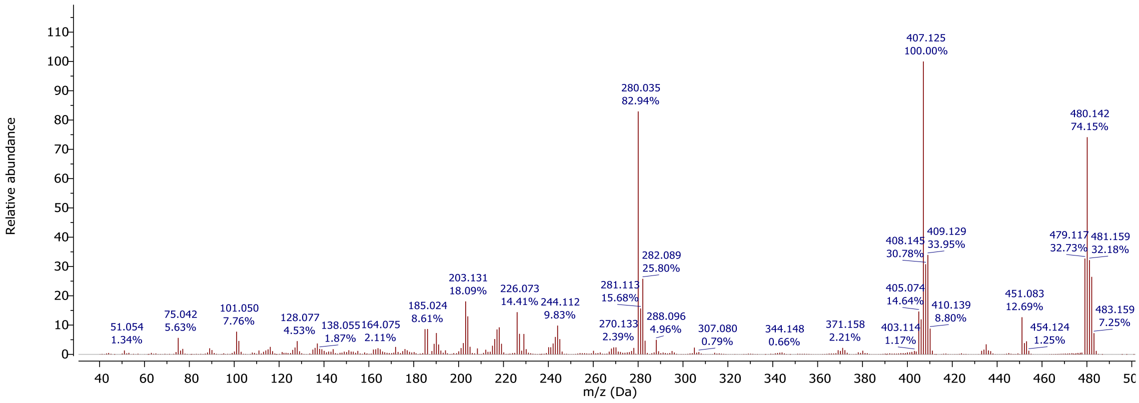

ESI-QTOF (positive ionization)

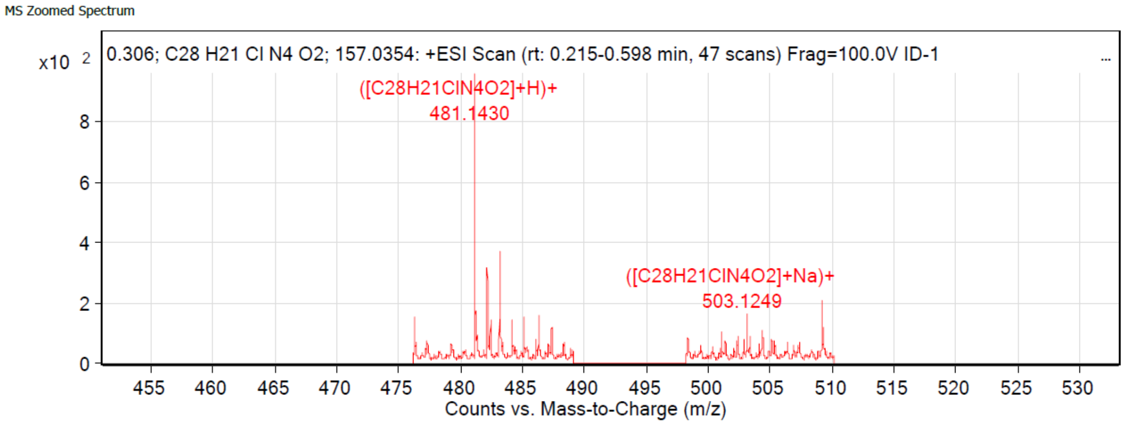

MS Spectrum Peak List

m/z	Calc *m/z*	Diff(ppm)	z	Abund	Formula	Ion
481.143	481.1426	-0.86	1	958.46	C28H21ClN4O2	(M+H)+
482.1492	482.1457	-7.24	1	319.89	C28H21ClN4O2	(M+H)+
483.1421	483.1409	-2.53	1	372.52	C28H21ClN4O2	(M+H)+
484.1443	484.1432	-2.3	1	144.92	C28H21ClN4O2	(M+H)+
503.1249	503.1245	-0.83	1	162.5	C28H21ClN4O2	(M+Na)+
504.1304	504.1276	-5.45	1	65.44	C28H21ClN4O2	(M+Na)+
505.1271	505.1229	-8.34	1	78.57	C28H21ClN4O2	(M+Na)+

IR

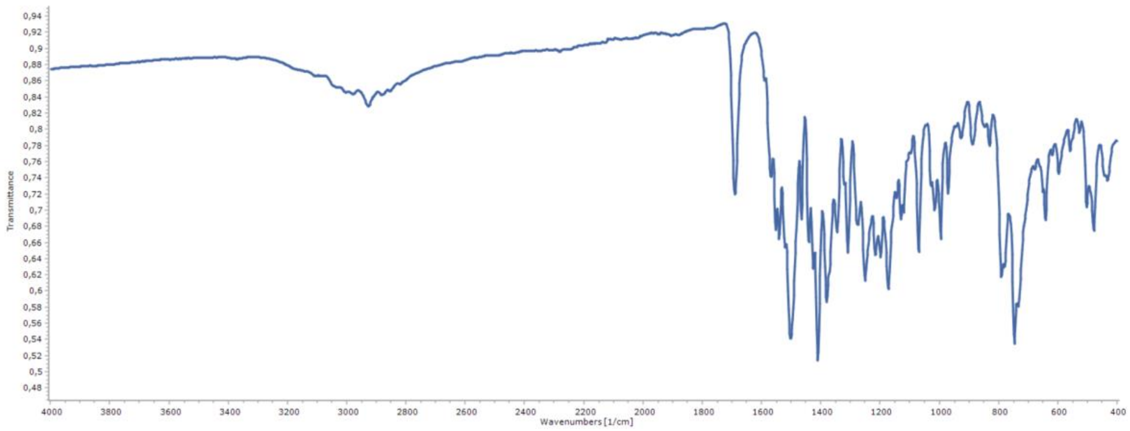

Compound **15b**

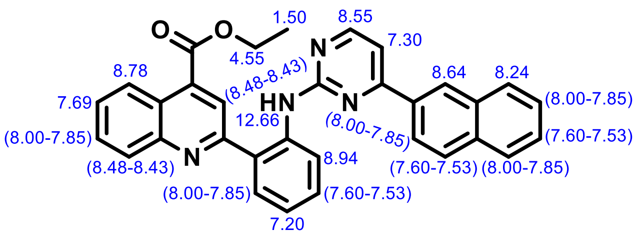

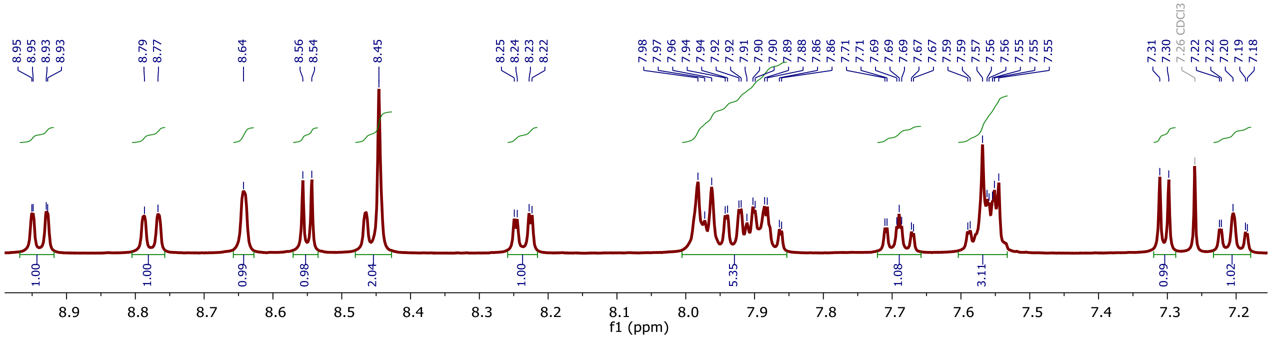

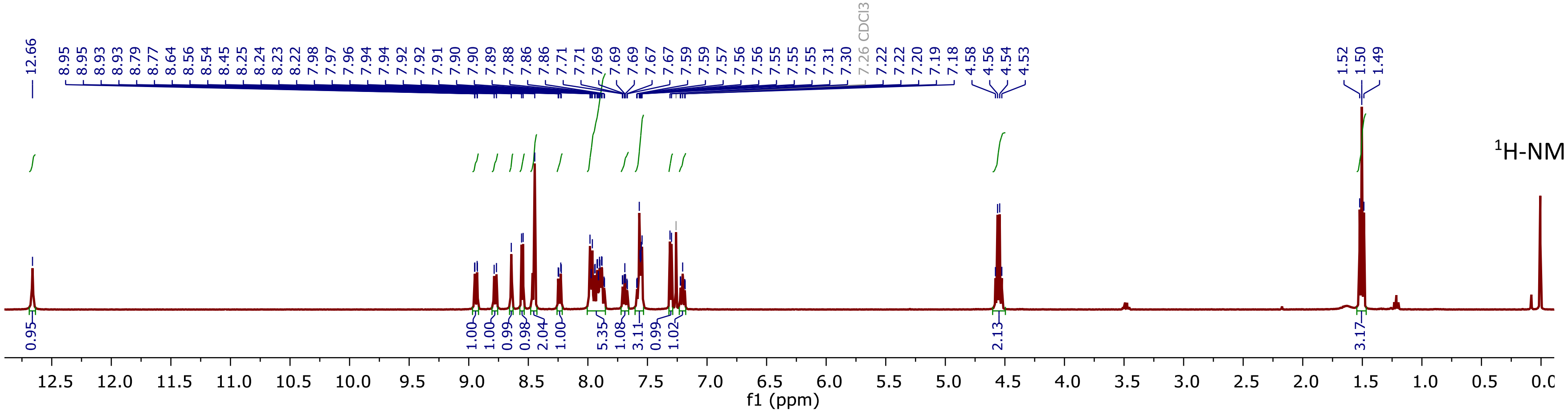

^{1}H-NMR ($CDCl_3$)

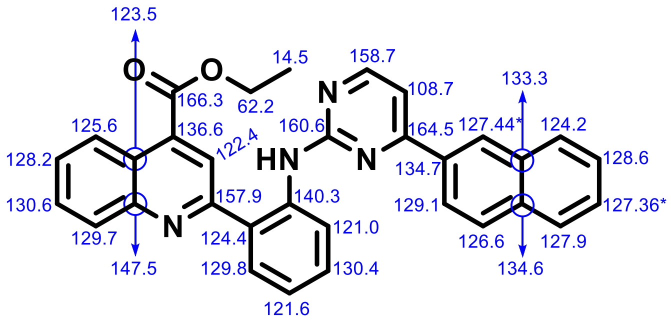

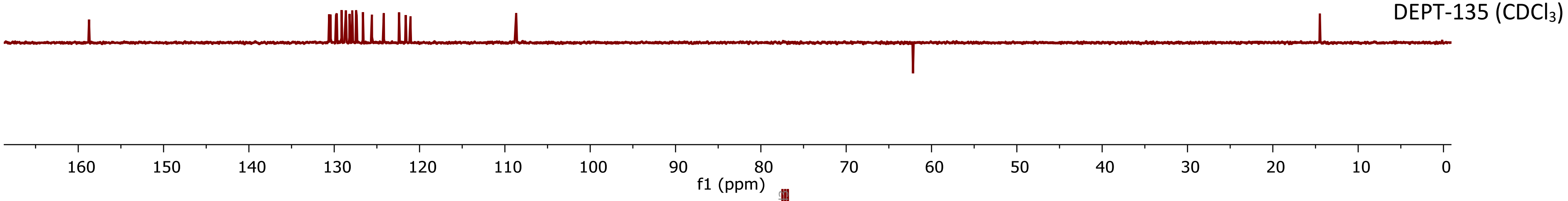

DEPT-135 (CDCl3)
f1 (ppm)

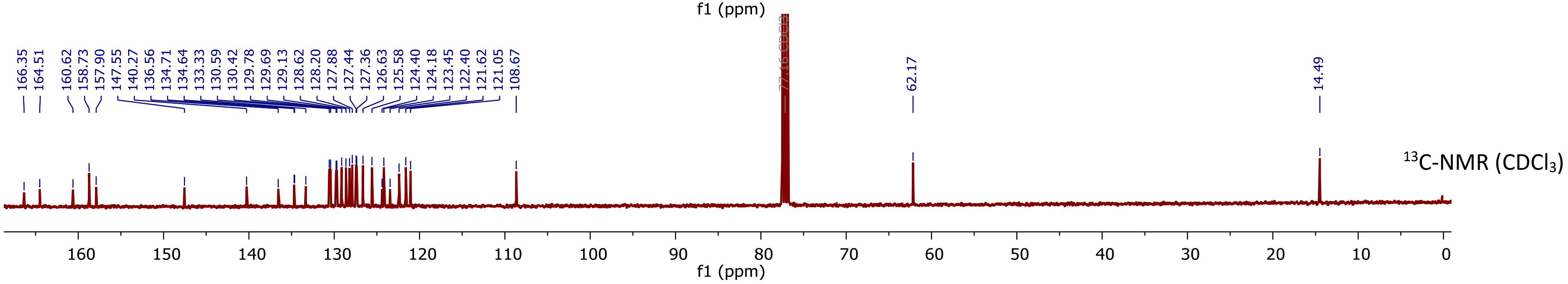

13C-NMR (CDCl3)
f1 (ppm)

EI MS (70eV)

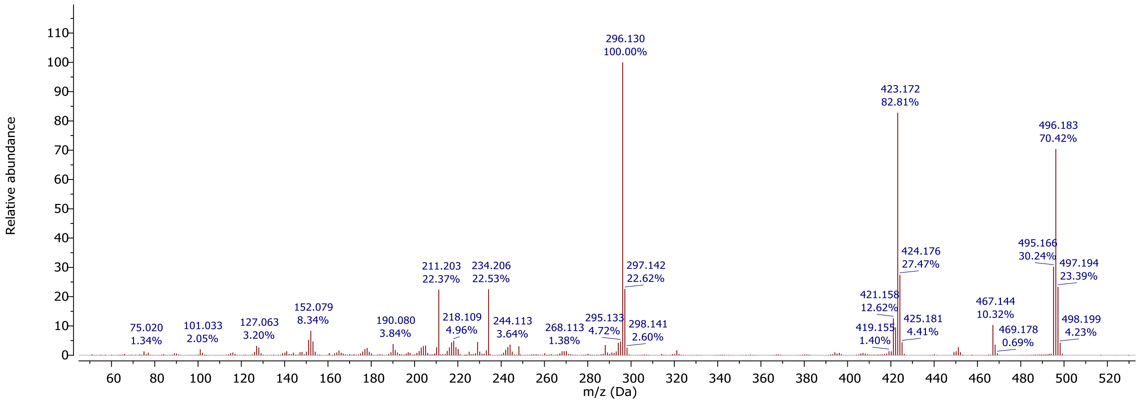

ESI-QTOF (positive ionization)

MS Zoomed Spectrum

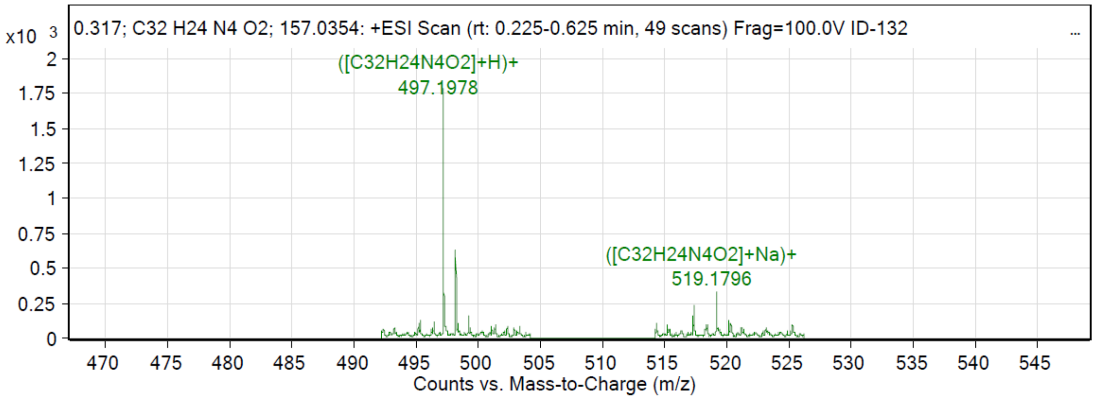

MS Spectrum Peak List

m/z	Calc *m/z*	Diff(ppm)	z	Abund	Formula	Ion
497.1978	497.1972	-1.27	1	1785.93	C32H24N4O2	(M+H)+
498.201	498.2003	-1.35	1	630.23	C32H24N4O2	(M+H)+
499.205	499.2033	-3.42	1	158.46	C32H24N4O2	(M+H)+
519.1796	519.1791	-0.93	1	333.82	C32H24N4O2	(M+Na)+
520.1818	520.1823	0.96	1	127.21	C32H24N4O2	(M+Na)+
521.1743	521.1853	21.01	1	73.03	C32H24N4O2	(M+Na)+

IR

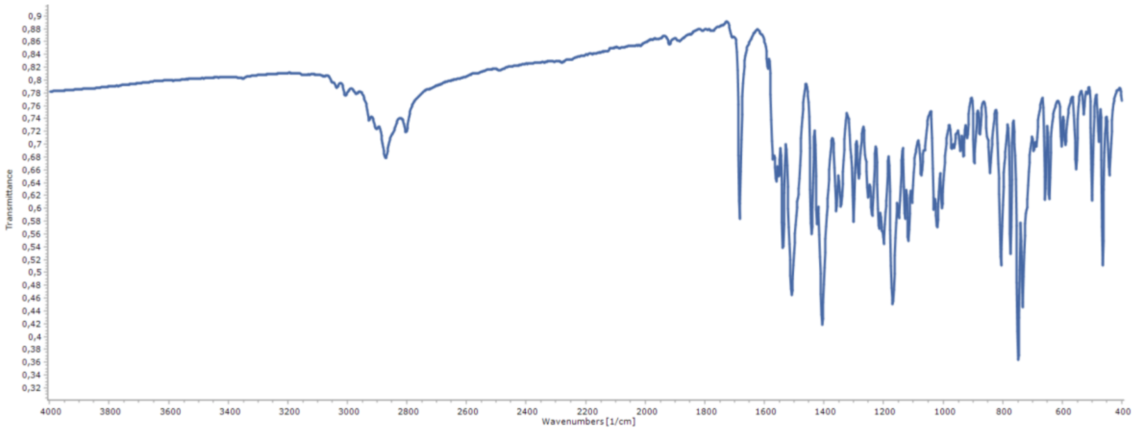

Compound **15c**

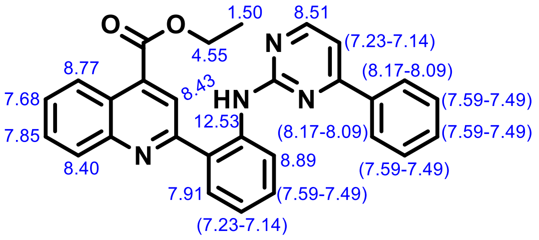

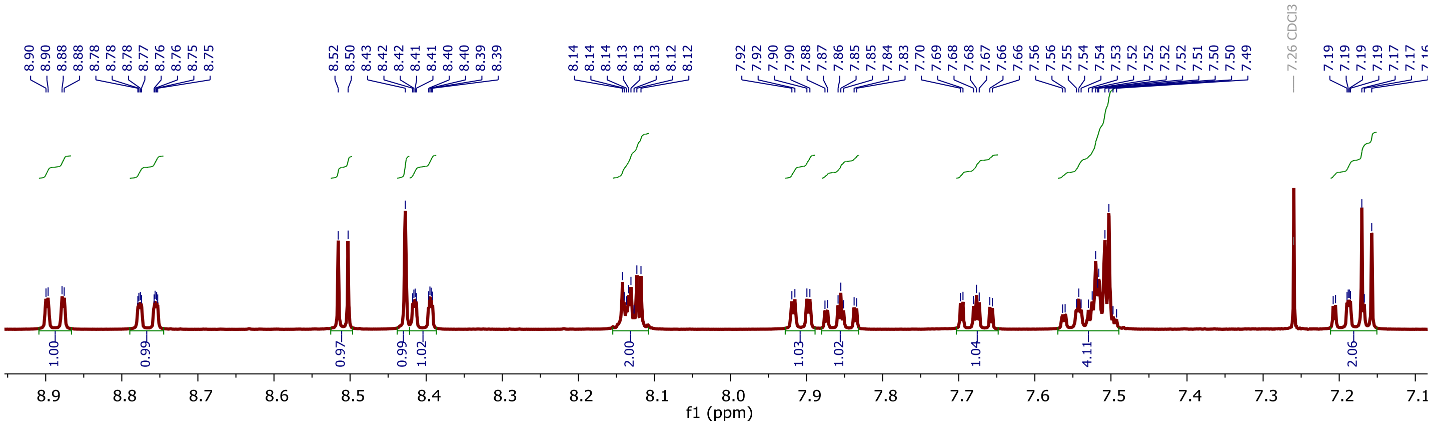

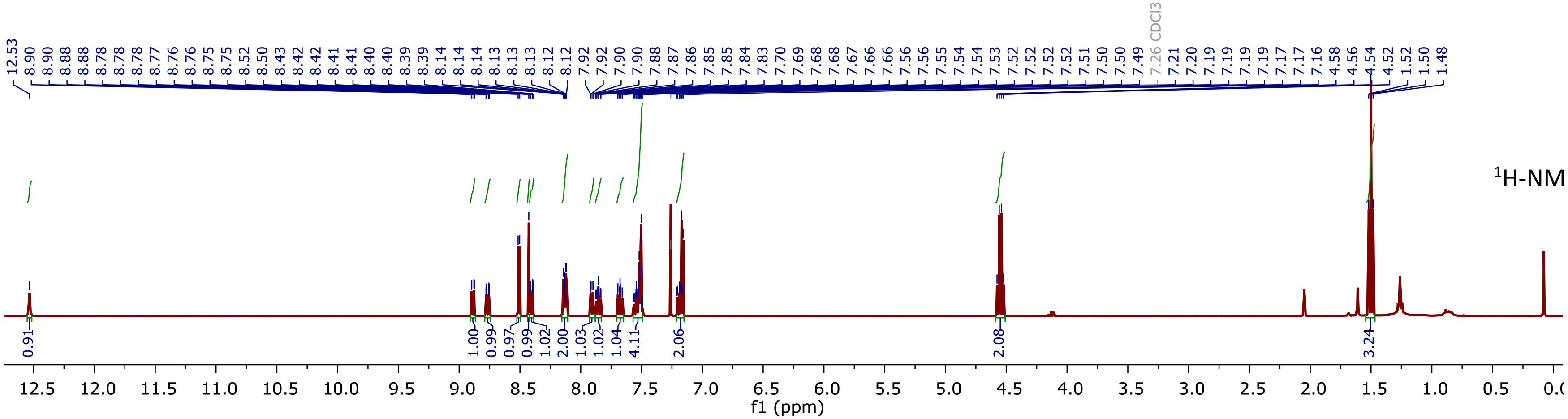

^{1}H-NMR ($CDCl_3$)

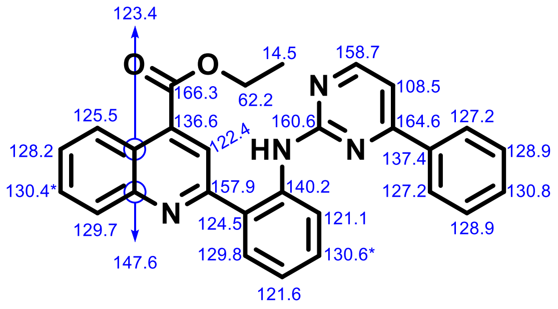

123.4
14.5
158.7
166.3
62.2
108.5
125.5
136.6
160.6
164.6
127.2
128.2
122.4
HN
137.4
128.9
130.4*
157.9
140.2
127.2
130.8
129.7
N
124.5
121.1
128.9
147.6
129.8
130.6*
121.6

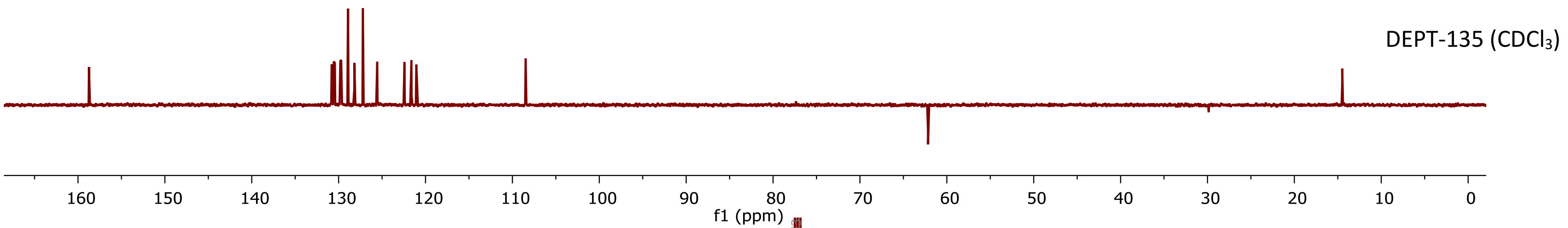

DEPT-135 (CDCl3)
160 150 140 130 120 110 100 90 80 70 60 50 40 30 20 10 0
f1 (ppm)

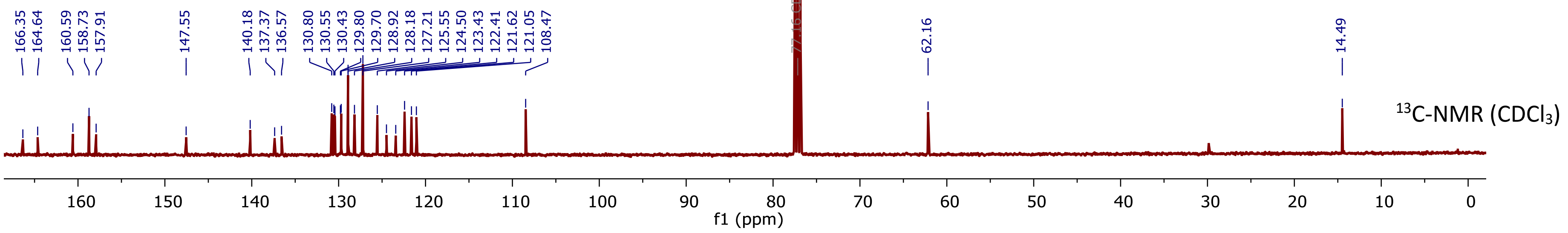

166.35
164.64
160.59
158.73
157.91
147.55
140.18
137.37
136.57
130.80
130.55
130.43
129.80
129.70
128.92
128.18
127.21
125.55
124.50
123.43
122.41
121.62
121.05
108.47
77.16 CDCl3
62.16
14.49
13C-NMR (CDCl3)
160 150 140 130 120 110 100 90 80 70 60 50 40 30 20 10 0
f1 (ppm)

EI MS (70eV)

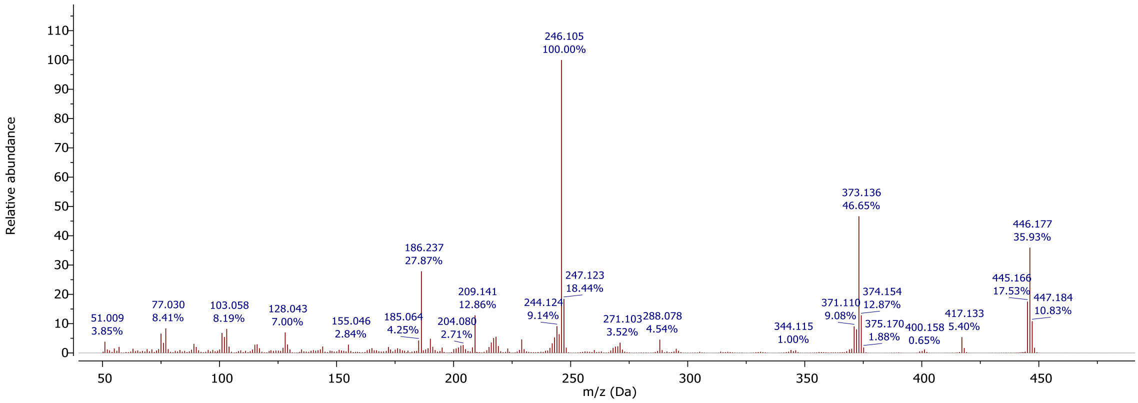

ESI-QTOF (positive ionization)

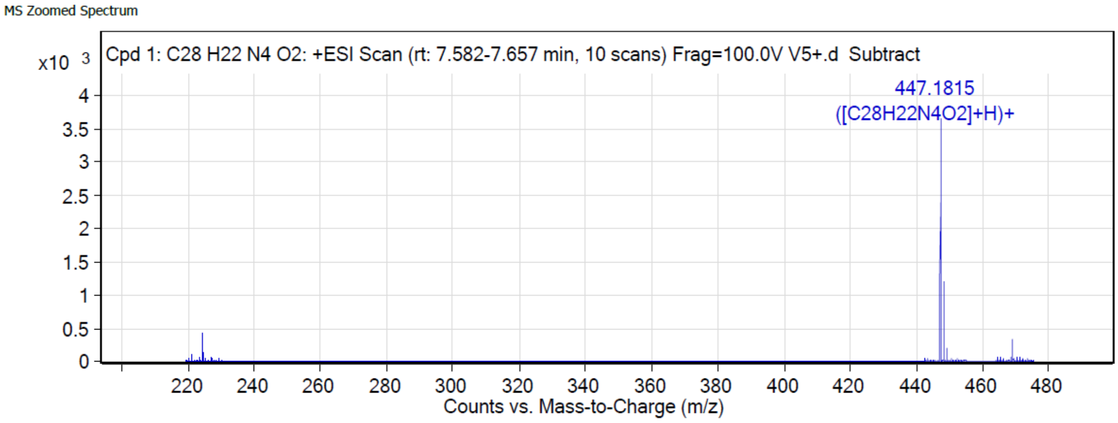

MS Spectrum Peak List

m/z	Calc *m/z*	Diff(ppm)	z	Abund	Formula	Ion
224.0945	224.0944	-0.17	2	424.48	C28H22N4O2	(M+2H)+2
224.5961	224.596	-0.65	2	142.09	C28H22N4O2	(M+2H)+2
225.1031	225.0974	-25.18	2	29.09	C28H22N4O2	(M+2H)+2
447.1815	447.1816	0.12	1	3716.82	C28H22N4O2	(M+H)+
448.185	448.1846	-0.73	1	1213.93	C28H22N4O2	(M+H)+
449.1875	449.1876	0.19	1	205.03	C28H22N4O2	(M+H)+
450.1954	450.1904	-11.02	1	28.23	C28H22N4O2	(M+H)+
469.1622	469.1635	2.8	1	338.05	C28H22N4O2	(M+Na)+
470.1666	470.1666	-0.12	1	77.08	C28H22N4O2	(M+Na)+

IR

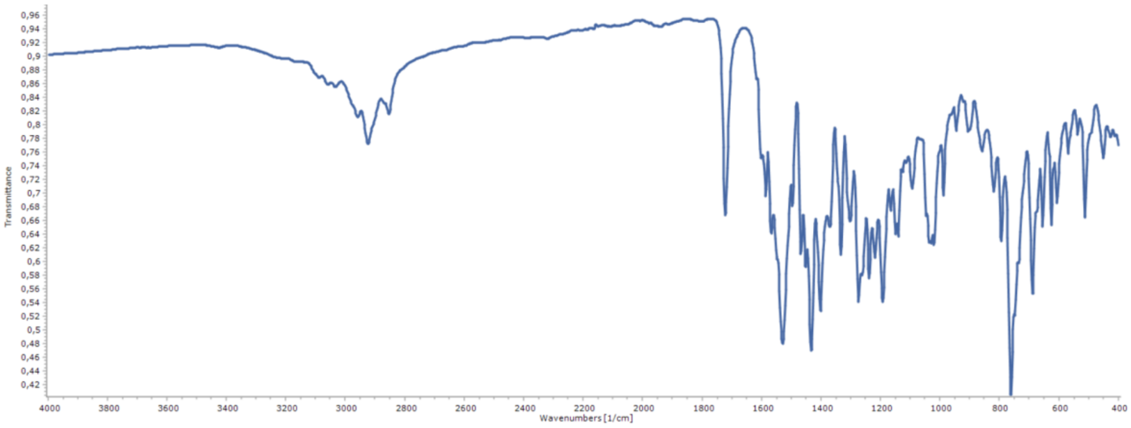

Compound **15d**

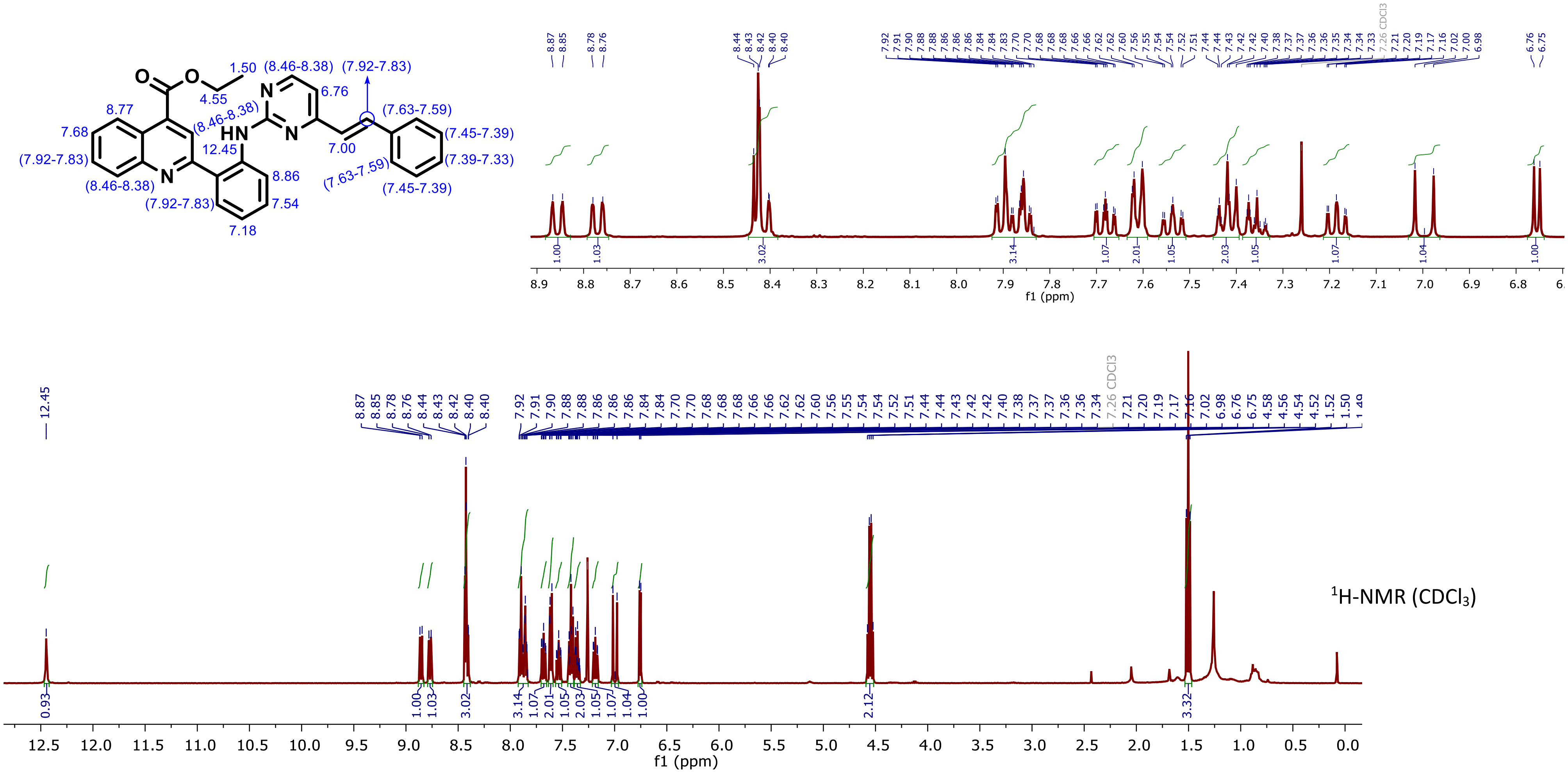

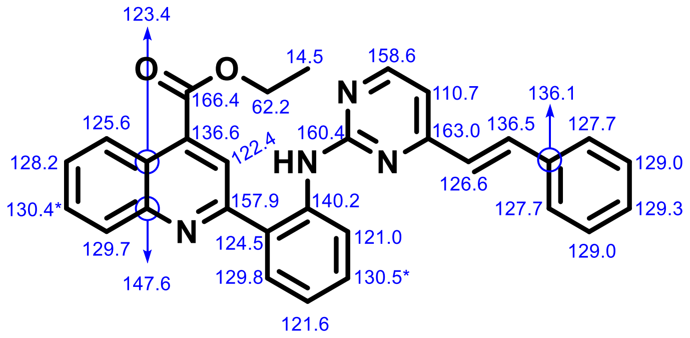

123.4
14.5
158.6
166.4
62.2
110.7
136.1
125.6
136.6
122.4
160.4
163.0
136.5
127.7
128.2
HN
N
O
129.0
157.9
140.2
126.6
130.4*
127.7
129.3
121.0
129.7
124.5
129.0
147.6
129.8
130.5*
121.6

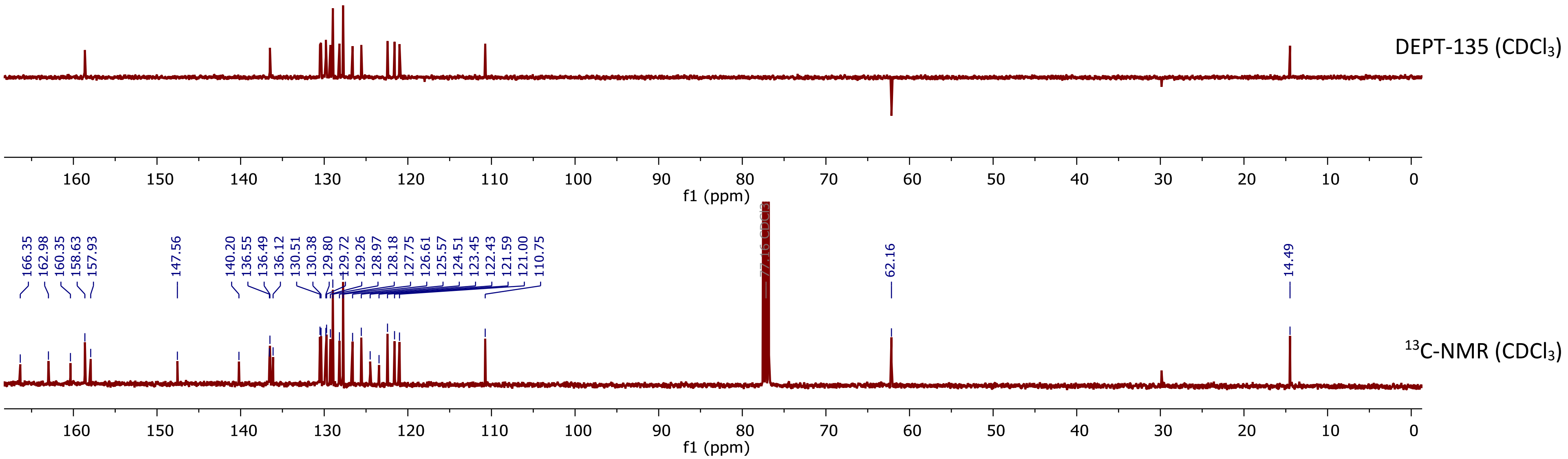

DEPT-135 (CDCl3)
160
150
140
130
120
110
100
90
80
70
60
50
40
30
20
10
0
f1 (ppm)
166.35
162.98
160.35
158.63
157.93
147.56
140.20
136.55
136.49
136.12
130.51
130.38
129.80
129.72
129.26
128.97
128.18
127.75
126.61
125.57
124.51
123.45
122.43
121.59
121.00
110.75
77.16 CDCl3
62.16
14.49
13C-NMR (CDCl3)

EI MS (70eV)

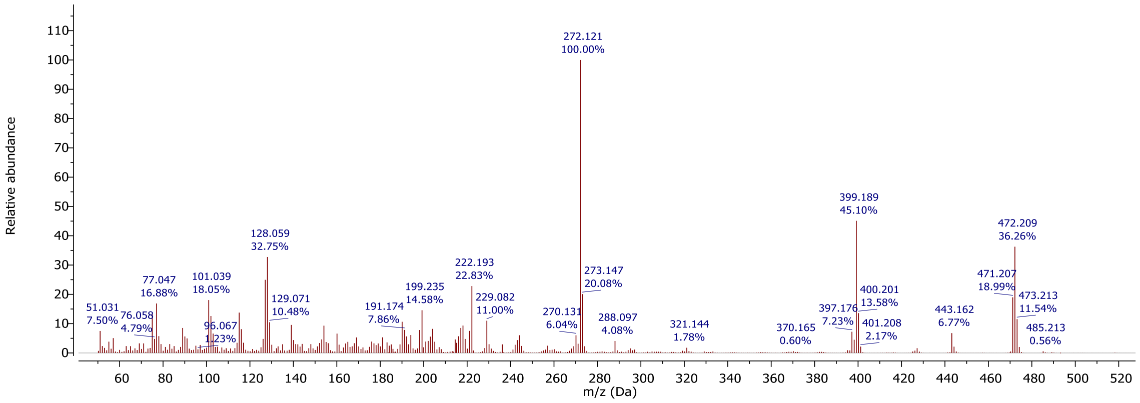

ESI-QTOF (positive ionization)

MS Zoomed Spectrum

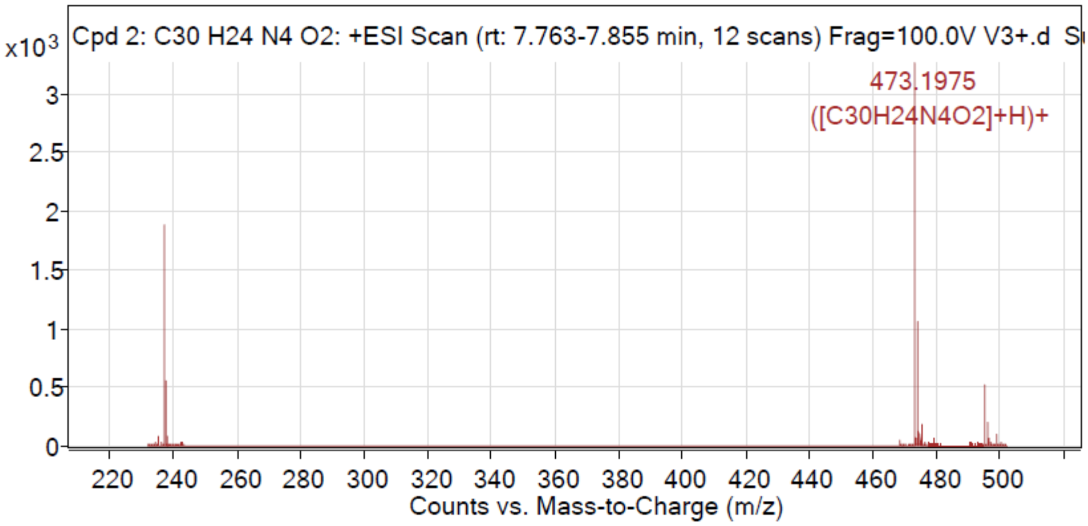

MS Spectrum Peak List

m/z	Calc *m/z*	Diff(ppm)	z	Abund	Formula	Ion
237.102	237.1022	0.88	2	1889.49	C30H24N4O2	(M+2H)+2
237.6038	237.6038	0.14	2	556.44	C30H24N4O2	(M+2H)+2
238.1072	238.1053	-8.09	2	83.07	C30H24N4O2	(M+2H)+2
473.1975	473.1972	-0.69	1	3276.68	C30H24N4O2	(M+H)+
474.1992	474.2003	2.26	1	1068.28	C30H24N4O2	(M+H)+
475.2035	475.2033	-0.36	1	180.66	C30H24N4O2	(M+H)+
476.2018	476.2061	9.07	1	27.33	C30H24N4O2	(M+H)+
495.1783	495.1791	1.73	1	518.96	C30H24N4O2	(M+Na)+
496.1815	496.1823	1.52	1	205.6	C30H24N4O2	(M+Na)+
497.1807	497.1852	9.06	1	35.19	C30H24N4O2	(M+Na)+

IR

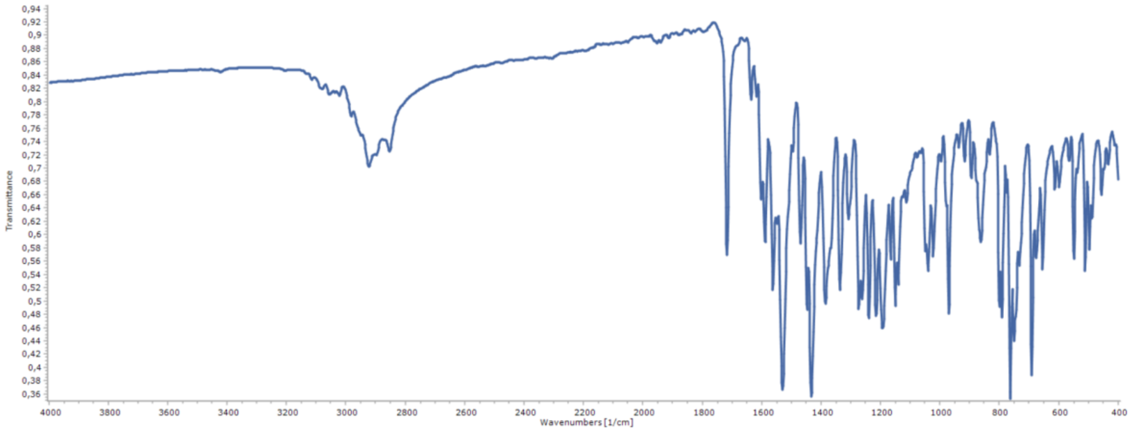

Compound **16a**

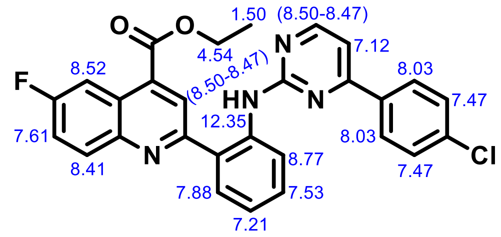

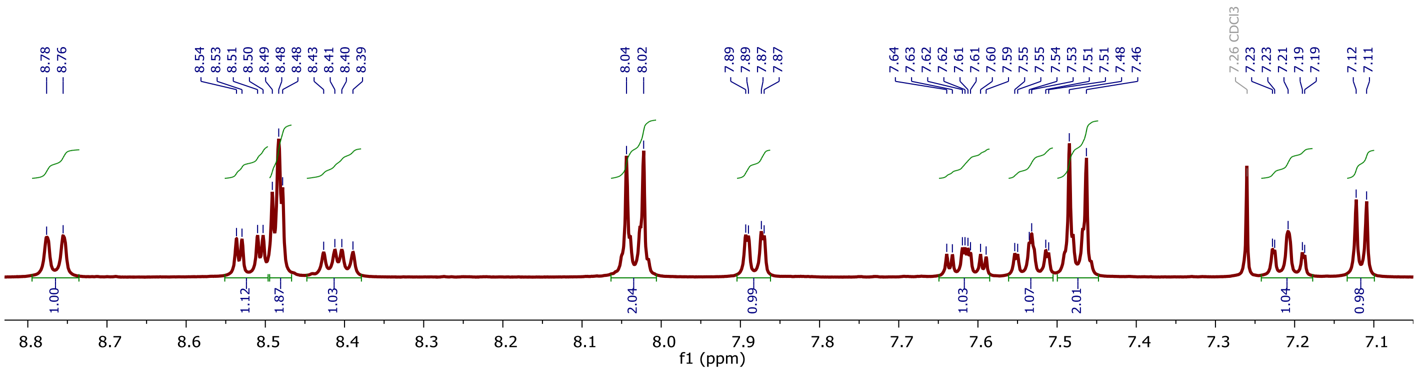

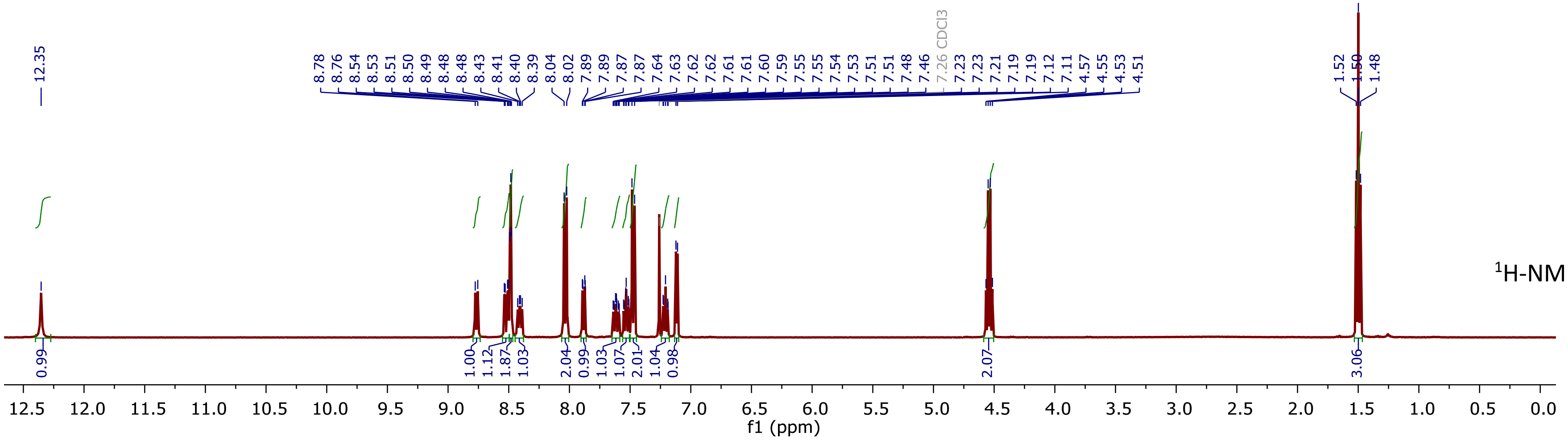

^{1}H-NMR ($CDCl_3$)

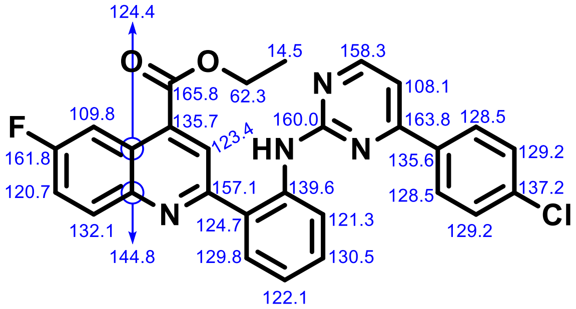
124.4
O
O
14.5
158.3
N
62.3
165.8
108.1
F
109.8
135.7
160.0
163.8
128.5
123.4
HN
N
161.8
135.6
129.2
157.1
139.6
137.2
120.7
128.5
N
Cl
132.1
124.7
121.3
129.2
144.8
129.8
130.5
122.1

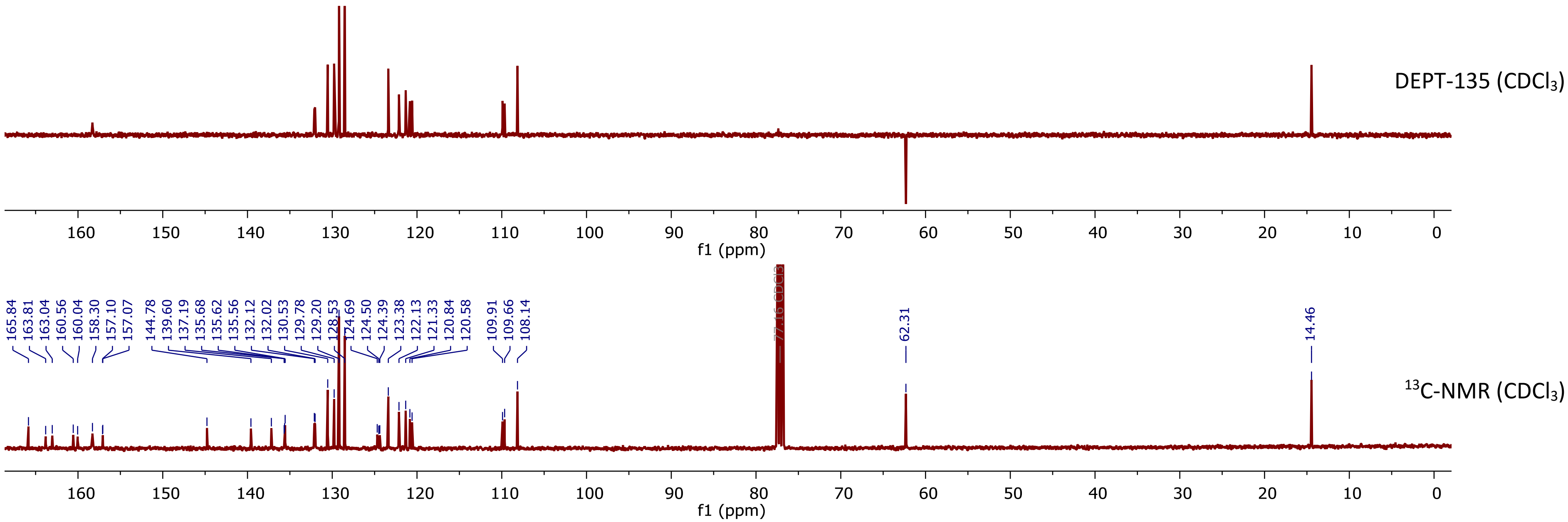
DEPT-135 (CDCl3)
160 150 140 130 120 110 100 90 80 70 60 50 40 30 20 10 0
f1 (ppm)
165.84
163.81
163.04
160.56
160.04
158.30
157.10
157.07
144.78
139.60
137.19
135.68
135.62
135.56
132.12
132.02
130.53
129.78
129.20
128.53
124.69
124.50
124.39
123.38
122.13
121.33
120.84
120.58
109.91
109.66
108.14
77.16 CDCl3
62.31
14.46
13C-NMR (CDCl3)
160 150 140 130 120 110 100 90 80 70 60 50 40 30 20 10 0
f1 (ppm)

^{13}C-^{19}F couplings in ^{13}C-NMR. Only coupled peaks have been represented to make it easier to understand.

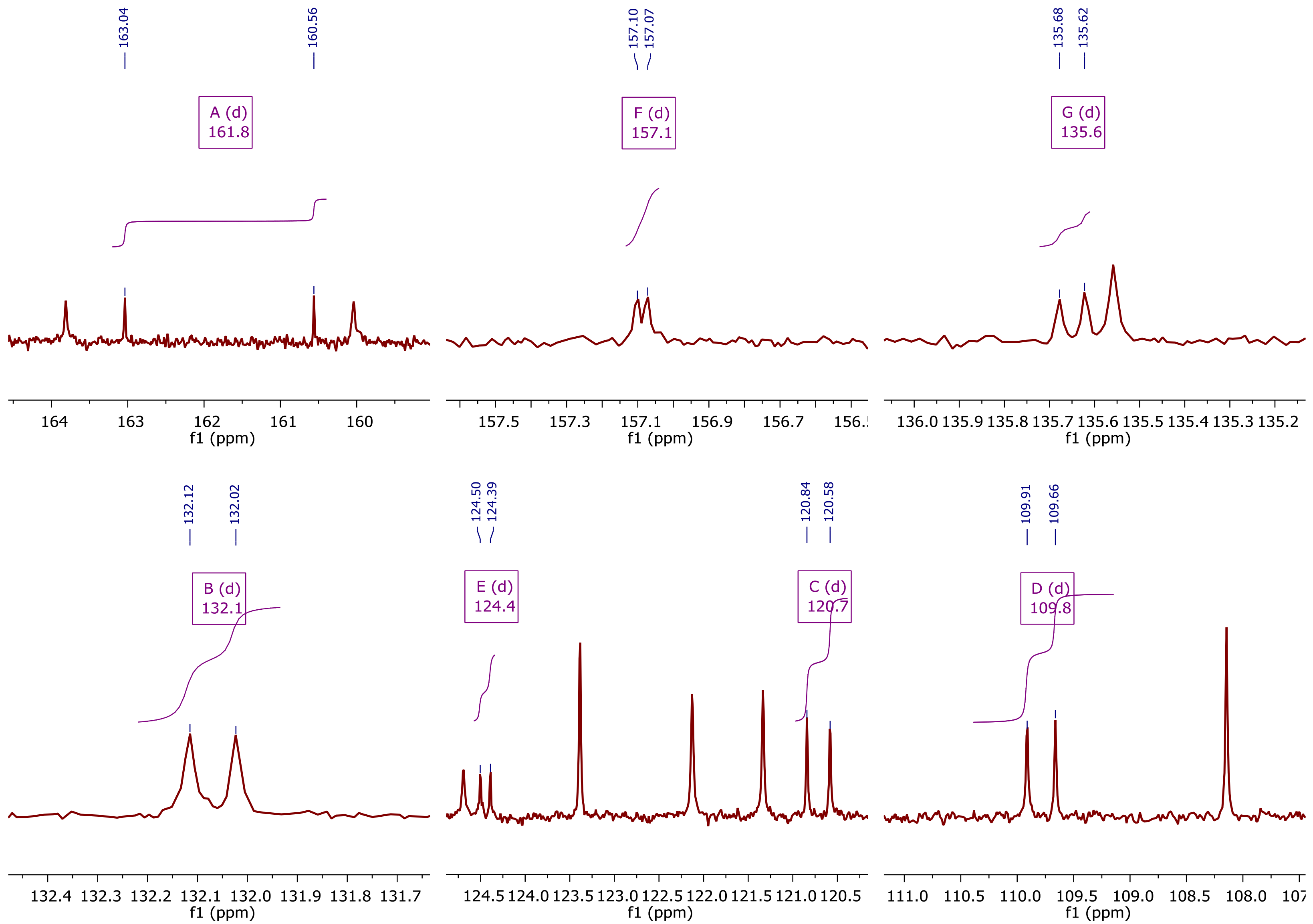

EI MS (70eV)

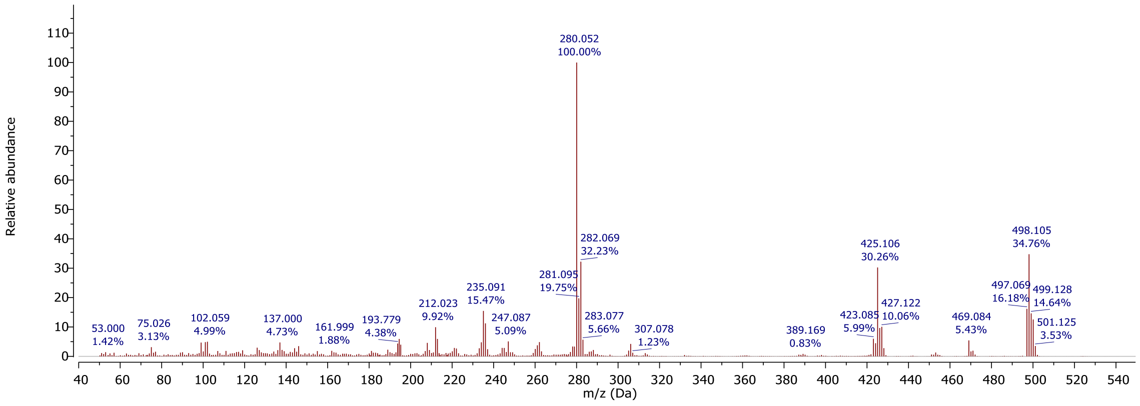

ESI-QTOF (positive ionization)

MS Zoomed Spectrum

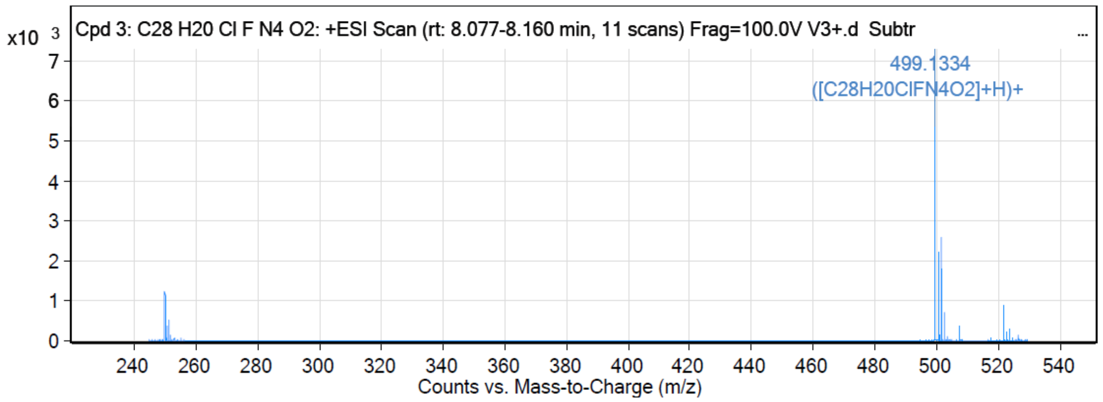

MS Spectrum Peak List

m/z	Calc *m/z*	Diff(ppm)	z	Abund	Formula	Ion
250.0705	250.0702	-1.26	2	1256.05	C28H20ClFN4O2	(M+2H)+2
250.5715	250.5718	0.99	2	369.37	C28H20ClFN4O2	(M+2H)+2
251.0693	251.0694	0.55	2	517.77	C28H20ClFN4O2	(M+2H)+2
499.1334	499.1332	-0.45	1	7497.92	C28H20ClFN4O2	(M+H)+
500.136	500.1362	0.44	1	2226.17	C28H20ClFN4O2	(M+H)+
501.1321	501.1315	-1.25	1	2621.84	C28H20ClFN4O2	(M+H)+
502.1341	502.1338	-0.67	1	708.56	C28H20ClFN4O2	(M+H)+
521.1152	521.1151	-0.1	1	907.89	C28H20ClFN4O2	(M+Na)+
522.1182	522.1182	-0.05	1	228.96	C28H20ClFN4O2	(M+Na)+
523.1112	523.1134	4.25	1	300.4	C28H20ClFN4O2	(M+Na)+

IR

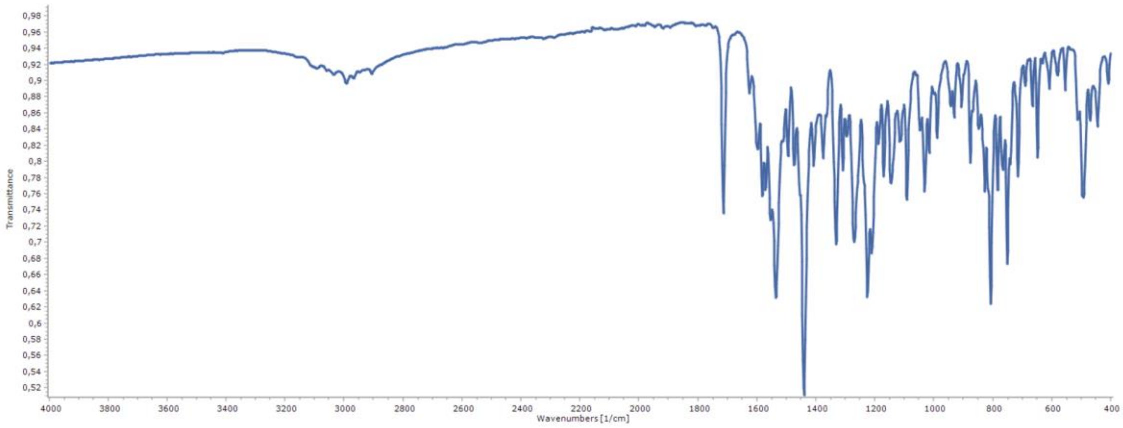

Compound **16b**

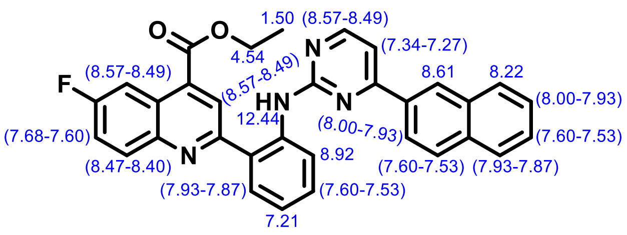

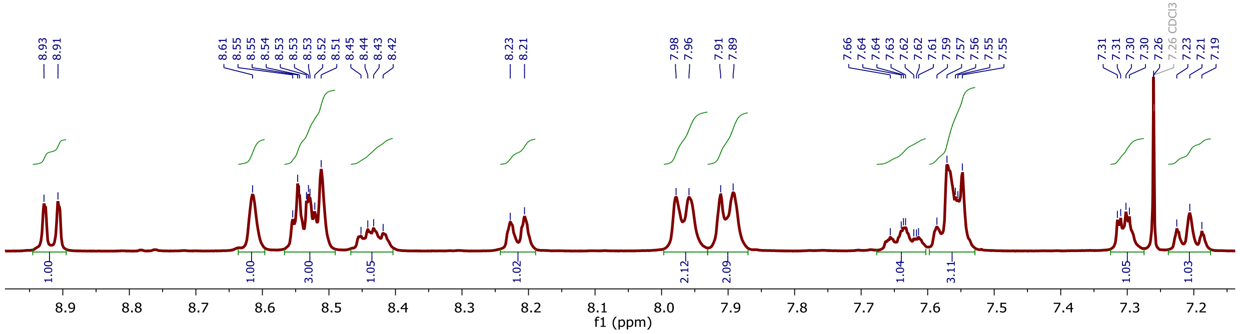

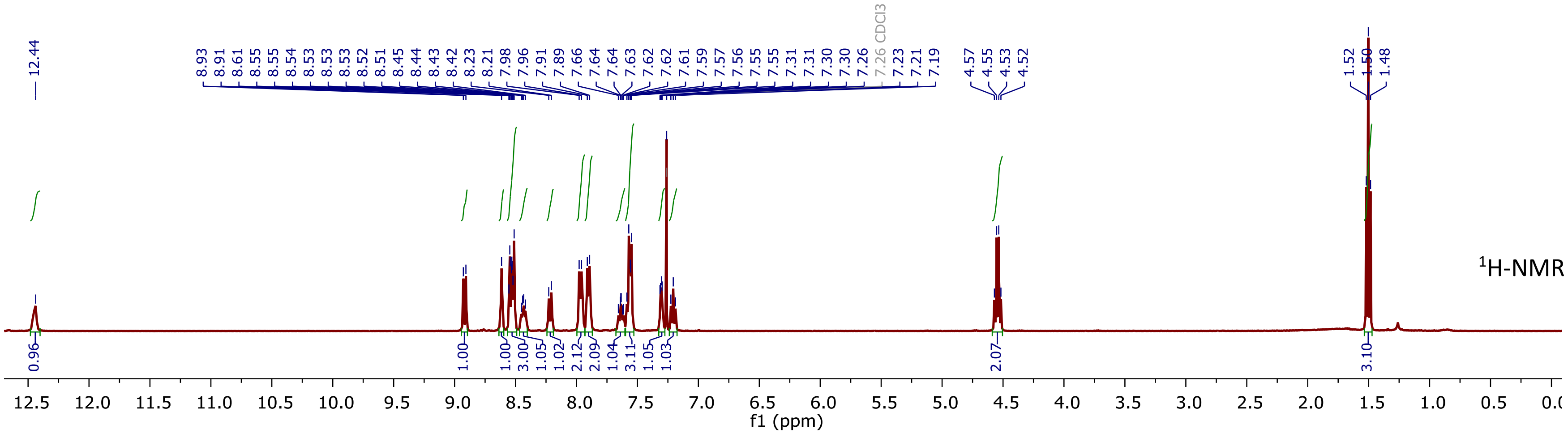

^{1}H-NMR ($CDCl_3$)

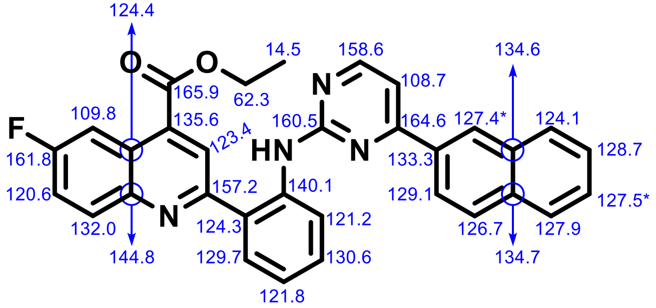

124.4
14.5
158.6
134.6
165.9
62.3
108.7
109.8
135.6
160.5
164.6
127.4*
124.1
123.4
133.3
128.7
F
161.8
140.1
120.6
157.2
129.1
127.5*
132.0
124.3
121.2
126.7
127.9
144.8
129.7
130.6
134.7
121.8

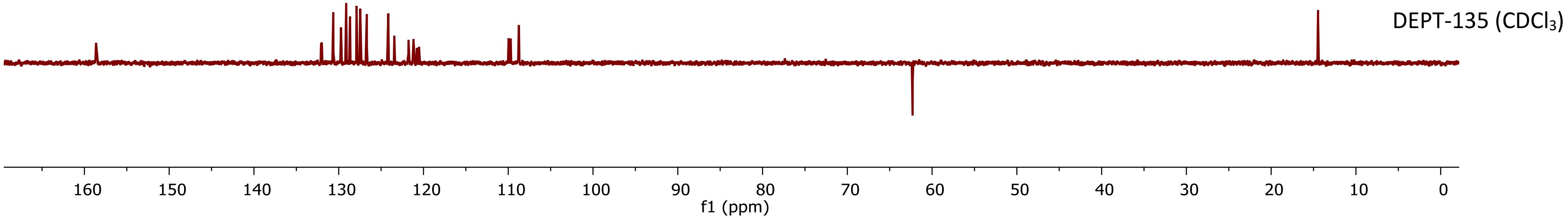

DEPT-135 (CDCl3)
f1 (ppm)

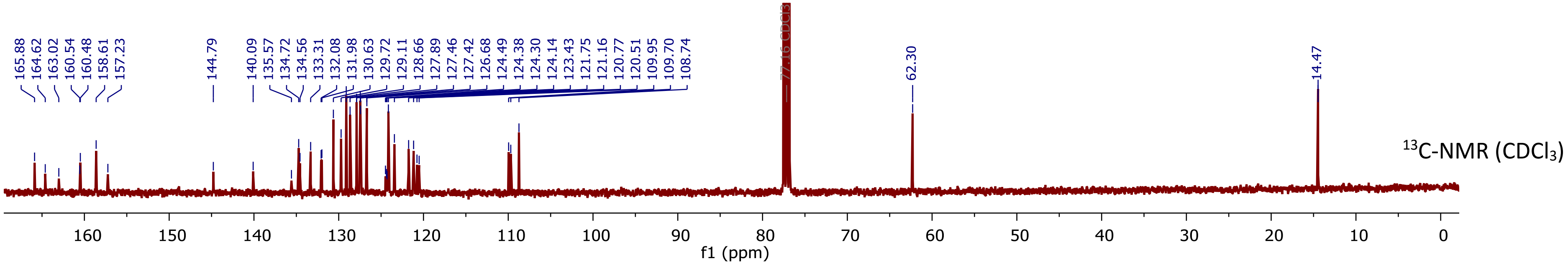

165.88
164.62
163.02
160.54
160.48
158.61
157.23
144.79
140.09
135.57
134.72
134.56
133.31
132.08
131.98
130.63
129.72
129.11
128.66
127.89
127.46
127.42
126.68
124.49
124.38
124.30
124.14
123.43
121.75
121.16
120.77
120.51
109.95
109.70
108.74
77.16 CDCl3
62.30
14.47
13C-NMR (CDCl3)
f1 (ppm)

^{13}C-^{19}F couplings in ^{13}C-NMR. Only coupled peaks have been represented to make it easier to understand.

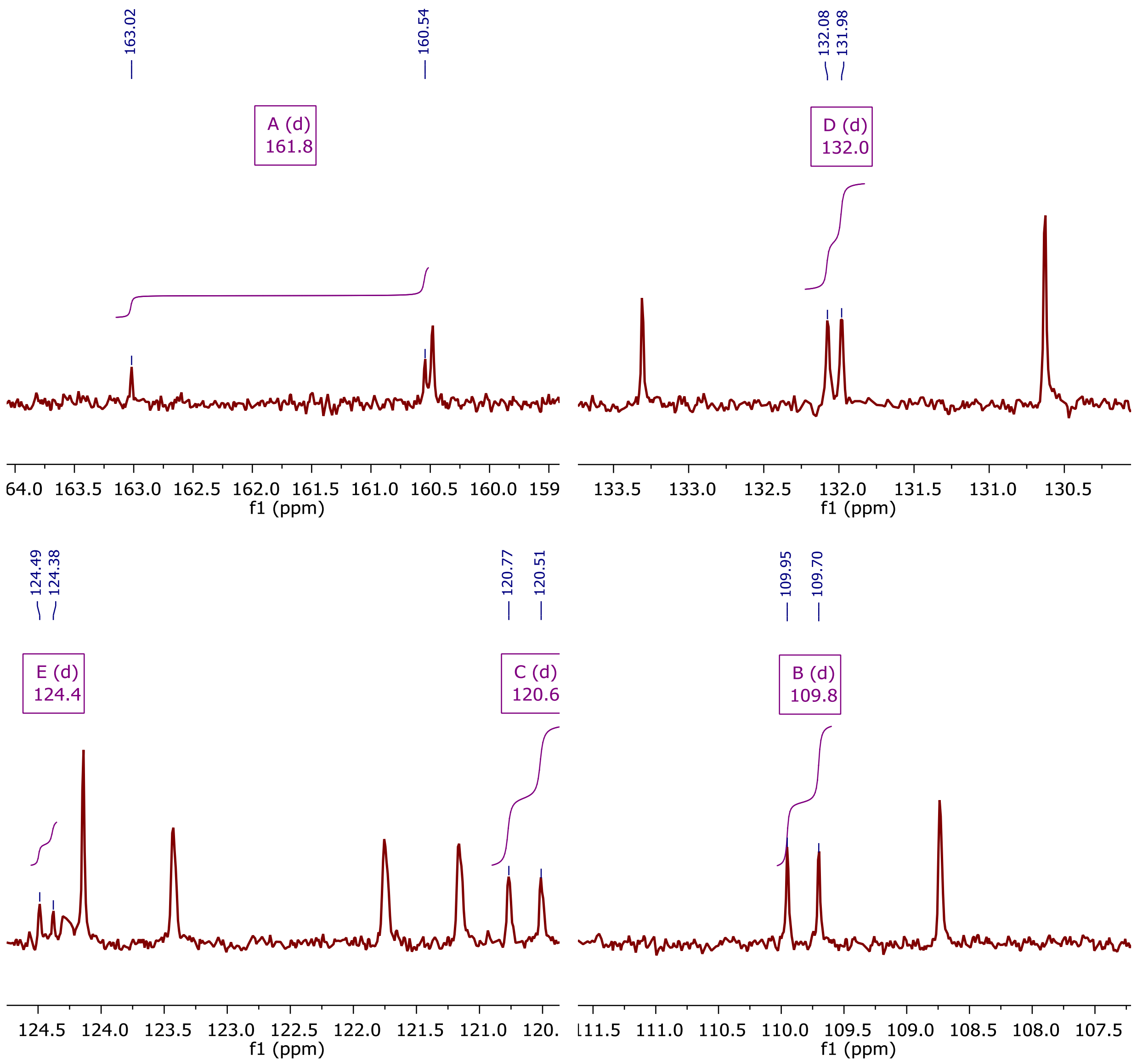

EI MS (70eV)

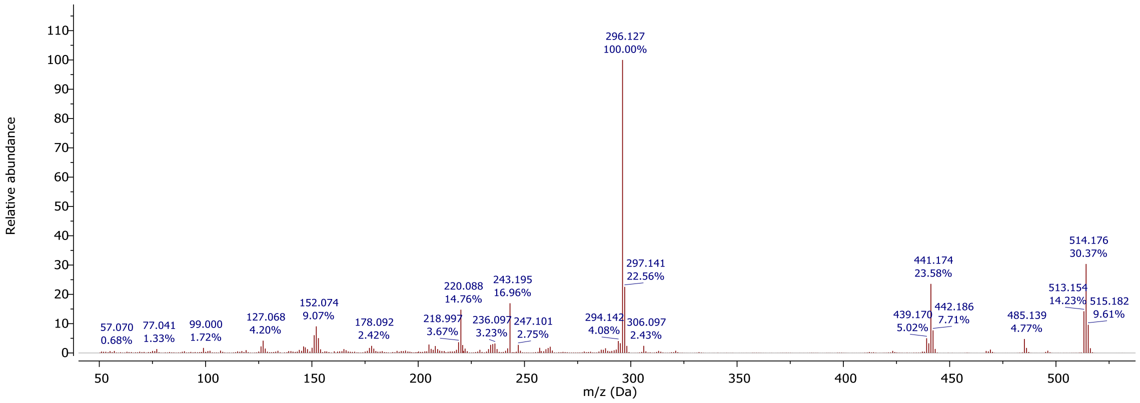

ESI-QTOF (positive ionization)

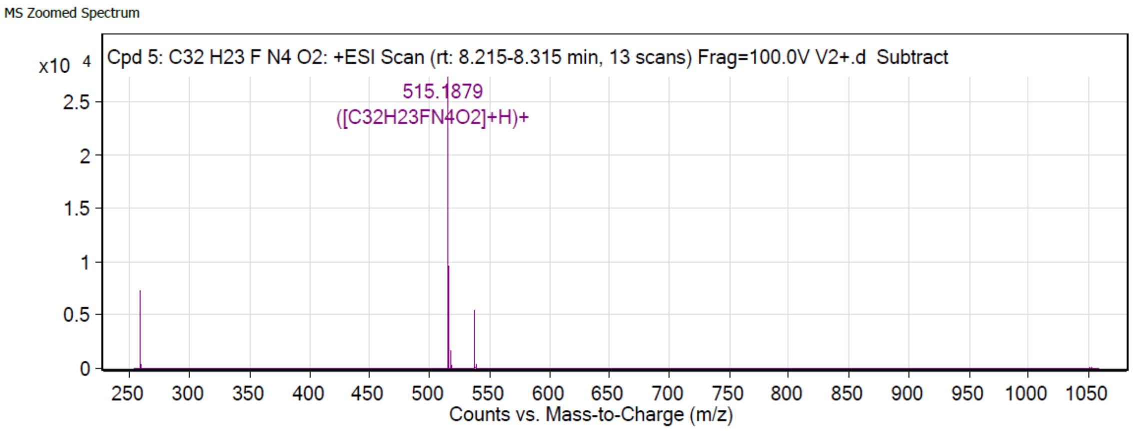

MS Spectrum Peak List

m/z	Calc *m/z*	Diff(ppm)	z	Abund	Formula	Ion
258.0976	258.0975	-0.47	2	7289.61	C32H23FN4O2	(M+2H)+2
258.5991	258.5991	0.05	2	2508.09	C32H23FN4O2	(M+2H)+2
259.1002	259.1006	1.61	2	459.76	C32H23FN4O2	(M+2H)+2
515.1879	515.1878	-0.32	1	27761.03	C32H23FN4O2	(M+H)+
516.1914	516.1909	-1.02	1	9549.47	C32H23FN4O2	(M+H)+
517.1939	517.1939	0.07	1	1710.24	C32H23FN4O2	(M+H)+
537.1699	537.1697	-0.34	1	5640.11	C32H23FN4O2	(M+Na)+
538.1734	538.1728	-1.07	1	1936.79	C32H23FN4O2	(M+Na)+
539.1759	539.1758	-0.14	1	461.44	C32H23FN4O2	(M+Na)+
1051.3494	1051.3502	0.76	1	218.16	C32H23FN4O2	(2M+Na)+

IR

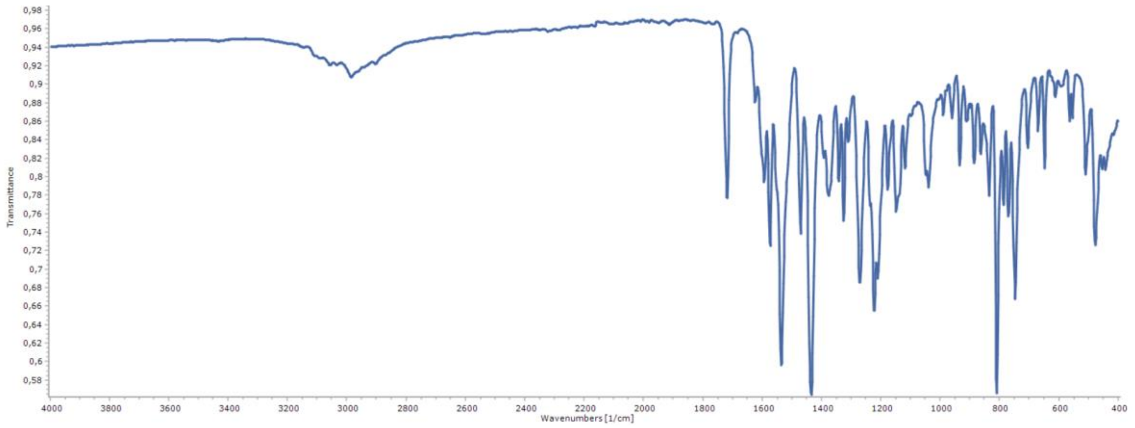

Compound **16c**

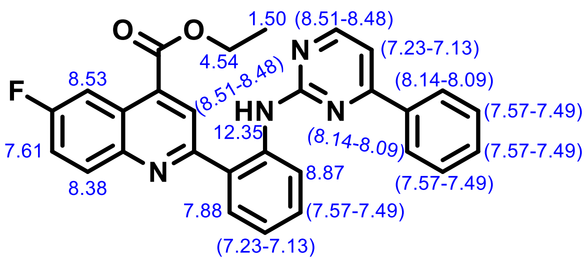

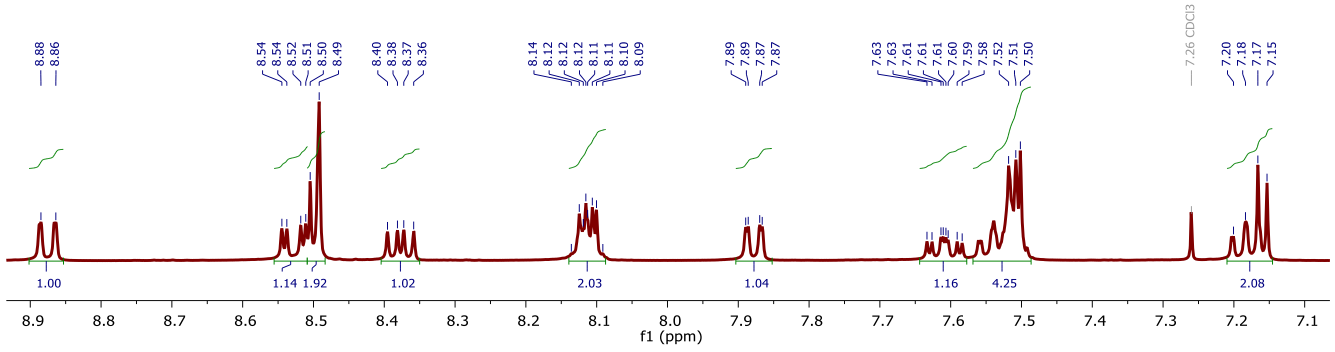

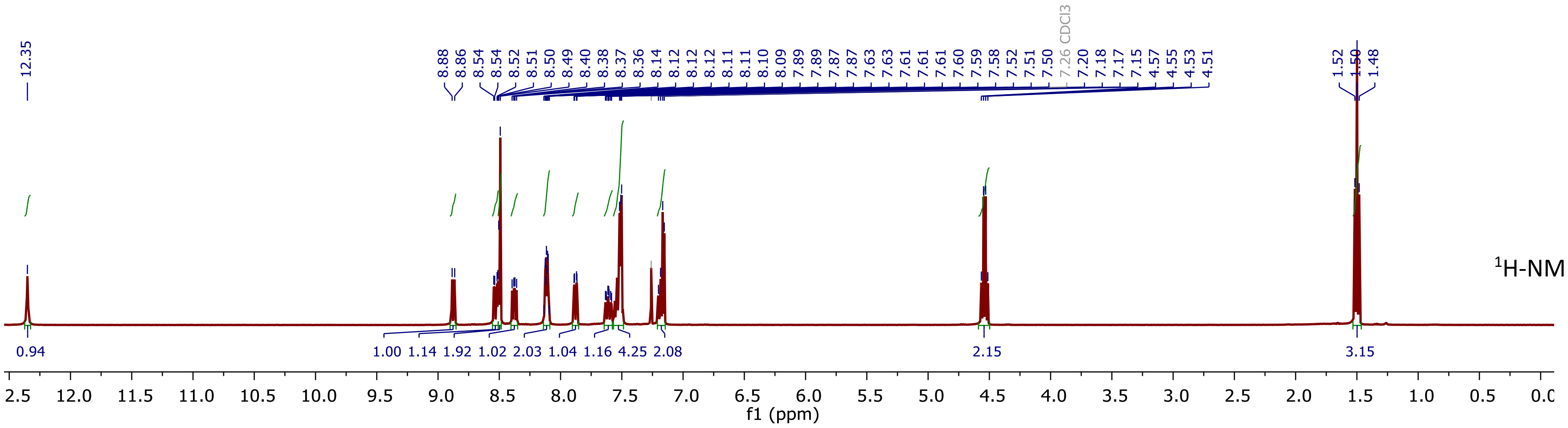

^{1}H-NMR ($CDCl_3$)

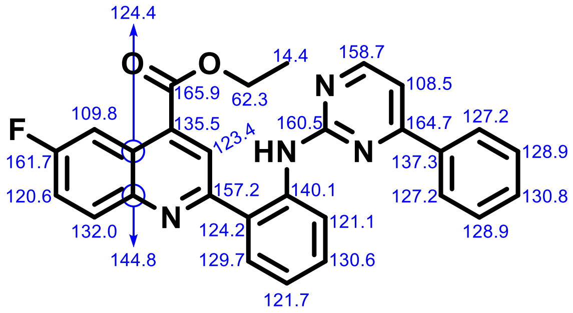
F
O
O
N
HN
N
N
124.4
14.4
158.7
165.9
62.3
108.5
109.8
135.5
123.4
160.5
164.7
127.2
128.9
161.7
137.3
120.6
157.2
140.1
127.2
130.8
132.0
124.2
121.1
128.9
144.8
129.7
130.6
121.7

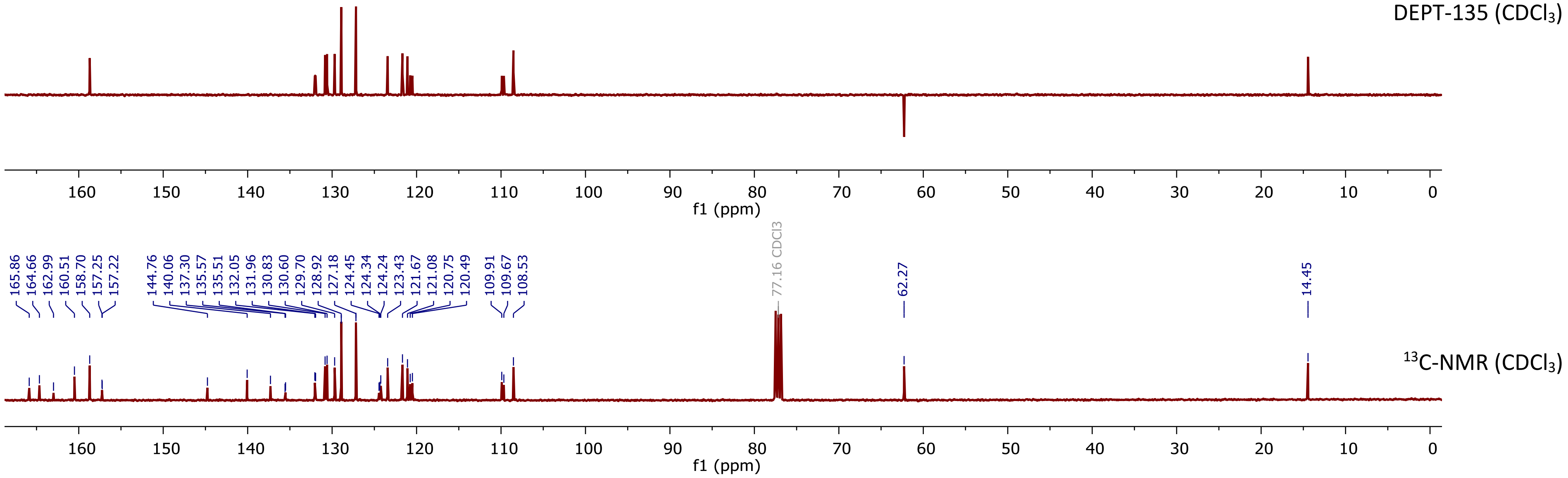
DEPT-135 (CDCl3)
f1 (ppm)
165.86
164.66
162.99
160.51
158.70
157.25
157.22
144.76
140.06
137.30
135.57
135.51
132.05
131.96
130.83
130.60
129.70
128.92
127.18
124.45
124.34
124.24
123.43
121.67
121.08
120.75
120.49
109.91
109.67
108.53
77.16 CDCl3
62.27
14.45
13C-NMR (CDCl3)
f1 (ppm)

^{13}C-^{19}F couplings in ^{13}C-NMR. Only coupled peaks have been represented to make it easier to understand.

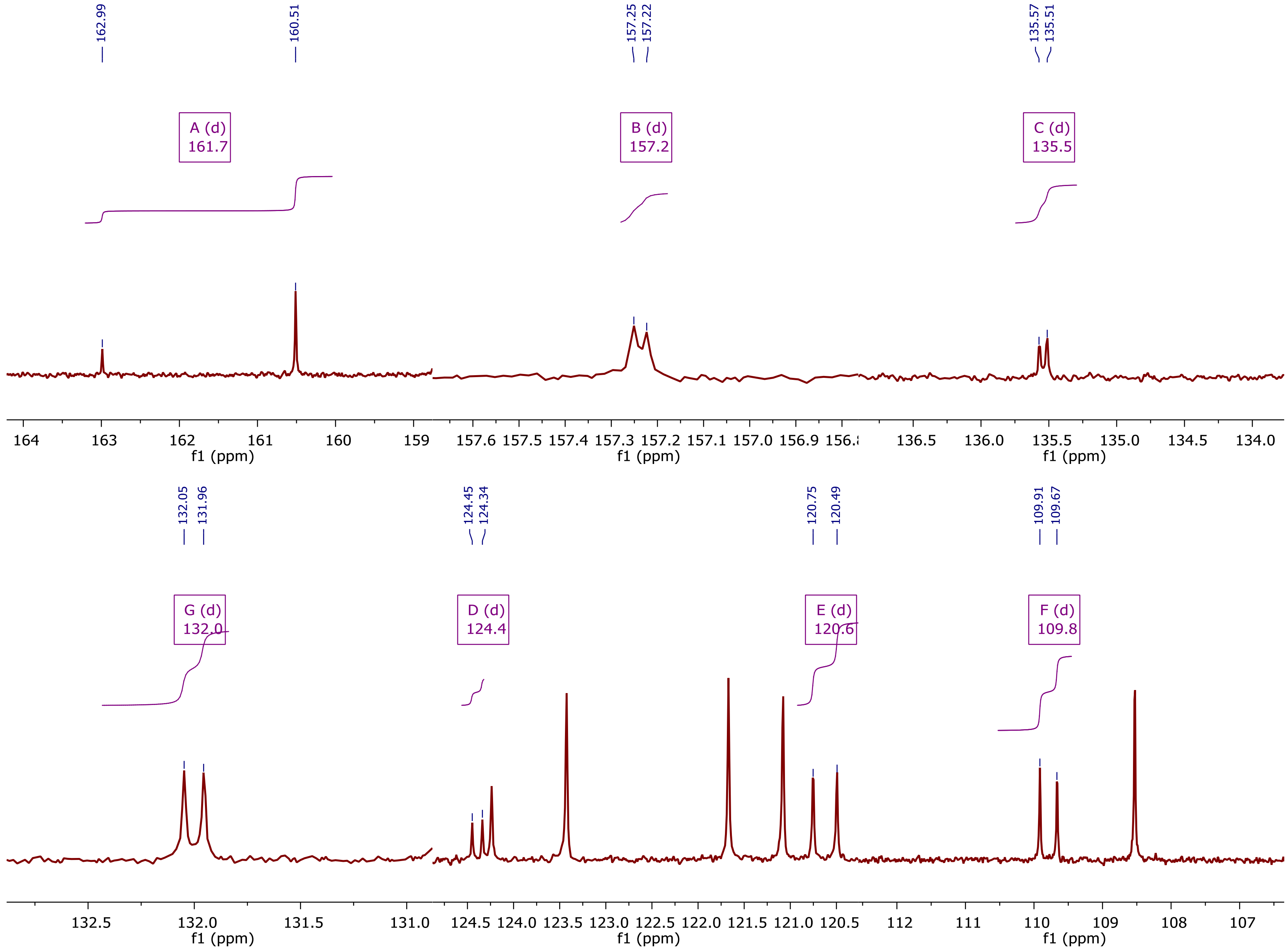

EI MS (70eV)

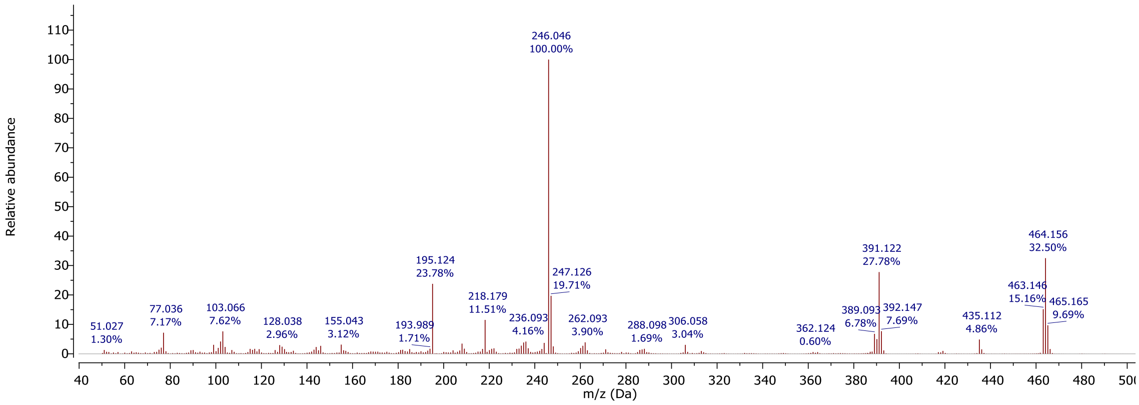

ESI-QTOF (positive ionization)

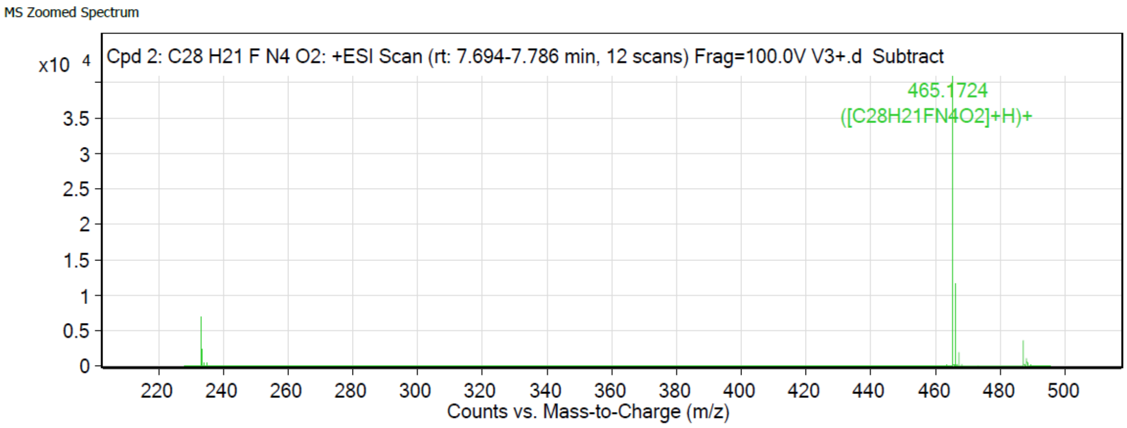

MS Spectrum Peak List

m/z	Calc *m/z*	Diff(ppm)	z	Abund	Formula	Ion
233.0897	233.0897	-0.02	2	6990.25	C28H21FN4O2	(M+2H)+2
233.5913	233.5913	-0.41	2	2270.24	C28H21FN4O2	(M+2H)+2
234.093	234.0927	-1.37	2	455.6	C28H21FN4O2	(M+2H)+2
465.1724	465.1721	-0.51	1	41167.47	C28H21FN4O2	(M+H)+
466.1756	466.1752	-0.75	1	11882.28	C28H21FN4O2	(M+H)+
467.1786	467.1782	-0.87	1	1978.88	C28H21FN4O2	(M+H)+
468.1807	468.181	0.5	1	270.14	C28H21FN4O2	(M+H)+
487.1541	487.1541	-0.01	1	3637.43	C28H21FN4O2	(M+Na)+
488.1566	488.1572	1.2	1	1146.81	C28H21FN4O2	(M+Na)+
489.1589	489.1601	2.51	1	183.59	C28H21FN4O2	(M+Na)+

IR

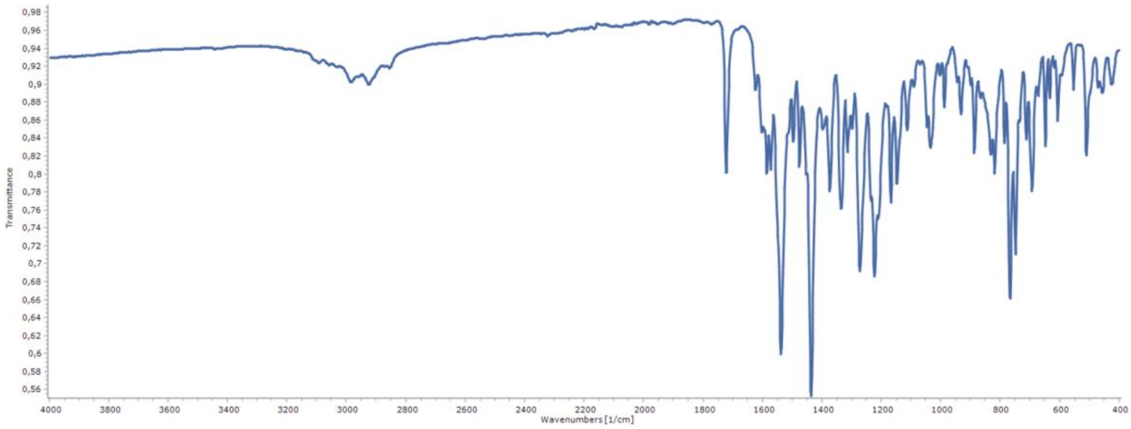

Compound **16d**

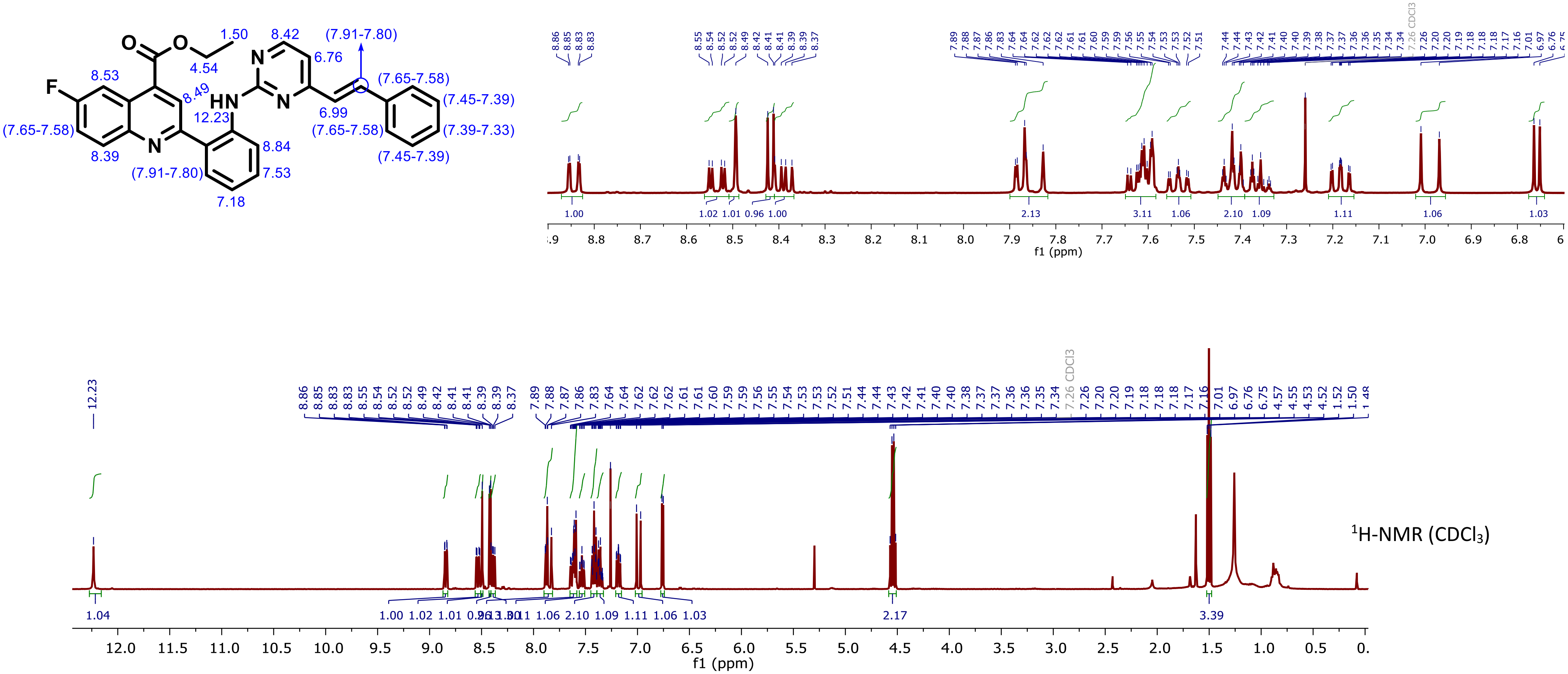

^{1}H-NMR ($CDCl_3$)

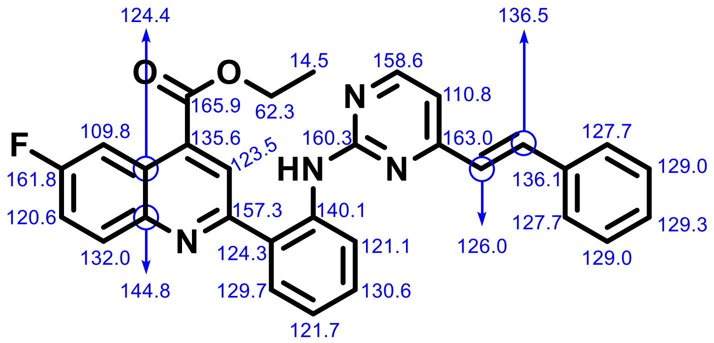

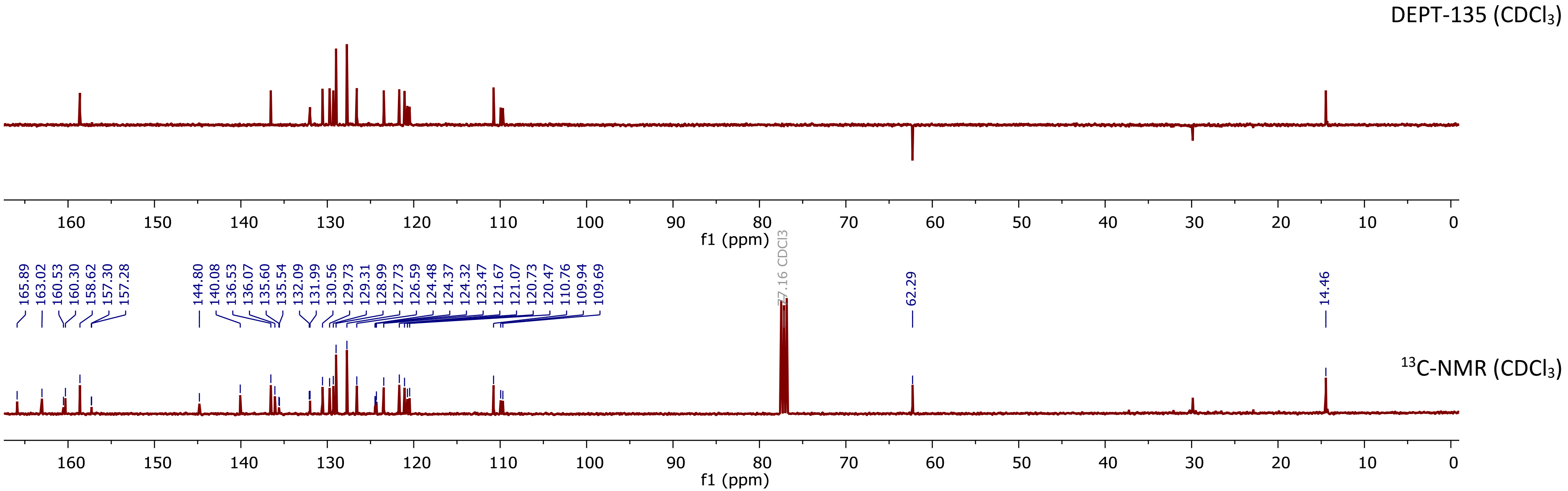
DEPT-135 (CDCl3)
13C-NMR (CDCl3)
f1 (ppm)
165.89
163.02
160.53
160.30
158.62
157.30
157.28
144.80
140.08
136.53
136.07
135.60
135.54
132.09
131.99
130.56
129.73
129.31
128.99
127.73
126.59
124.48
124.37
124.32
123.47
121.67
121.07
120.73
120.47
110.76
109.94
109.69
77.16 CDCl3
62.29
14.46

^{13}C-^{19}F couplings in ^{13}C-NMR. Only coupled peaks have been represented to make it easier to understand.

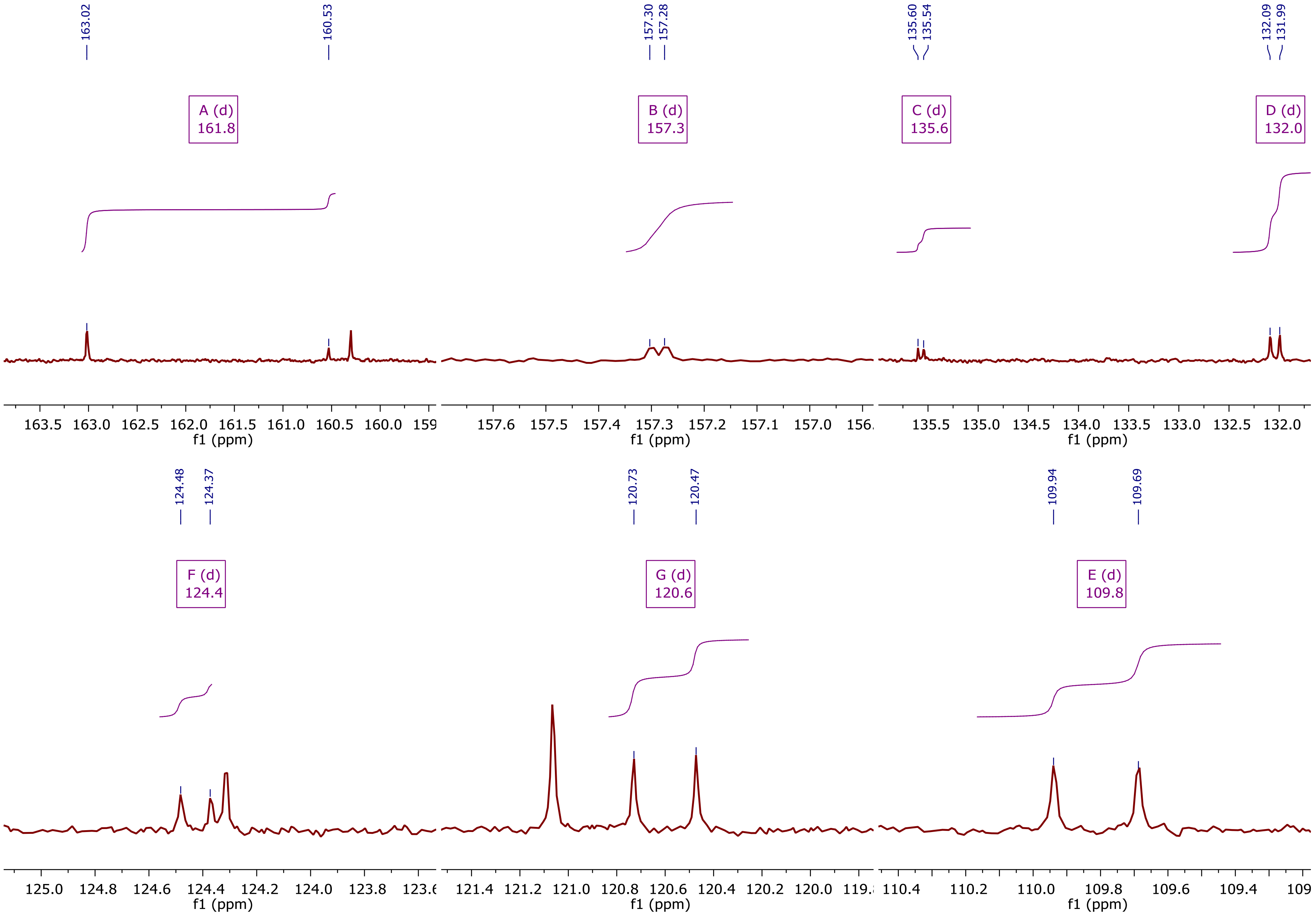

EI MS (70eV)

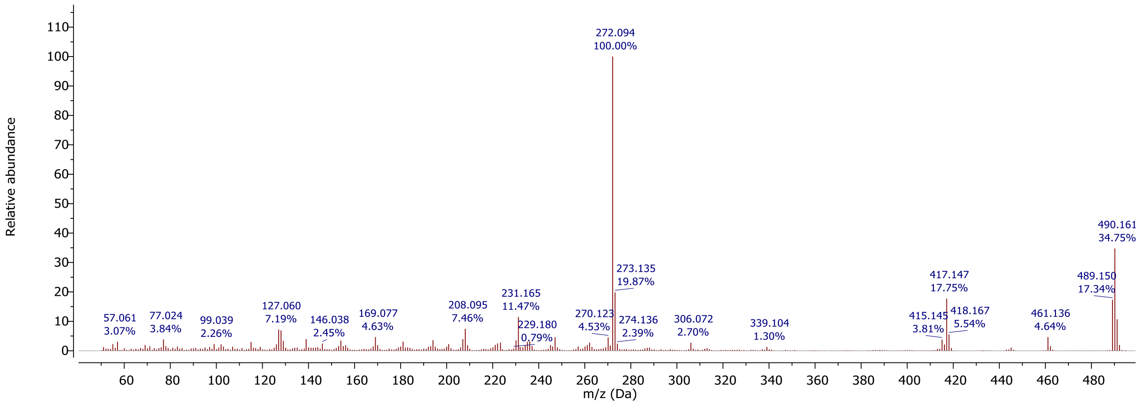

ESI-QTOF (positive ionization)

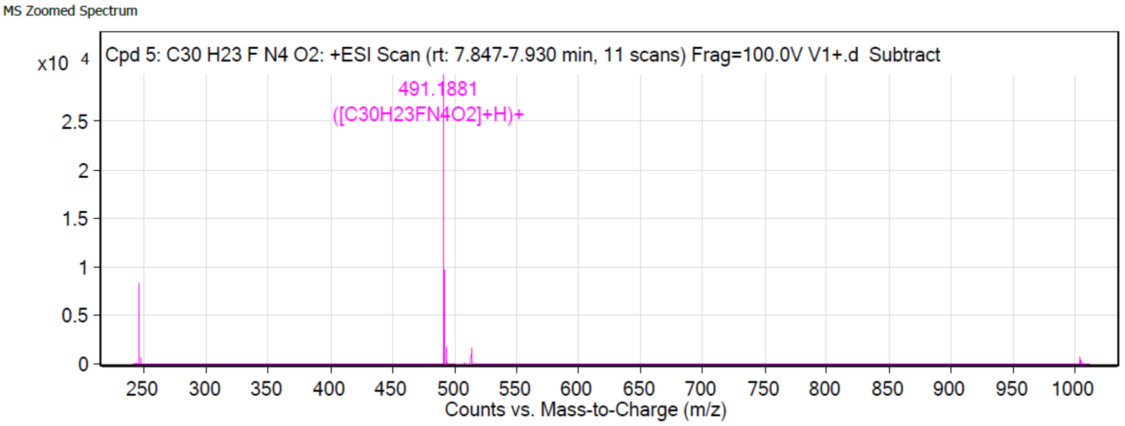

MS Spectrum Peak List

m/z	Calc *m/z*	Diff(ppm)	z	Abund	Formula	Ion
246.0977	246.0975	-0.76	2	8294.67	C30H23FN4O2	(M+2H)+2
246.5989	246.5991	0.83	2	2792.43	C30H23FN4O2	(M+2H)+2
247.1008	247.1006	-1.07	2	568.16	C30H23FN4O2	(M+2H)+2
491.1881	491.1878	-0.67	1	30014.32	C30H23FN4O2	(M+H)+
492.1913	492.1909	-0.8	1	9804.74	C30H23FN4O2	(M+H)+
493.1942	493.1939	-0.6	1	1775.04	C30H23FN4O2	(M+H)+
513.1707	513.1697	-1.83	1	1687.41	C30H23FN4O2	(M+Na)+
514.1715	514.1728	2.51	1	632.44	C30H23FN4O2	(M+Na)+
1003.3504	1003.3502	-0.14	1	802.72	C30H23FN4O2	(2M+Na)+
1004.3531	1004.3533	0.27	1	522.24	C30H23FN4O2	(2M+Na)+

IR

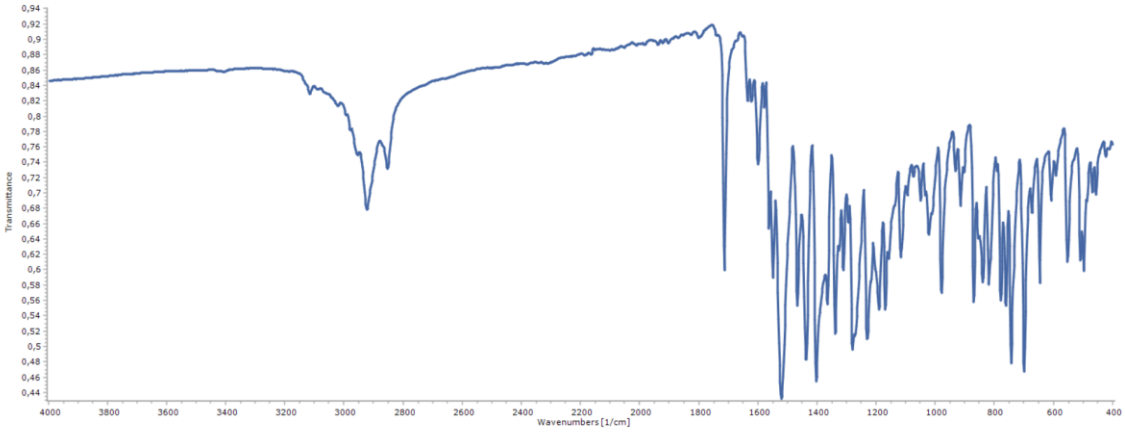

Compound **17a**

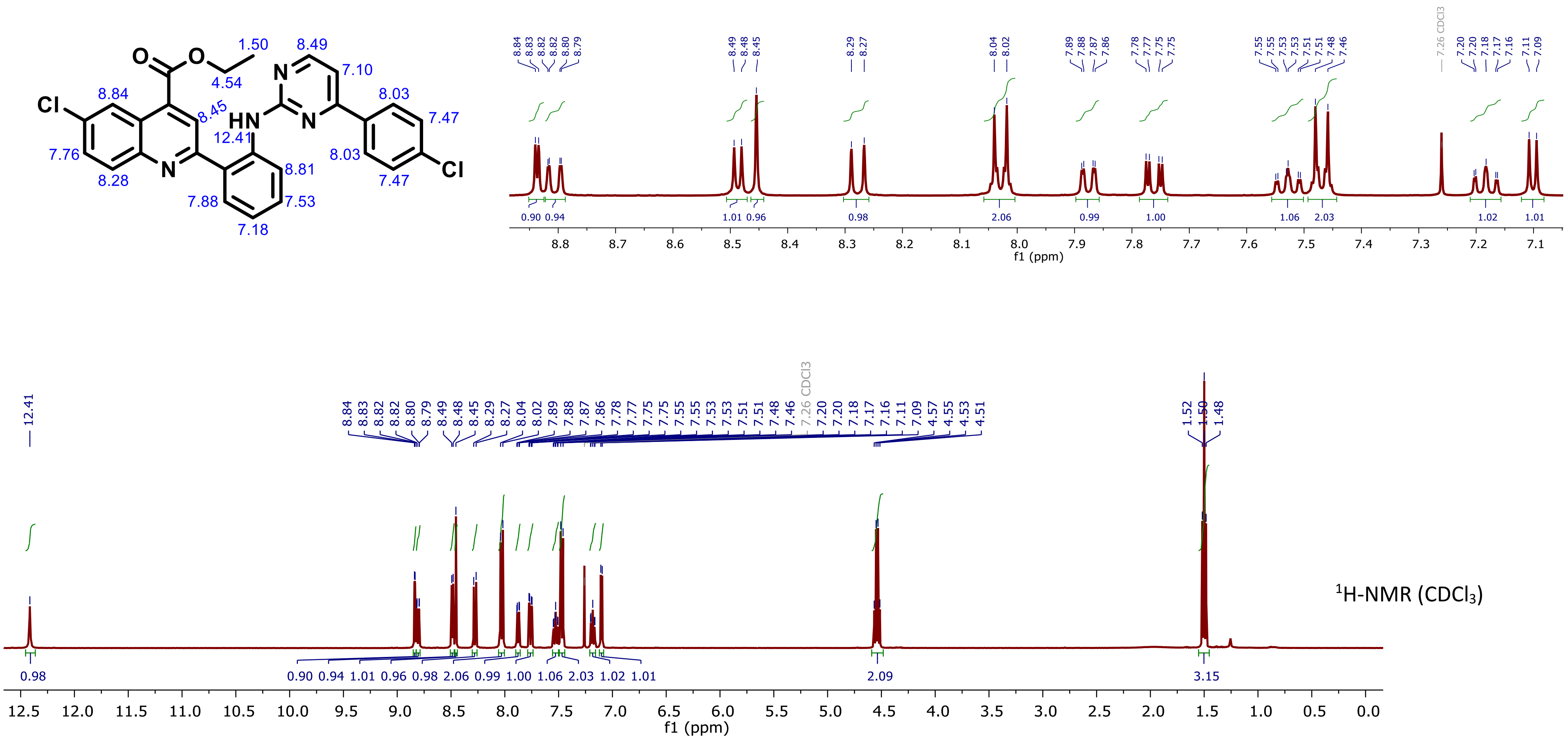

^{1}H-NMR ($CDCl_3$)

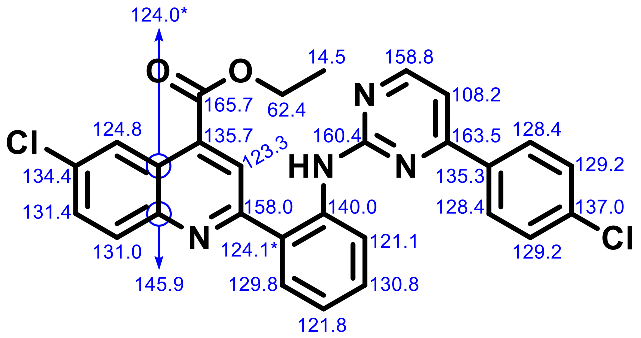

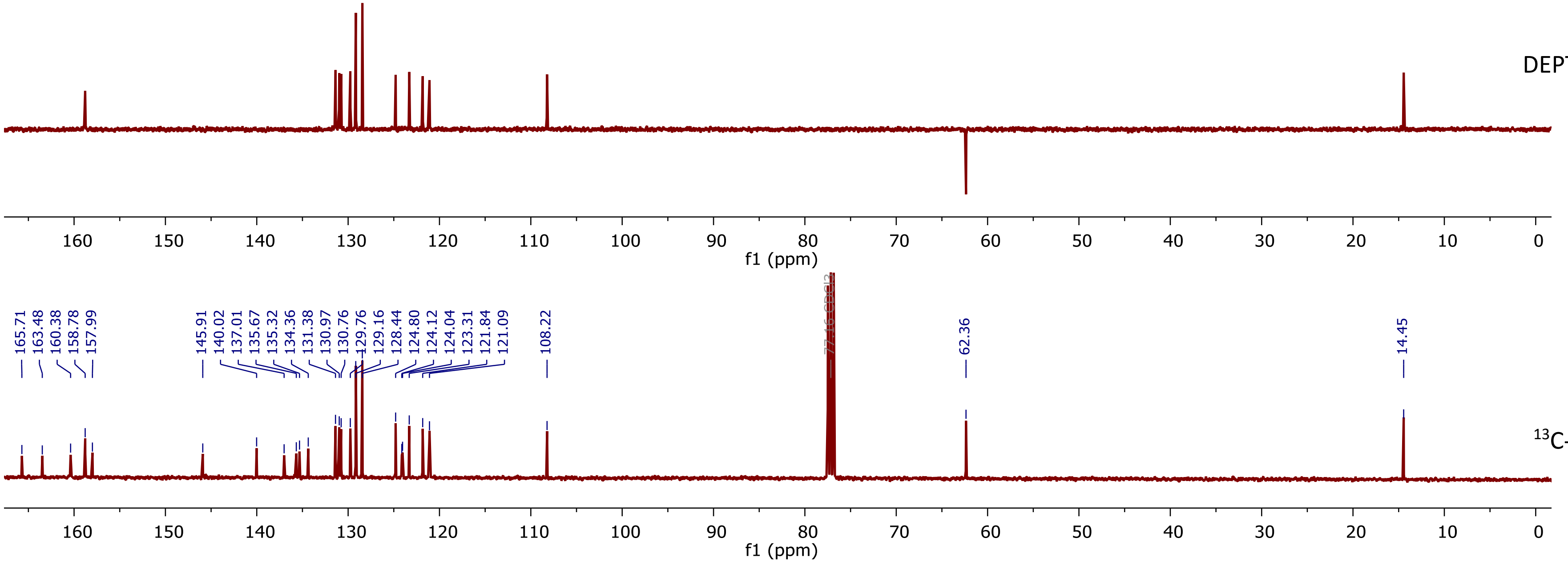

DEPT-135 ($CDCl_3$)

^{13}C-NMR ($CDCl_3$)

EI MS (70eV)

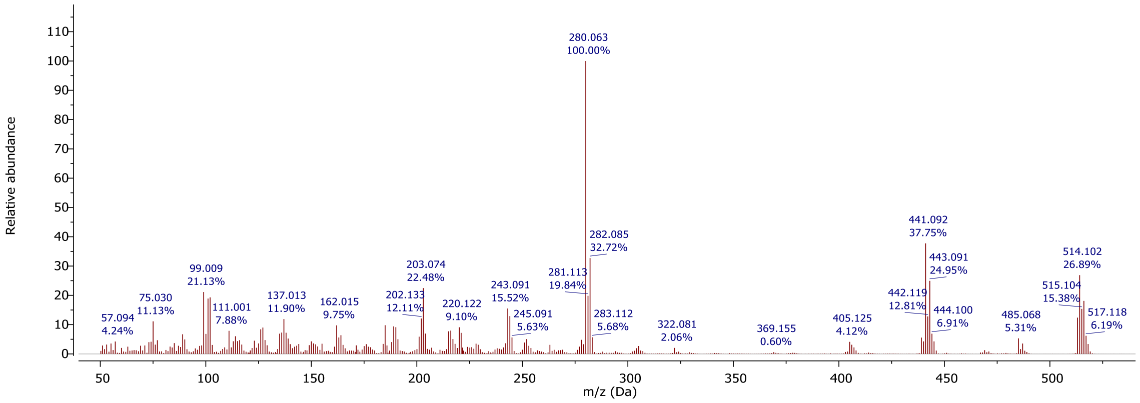

ESI-QTOF (positive ionization)

MS Zoomed Spectrum

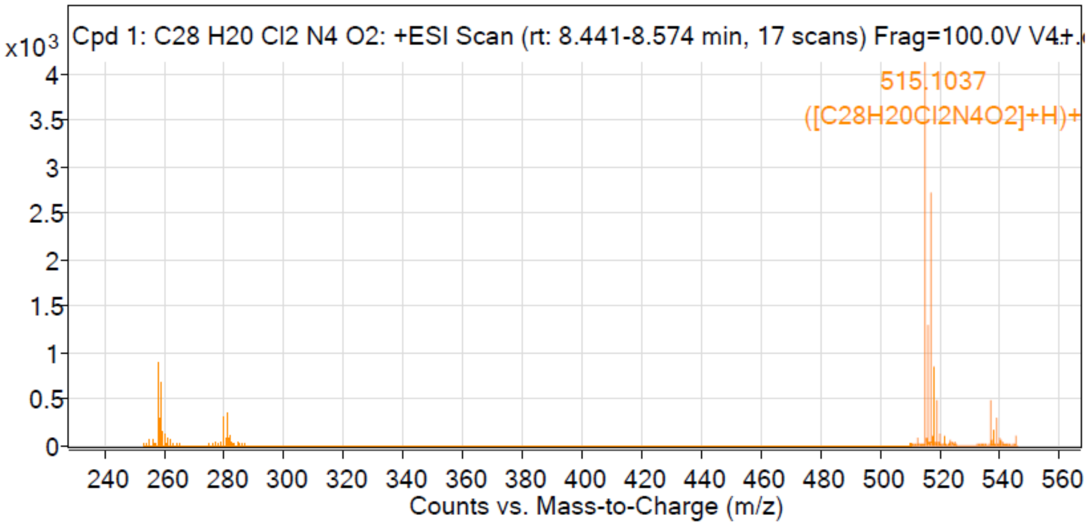

MS Spectrum Peak List

m/z	Calc *m/z*	Diff(ppm)	z	Abund	Formula	Ion
258.0563	258.0554	-3.31	2	911.94	C28H20Cl2N4O2	(M+2H)+2
259.0539	259.0543	1.65	2	676.93	C28H20Cl2N4O2	(M+2H)+2
280.0298	280.0374	26.99	2	313.4	C28H20Cl2N4O2	(M+2Na)+2
281.0292	281.0363	24.98	2	366.71	C28H20Cl2N4O2	(M+2Na)+2
515.1037	515.1036	-0.08	1	4132.52	C28H20Cl2N4O2	(M+H)+
516.1071	516.1067	-0.86	1	1302.22	C28H20Cl2N4O2	(M+H)+
517.101	517.1014	0.72	1	2715.84	C28H20Cl2N4O2	(M+H)+
518.1046	518.104	-1.05	1	856.73	C28H20Cl2N4O2	(M+H)+
519.1002	519.1	-0.27	1	482.72	C28H20Cl2N4O2	(M+H)+
537.0854	537.0856	0.35	1	493.46	C28H20Cl2N4O2	(M+Na)+

IR

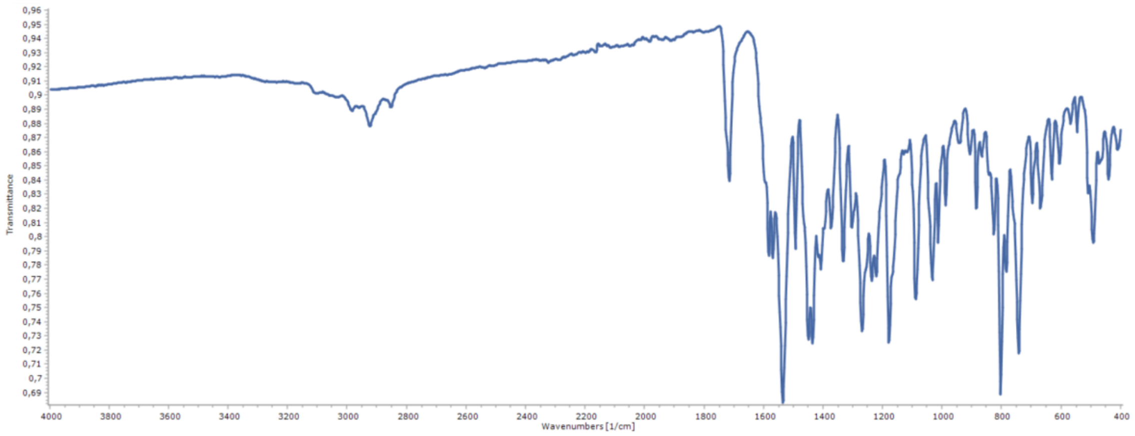

Compound **17b**

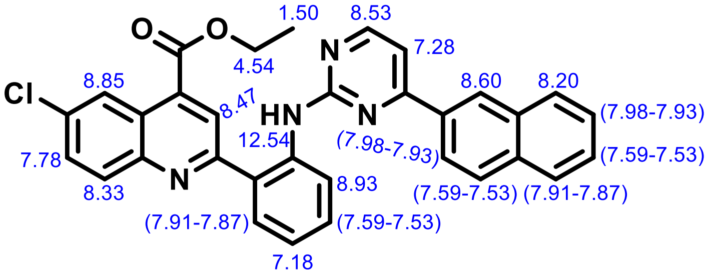

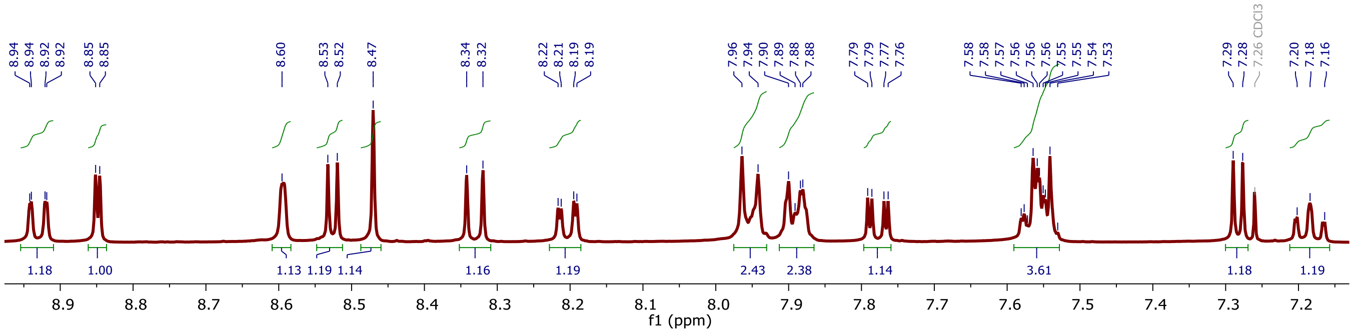

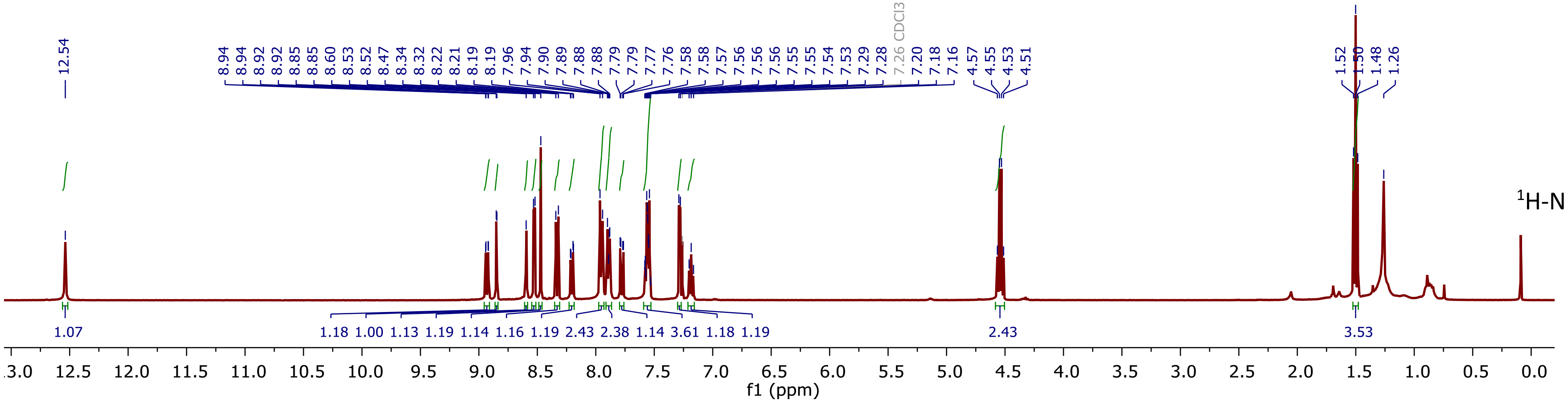

^{1}H-NMR ($CDCl_3$)

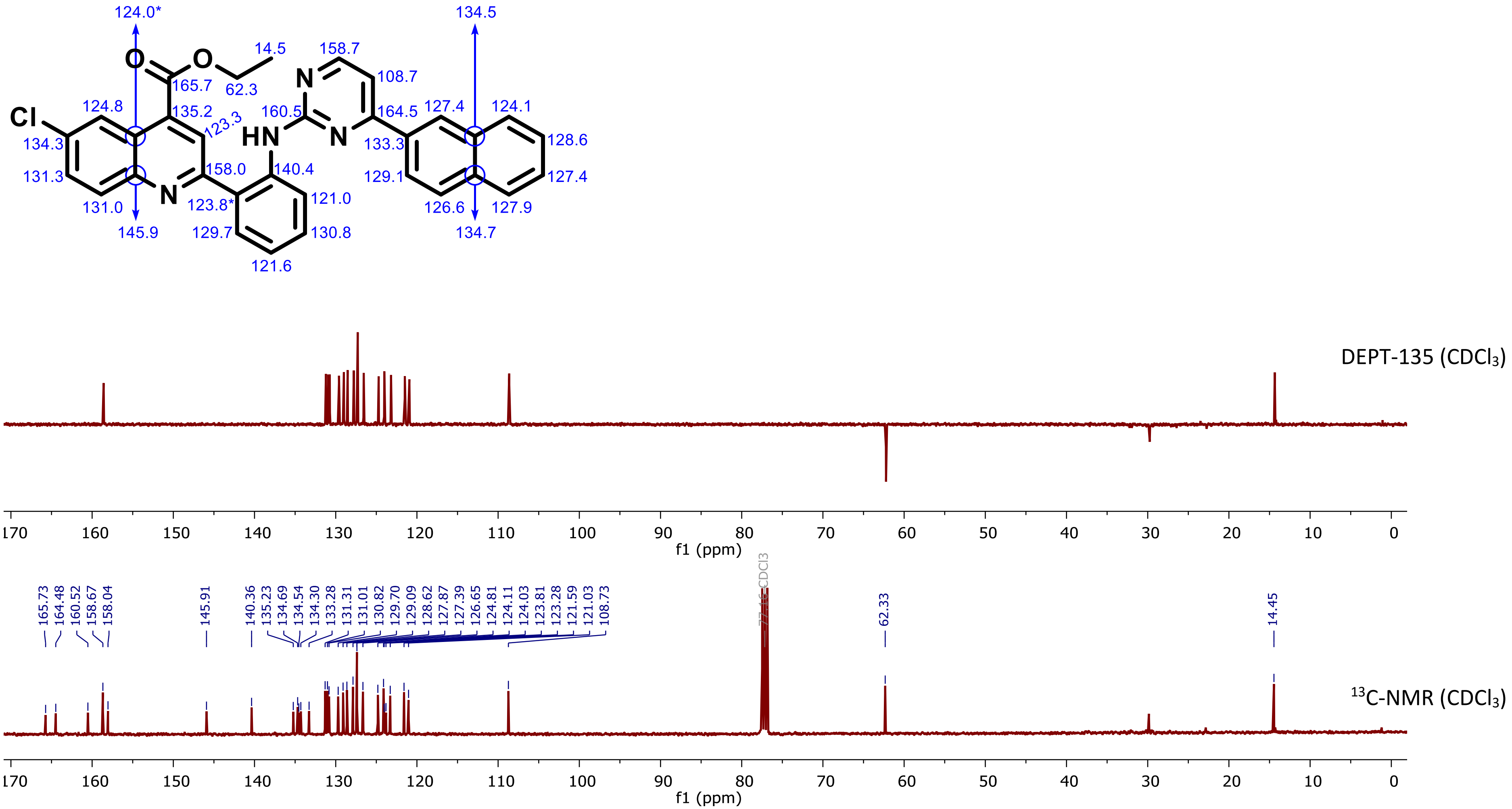

124.0*
134.5
14.5
158.7
165.7
62.3
108.7
124.8
135.2
123.3
160.5
164.5
127.4
124.1
Cl
134.3
HN
133.3
128.6
131.3
158.0
140.4
129.1
127.4
131.0
N
123.8*
121.0
126.6
127.9
145.9
129.7
130.8
134.7
121.6
DEPT-135 (CDCl3)
170 160 150 140 130 120 110 100 90 80 70 60 50 40 30 20 10 0
f1 (ppm)
165.73 164.48 160.52 158.67 158.04 145.91 140.36 135.23 134.69 134.54 134.30 133.28 131.31 131.01 130.82 129.70 129.09 128.62 127.87 127.39 126.65 124.81 124.11 124.03 123.81 123.28 121.59 121.03 108.73
CDCl3
62.33
14.45
13C-NMR (CDCl3)
170 160 150 140 130 120 110 100 90 80 70 60 50 40 30 20 10 0
f1 (ppm)

EI MS (70eV)

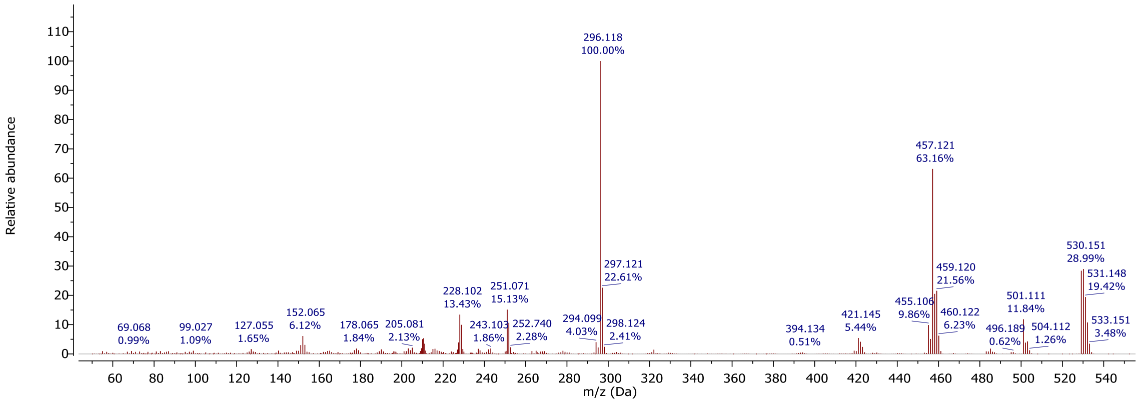

ESI-QTOF (positive ionization)

MS Zoomed Spectrum

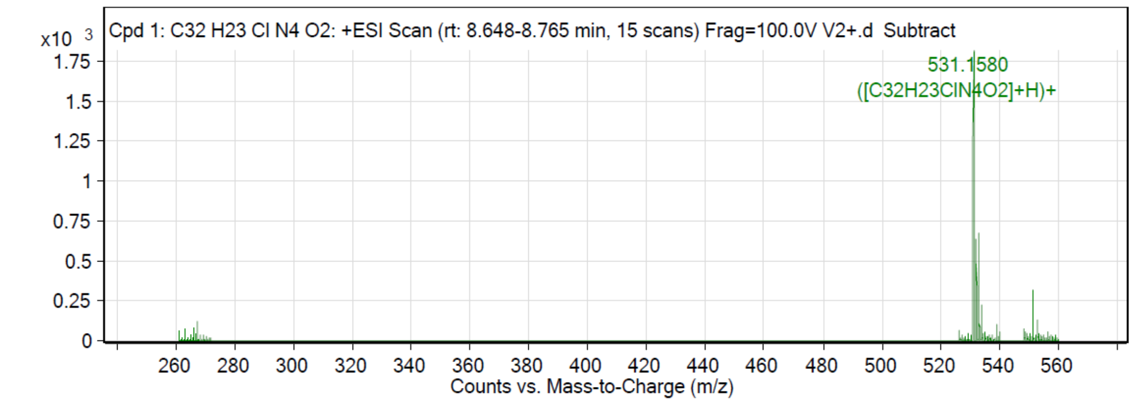

MS Spectrum Peak List

m/z	Calc *m/z*	Diff(ppm)	z	Abund	Formula	Ion
266.0844	266.0828	-6.08	2	83.09	C32H23ClN4O2	(M+2H)+2
266.5848	266.5843	-1.63	2	49.47	C32H23ClN4O2	(M+2H)+2
531.158	531.1582	0.49	1	1876.26	C32H23ClN4O2	(M+H)+
532.1624	532.1614	-1.92	1	648.25	C32H23ClN4O2	(M+H)+
533.1573	533.1569	-0.83	1	675.78	C32H23ClN4O2	(M+H)+
534.158	534.159	2.02	1	228.35	C32H23ClN4O2	(M+H)+
535.1643	535.1617	-4.83	1	53.32	C32H23ClN4O2	(M+H)+
553.1393	553.1402	1.65	1	133.97	C32H23ClN4O2	(M+Na)+
554.1487	554.1433	-9.71	1	35.44	C32H23ClN4O2	(M+Na)+
555.1365	555.1388	4.12	1	41.97	C32H23ClN4O2	(M+Na)+

IR

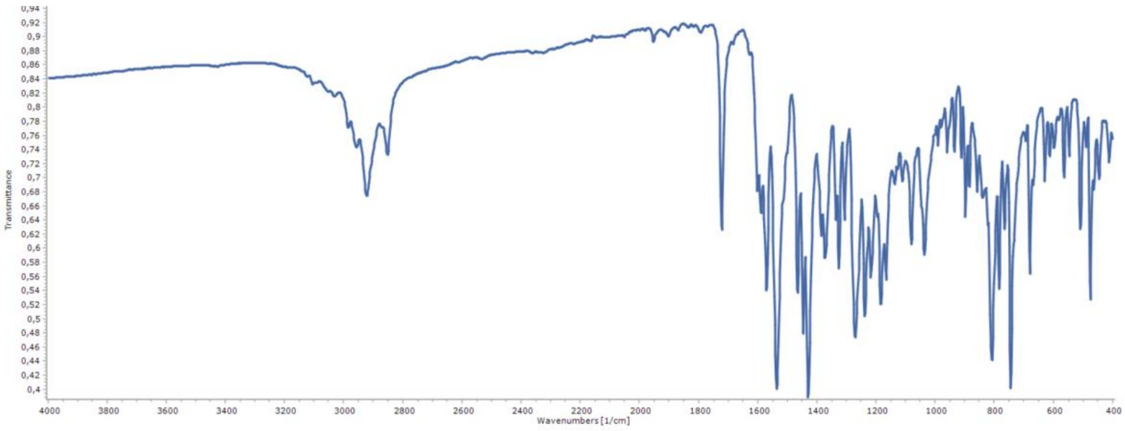

Compound **17c**

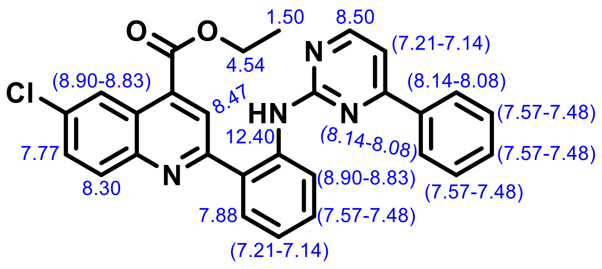

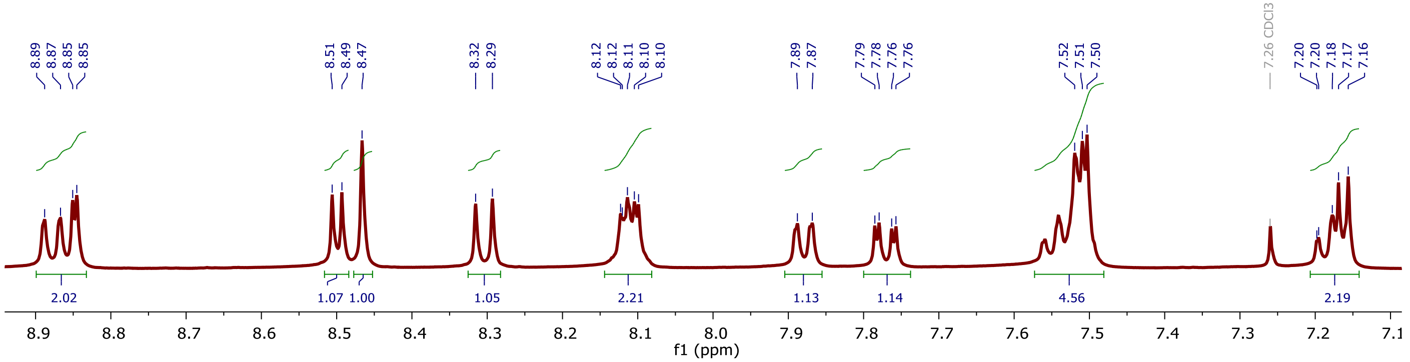

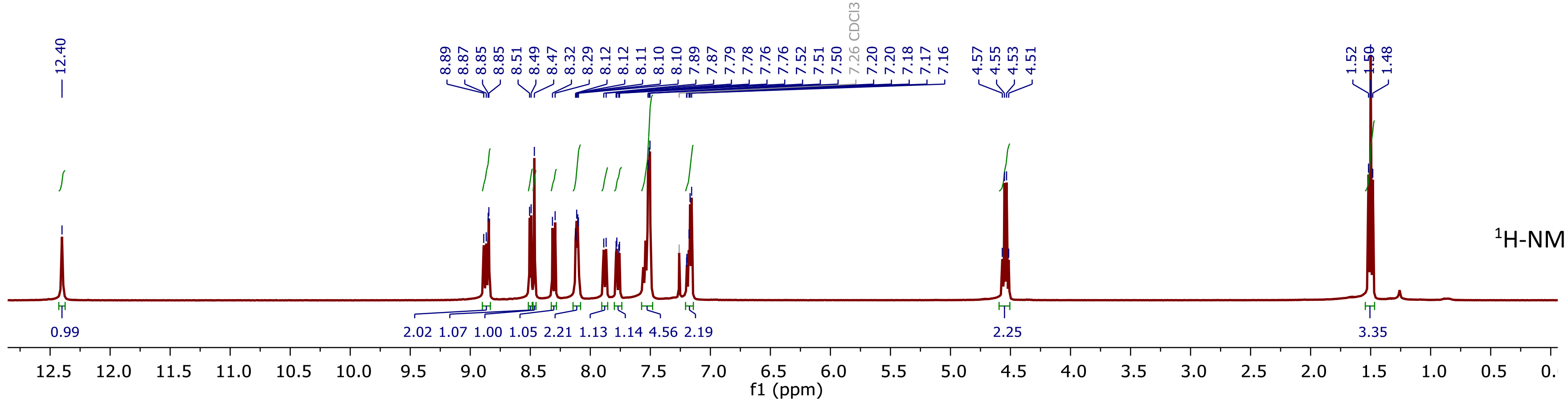

^{1}H-NMR ($CDCl_3$)

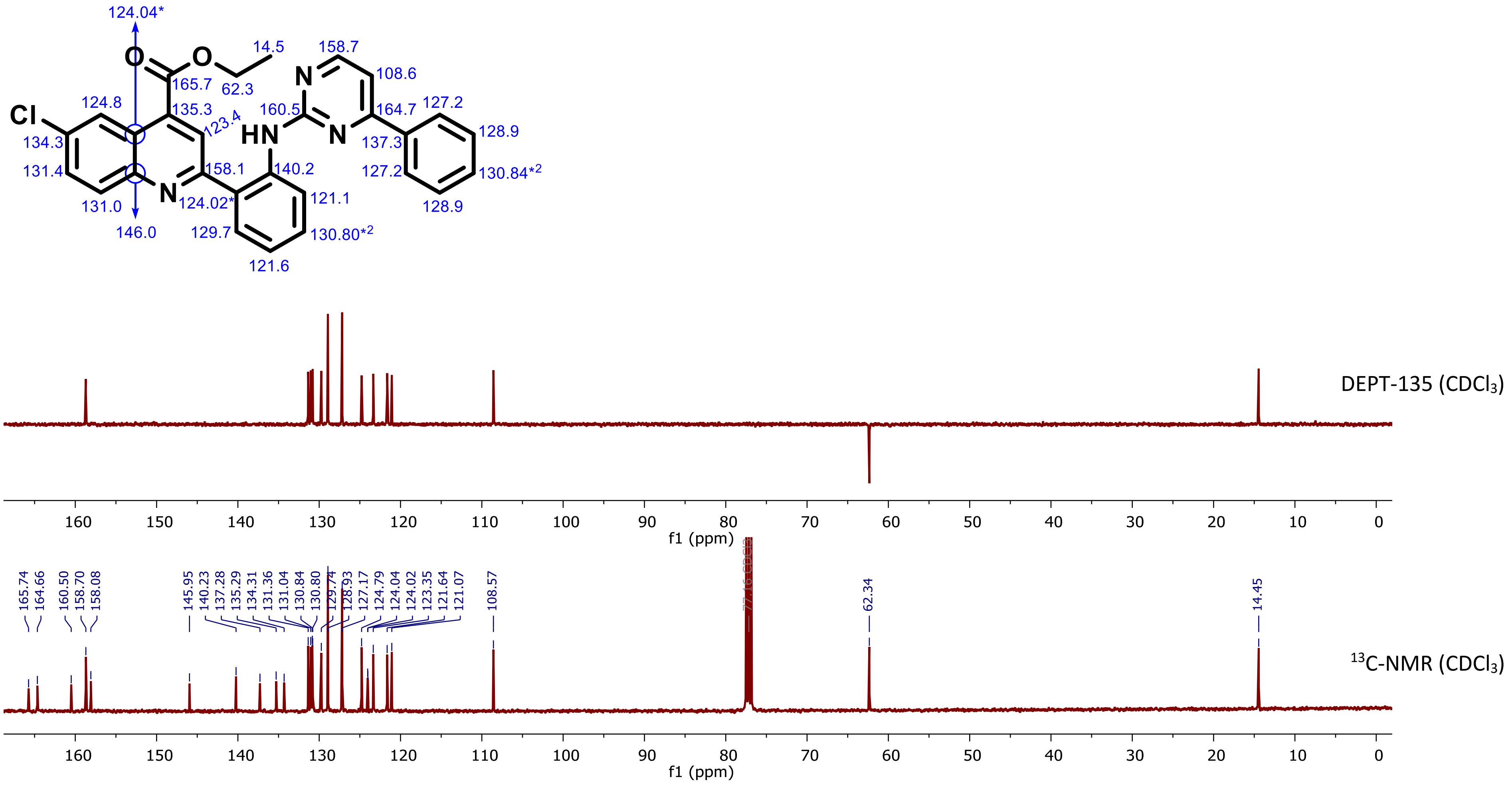
124.04*
14.5
158.7
165.7
62.3
108.6
Cl
124.8
135.3
123.4
160.5
164.7
127.2
HN
134.3
137.3
128.9
131.4
158.1
140.2
127.2
130.84*2
131.0
124.02*
121.1
128.9
146.0
129.7
130.80*2
121.6
DEPT-135 (CDCl3)
f1 (ppm)
165.74
164.66
160.50
158.70
158.08
145.95
140.23
137.28
135.29
134.31
131.36
131.04
130.84
130.80
129.74
128.93
127.17
124.79
124.04
124.02
123.35
121.64
121.07
108.57
62.34
14.45
13C-NMR (CDCl3)
f1 (ppm)

EI MS (70eV)

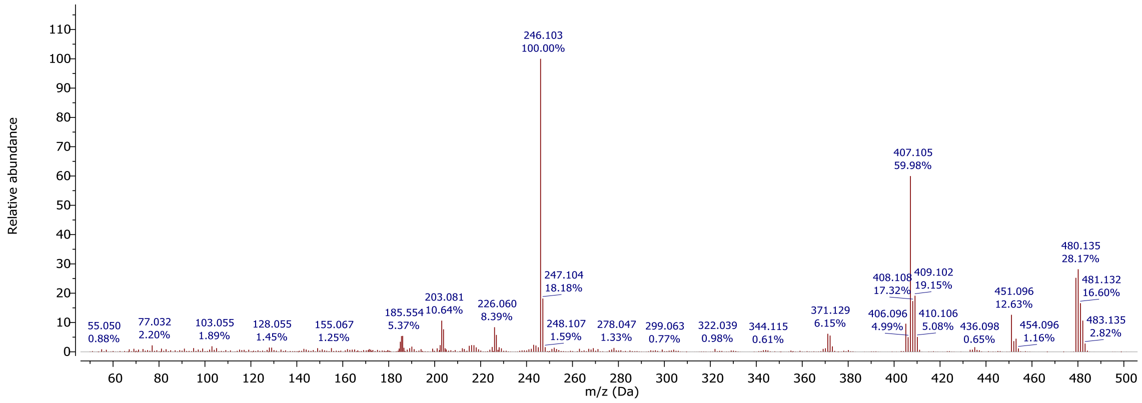

ESI-QTOF (positive ionization)

MS Zoomed Spectrum

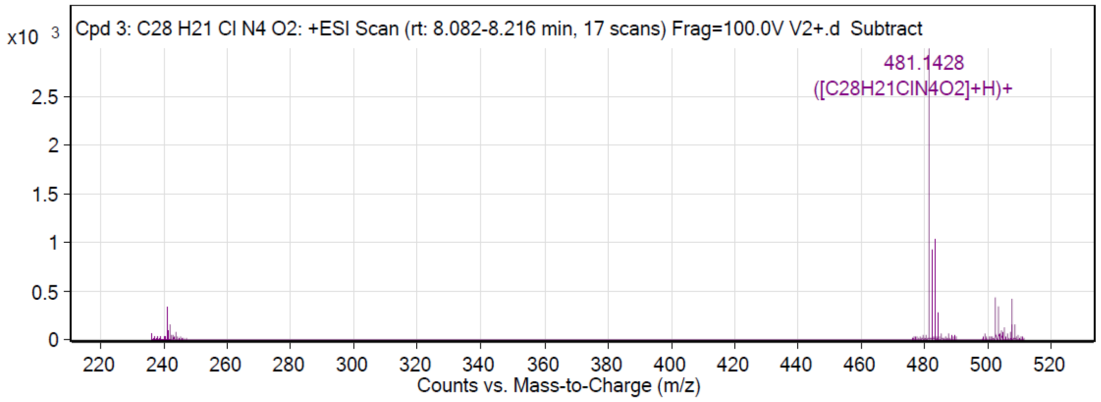

MS Spectrum Peak List

m/z	Calc *m/z*	Diff(ppm)	z	Abund	Formula	Ion
241.0747	241.0749	0.79	2	351.13	C28H21ClN4O2	(M+2H)+2
241.5762	241.5765	1.3	2	98.06	C28H21ClN4O2	(M+2H)+2
242.073	242.0741	4.54	2	167.99	C28H21ClN4O2	(M+2H)+2
481.1428	481.1426	-0.49	1	3019.39	C28H21ClN4O2	(M+H)+
482.1458	482.1457	-0.29	1	921.27	C28H21ClN4O2	(M+H)+
483.1411	483.1409	-0.29	1	1029.44	C28H21ClN4O2	(M+H)+
484.1433	484.1432	-0.07	1	278.04	C28H21ClN4O2	(M+H)+
503.1234	503.1245	2.3	1	340.26	C28H21ClN4O2	(M+Na)+
504.125	504.1276	5.1	1	87.89	C28H21ClN4O2	(M+Na)+
505.1216	505.1229	2.44	1	118.29	C28H21ClN4O2	(M+Na)+

IR

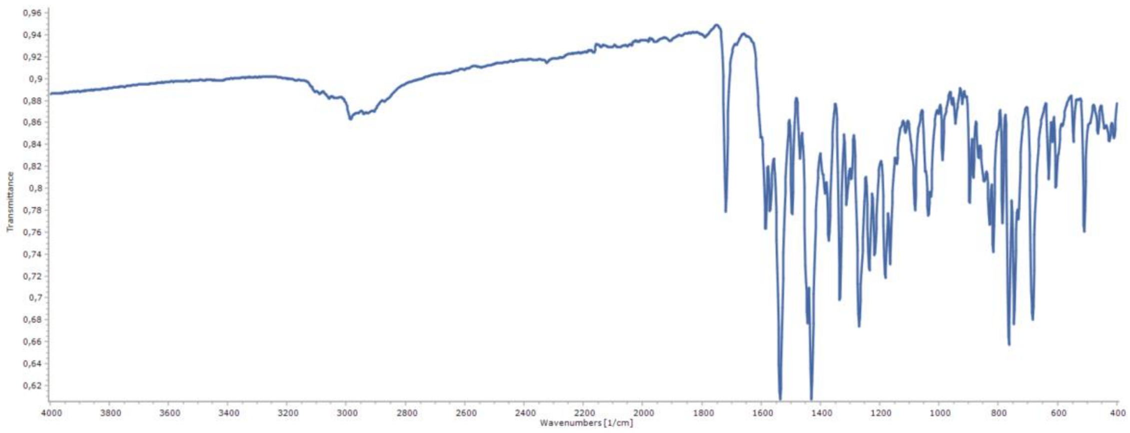

Compound **17d**

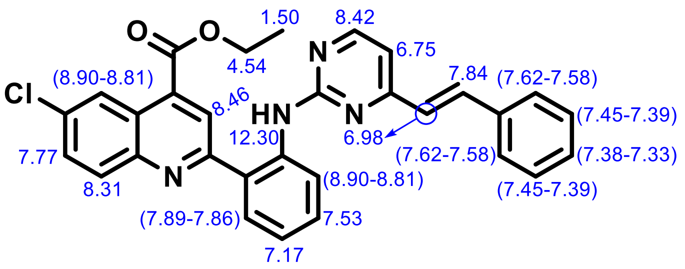

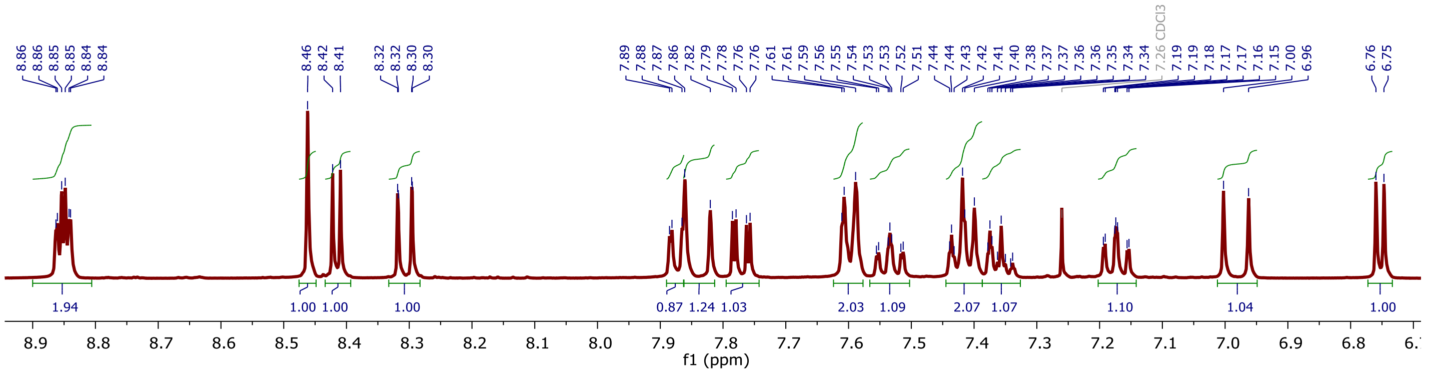

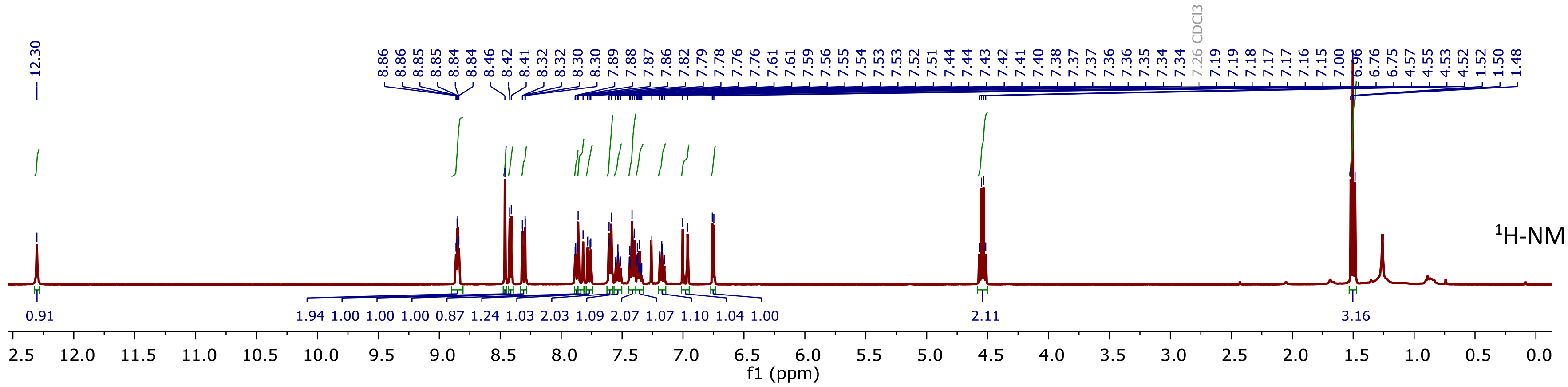

^{1}H-NMR ($CDCl_3$)

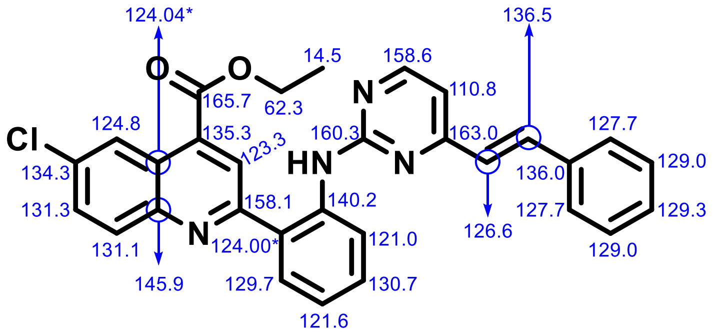

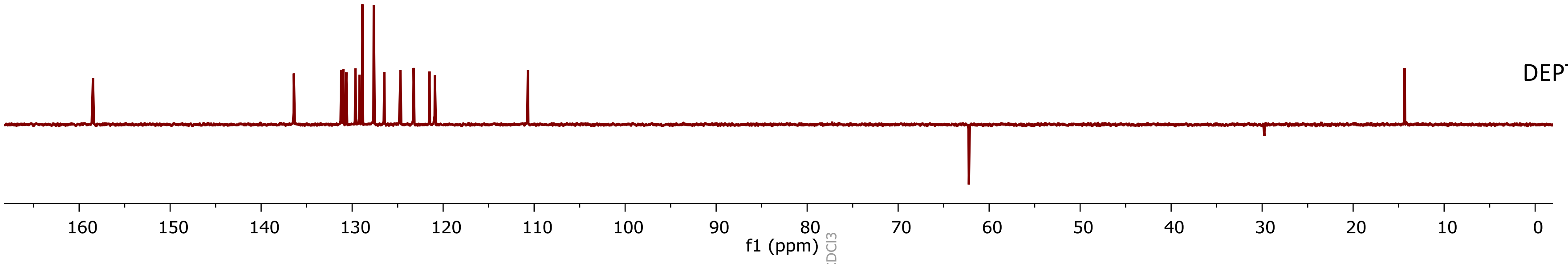

DEPT-135 ($CDCl_3$)

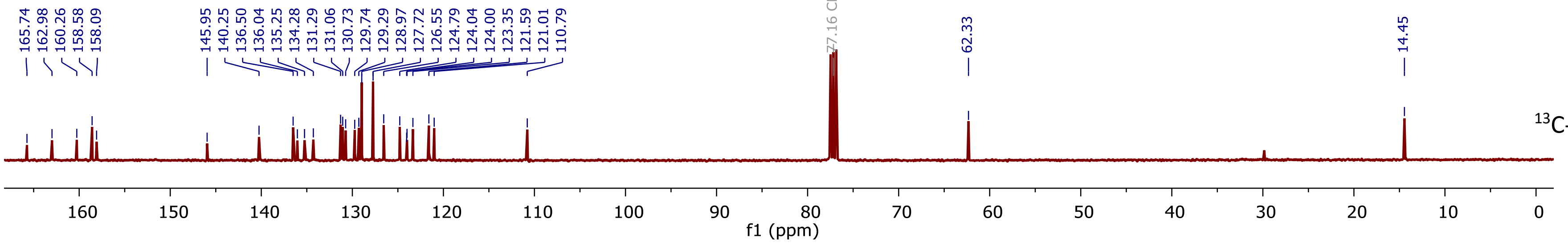

^{13}C-NMR ($CDCl_3$)

EI MS (70eV)

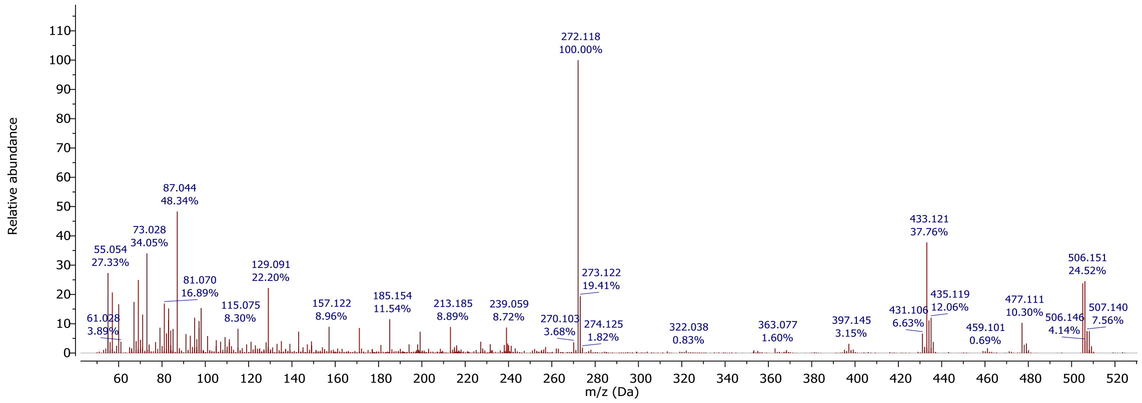

ESI-QTOF (positive ionization)

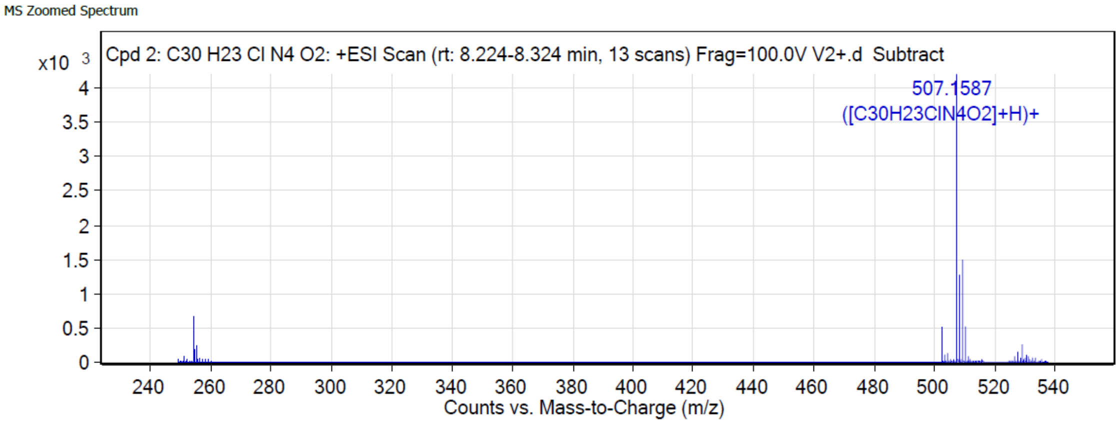

MS Spectrum Peak List

m/z	Calc *m/z*	Diff(ppm)	z	Abund	Formula	Ion
254.0823	254.0828	1.97	2	698	C30H23ClN4O2	(M+2H)+2
254.585	254.5843	-2.84	2	197.81	C30H23ClN4O2	(M+2H)+2
255.0822	255.082	-0.67	2	215.16	C30H23ClN4O2	(M+2H)+2
507.1587	507.1582	-0.96	1	4194.85	C30H23ClN4O2	(M+H)+
508.1616	508.1613	-0.52	1	1314.03	C30H23ClN4O2	(M+H)+
509.157	509.1567	-0.58	1	1522	C30H23ClN4O2	(M+H)+
510.1582	510.159	1.56	1	525.51	C30H23ClN4O2	(M+H)+
511.1611	511.1617	1.2	1	90.95	C30H23ClN4O2	(M+H)+
529.1405	529.1402	-0.61	1	264.16	C30H23ClN4O2	(M+Na)+
531.141	531.1387	-4.47	1	97.29	C30H23ClN4O2	(M+Na)+

IR

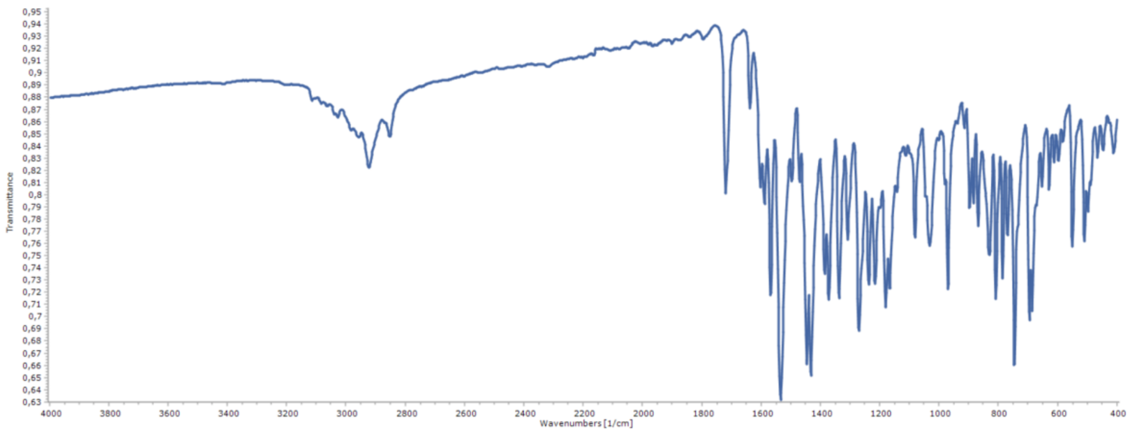

Compound **18a**

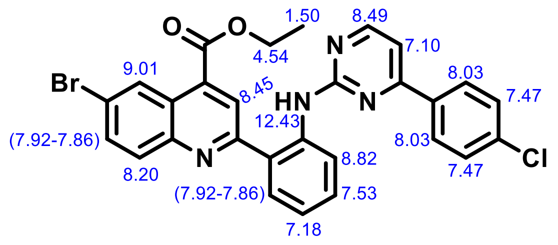

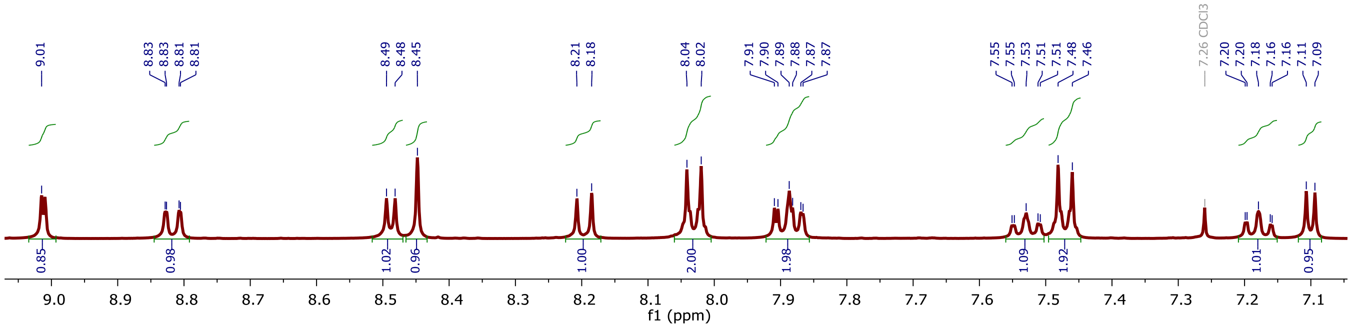

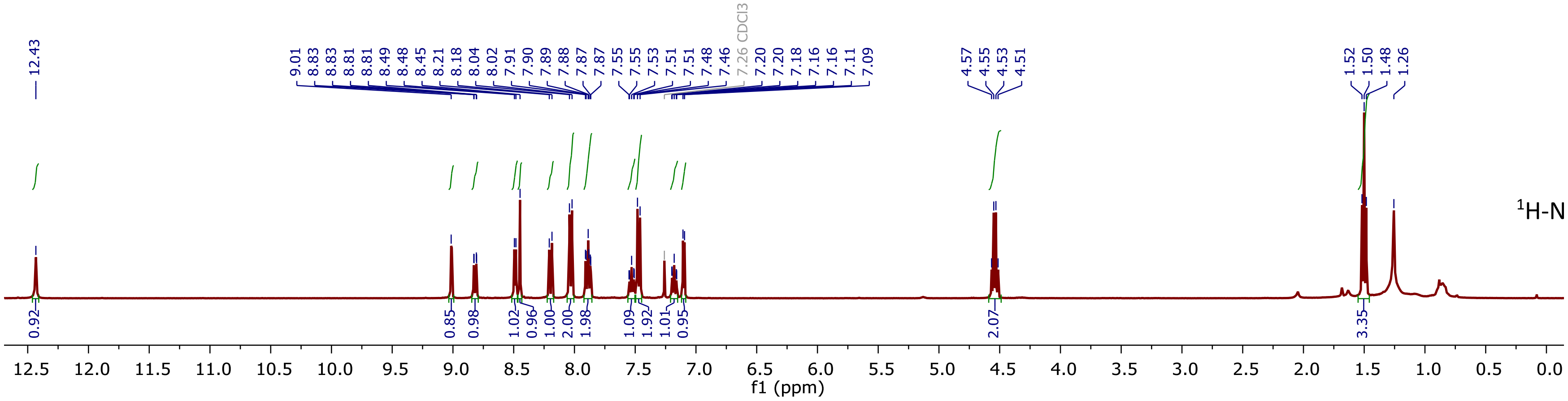

^{1}H-NMR ($CDCl_3$)

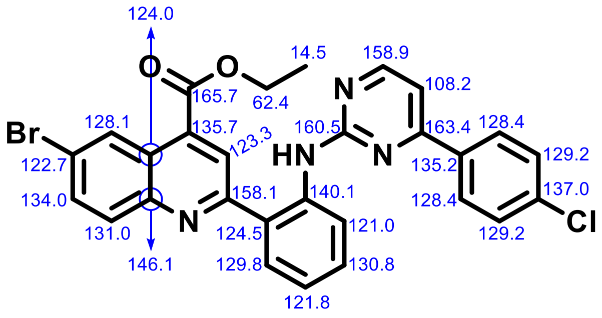

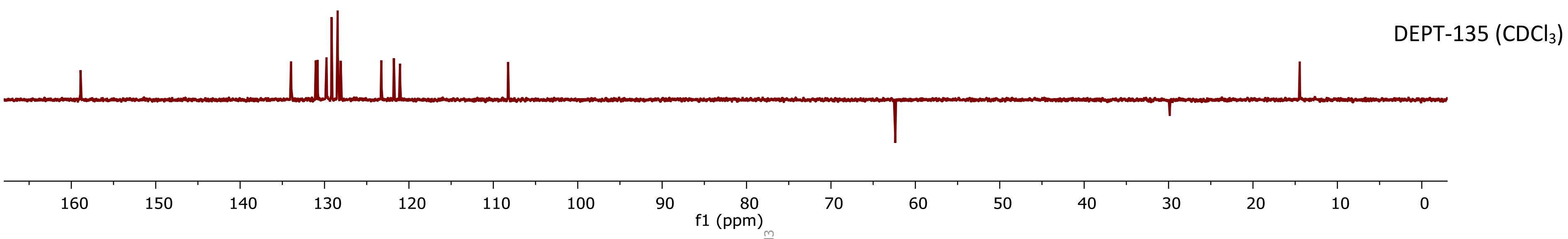
DEPT-135 (CDCl3)
f1 (ppm)

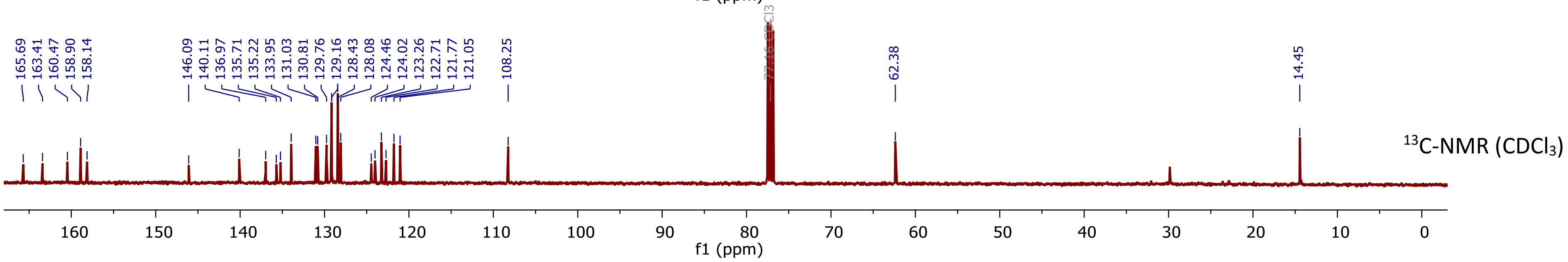
165.69
163.41
160.47
158.90
158.14
146.09
140.11
136.97
135.71
135.22
133.95
131.03
130.81
129.76
129.16
128.43
128.08
124.46
124.02
123.26
122.71
121.77
121.05
108.25
62.38
14.45
13C-NMR (CDCl3)
f1 (ppm)

EI MS (70eV)

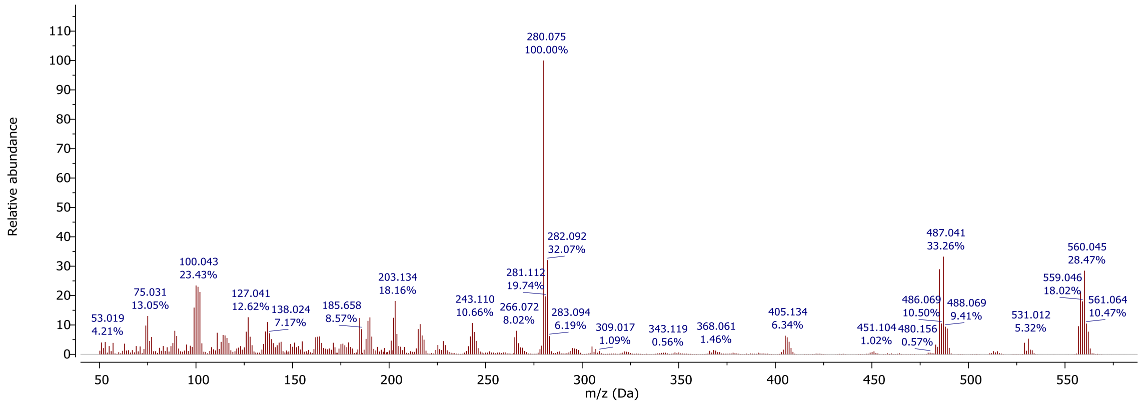

ESI-QTOF (positive ionization)

MS Zoomed Spectrum

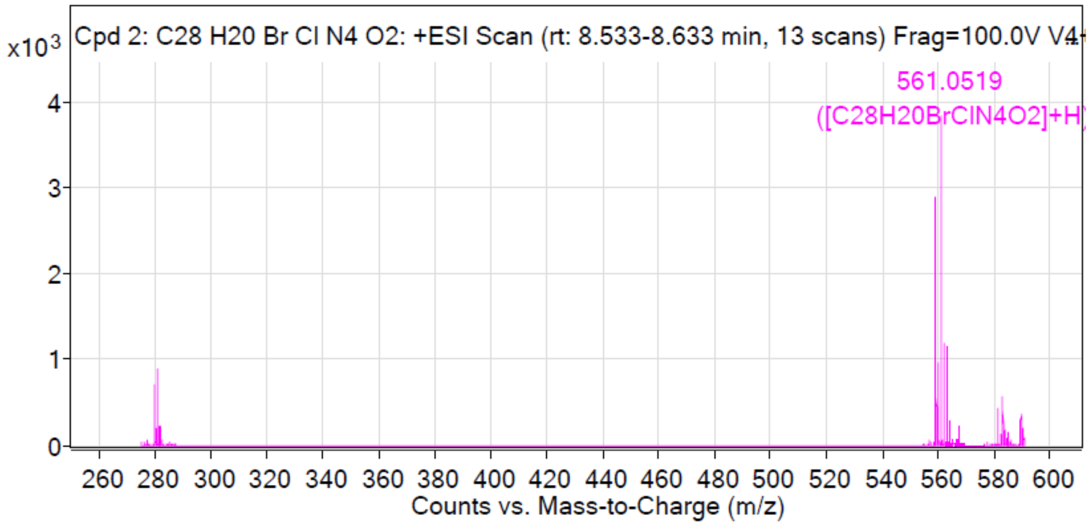

MS Spectrum Peak List

m/z	Calc *m/z*	Diff(ppm)	z	Abund	Formula	Ion
280.0303	280.0302	-0.41	2	710.43	C28H20BrClN4O2	(M+2H)+2
281.0294	281.0292	-0.5	2	905.43	C28H20BrClN4O2	(M+2H)+2
559.053	559.0531	0.1	1	2964.42	C28H20BrClN4O2	(M+H)+
560.0554	560.0562	1.33	1	986.36	C28H20BrClN4O2	(M+H)+
561.0519	561.0512	-1.37	1	3902.9	C28H20BrClN4O2	(M+H)+
562.0535	562.054	0.92	1	1190.65	C28H20BrClN4O2	(M+H)+
563.0493	563.0497	0.66	1	1154.18	C28H20BrClN4O2	(M+H)+
564.0516	564.0518	0.34	1	313.9	C28H20BrClN4O2	(M+H)+
581.0343	581.035	1.31	1	436.42	C28H20BrClN4O2	(M+Na)+
583.0332	583.0331	-0.16	1	578.74	C28H20BrClN4O2	(M+Na)+

IR

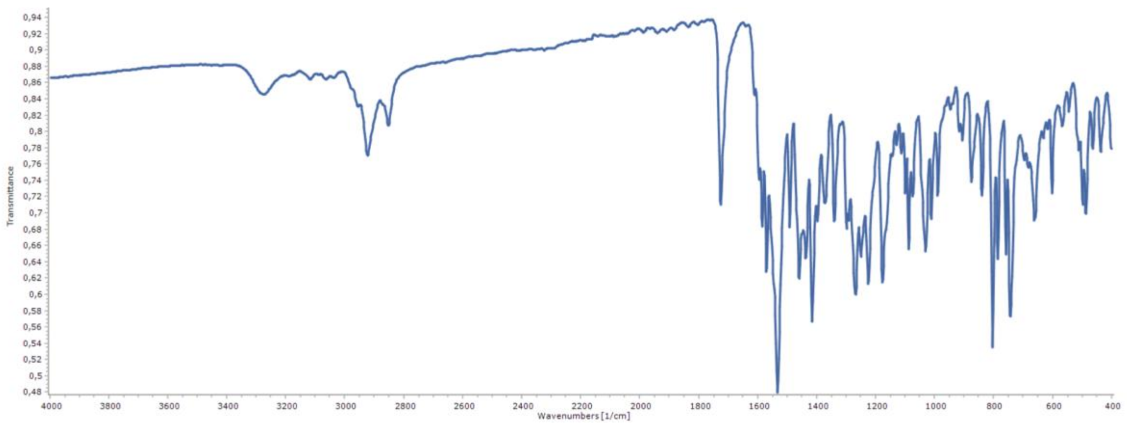

Compound **18b**

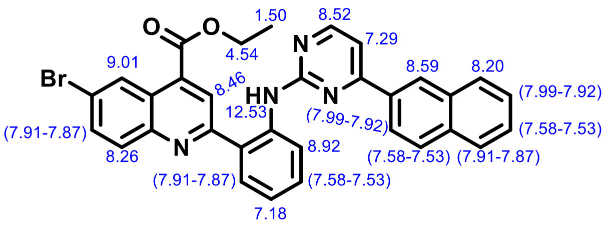

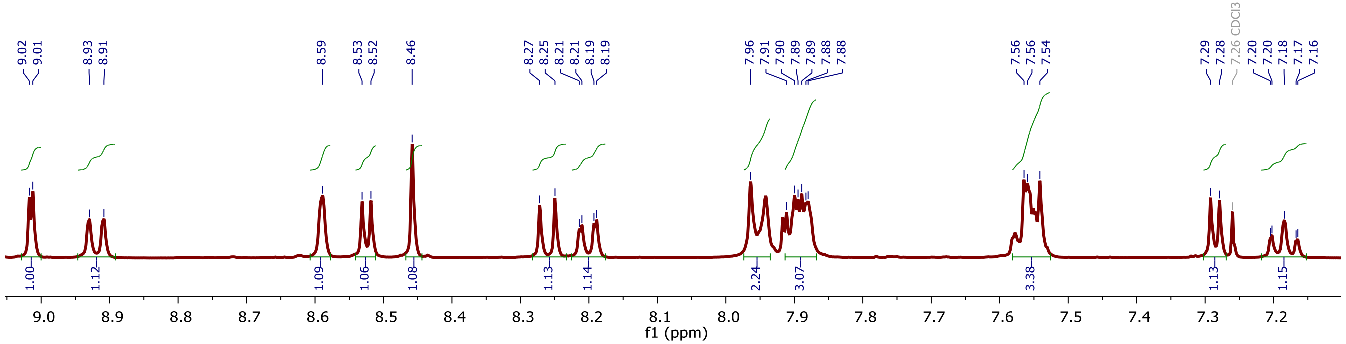

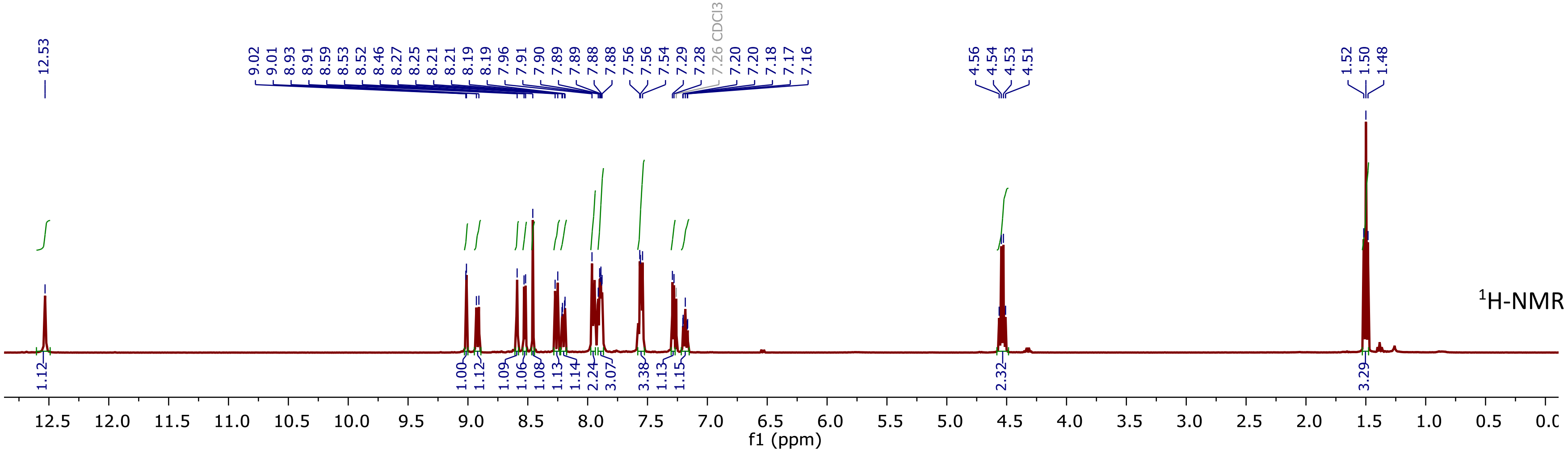

^{1}H-NMR ($CDCl_3$)

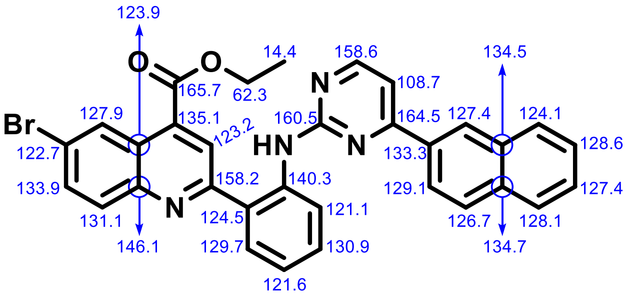
Br
N
O
O
HN
N
N
123.9
14.4
158.6
134.5
165.7
62.3
108.7
127.9
135.1
160.5
164.5
127.4
124.1
122.7
123.2
133.3
128.6
133.9
158.2
140.3
129.1
127.4
131.1
124.5
121.1
126.7
128.1
146.1
129.7
130.9
134.7
121.6

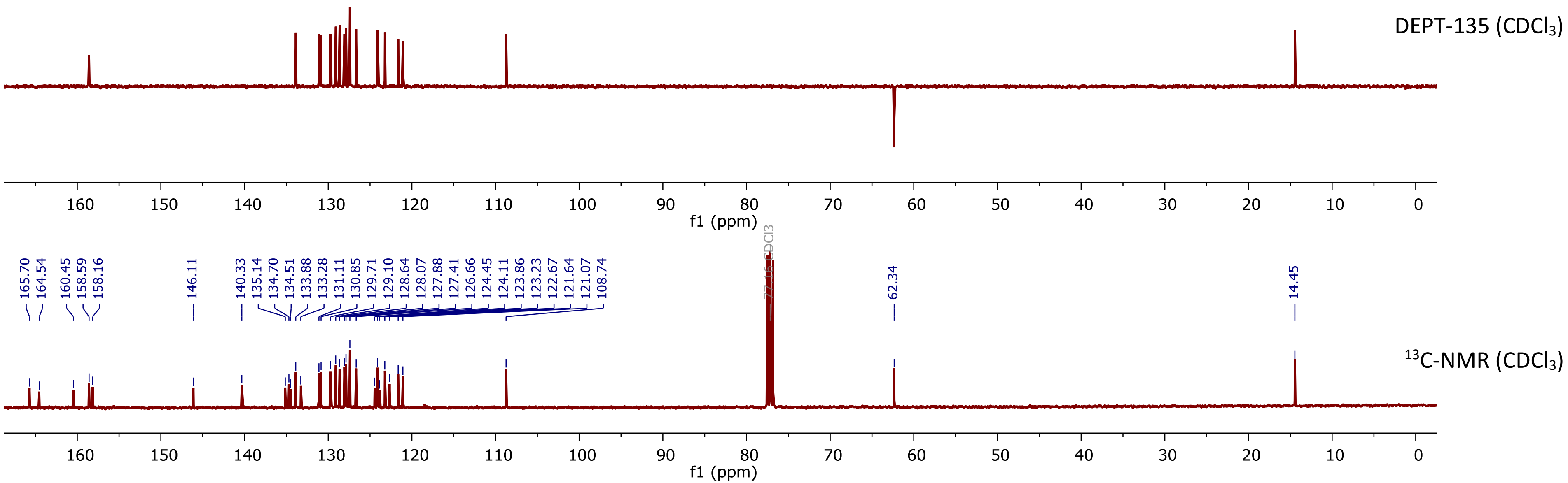
DEPT-135 ($CDCl_3$)
f1 (ppm)
165.70
164.54
160.45
158.59
158.16
146.11
140.33
135.14
134.70
134.51
133.88
133.28
131.11
130.85
129.71
129.10
128.64
128.07
127.88
127.41
126.66
124.45
124.11
123.86
123.23
122.67
121.64
121.07
108.74
77.16 CDCl3
62.34
14.45
^{13}C-NMR ($CDCl_3$)
f1 (ppm)

EI MS (70eV)

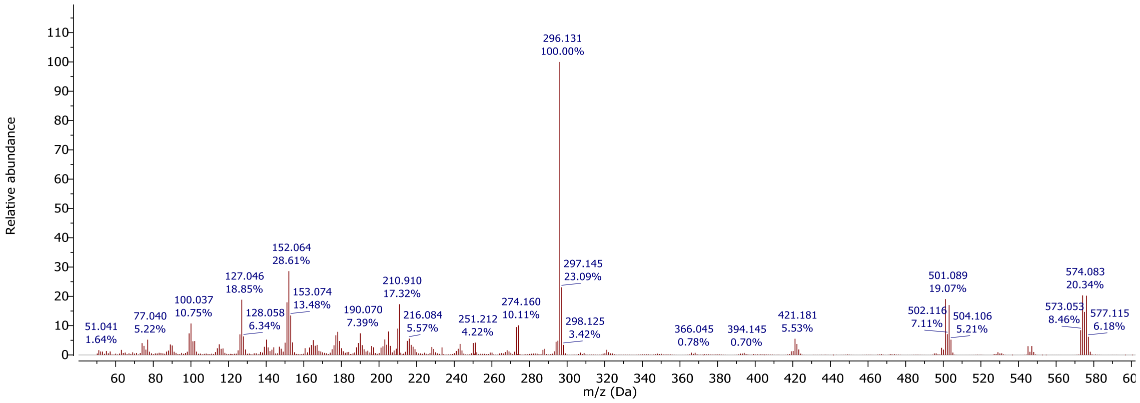

ESI-QTOF (positive ionization)

MS Zoomed Spectrum

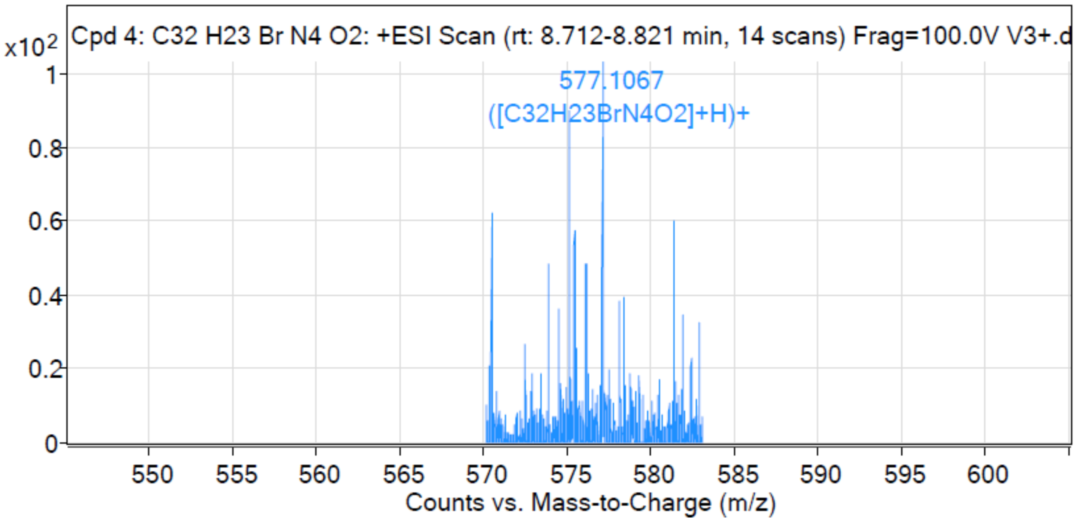

MS Spectrum Peak List

m/z	Calc *m/z*	Diff(ppm)	z	Abund	Formula	Ion
575.1075	575.1077	0.45	1	90.95	C32H23BrN4O2	(M+H)+
576.1155	576.1108	-8.1	1	49.22	C32H23BrN4O2	(M+H)+
577.1067	577.1062	-0.87	1	106.68	C32H23BrN4O2	(M+H)+
578.104	578.109	8.57	1	39.53	C32H23BrN4O2	(M+H)+

IR

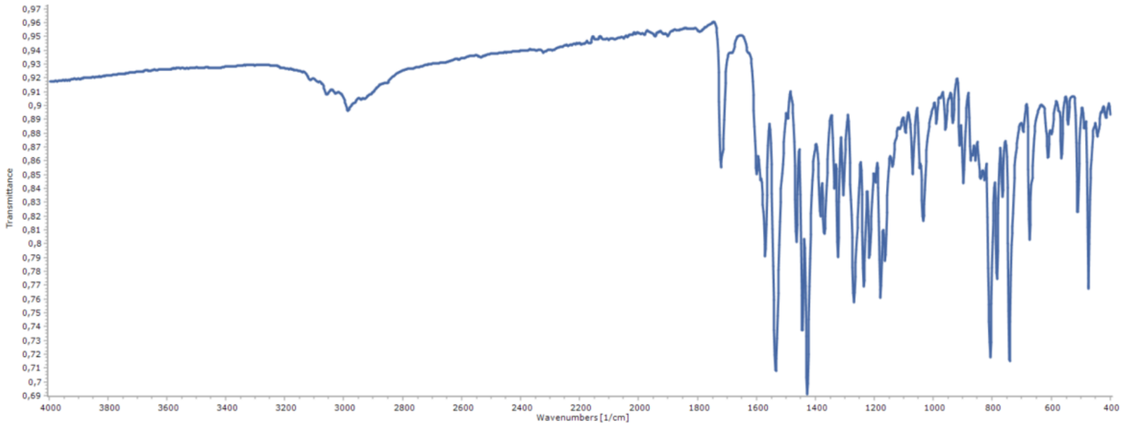

Compound **18c**

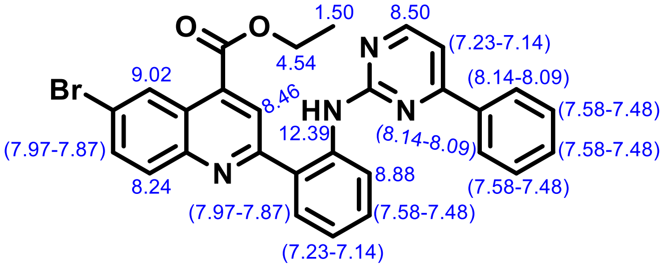

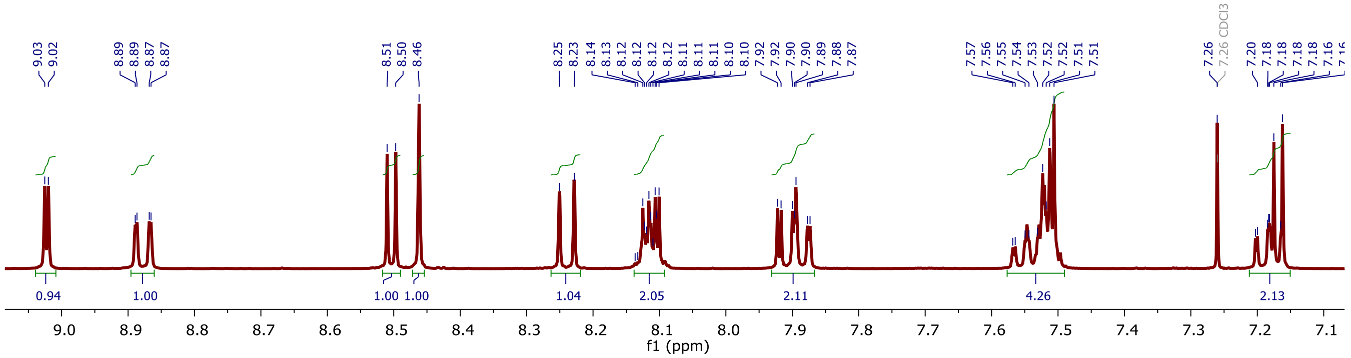

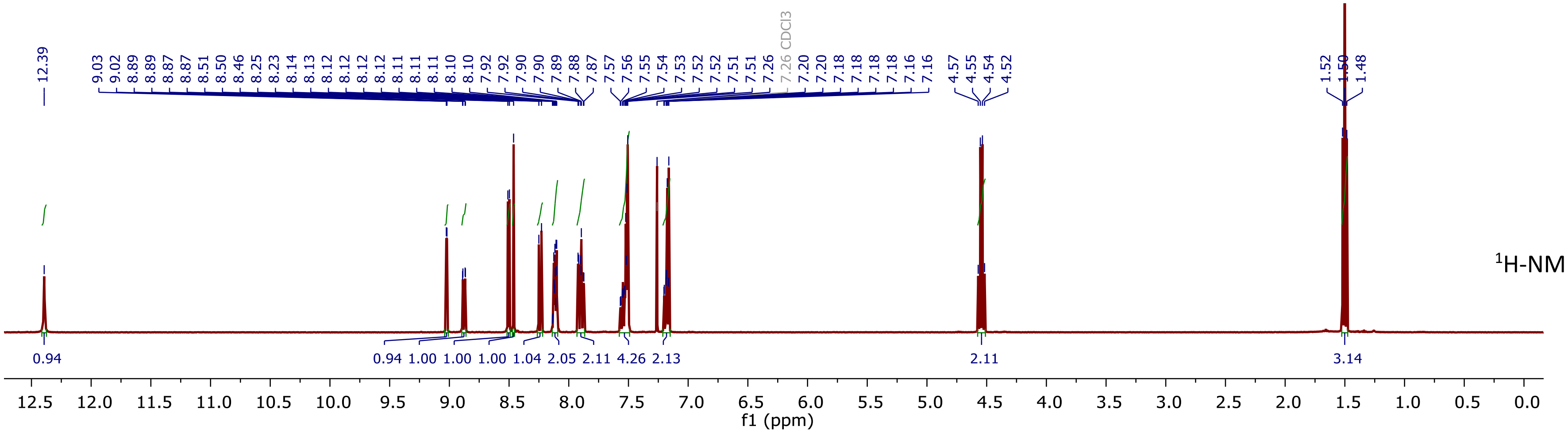

^{1}H-NMR ($CDCl_3$)

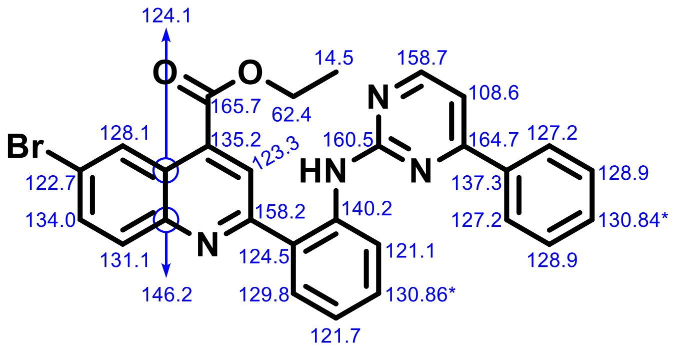

DEPT-135 ($CDCl_3$)

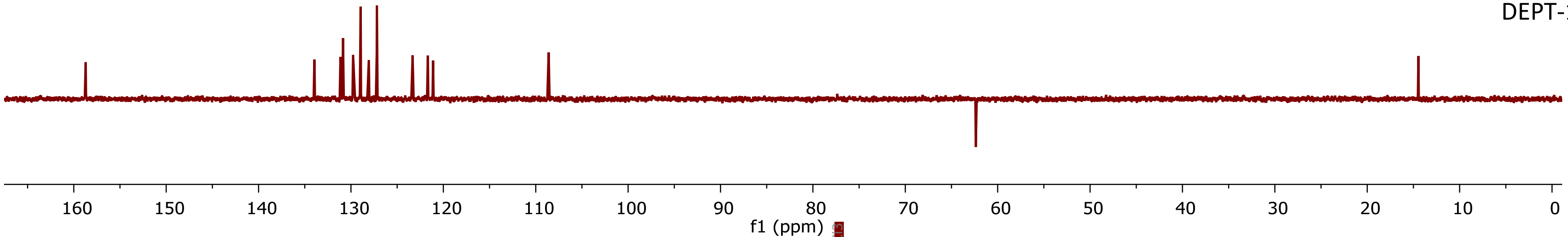

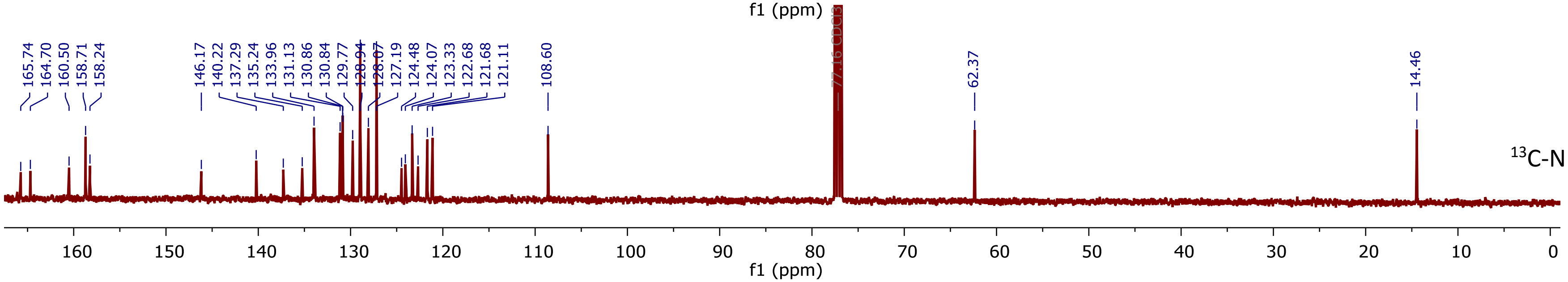

^{13}C-NMR ($CDCl_3$)

EI MS (70eV)

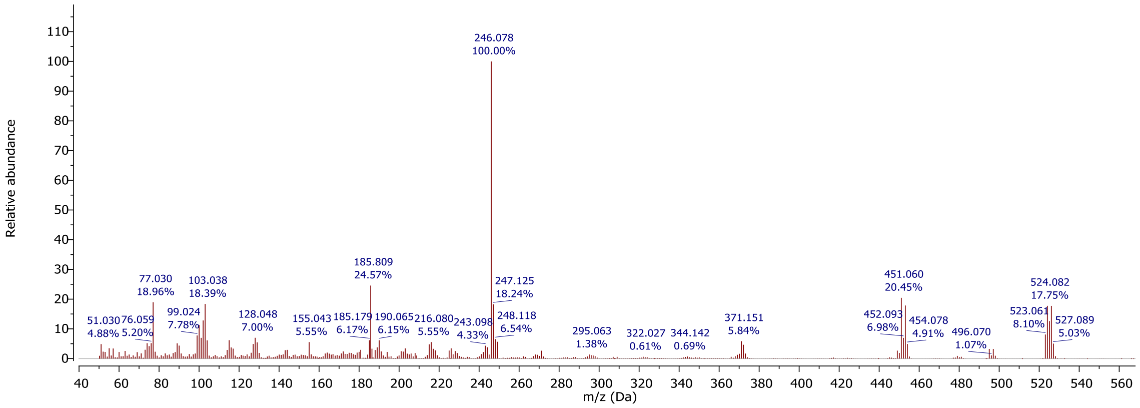

ESI-QTOF (positive ionization)

MS Zoomed Spectrum

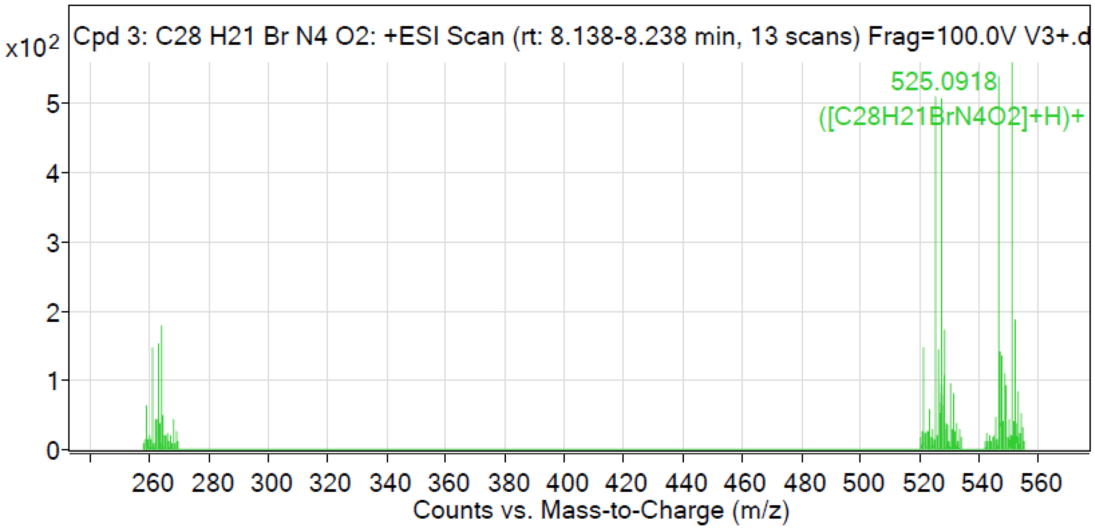

MS Spectrum Peak List

m/z	Calc *m/z*	Diff(ppm)	z	Abund	Formula	Ion
263.0489	263.0497	2.95	2	152.08	C28H21BrN4O2	(M+2H)+2
264.0483	264.0489	2.25	2	180.29	C28H21BrN4O2	(M+2H)+2
264.5542	264.5503	-14.99	2	48.69	C28H21BrN4O2	(M+2H)+2
525.0918	525.0921	0.57	1	515.22	C28H21BrN4O2	(M+H)+
526.0949	526.0952	0.46	1	145.53	C28H21BrN4O2	(M+H)+
527.0899	527.0904	0.99	1	508.34	C28H21BrN4O2	(M+H)+
528.0919	528.0933	2.68	1	178.83	C28H21BrN4O2	(M+H)+
547.0758	547.074	-3.31	1	141.71	C28H21BrN4O2	(M+Na)+
549.0754	549.0724	-5.44	1	92.4	C28H21BrN4O2	(M+Na)+
550.0731	550.0752	3.83	1	44.81	C28H21BrN4O2	(M+Na)+

IR

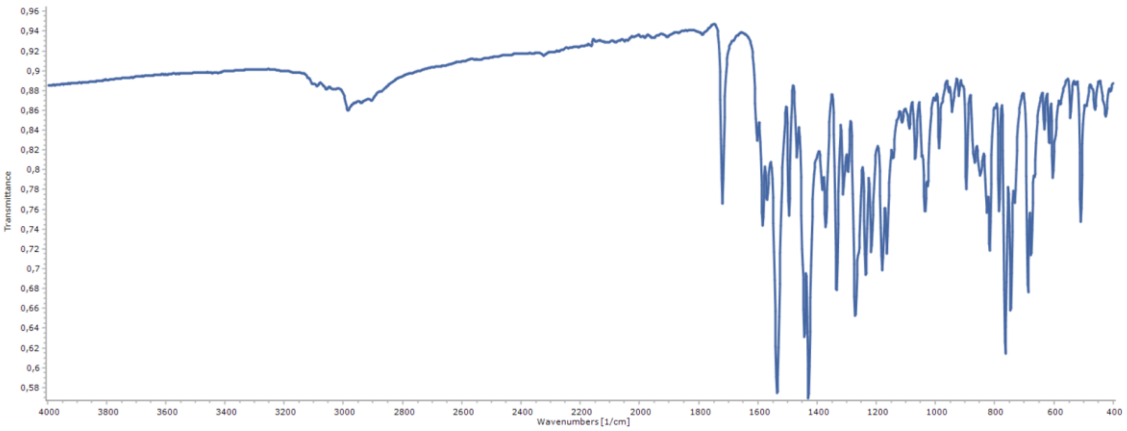

Compound **18d**

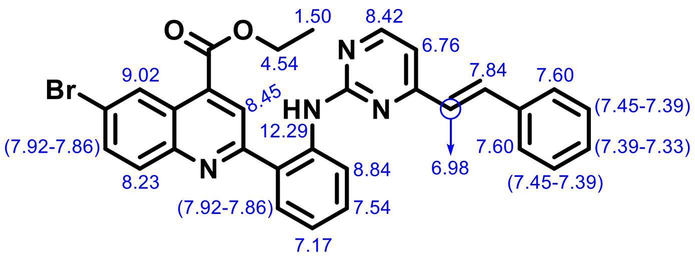

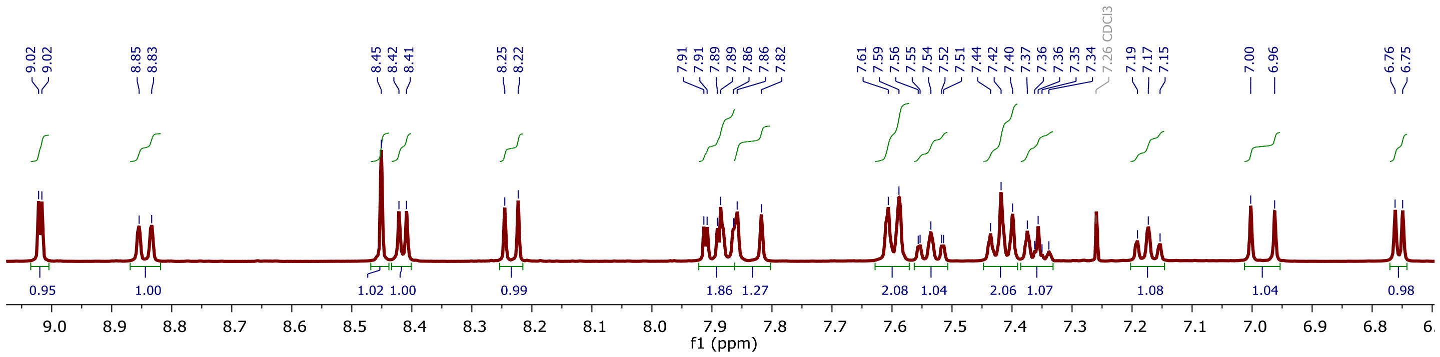

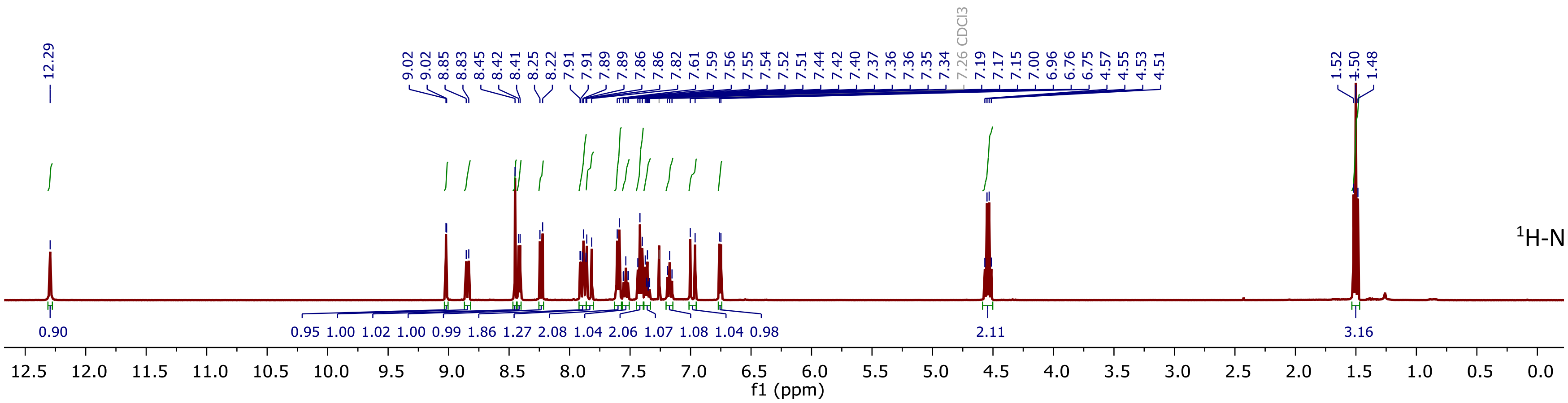

^{1}H-NMR ($CDCl_3$)

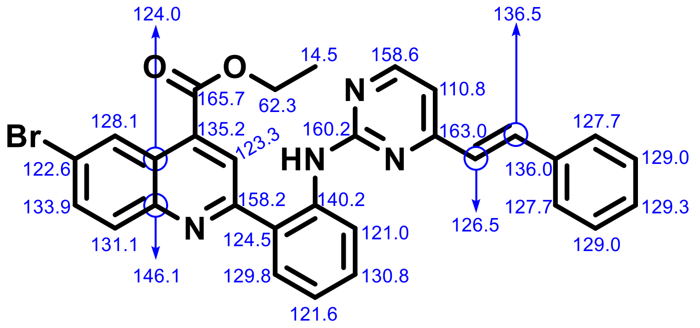

124.0
136.5
14.5
158.6
165.7
62.3
110.8
128.1
135.2
123.3
160.2
163.0
127.7
Br
122.6
HN
136.0
129.0
133.9
158.2
140.2
127.7
129.3
126.5
121.0
131.1
124.5
129.0
146.1
129.8
130.8
121.6

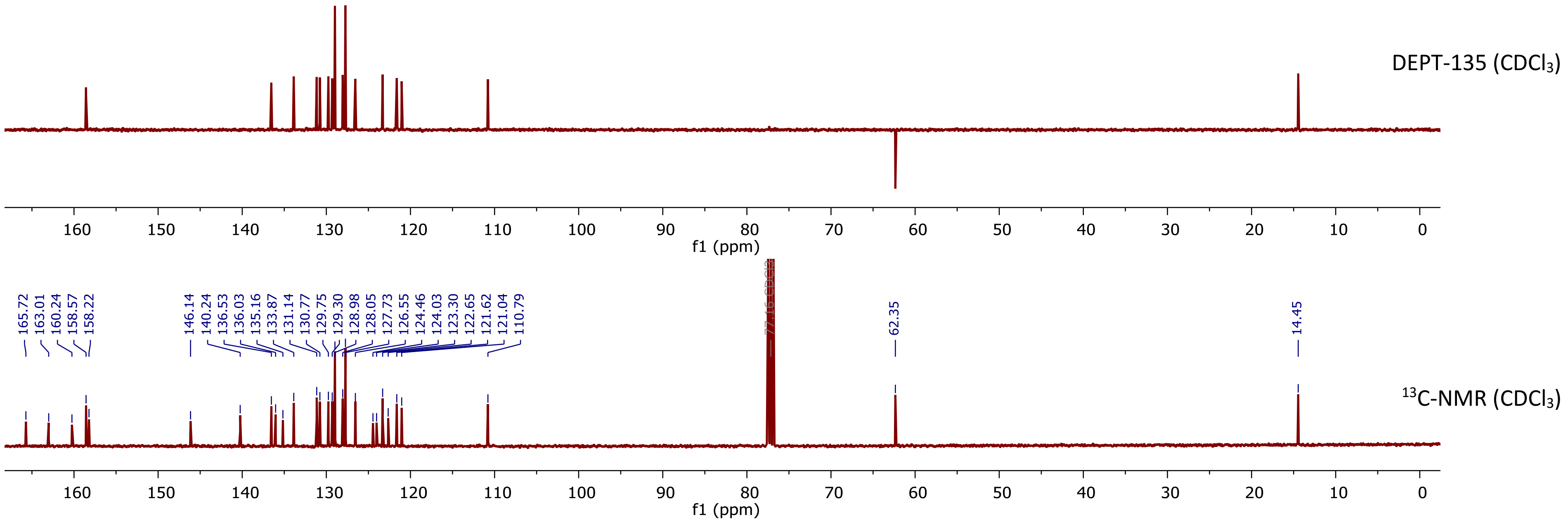

DEPT-135 (CDCl3)
f1 (ppm)
165.72
163.01
160.24
158.57
158.22
146.14
140.24
136.53
136.03
135.16
133.87
131.14
130.77
129.75
129.30
128.98
128.05
127.73
126.55
124.46
124.03
123.30
122.65
121.62
121.04
110.79
77.16 CDCl3
62.35
14.45
13C-NMR (CDCl3)
f1 (ppm)

EI MS (70eV)

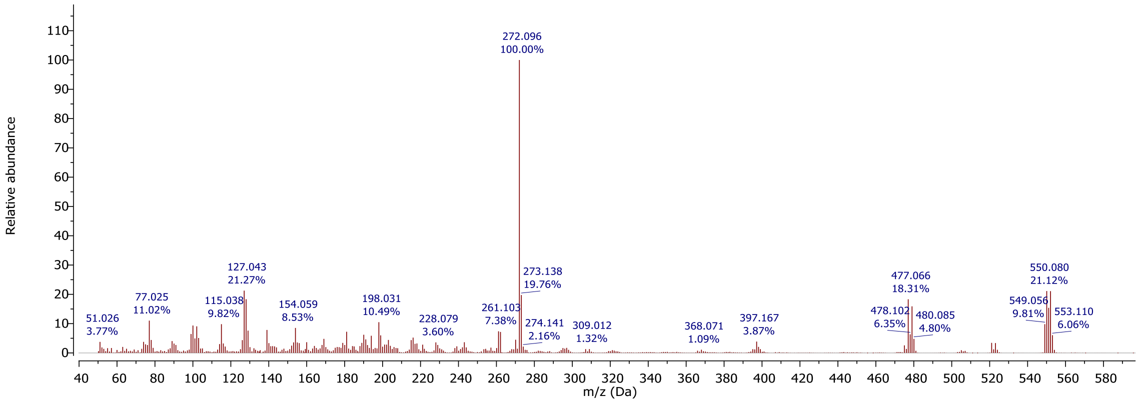

ESI-QTOF (positive ionization)

MS Zoomed Spectrum

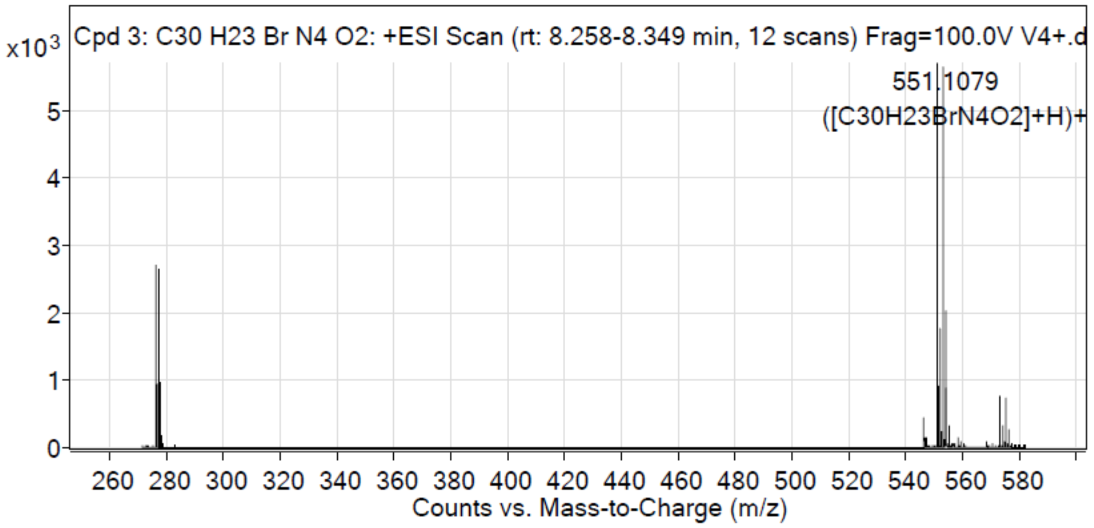

MS Spectrum Peak List

m/z	Calc *m/z*	Diff(ppm)	z	Abund	Formula	Ion
276.0575	276.0575	-0.04	2	2698.16	C30H23BrN4O2	(M+2H)+2
276.5592	276.5591	-0.6	2	953.46	C30H23BrN4O2	(M+2H)+2
277.0567	277.0567	0.1	2	2748.94	C30H23BrN4O2	(M+2H)+2
277.5581	277.5581	-0.1	2	984.77	C30H23BrN4O2	(M+2H)+2
551.1079	551.1077	-0.3	1	5713.22	C30H23BrN4O2	(M+H)+
552.1117	552.1108	-1.56	1	1802.24	C30H23BrN4O2	(M+H)+
553.1063	553.1061	-0.22	1	5708.04	C30H23BrN4O2	(M+H)+
554.1096	554.109	-1.16	1	2043.45	C30H23BrN4O2	(M+H)+
573.0888	573.0897	1.56	1	764.8	C30H23BrN4O2	(M+Na)+
575.0882	575.0881	-0.13	1	753.29	C30H23BrN4O2	(M+Na)+

IR

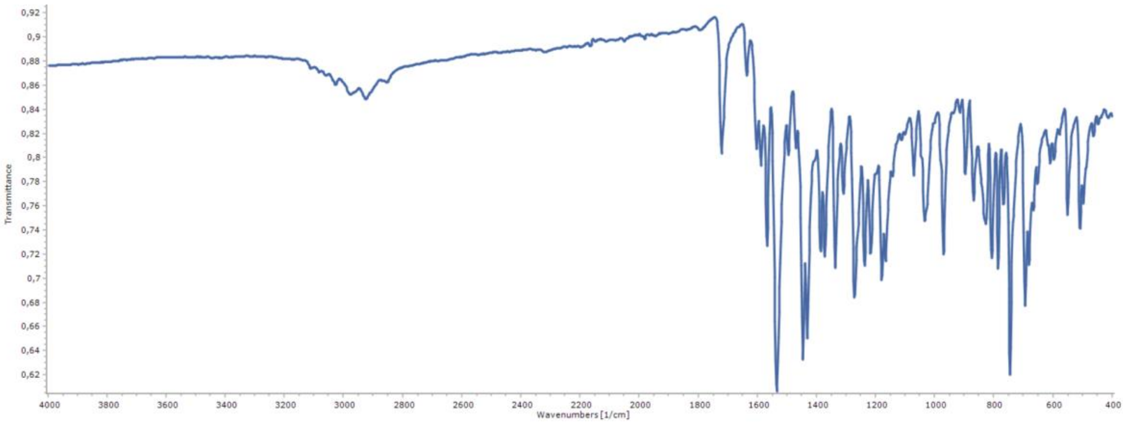

3. Biological assays

3.1. LDHA dose-response inhibition curves

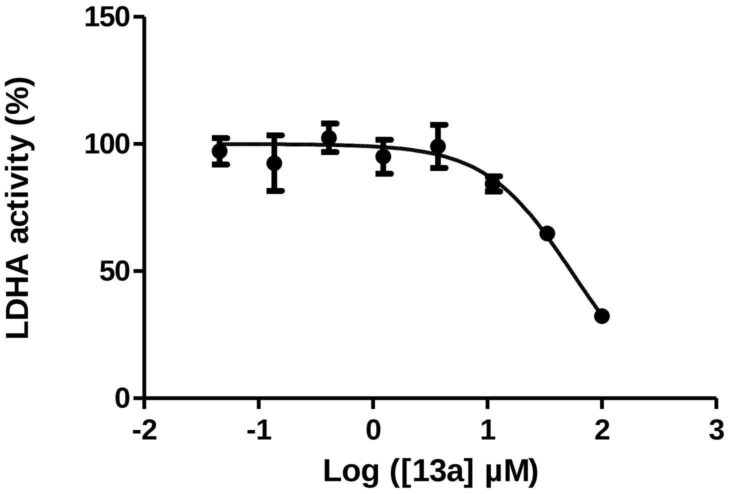

Figure S33. LDHA inhibition curve of compound **13a** (mean ± SD of *n* = 3 replicates)

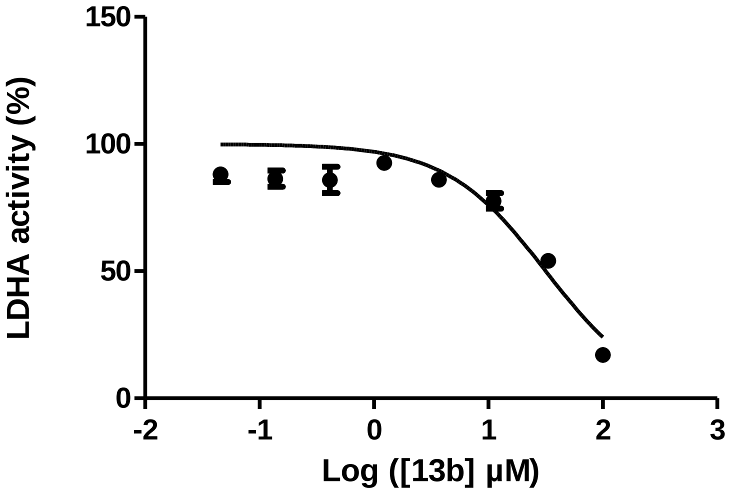

Figure S34. LDHA inhibition curve of compound **13b** (mean ± SD of *n* = 3 replicates)

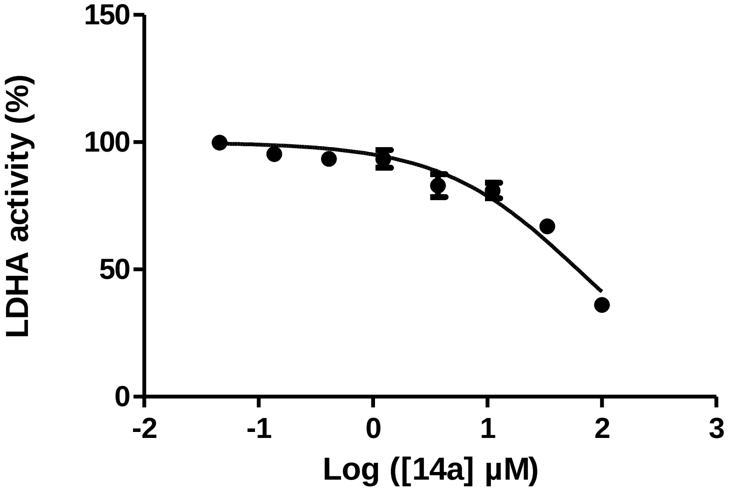

Figure S35. LDHA inhibition curve of compound **14a** (mean ± SD of *n* = 3 replicates)

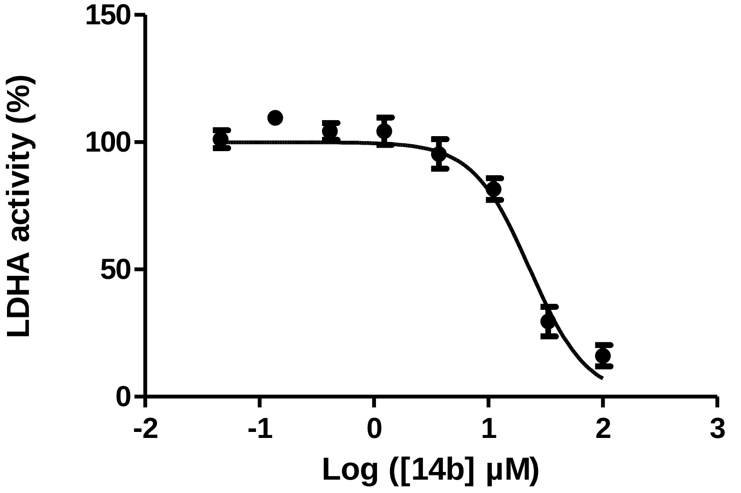

Figure S36. LDHA inhibition curve of compound **14b** (mean ± SD of *n* = 3 replicates)

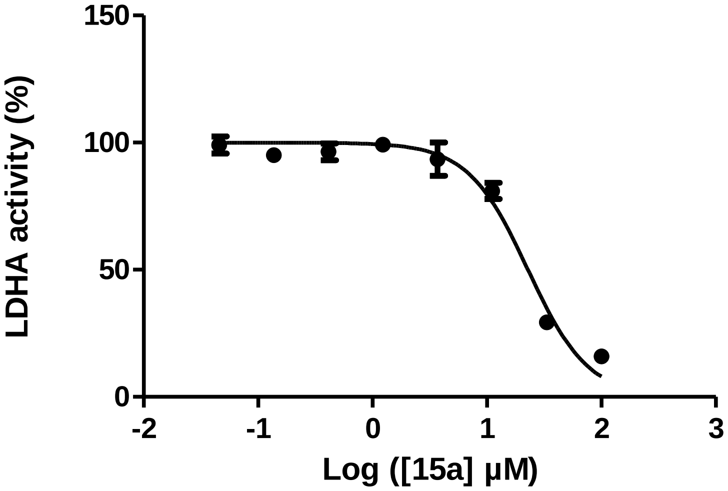

Figure S37. LDHA inhibition curve of compound **15a** (mean ± SD of *n* = 3 replicates)

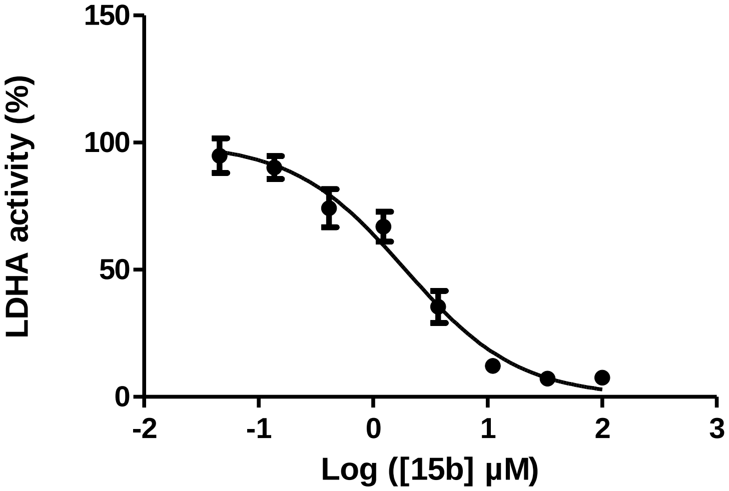

Figure S38. LDHA inhibition curve of compound **15b** (mean ± SD of *n* = 3 replicates)

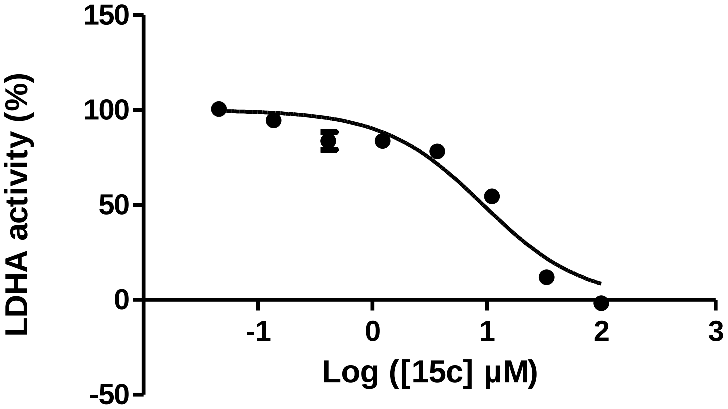

Figure S39. LDHA inhibition curve of compound **15c** (mean ± SD of *n* = 3 replicates)

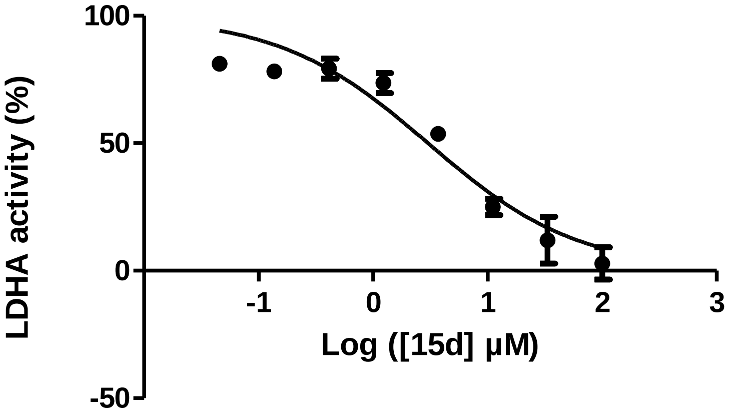

Figure S40. LDHA inhibition curve of compound **15d** (mean ± SD of *n* = 3 replicates)

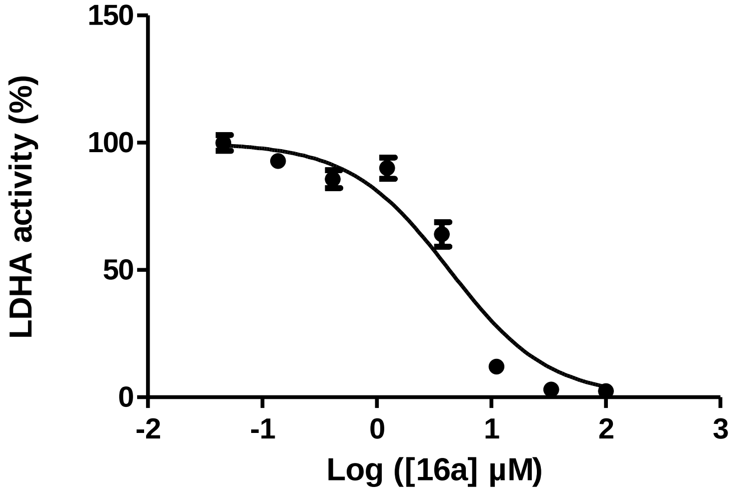

Figure S41. LDHA inhibition curve of compound **16a** (mean ± SD of *n* = 3 replicates)

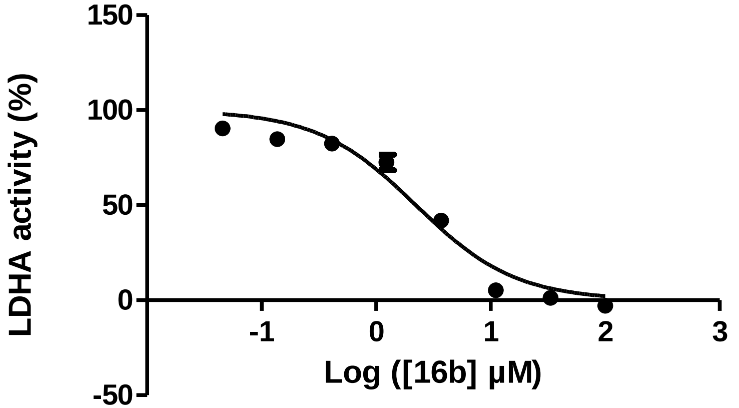

Figure S42. LDHA inhibition curve of compound **16b** (mean ± SD of *n* = 3 replicates)

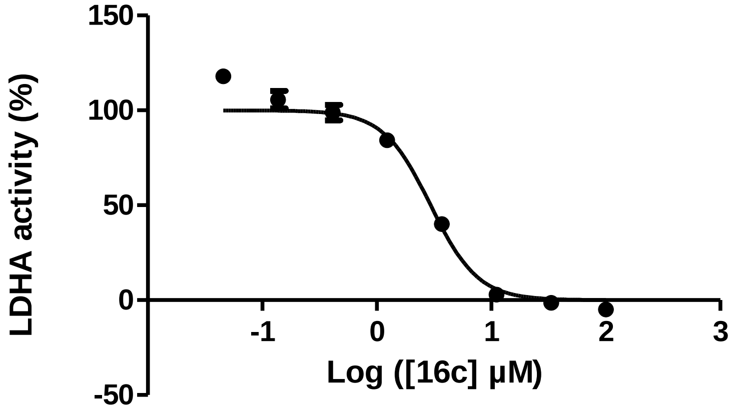

Figure S43. LDHA inhibition curve of compound **16c** (mean ± SD of *n* = 3 replicates)

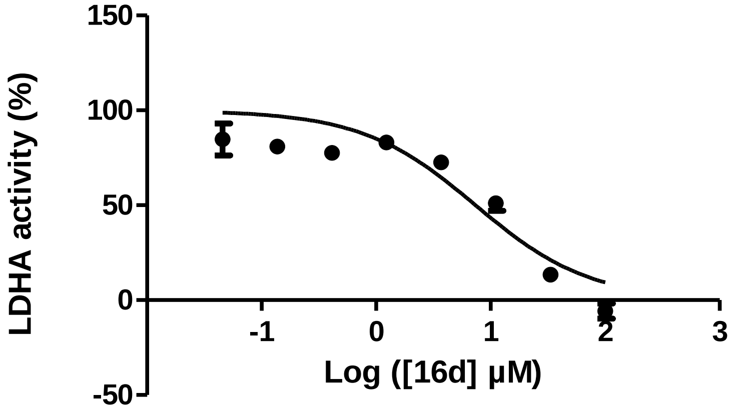

Figure S44. LDHA inhibition curve of compound **16d** (mean ± SD of *n* = 3 replicates)

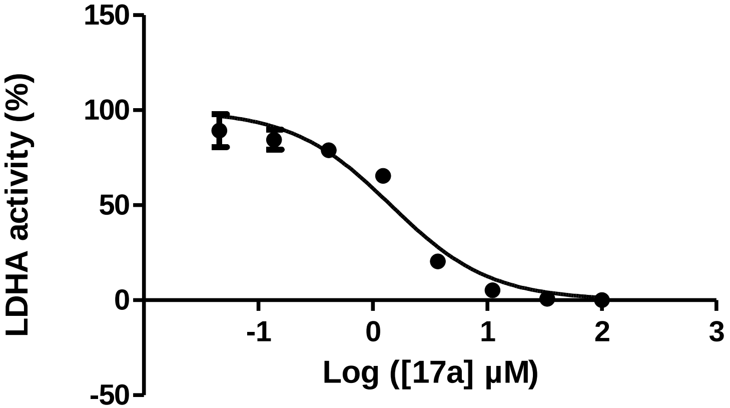

Figure S45. LDHA inhibition curve of compound **17a** (mean ± SD of *n* = 3 replicates)

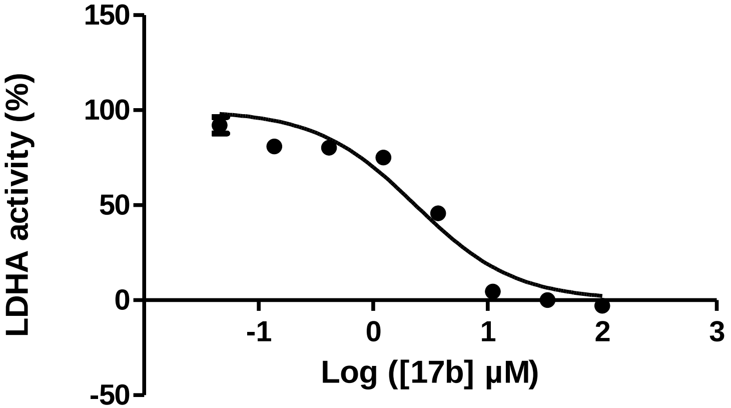

Figure S46. LDHA inhibition curve of compound **17b** (mean ± SD of *n* = 3 replicates)

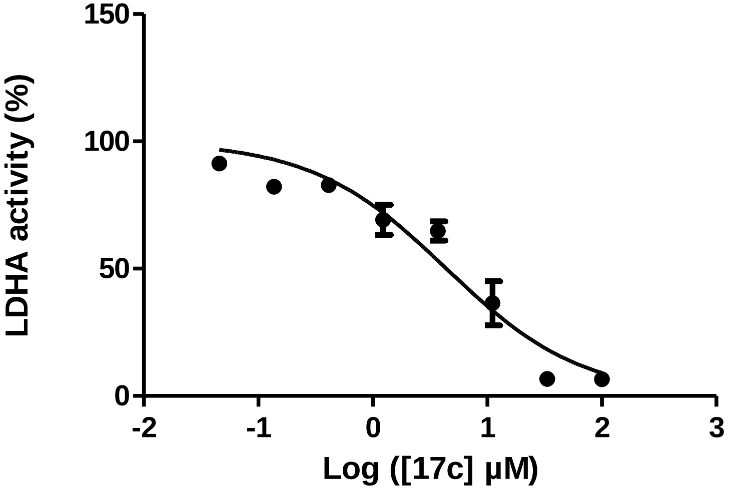

Figure S47. LDHA inhibition curve of compound **17c** (mean ± SD of *n* = 3 replicates)

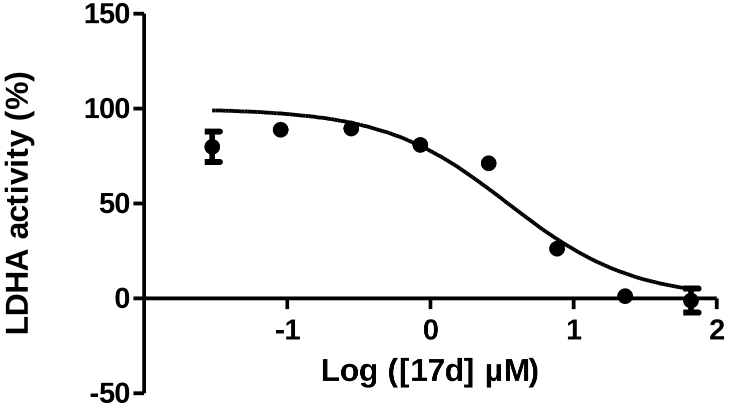

Figure S48. LDHA inhibition curve of compound **17d** (mean ± SD of *n* = 3 replicates)

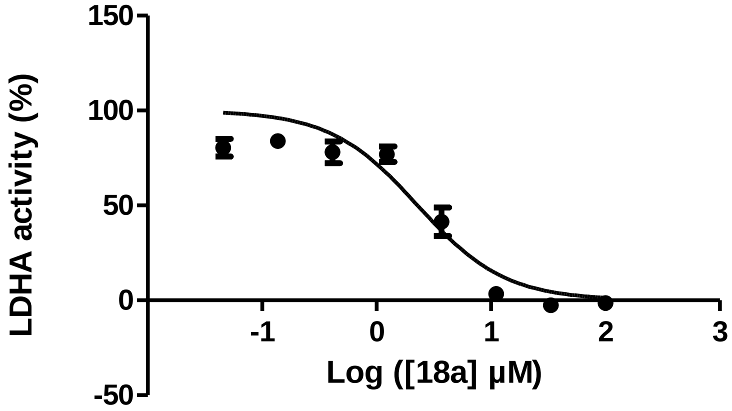

Figure S49. LDHA inhibition curve of compound **18a** (mean ± SD of *n* = 3 replicates)

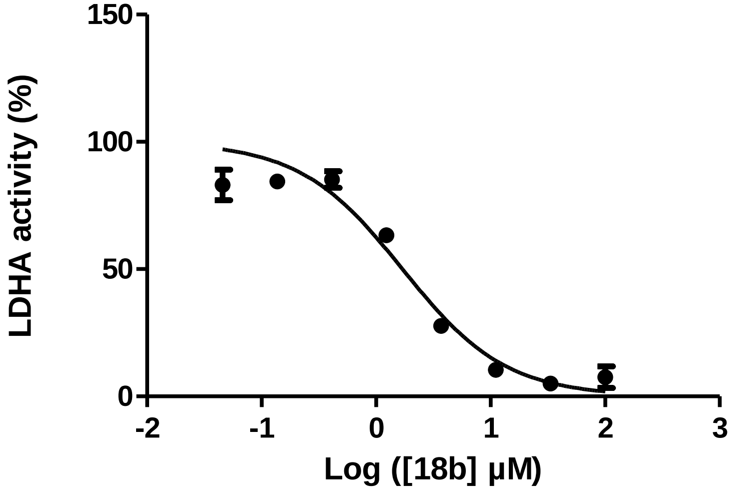

Figure S50. LDHA inhibition curve of compound **18b** (mean ± SD of *n* = 3 replicates)

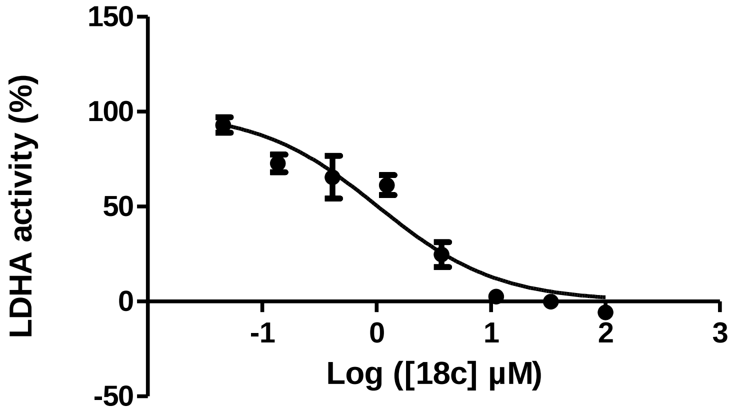

Figure S51. LDHA inhibition curve of compound **18c** (mean ± SD of *n* = 3 replicates)

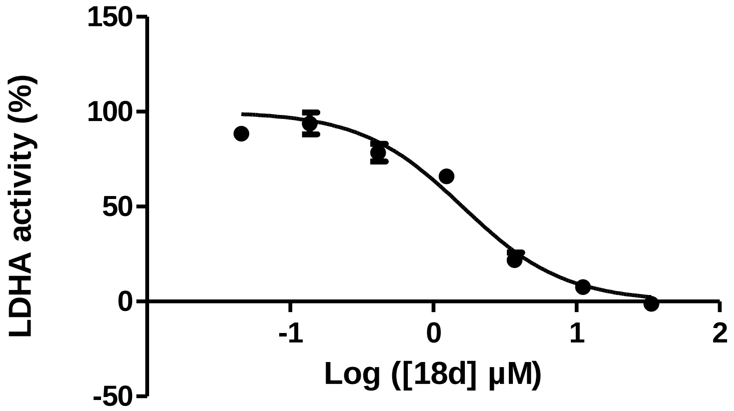

Figure S52. LDHA inhibition curve of compound **18d** (mean ± SD of *n* = 3 replicates)

3.2. LDHB dose-response inhibition curves

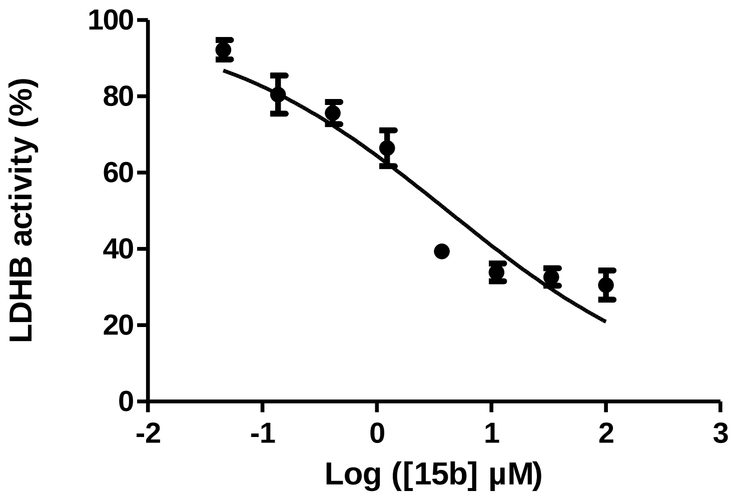

Figure S53. LDHB inhibition curve of compound **15b** (mean ± SD of *n* = 3 replicates)

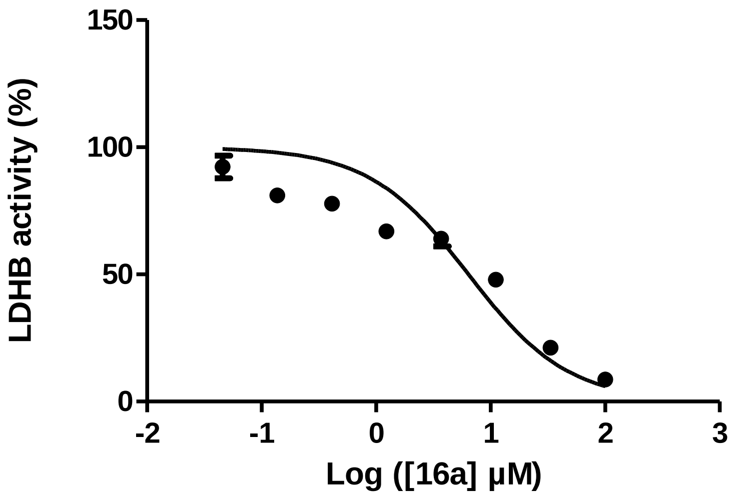

Figure S54. LDHB inhibition curve of compound **16a** (mean ± SD of *n* = 3 replicates)

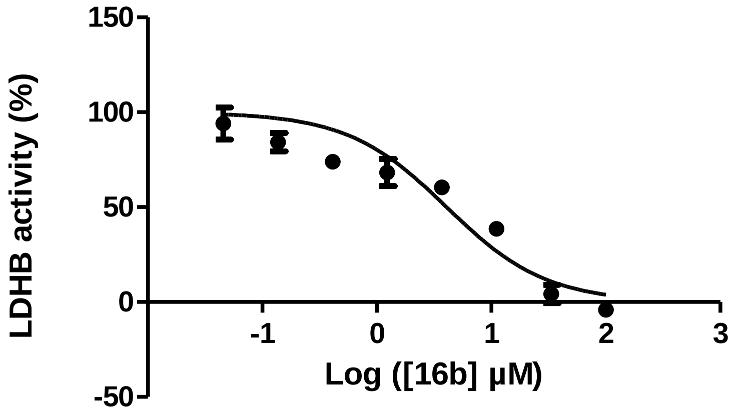

Figure S55. LDHB inhibition curve of compound **16b** (mean ± SD of *n* = 3 replicates)

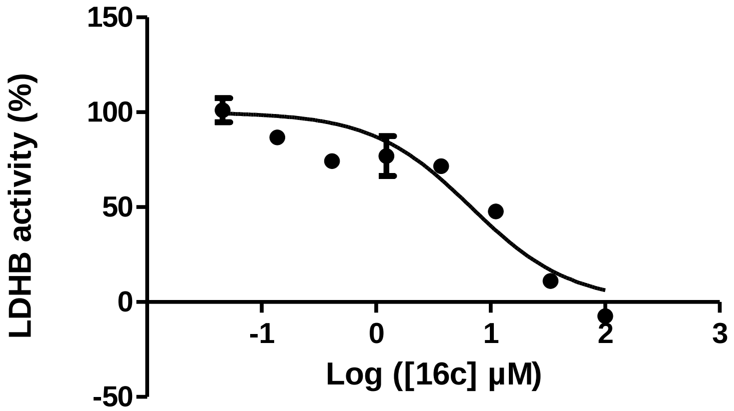

Figure S56. LDHB inhibition curve of compound **16c** (mean ± SD of *n* = 3 replicates)

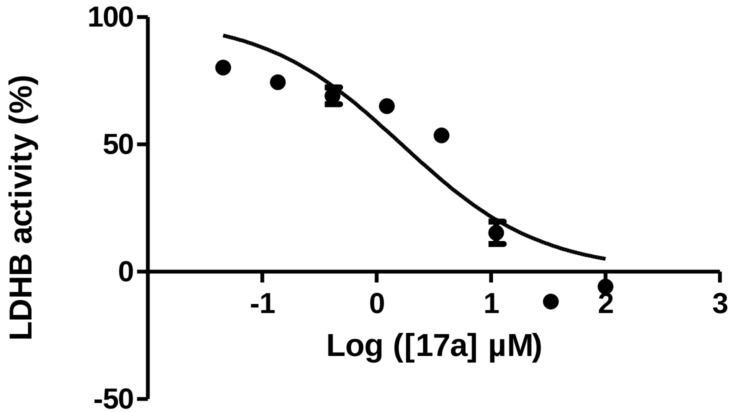

Figure S57. LDHB inhibition curve of compound **17a** (mean ± SD of *n* = 3 replicates)

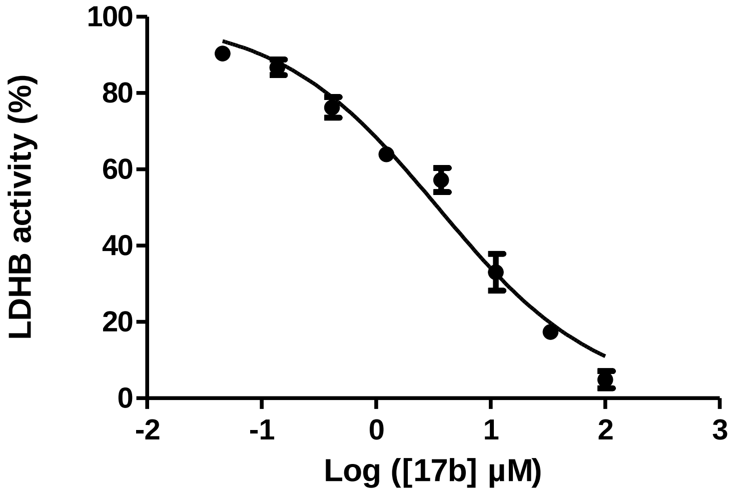

Figure S58. LDHB inhibition curve of compound **17b** (mean ± SD of *n* = 3 replicates)

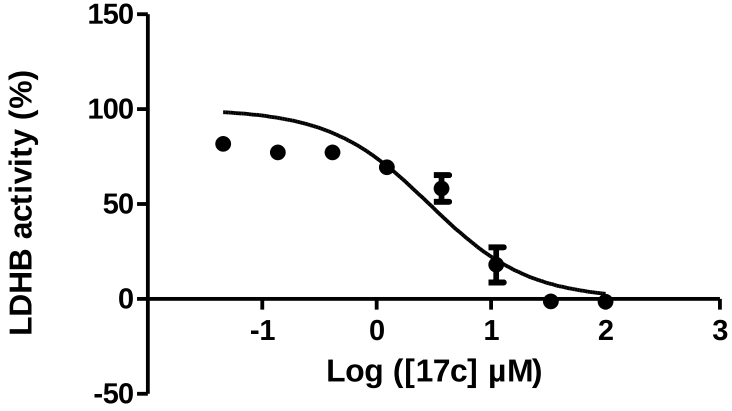

Figure S59. LDHB inhibition curve of compound **17c** (mean ± SD of *n* = 3 replicates)

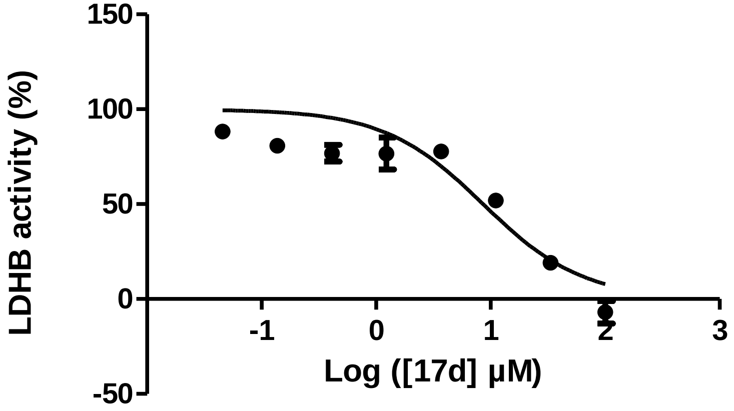

Figure S60. LDHB inhibition curve of compound **17d** (mean ± SD of *n* = 3 replicates)

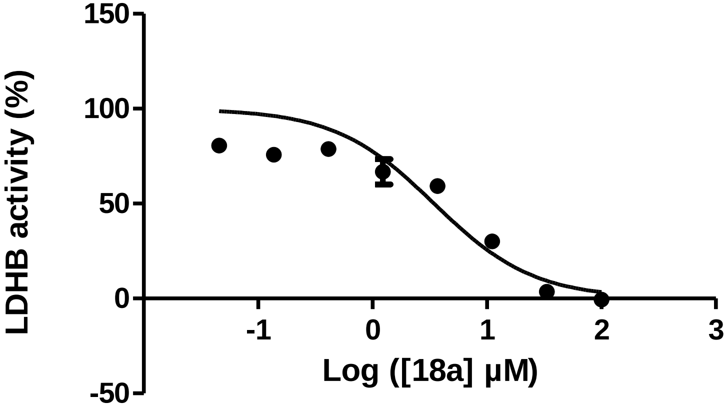

Figure S61. LDHB inhibition curve of compound **18a** (mean ± SD of *n* = 3 replicates)

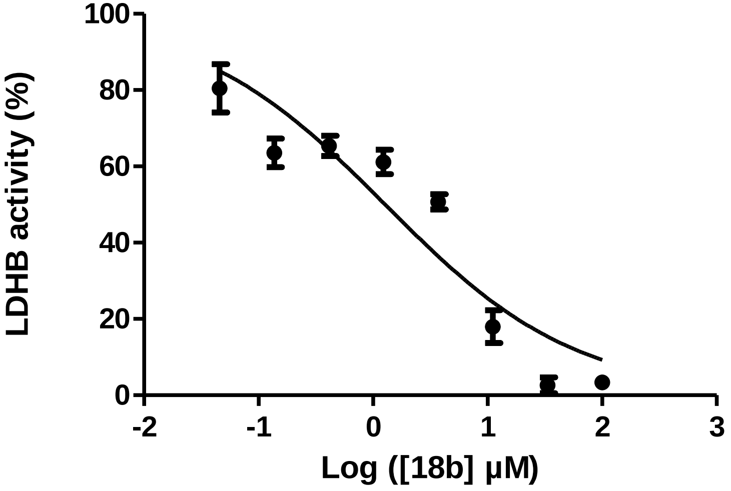

Figure S62. LDHB inhibition curve of compound **18b** (mean ± SD of *n* = 3 replicates)

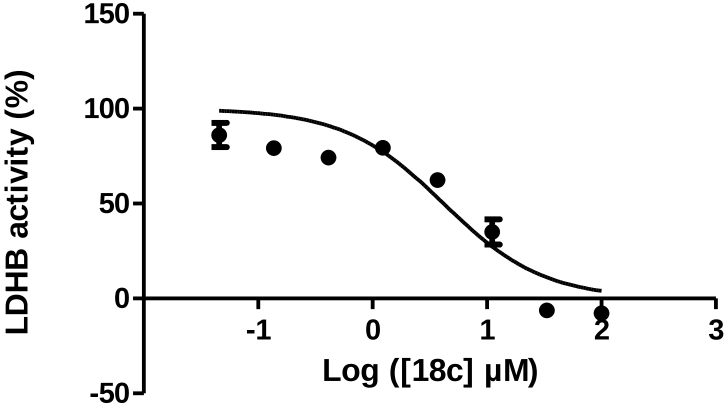

Figure S63. LDHB inhibition curve of compound **18c** (mean ± SD of *n* = 3 replicates)

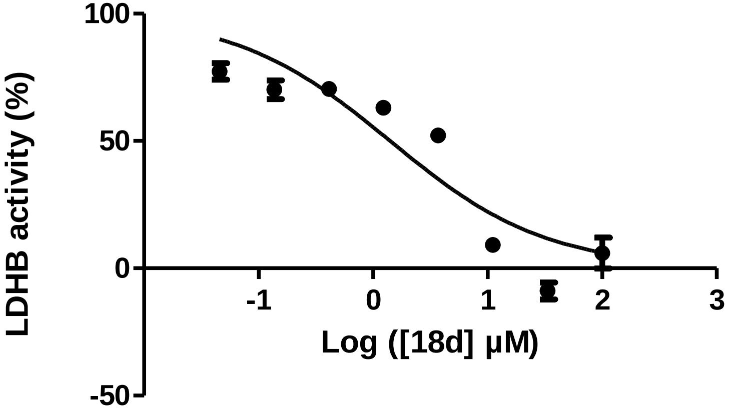

Figure S64. LDHB inhibition curve of compound **18d** (mean ± SD of *n* = 3 replicates)
